# Expanding the limits of the second genetic code with ribozymes

**DOI:** 10.1038/s41467-019-12916-w

**Published:** 2019-11-08

**Authors:** Joongoo Lee, Kenneth E. Schwieter, Andrew M. Watkins, Do Soon Kim, Hao Yu, Kevin J. Schwarz, Jongdoo Lim, Jaime Coronado, Michelle Byrom, Eric V. Anslyn, Andrew D. Ellington, Jeffrey S. Moore, Michael C. Jewett

**Affiliations:** 1 Department of Chemical and Biological Engineering, Northwestern University, Evanston, 60208 IL USA; 2 Department of Chemistry, University of Illinois at Urbana−Champaign, Urbana, 61801 IL USA; 3 Departments of Biochemistry and Physics, Stanford University, Stanford, 94305 CA USA; 4 Departments of Chemical and Biomolecular Engineering, University of Illinois at Urbana−Champaign, Urbana, 61801 IL USA; 5 Department of Chemistry, University of Texas at Austin, Austin, 78712 TX USA; 6 Department of Chemistry and Biochemistry, Institute for Cellular and Molecular Biology, University of Texas at Austin, Austin, 78712 TX USA; 7 The Beckman Institute for Advanced Science and Technology, University of Illinois at Urbana-Champaign, Urbana, IL 61801 USA

**Keywords:** Metabolic engineering, Synthetic biology, Chemical biology, Computational models

## Abstract

The site-specific incorporation of noncanonical monomers into polypeptides through genetic code reprogramming permits synthesis of bio-based products that extend beyond natural limits. To better enable such efforts, flexizymes (transfer RNA (tRNA) synthetase-like ribozymes that recognize synthetic leaving groups) have been used to expand the scope of chemical substrates for ribosome-directed polymerization. The development of design rules for flexizyme-catalyzed acylation should allow scalable and rational expansion of genetic code reprogramming. Here we report the systematic synthesis of 37 substrates based on 4 chemically diverse scaffolds (phenylalanine, benzoic acid, heteroaromatic, and aliphatic monomers) with different electronic and steric factors. Of these substrates, 32 were acylated onto tRNA and incorporated into peptides by in vitro translation. Based on the design rules derived from this expanded alphabet, we successfully predicted the acylation of 6 additional monomers that could uniquely be incorporated into peptides and direct N-terminal incorporation of an aldehyde group for orthogonal bioconjugation reactions.

## Abstract (web summary)

Flexizymes have been used to expand the scope of chemical substrates for ribosome-directed polymerization in vitro. Here the authors deduce design rules of Flexizyme-mediated tRNA acylation that more effectively predict the incorporation of new monomers into peptides.

## Introduction

The translation apparatus is the cell’s factory for protein synthesis, stitching together L-α-amino acid substrates into sequence-defined polymers from a defined genetic template. With protein elongation rates of up to 20 amino acids per second and remarkable precision (fidelity of ~99.99%)^1–3^, the *Escherichia coli* protein biosynthesis system (the ribosome and associated factors necessary for polymerization) possesses an incredible catalytic capability. This has long motivated efforts to understand and harness artificial versions for biotechnology. In nature, however, only limited sets of protein monomers are utilized, thereby resulting in limited sets of biopolymers.

Expanding nature’s repertoire of ribosomal monomers^4–12^ promises to yield distinct kinds of new bio-based products with diverse genetically encoded chemistry. So far, the natural ribosome has been shown capable of selectively incorporating a wide range of chemical substrates into an elongating polymer chain, especially in vitro where greater control and freedom of design is possible^13^. These include α-^14^, β-^15^, γ-^16^, *D*-^17,18^, *N*-alkylated^19,20^, noncanonical amino acids^21^, hydroxy acids^22,23^, peptides^24^, oligomeric foldamer–peptide hybrids^25^, and non-amino carboxylic acids^26,27^. The impact of incorporating such a broad and diverse set of monomers, especially for the site-specific incorporation of noncanonical amino acids into peptides and proteins, has been the production of novel therapeutics, enzymes, and materials^28–34^. For example, the introduction of a benzoic acid at the N-terminus of a peptide led to protein-targeted cyclized *N*-alkyl peptidomimetic drugs^27^. In addition, an aliphatic carbon chain (polyene) attached to an auxiliary amino acid was incorporated into the N-terminus of a peptide to produce a natural product-like macrocyclic peptide^26^. Furthermore, foldamer–dipeptides incorporated into the N-terminus of a peptide have created foldamer–peptide hybrids that undergo cyclization giving enhanced thermal stability^25^. Such pioneering advances motivate interests to pursue further expansion of monomers useful in ribosome-mediated polymerization.

For the selective incorporation of monomers into a growing chain by the ribosome, they must first be covalently attached (or charged) to transfer RNAs (tRNAs), making aminoacyl-tRNA substrates. Multiple strategies have been devised to synthesize such noncanonical aminoacyl-tRNAs, or ‘mis-acylated’ tRNAs. The classical strategy is chemical aminoacylation, which requires the cumbersome synthesis of 5’-phospho-2’-deoxyribocytidylylriboadenosine (pdCpA) dinucleotide, ester coupling with the amino-acid substrate, and enzymatic ligation (e.g., T4 RNA ligase) with a truncated tRNA^35–39^. Unfortunately, chemical aminoacylations are laborious and technically difficult, often giving poor results in translation owing to the generation of a cyclic tRNA by-product, which inhibits ribosomal peptide synthesis^40^. Another strategy is to engineer protein enzymes called aminoacyl-tRNA synthetases (aaRS), which naturally charge canonical amino acids to tRNAs, by directed evolution^41–50^. However, aaRSs have limited promiscuity for noncanonical chemical substrates, and are generally confined to a narrow range of amino-acid analogs that resemble natural ones.

**Fig. 1:**
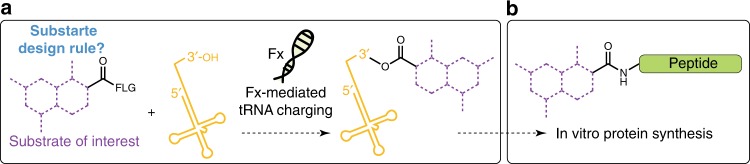
Expanding the chemical substrate scope of flexizymes for genetic code reprogramming. **a** Flexizyme (Fx) recognizes the 3’-CCA sequence of tRNAs^64^ and catalyzes the acylation of tRNA using acid substrates. We seek to develop substrate design rules for flexizyme-mediated acylation reactions that expand the scope of chemical substrates used in ribosome-directed polymerization. **b** An *Escherichia coli* cell-free protein synthesis system reconstituted from the purified wild-type translational machinery (PURExpress) is used to produce peptide^25,65^ containing noncanonical acid substrates. This approach for incorporating noncanonical monomers at the N-terminus of peptides is well established. Flexizyme-Leaving Group (FLG) alternatives include CME, DNBE, and ABT

More recently, an alternative approach to produce mis-acylated tRNAs that uses an RNA enzyme known as flexizyme (Fx) was developed. This flexible and powerful approach, pioneered by Suga and colleagues, is capable of aminoacylating the 3′-OH of an arbitrary tRNA^51^ (Fig. 1) with activated esters^52–55^. Fx not only is important for making a second genetic code, but also serves as a modern experimental surrogate for the ancient establishment of the genetic code, one that may be closer to an ‘RNA world.’^56,57^ Through directed evolution and sequence optimization, three different Fxs (eFx, dFx, and aFx)^5^ have been developed to recognize specific combinations of substrate:activating group. A crystallographic study^58^ elucidated that an aryl group either on the substrate side chain or leaving group is crucial for substrate interaction with the catalytic binding pocket of Fx. For example, eFx acylates tRNA with cyanomethyl ester (CME)-activated acids containing aryl functionality, whereas dFx recognizes dinitrobenzyl ester (DNBE)-activated non-aryl acids^59^. For substrates that lack an aryl group or have poor solubility owing to the presence of DNBE, aFx has been developed recognizing a (2-aminoethyl)amidocarboxybenzyl thioester (ABT)^60^ leaving group, which provides the required aryl group and better aqueous solubility.

The unique potential of the flexizyme approach is the broad scope of monomers that are successfully charged, limited by the side-chain stability toward the conditions of the acylation reaction (or suitably protected/deprotected in the case of reactive side chains), enabling the reassignment of a specific codon to an amino acid de novo. As such, the development of flexizyme has significantly expanded the known permissible space of monomers used in translation by genetic code reprogramming. However, to date, design rules for flexizyme-mediated charging, that may more effectively guide the search for noncanonical monomers have remained not fullydefined. To expand the available design space for template-guided polymerization by the ribosome to polymers beyond polypeptides or polyesters, efforts to explore constraints that limit the scope of noncanonical monomer diversity permissible to both flexizyme-mediated charging and translation by the ribosome are needed.

Here, we set out to fill this gap in knowledge by systematically expanding the range of chemical substrates for flexizyme-catalyzed acylation followed by translation using natural ribosomes (Fig. 1). Initially, we synthesize a repertoire of 37 phenylalanine derivatives, benzoic acid derivatives, heteroaromatic monomers, and aliphatic monomers that are designed based on known compatible scaffolds. We intentionally choose potential substrates that feature chemical moieties inaccessible to natural ribosomally synthesized peptides or their post-translationally modified derivatives. After chemical synthesis of the activated esters, we assess the ability of flexizyme charging of these substrates to tRNAs by varying pH and time to create optimized acylation conditions. We find that 32 of the 37 substrates are charged to tRNAs from which three substrate design rules supported by computational modeling emerged. Next, we examine the competency of the resulting tRNA-monomers for ribosomal incorporation using  the commercially available PURExpress cell-free translation system. It is found that N-terminal incorporation of noncanonical monomers into peptides from substrate–tRNA^fMet^ complexes is possible for 32 of the substrates by wild-type ribosomes, however, incorporation into the C-terminus of peptides is not. Finally, we ask if the substrate design rules predictably guide the search for new noncanonical monomers when peptides are produced for bioconjugation reactions. To do this, we design and synthesize an additional six substrates, each of which was sucessfully charged by flexizyme as predicted by the design rules. Each monomer acylated by flexizyme is incorporated into the N-terminus of a peptide, with which we demonstrate hydrazine-aldehyde bioconjugation chemistry that is applicable to a broad set of proteins^61–63^.

## Results

### Expanding the substrate repertoire for Fx-mediated acylation

**Fig. 2:**
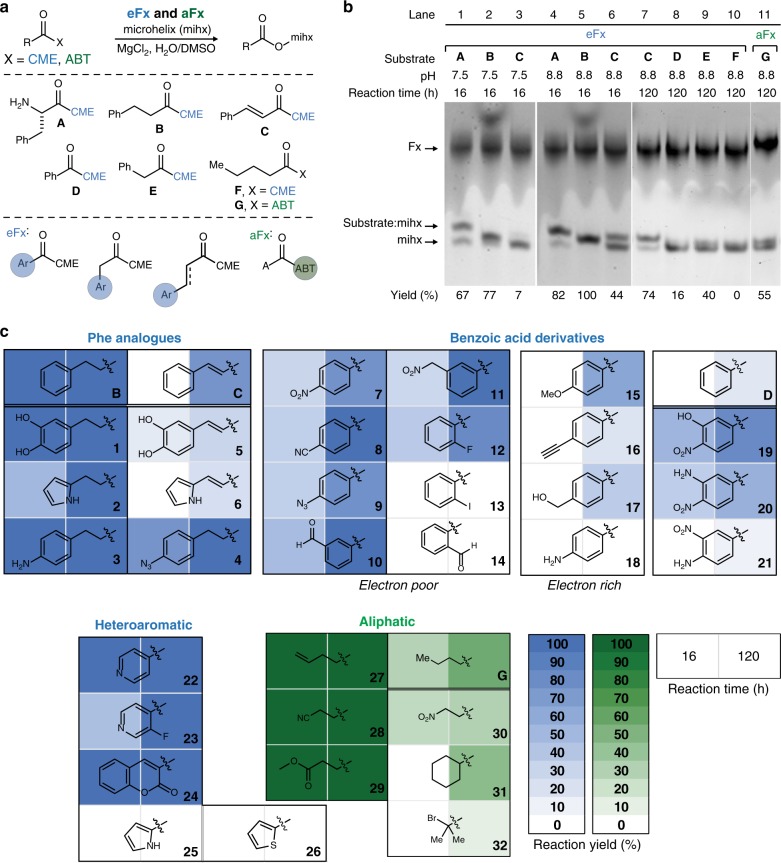
Expanding the Fx substrate scope to analogs with various scaffolds. **a** Systematic design of noncanonical substrates for ribosome mediated polymerization. Phe (**A**) and structurally diversified noncanonical substrates (**B–G**). **b** Fx-catalyzed acylation under optimized conditions. Acid (pH 5.2) denaturing PAGE analysis under various conditions for Fx-catalyzed acylations of a microhelix tRNA (22 nt) with **A–G**. The acylation reactions were performed using eFx (45 nt) or aFx (47 nt) and monitored over 120 h at two different pHs (7.5 vs. 8.8). Reaction condition: 50 mM HEPES (pH 7.5) or bicine (pH 8.8), 60 mM MgCl_2_, 1 µM microhelix, 5 µM Fx, and 5 mM substrates in 20 % (v/v) DMSO solution. **c** The range of noncanonical substrates compatible with Fx was further extended on four different monomer structure (Phe analogs, benzoic acid derivatives, heteroaromatic, and aliphatic substrates). All acylation heat maps are shaded by percent conversion of microhelix. The blue and green color codes are used for the reaction with the CME leaving group:eFx pair and the ABT leaving group:aFx pair, respectively. See in Supplementary Fig. 3 for the numerical values of acylation

To identify design rules for expanding the substrate scope for Fx-catalyzed tRNA mis-acylation, we initially determined compatible substrate scaffolds. For this, we benchmarked the molecular structure of CME-activated phenylalanine (Phe-CME, **A**, Fig. 2a, middle panel) as the optimal substrate for eFx^51,58,64,66^ and investigated eFx’s substrate flexibility toward a series of five substrates with systematic increases in the degree of modification from the parent structure, **A** (**B–F**, Fig. 2a, middle panel). These include: **B** (hydrocinnamic acid): amine excluded from **A**; **C** (cinnamic acid): the unsaturated form of **B**; **D** and **E** (benzoic and phenylacetic acid, respectively): two or one carbon excluded from **B**; and **F** (propanoic acid): aryl replaced with aliphatic group in **B**.

First, we determined the acylation efficiency of **A** to a small tRNA mimic, microhelix tRNA (mihx, 22 nt) by eFx using the previously reported standard acylation conditions (pH 7.5, 0 °C)^67^ (Fig. 2a, top panel). Analysis of the reaction mixture by denaturing acidic polyacrylamide gel electrophoresis (PAGE) indicated that 67% of mihx was acylated with **A** (Fig. 2b, lane 1). With this benchmark established, we then screened substrate-eFx compatibility of the five substrates. eFx successfully acylated mihx with **B** in 77% yield, indicating that an amine functional group is not required for aminoacylation (Fig. 2b, lane 2). Moving further from the Phe structure proved difficult, as α,β-unsaturated substrate **C** was incompatible for mihx acylation via flexizyme under standard reaction conditions (Fig. 2b, lane 3). However, as we increased reaction pH and time (pH 7.5 to 8.8 and 16 h to 120 h, see Supplementary Figs. 1, 2 for full details), mihx acylation with **C** improved yielding 44% and 74% after 16 and 120 h, respectively (Fig. 2b, lanes 6, 7). Notably, reactions with a pH of 8.8 increased the yields for **A** and **B** to 82% and 100%, respectively (Fig. 2b, lanes 4, 5). Using these same conditions, substrates **D** and **E** were also acylated to the mihx in 16% and 40% yield, respectively (Fig. 2b, lane 8, 9). As expected, the aliphatic substrate **F** was not charged to the mihx by eFx, as the substrate does not contain an aryl group for substrate recognition by eFx (Fig. 2b, lane 10). However, changing the substrate’s leaving group from CME to ABT and employing aFx in place of eFx enabled charging of the same aliphatic substrate **G** in 55% yield after 120 h (Fig. 2b, lane 11). Hence, using the optimized acylation conditions and the appropriate leaving group and Fx, all five substrates are successfully charged to the tRNA mimic.

Next, we sought to further expand the substrate scope by elaborating the scaffolds of **B**, **C**, **D**, and **G** in which we adopted the ‘Design-Build-Test-Analyze’ approach of synthetic biology, and stepped through monomer space in chemical increments to inform design rules for flexizyme-mediated acylation. The key idea was to teach us about permissible substrates, not only substrates that could be used by the Fx system, but also, later, the ribosome. For this, we determined the mihx-acylation efficiency of eFx and aFx with four sets of scaffold analogs: Phe analogs harboring saturated and unsaturated aliphatic scaffolds with an aryl group, benzoic acid derivatives with a variety of functional groups, heteroaromatic scaffolds with different electronic properties, as well as aliphatic scaffolds with various steric hindrances (Fig. 2c). Importantly, the idea was not to simply optimize flexizyme charging conditions (e.g., by changing reaction time or pH), which is well-known in the field. Rather, we set out to gain insights about the importance of substrate structure for efficient flexizyme charging, and in follow-up experiments, independently verify these insights.

To investigate saturated and unsaturated aliphatic scaffolds containing an aryl group, we explored Phe analogs derived to bear a variety of functionalities (**1**–**6**) from the Fx substrates **B** and **C**. Under optimal conditions, the substrates **1**–**4** were charged to the mihx by eFx in yields of 50–100% after 16 h and 100% after 120 h (Supplementary Figs. 3, 4). Substrate **5** and **6** containing α,β-unsaturated scaffolds showed similar yield to their parent structure **C**. Both were charged by eFx at lower efficiencies (30% and 22% yield, respectively) than the saturated substrates, likely owing to their increased structural rigidity, hindering interaction with the Fx binding pocket.

To further understand the substrate compatibility of eFx toward benzoic acid (**D**), a monomer that has been incorporated by the ribosome before^27^, we prepared a series of derivatives with altering electronic character (electron-poor: **7**–**14**, electron-rich: **15**–**18**) as well as substituent position (ortho, meta, para), performed Fx-catalyzed acylation reactions, and determined the acylation efficiency by acid denaturing PAGE and densiometric analysis (Supplementary Figs. 3, 5, 6). For *p*-nitro-substituted substrate (**7**), acylation yields of eFx were 30% after 16 h and 76% after 120 h, and for the unsubstituted substrate (**D**), 0% at 16 h, 16% at 120 h. Similarly, high yields (28–48% at 16 h, 78–100% at 120 h) were observed for the electron-poor substrates (**8**–**11**) bearing a *p*-nitrile, *p*-azide, *m*-formyl group, and *m*-nitromethyl group, respectively. In contrast, substrates with moderate electron-donating groups such as *p*-methoxy (**15**), *p*-ethynyl (**16**), and *p*-hydroxymethyl (**17**) showed lower reaction rates; no acylation was observed after 16 h and only with moderate yields after 120 h (19–63%). We observed no conversion after 120 h for electron-rich *p*-amino substrate **18**. These results indicate a significant influence of electronic effects; reaction rates generally increased for electron-poor substrates and decreased for electron-rich substrates.

We tested this hypothesis by installing an electron-withdrawing nitro-group at the meta position of the Fx incompatible substrate, **18**, a modification that gives substrate **21**. As predicted, the modified substrate became viable, giving a yield of 10% after 120 h. Switching the nitro and amino groups (substrate **20**) further improved the efficiency to 55% yield after 120 h, supporting the reactivity trend based on electronic character. In addition, we observed that ortho-substituent tolerance was governed by steric effects as *o*-fluoro **12** resulted in 82% yield after 120 h, whereas substrates with larger ortho substituents (iodo **13**, formyl **14**) were not charged to the mihx. The correlation between electronic character and Fx-catalyzed acylation was further confirmed by investigating the electron-poor heteroaromatic substrates pyridine **22**, fluoro-pyridine **23**, and coumarin **24**. All three substrates were charged with high yields (45–100% at 16 h and 100% at 120 h), consistent with the putative electronic trends. In contrast, five-membered electron-rich heteroaromatic substrates (pyrrole **25**, **25a** and thiophene **26**, **26a**; see Supplementary Fig. 7 for **25a** and **26a**) did not show any reactivity in the Fx-catalyzed tRNA acylation reaction.

Finally, we investigated the substrate compatibility of aFx by exploring its catalytic activity toward aliphatic variants of substrate **G**. We found that straight chain aliphatic acids are highly favored substrates; alkenyl (**27**), cyano (**28**), and ester (**29**) analogs were charged with 100% yield after 16 h. Nitroalkane (**30**) was a competent substrate, albeit in diminished yield (25%, 16 h and 30%, 120 h). In contrast, sterically hindered cyclohexyl (**31**) were charged at a slower rate (30% yield, 120 h). Moreover, α-bromoisobutyrate (**32**) was charged to only 10% after 120 h, suggesting that increased steric bulk decreases Fx-catalyzed acylation.

**Fig. 3:**
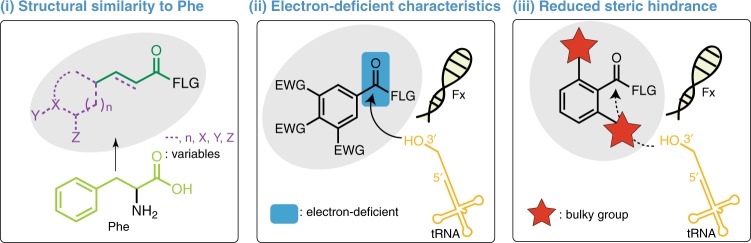
General substrate design rules for flexizyme tRNA-charging. Substrates with structural similarity to Phe, electron-deficient characteristics, and reduced steric hindrance around the carbonyl group show high compatibility with the flexizyme system

In summary, from the 37 tested analogs, 32 hitherto unknown Fx substrates were identified, significantly expanding the scope of the Fx-catalyzed aminoacylation reaction. Based on their molecular characteristics and efficiencies in Fx-catalyzed acylation, general design rules for successful Fx substrates were deduced: (i) higher structural similarity to Phe for eFx (a feature that has been previously established, and derives from the fact that Fx was initially evolved to work with a Phe derivative), (ii) increasing the electrophilicity of the carbonyl region, and (iii) reduced steric hindrance at the acylation site (Fig. 3). Of note, for some substrates, the obtained reaction yields may not precisely follow the theoretical trends of the electronic properties of functional groups. This discrepancy may be owing to the different solubility of the substrate in water as in the case of substrates **15**, **17**, and **D**.

**Fig. 4:**
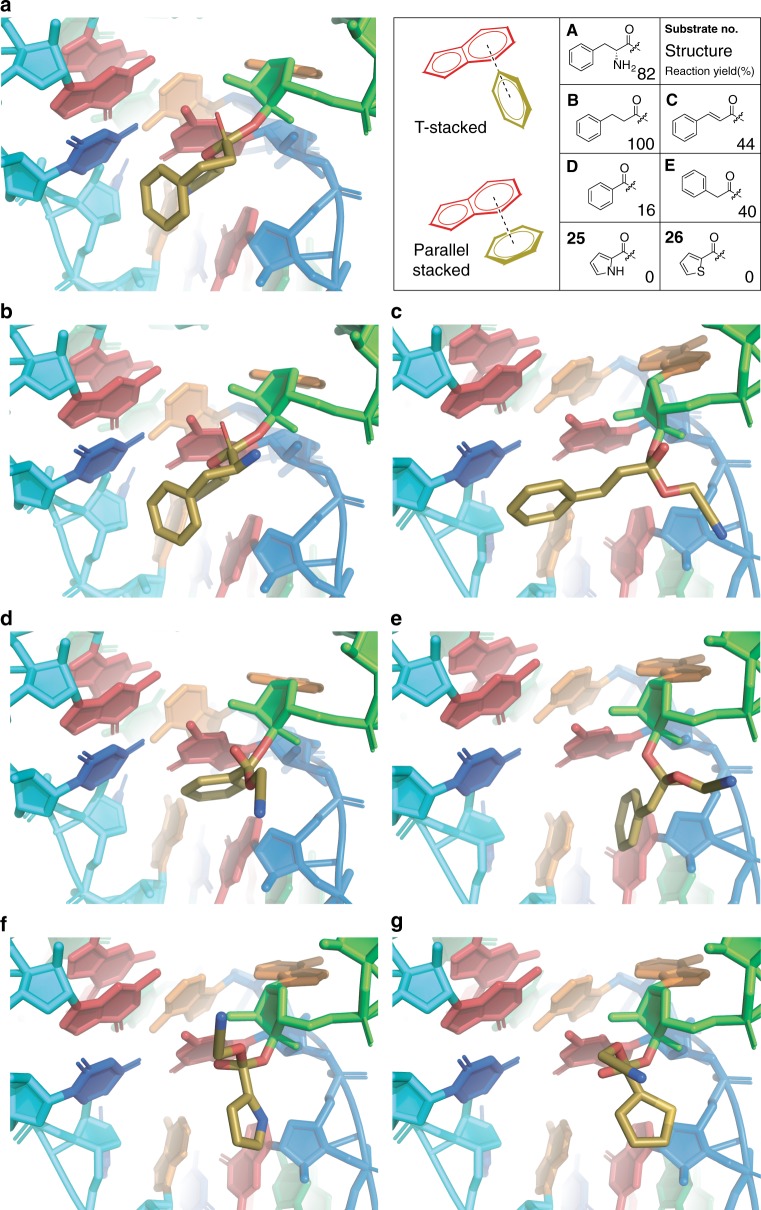
Simulated molecular interactions between selected substrates and the binding pocket of eFx. Tetrahedral intermediate models of the CME esters were optimized and subjected to Monte Carlo energy optimization via Rosetta. Dark yellow represents **a** Phe (**A**), **b** hydrocinnamic acid (**B**), **c** cinnamic acid (**C**), **d** benzoic acid (**D**), **e** phenylacetic acid (**E**). No strong interaction with the guanine residue (top red) is observed for **f** pyrrole-2-carboxylic acid (**25**) and **g** 2-thiophenecarboxylic acid (**26**); (green: substrate-charged tRNA)

We next used computational modeling to gain further insights about possible constraints for using flexizyme to charge noncanonical chemical substrates onto tRNAs. A previous crystallographic study^58^ suggests that when an aromatic amino acid such as Phe is charged by Fx, the phenyl ring of the substrate stacks against the terminal J1a/3 base pair. Notably, the structure as crystallized (PDB: 3CUL and 3CUN) contains only residual density for a phenylalanyl-ethyl ester ligand, which is suggestive of a possible location of the active site and the substrate’s conformation. Using Rosetta^68^, we generated models (Supplementary Fig. 8) of the tetrahedral intermediates formed with tRNA and five representative substrates (**A**–**E**) as well as five-membered heterocyclic substrates (**25**, **25a**, **26**, and **26a**) that give no acylation yield on Fx-catalysis (Fig. 2c). The modeling (Fig. 4) supports either T-stacked interaction for Phe and hydrocinnamic acid (**B**) or parallel-stacked interactions for cinnamic acid (**C**), benzoic acid (**D**), and phenylacetic acid (**E**). In contrast, pyrrole and thiophene groups are unable to form particularly favorable interactions with the terminal J1a/3 base pair. The absence of these interactions indirectly reflects the three design rules and may explain our empirical observation that **25, 25a** and **26, 26a** containing an electron-rich heteroaromatic group are poor substrates for eFx.

### Ribosome-mediated synthesis with Fx-acylated tRNA substrates

**Fig. 5:**
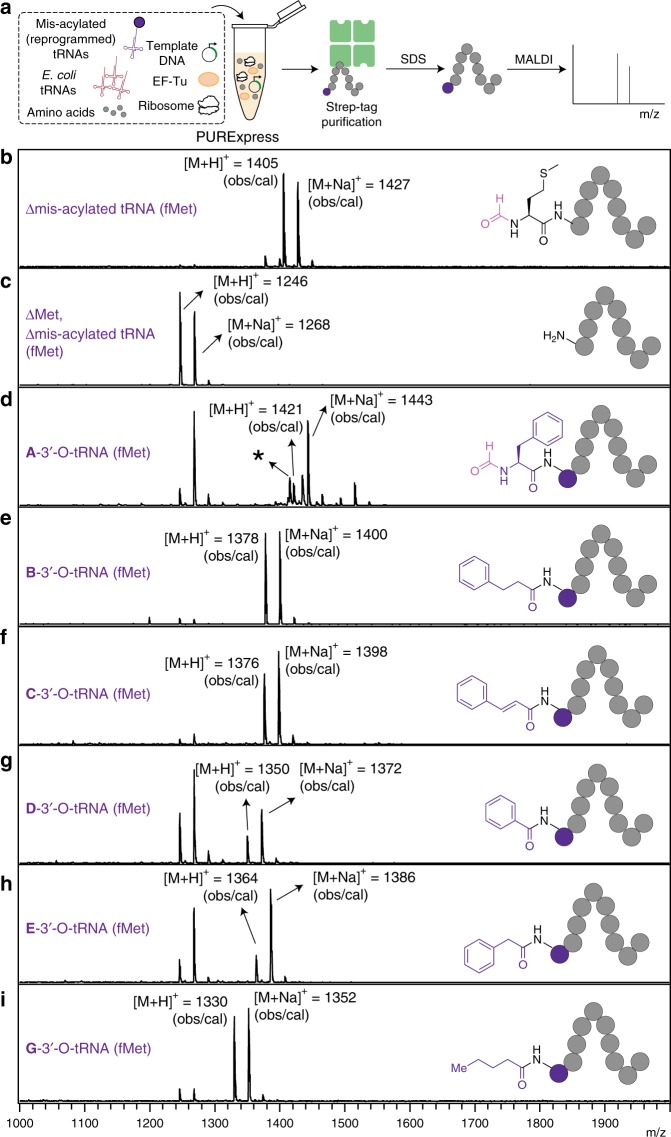
Ribosomal synthesis of N-terminal functionalized peptides with noncanonical substrates. **a** Schematic overview of peptide synthesis and characterization. N-terminal functionalized peptides were prepared in the PURExpress system by using Fx-charged tRNA^fMet^, purified via the Strep tag, denatured with SDS, and characterized by MALDI mass spectrometry. **b** Mass spectrum of the peptide in the presence of all 20 natural amino acids and absence of Fx-charged tRNA. **c** Mass spectrum of the peptide in the absence of methionine and Fx-charged tRNA. **d**–**i** Mass spectra of peptides with N-terminally incorporated noncanonical substrates. *A minor amount of peptide containing phenylalanine at the N-terminus was unformylated. NH_2_-**F**WSHPQFEKST-OH; [M + Na]^+^ = 1415, **A**: phenylalanine, **B**: hydrocinnamic acid, **C**: cinnamic acid, **D**: benzoic acid, **E**: phenylacetic acid, **G**: propanoic acid

Next, we investigated whether the noncanonical Fx substrates are accepted by the natural protein translation machinery. The goal was to demonstrate that the ribosome was compatible with these substrates, rather than focus on a specific biopolymer and its application. Based on our optimized conditions, we performed Fx-catalyzed acylation reactions using Fx-optimized tRNAs^67^ instead of the mihx. Then, we purified the tRNA-monomers and added them to a cell-free protein synthesis reaction, allowed translation to proceed, and determined the incorporation of the noncanonical substrates into a small reporter peptide by MALDI-TOF mass spectrometry (Fig. 5, Supplementary Figs. 10, 15).

Initially, we attempted to use a well-established crude extract-based *E. coli* cell-free protein synthesis^34,69–73^, which is capable of high-level incorporation of noncanonical amino acids. However, we were not able to characterize the reporter peptide, presumably because active peptidases in the extract digested the peptide. In order to circumvent possible undesired degradation, we turned to the commercially available PURExpress (Protein synthesis Using Recombinant Elements) system^74^. The PURExpress system contains the minimal set of components required for protein translation, thereby minimizing any undesired peptide degradation, and allows addition of custom sets of amino acids and tRNAs of interest. Previous works from the Suga lab, among others, have shown that this platform is suitable for assessing peptide synthesis^75^, especially for N-terminal incorporation of noncanonical monomers^25,65^. As a reporter peptide, we designed a T7 promoter-controlled DNA template (pJL1_StrepII) encoding the translation initiation codon AUG for N-terminal incorporation of the noncanonical Fx substrates, a Streptavidin (Strep) tag and a Ser and Thr codon (**X**WHSPQFEKST (strep tag), where **X** indicates the position of the noncanonical Fx substrate, for details, see SI). Peptide synthesis was performed using only the nine amino acids that decode the initiation codon AUG and the purification tag (Supplementary Fig. 9). We excluded the other 11 amino acids to prevent corresponding endogenous tRNAs from being aminoacylated and used in translation, thereby, eliminating competition between endogenous tRNAs and Fx-charged tRNAs during peptide synthesis. For this, PURExpress reactions were incubated at 37 °C for 4 h. The synthesized peptides were then purified using Strep-Tactin-coated magnetic beads (IBA), denatured with SDS, and characterized by matrix-assisted laser desorption/ionization–time-of-flight (MALDI-TOF) mass spectrometry (Fig. 5a).

As a positive control experiment, we prepared a peptide in the presence of all 20 natural amino acids and absence of any Fx-charged tRNA, so that the reporter mRNA is translated into MWHSPQFEKST according to the standard genetic code. Indeed, we detected two major peaks corresponding to the theoretical mass of the peptide ions. The Met residue at the N-terminus was found to be formylated (fM) (fMWHSPQFEKST) by a formylase present in the PURExpress system^76^; [M + H]^+^ = 1405 (observed, obs), 1405 Da (calculated, cal), [M + Na]^+^ = 1427 (obs), 1427 Da (cal) (Fig. 5b). As a negative control experiment, we performed a PURExpress reaction in the presence of only nine amino acids encoding the residues downstream of the initiating codon (W, S, H, P, Q, F, E, K, and T); no Met or mis-acylated tRNA^fMet^ was added to the reaction mixture.

The MALDI spectrum shows only a single species for the synthesized peptide giving a mass of 1246 ([M + H]^+^) and 1268 Da ([M + Na]^+^) (Fig. 5c). The observed peaks correspond to the theoretical mass of a peptide with sequence WHSPQFEKST, indicating that translation initiation occurs on the succeeding mRNA codon if the amino acid for the initiating codon is not present, a phenomenon previously reported^76^.

For incorporation of the noncanonical substrates (**B–E** and **G**) at the start codon, we used the tRNA^fMet^ containing the CAU anticodon, corresponding to the AUG codon on the mRNA and charged all five substrates onto the tRNA separately. The same amount of precipitated tRNA containing a mixture of substrate-charged/uncharged tRNA was added to the PURExpress reaction. Methionine was not added to the reaction so as to avoid the incorporation of Met at the start codon by Met-charged endogenous tRNA^fMet^ produced in the PURExpress system. We discovered that all the peaks found in the MALDI spectra corresponded to a theoretical mass of peptide that contains the substrate on the N-terminus (Fig. 5d–i). It is notable that N-terminal Trp was found to be unformylated (Fig. 5c) in comparison with that the N-terminal Met in Fig. 5b, which was partially formylated. The N-terminus Phe (Fig. 5d) was found with (**fF**) and without (**F**) formylation, suggesting that a larger side chain may prohibit the formylase from efficiently formylating the residue.

We carried out the same acylation reaction onto a tRNA^fMet^ for the other noncanonical substrates (**B**–**G** and **1**–**32**, except for the six substrates that showed no acylation; **F**, **13**, **14**, **18**, **25**, and **26**) and subsequently synthesized 32 noncanonical peptide hybrid molecules, each with its substrate on the N-terminus, indicating all the noncanonical substrates were incorporated into a peptide, respectively. The MALDI spectra of the purified peptides are shown in Supplementary Figs. 10–15 (Supplementary Fig. 10: Phe analogs, Supplementary Fig. 11: benzoic acid derivatives with an electron-withdrawing group (EWG), Supplementary Fig. 12: benzoic acid derivatives with an electron-donating group (EDG), Supplementary Fig. 13: benzoic acid derivatives with an EWG and EDG, Supplementary Fig. 14: heteroaromatic substrates, Supplementary Fig. 15: aliphatic substrates). The substrates with higher acylation yields tend to show higher translation efficiency (Supplementary Fig. 16), suggesting that the concentration of mis-acylated tRNA is a limiting factor for the translation. To more rigorously characterize the N-terminal peptides, we additionally quantified peptide yields (Supplementary Fig. 17). These data support our hypothesis that the system is limited by mis-acylated tRNA.

### Design rules as a prediction tool for tRNA charging

**Fig. 6:**
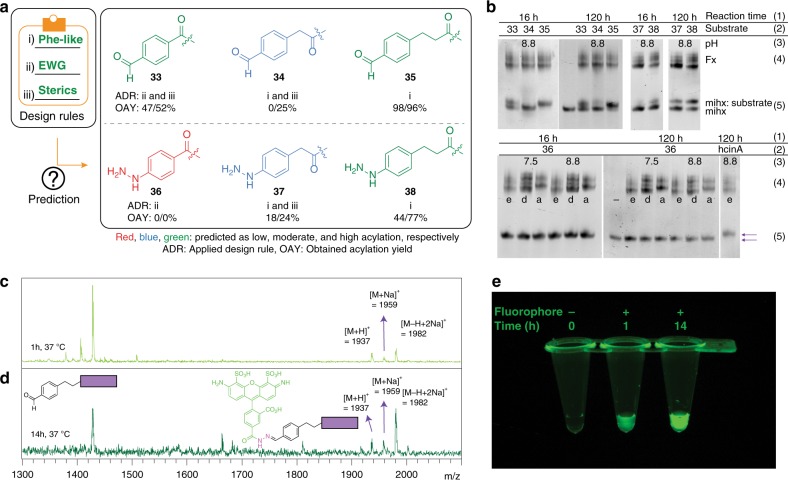
Putting flexizyme design rules into action for aldehyde and hydrazine bioconjugation. **a** We designed and synthesized 6 additional substrates (**33**–**36**). Before Fx-mediated acylation, we estimated acylation of the substrates would give a low (<20 %, red), moderate (20–50%, blue), and high (>50%, green) yield. **b 33**–**35** and **37–38** were charged to mihx with the predicted acylation yields (upper panel). In contrast, **36** containing an electron-donating group was not charged to mihx at any pHs, reaction times, and flexizymes, whereas hydrocinnamic acid (**B**) was charged in 100% yield (lower panel), suggesting our design rules are in a good agreement with our prediction and useful as an efficient tool for predicting a substrate tRNA-charging yield. The obtained acylation yields (OAY, Fig. 6a) were determined by quantifying the relative band intensity on the gel using ImageJ software. **c**, **d** Mass spectra of the **35** peptides incubated with a hydrazide dye (Alex Fluor 488) at 37 °C for 1 and 14 h, respectively. The peaks correspond to the peptide with the dye chemically attached to **35**. **e** The purified products obtained from the PURExpress reaction at the time point of 1 h and 14 h showed fluorescence after exposure of UV light filtered by 560/50 nm

We next set out to exploit the design rules for flexizyme-mediated acylation reactions we identified earlier (Fig. 3). To do so, we selected an additional set of six substrates with parent structures from groups **B**, **D**, and **E** that contain a hydrazine or an aldehyde functional group (**33**–**38**, Fig. 6a). Hydrazine and aldehyde chemistry were selected because they are useful for bioconjugation reactions on the N-terminus of a peptide. Although the reaction between an aldehyde and a hydrazine is chemoselective and is as highly reactive (*k* = ~10^1^–10^3^ M^−1^ s^−1^)^63^ as the widely used Cu (I)-catalyzed azide and alkyne cycloaddition reaction (CuAAC, *k* = ~10^1^–10^2^ M^−1^ s^−1^)^77^, hydrazone conjugation is limited because the aldehyde and hydrazine groups have been generally introduced into peptides and proteins indirectly using post-translational modification reactions, requiring additional chemical or enzymatical reactions^78,79^. Direct incorporation of these functional groups through the Fx-mediated acylation may more effectively attach synthetic molecules site-specifically to peptides or proteins.

Following monomer design, we predicted that flexizyme-catalyzed acylation would be successful based on our design rules, classifying the substrates with low (<20%, red), moderate (20–50%, blue), and high (>50%, green in Fig. 6a) acylation yield. We hypothesized that **33**–**35** and **37**–**38** would give moderate-to-high acylation yield because of the structural similarity to Phe (design rule i) and electronic/steric effect (design rules ii/iii), respectively. We anticipated a lower efficiency of **34** and **37** because of the electronic effect from the carbonyl of the substrates. We also predicted that **36** would not be a good flexizyme substrate owing to the strong electron-donating characteristics of the hydrazine (design rule ii). As predicted, our acylation results showed no acylated mihx was produced with **36** under 12 different reaction conditions. Also, as expected, **34** and **37** gave moderate (~25%) acylation yields, whereas **35** and **38** gave high (>70%) acylation yields (Fig. 6b). Substrate **33** resulted in yields of ~50%. The five substrates acylated to mihx (**33**–**35** and **37–38**) were subsequently charged to tRNA^fMet^ and incorporated into the N-terminus of a peptide using the PURExpress system (Supplementary Fig. 18). These data demonstrate the use of our rules to guide noncanonical monomers for flexizyme-catalyzed acylation.

### Application of non-natural monomers in peptide hybrids

We next applied our aldehyde substrates in bioconjugation reactions. To do so, we leverage the thermodynamically favorable imine-forming reaction in water between an aldehyde and hydrazine. Such chemistry has been used before to link biomolecules to various probes because the reaction is mild, facile, and a has high tolerance reaction toward a wide range of molecular weights over a wide pH range^61,63,80^. However, no direct incorporation of an aldehyde group on a peptide N-terminal for bioconjugation via a ribosome catalyzed coupling has, to our knowledge, been reported. For a demonstration purpose, the aldehyde functional group of the peptide containing **35** at the N-terminus, was condensed with a fluorescent molecule (Alex Fluor 488 hydrazide). After incubation of the crude PURExpress product (1 and 14 h) with the fluorophore at 37 °C, we purified the peptides and analyzed them by MALDI-TOF spectrometry. We observed a new peak corresponding to the mass of peptide bearing the dye at the N terminus (Fig. 6c, d) and the resulting peptide showed fluorescent characteristics (Fig. 6e). Although the ribosome has shown a high tolerance for noncanonical substrates at the N-terminus, introduction of the substrates with a large molecular size or conformational rigidity has been limited^25,81^. Our results therefore suggest that substrates incompatible with the direct incorporation by the wild-type ribosome can be chemically attached using rationally designed synthetic handles.

## Discussion

In this work, we set out to systematically expand the range of chemical substrates for translation through the identification of design rules for flexizyme-mediated charging of noncanonical monomers to tRNAs. We showed that a diverse repertoire of substrates systematically built from four scaffolds (phenylalanine, benzoic acid, heteroaromatic, and aliphatic) are acylated to tRNAs. We then showed that these acylated tRNA-monomers could be used in ribosome mediated polymerization to make 10 s of unique peptide hybrid products. Our results have several key features.

First, our rational synthetic biology design cycle approach to scaffold design allowed us to identify design rules for using flexizymes to charge noncanonical monomers onto tRNA. We found, as expected, that phenylalanine-resembling substrates are favorable for Fx-catalyzed acylation reactions. We also found important guiding principles. For example, electron-deficient substrates are favored over electron-rich, and certain bulky groups are poorly tolerated near the acylation site. In addition, by investigating the molecular interaction of key substrates in the binding pocket of flexizyme using computational modeling, we found that either T-stacked or parallel-stacked interactions are key features that enable charging by Fx. The substrate design rules we discovered here should reduce the need for ad hoc/trial-and-error exercises when developing new substrates for ribosome catalyzed transformations.

Second, we showed that tRNA-monomers from our expanded substrates successfully yield a wide variety of N-functionalized peptides in a PURExpress system through genetic code reprogramming. This is important because our data join an emerging number of studies showing that the ribosome is capable of polymerizing a wide array of substrates, especially at the N-terminus. Although the production of noncanonical N-terminal peptides themselves was not our focus, they might be used directly by others in the field in multiple ways.

Third, we showed utility for using peptide hybrids in bioconjugation through the imine-forming reaction between an aldehyde and hydrazine. This has the potential to combine the advantages of synthetic polymers and sequence-defined peptides by chemically attaching a molecule with a polymerizable unit, which could lead to innovative hybrid materials. As was recently suggested by Ad et al.^82^, other moieties such as the benzoic acids we incorporated in the development of our design rules could be used to make aramid type structures. Notably, the monomers in that study all follow the design rules we discovered here.

Looking forward, we anticipate that our work will enable the design and selection of new classes of noncanonical monomers for use in translation. For example, some of the monomers we describe begin the march towards ribosome-mediated synthesis of different classes of sequence-defined polymers that are not polyesters or polyamides, perhaps even those with carbon–carbon bonds. However, since the shape, physiochemical, and dynamic properties of the ribosome and its active site have been evolutionarily optimized to operate with proteins built of ~20 canonical amino acids, such advances will need to be supported by additional efforts in engineering the translation apparatus^83–90^.

## Methods

### General procedure for formation of CME

To a glass vial with a stir bar was added carboxylic acid (1 equiv.), CH_2_Cl_2_ (1.0 M), triethylamine (1.5 equiv.), and chloroacetonitrile (1.2 equiv.). After stirring for 16 h at 25 °C, the reaction mixture was diluted with EtOAc and washed with water or brine. The organic phase was dried and concentrated to provide the crude product. The product was purified by flash column chromatography if necessary.

### General procedure for formation of ABT ester

According to standard procedures^60^, to a glass vial equipped with a stir bar was added tert-butyl (2-(4-(mercaptomethyl)benzamido)ethyl) carbamate (ABT) (1 equiv.), carboxylic acid (1.4 equiv.), CH_2_Cl_2_ (0.3 M), DMAP (2.8 equiv.), and EDC•HCl (2.8 equiv.). After stirring for 3 h at 25 °C, the reaction was evaporated under reduced pressure, diluted with EtOAc, and washed with 1 M HCl and saturated NaHCO_3_. The organic phase was dried and concentrated to provide the crude Boc-protected product. The Boc-protected product was purified by flash column chromatography. The purified product was dissolved in 4 M HCl•dioxane and stirred for 1 h. Concentration under reduced pressure provided the product in sufficient purity.

### Acylation of microhelix

The experiment using microhelix was performed using two flexizymes (eFx and aFx). The coupling reaction of activated ester with microhelix was carried out as follows: 1 μL of 0.5 M HEPES (pH 7.5) or bicine (pH 8.8), 1 μL of 10 μM microhelix, and 3 μL of nuclease-free water were mixed in a PCR tube with 1 μL of 10 μM eFx, dFx, and aFx, respectively. The mixture was heated for 2 min at 95°C and cooled down to room temperature over 5 min. 2 μL of 300 mM MgCl_2_ was added to the cooled mixture and incubated for 5 min at room temperature. Followed by the incubation of the reaction mixture on ice for 2 min, 2 μL of 25 mM activated ester substrate in dimethyl sulfoxide (DMSO) was then added to the reaction mixture. The reaction mixture was further incubated for 6–120 h on ice in cold room.

### Acidic PAGE analysis

 1 μL of crude reaction mixture was aliquoted at a desired time point and the reaction was quenched by the aliquot with 4 μL of acidic loading buffer (150 mM NaOAc, pH 5.2, 10 mM EDTA, 0.02% BPB, 93% formamide). The crude mixture was loaded on 20% polyacrylamide gel containing 50 mM NaOAc (pH 5.2) without further RNA precipitation process. The electrophoresis was carried out in cold room using 50 mM NaOAc (pH 5.2) as a running buffer. The gel was stained with GelRed (Biotium) and visualized on a Bio-Rad Gel Doc XR+. The acylation yield was determined by quantifying the intensity of the microhelix bands using ImageJ (NIH).

### Acylation of tRNA

The acylation reaction of tRNA was carried out as follows: 2 μL of 0.5 M HEPES (pH 7.5), 2 μL of 250 μM tRNA, 2 μL of 250 μM of aFx selected on the microhelix experiment and 6 μL of nuclease-free water were mixed in a PCR tube. The mixture was heated for 2 min at 95°C and cooled down to room temperature over 5 min. In all, 4 μL of 300 mM MgCl_2_ was added to the cooled mixture and incubated for 5 min at room temperature. Followed by the incubation of the reaction mixture on ice for 2 min, 4 μL of 25 mM activated ester substrate in DMSO was then added to the reaction mixture. The reaction mixture was further incubated for the optimal time determined on the microhelix experiment on ice in cold room.

### In vitro translation

The produced using the reprogrammed genetic code approach was produced by the PURExpress (Δ aa, Δ tRNA, E6840) system. Six micrograms of the mis-acylated tRNA dissolved in 1 μL of 1 mM NaOAc (pH 5.2) was added into a 9 μL solution mixture containing a 2 μL of Solution A, 1 μL of tRNA, 3 μL of Solution B, 1 μL of DNA template (130 ng μL^−1^), 1 μL of nuclease-free water, and 1 μL of 5 mM amino acid mixtures in 20 mM Tris buffer (pH 7.5). The reaction mixture was incubated in 37°C for 4 h.

### Aldehyde and hydrazine chemistry

Into the 20 µL of crude PURExpress product, 5 µL of 50 mM Alexa Fluor 488 hydrazide (Thermo Fisher) was added. The reaction mixture was incubated at 37°C for 14 h. 10 µL of mixture was obtained at the time point of 1 and 14 h. The reaction mixture was purified and characterized by the same methods described in the previous and next section.

## Supplementary information

Supplementary Information

## Data Availability

All data generated or analyzed during this study are included in this article (and its supplementary information) or are available from the corresponding authors on reasonable request. Direct correspondence to Michael C. Jewett.

## Code availability

Scripts for conducting modeling simulations are available at https://github.com/everyday847/flexizyme.

## References

[1] Edelmann P, Gallant J (1977). Mistranslation in E. coli. Cell.

[2] Precup J, Ulrich AK, Roopnarine O, Parker J (1989). Context specific misreading of phenylalanine codons. Mol. Gen. Genet..

[3] Rodnina MV, Wintermeyer W (2001). Fidelity of aminoacyl-tRNA selection on the ribosome: kinetic and structural mechanisms. Annu Rev. Biochem..

[4] Cropp TA, Anderson JC, Chin JW (2007). Reprogramming the amino-acid substrate specificity of orthogonal aminoacyl-tRNA synthetases to expand the genetic code of eukaryotic cells. Nat. Protoc..

[5] Morimoto J, Hayashi Y, Iwasaki K, Suga H (2011). Flexizymes: their evolutionary history and the origin of catalytic function. Acc. Chem. Res..

[6] Albayrak C, Swartz JR (2013). Cell-free co-production of an orthogonal transfer RNA activates efficient site-specific non-natural amino acid incorporation. Nucleic Acids Res..

[7] Chin JW (2017). Expanding and reprogramming the genetic code. Nature.

[8] Mukai T, Lajoie MJ, Englert M, Soll D (2017). Rewriting the genetic code. Annu Rev. Microbiol..

[9] Voller JS, Budisa N (2017). Coupling genetic code expansion and metabolic engineering for synthetic cells. Curr. Opin. Biotech..

[10] Vargas-Rodriguez O, Sevostyanova A, Soll D, Crnkovic A (2018). Upgrading aminoacyl-tRNA synthetases for genetic code expansion. Curr. Opin. Chem. Biol..

[11] Arranz-Gibertt P, Vanderschurent K, Isaacs FJ (2018). Next-generation genetic code expansion. Curr. Opin. Chem. Biol..

[12] Tajima K, Katoh T, Suga H (2018). Genetic code expansion via integration of redundant amino acid assignment by finely tuning tRNA pools. Curr. Opin. Chem. Biol..

[13] Rogers JM, Suga H (2015). Discovering functional, non-proteinogenic amino acid containing, peptides using genetic code reprogramming. Org. Biomol. Chem..

[14] Obexer R, Walport LJ, Suga H (2017). Exploring sequence space: harnessing chemical and biological diversity towards new peptide leads. Curr. Opin. Chem. Biol..

[15] Fujino T, Goto Y, Suga H, Murakami H (2016). Ribosomal synthesis of peptides with multiple beta-amino acids. J. Am. Chem. Soc..

[16] Ohshiro Y (2011). Ribosomal synthesis of backbone-macrocyclic peptides containing gamma-amino acids. Chembiochem.

[17] Goto Y, Murakami H, Suga H (2008). Initiating translation with D-amino acids. RNA.

[18] Katoh T, Tajima K, Suga H (2017). Consecutive elongation of D-amino acids in translation. Cell Chem. Biol..

[19] Kawakami T, Ishizawa T, Murakami H (2013). Extensive reprogramming of the genetic code for genetically encoded synthesis of highly N-alkylated polycyclic peptidomimetics. J. Am. Chem. Soc..

[20] Iwane Y (2016). Expanding the amino acid repertoire of ribosomal polypeptide synthesis via the artificial division of codon boxes. Nat. Chem..

[21] Terasaka N, Iwane Y, Geiermann AS, Goto Y, Suga H (2015). Recent developments of engineered translational machineries for the incorporation of non-canonical amino acids into polypeptides. Int. J. Mol. Sci..

[22] Ohta A, Murakami H, Higashimura E, Suga H (2007). Synthesis of polyester by means of genetic code reprogramming. Chem. Biol..

[23] Ohta A, Murakami H, Suga H (2008). Polymerization of alpha-hydroxy acids by ribosomes. Chembiochem.

[24] Goto Y, Suga H (2009). Translation initiation with initiator tRNA charged with exotic peptides. J. Am. Chem. Soc..

[25] Rogers JM (2018). Ribosomal synthesis and folding of peptide-helical aromatic foldamer hybrids. Nat. Chem..

[26] Torikai K, Suga H (2014). Ribosomal synthesis of an amphotericin-B inspired macrocycle. J. Am. Chem. Soc..

[27] Kawakami T, Ogawa K, Hatta T, Goshima N, Natsume T (2016). Directed evolution of a cyclized peptoid-peptide chimera against a cell-free expressed protein and proteomic profiling of the interacting proteins to create a protein-protein interaction inhibitor. ACS Chem. Biol..

[28] Kanter G (2007). Cell-free production of scFv fusion proteins: an efficient approach for personalized lymphoma vaccines. Blood.

[29] Cho H (2011). Optimized clinical performance of growth hormone with an expanded genetic code. Proc. Natl Acad. Sci. USA.

[30] Axup JY (2012). Synthesis of site-specific antibody-drug conjugates using unnatural amino acids. Proc. Natl Acad. Sci. USA.

[31] Zimmerman ES (2014). Production of site-specific antibody-drug conjugates using optimized non-natural amino acids in a cell-free expression system. Bioconjug. Chem..

[32] Raucher D, Ryu JS (2015). Cell-penetrating peptides: strategies for anticancer treatment. Trends Mol. Med..

[33] Despanie J, Dhandhukia JP, Hamm-Alvarez SF, MacKay JA (2016). Elastin-like polypeptides: therapeutic applications for an emerging class of nanomedicines. J. Control Release.

[34] Martin RW (2017). Development of a CHO-based cell-free platform for synthesis of active monoclonal antibodies. ACS Synth. Biol..

[35] Heckler TG (1984). T4 RNA ligase mediated preparation of novel “chemically misacylated” tRNAPheS. Biochemistry.

[36] Robertson SA, Noren CJ, Anthony-Cahill SJ, Griffith MC, Schultz PG (1989). The use of 5’-phospho-2 deoxyribocytidylylriboadenosine as a facile route to chemical aminoacylation of tRNA. Nucleic Acids Res..

[37] Robertson SA, Ellman JA, Schultz PG (1991). A general and efficient route for chemical aminoacylation of transfer RNAs. J. Am. Chem. Soc..

[38] Kwiatkowski M, Wang JF, Forster AC (2014). Facile synthesis of N-acyl-aminoacyl-pCpA for preparation of mischarged fully ribo tRNA. Bioconjug. Chem..

[39] Wang JF, Kwiatkowski M, Forster AC (2016). Ribosomal peptide syntheses from activated substrates reveal rate limitation by an unexpected step at the peptidyl site. J. Am. Chem. Soc..

[40] Yamanaka K, Nakata H, Hohsaka T, Sisido M (2004). Efficient synthesis of nonnatural mutants in Escherichia coli S30 in vitro protein synthesizing system. J. Biosci. Bioeng..

[41] Liu DR, Schultz PG (1999). Progress toward the evolution of an organism with an expanded genetic code. Proc. Natl Acad. Sci. USA.

[42] Wang L, Brock A, Herberich B, Schultz PG (2001). Expanding the genetic code of Escherichia coli. Science.

[43] Nozawa K (2009). Pyrrolysyl-tRNA synthetase-tRNA(Pyl) structure reveals the molecular basis of orthogonality. Nature.

[44] Hancock SM, Uprety R, Deiters A, Chin JW (2010). Expanding the genetic code of yeast for incorporation of diverse unnatural amino acids via a pyrrolysyl-tRNA synthetase/tRNA pair. J. Am. Chem. Soc..

[45] Neumann H, Slusarczyk AL, Chin JW (2010). De novo generation of mutually orthogonal aminoacyl-tRNA synthetase/tRNA pairs. J. Am. Chem. Soc..

[46] Chin JW (2014). Expanding and reprogramming the genetic code of cells and animals. Annu Rev. Biochem..

[47] Ellefson JW (2014). Directed evolution of genetic parts and circuits by compartmentalized partnered replication. Nat. Biotechnol..

[48] Schmied WH, Elsasser SJ, Uttamapinant C, Chin JW (2014). Efficient multisite unnatural amino acid incorporation in mammalian cells via optimized pyrrolysyl tRNA synthetase/tRNA expression and engineered eRF1. J. Am. Chem. Soc..

[49] Amiram M (2015). Evolution of translation machinery in recoded bacteria enables multi-site incorporation of nonstandard amino acids. Nat. Biotechnol..

[50] Willis JCW, Chin JW (2018). Mutually orthogonal pyrrolysyl-tRNA synthetase/tRNA pairs. Nat. Chem..

[51] Saito H, Suga H (2001). A ribozyme exclusively aminoacylates the 3’-hydroxyl group of the tRNA terminal adenosine. J. Am. Chem. Soc..

[52] Lee N, Bessho Y, Wei K, Szostak JW, Suga H (2000). Ribozyme-catalyzed tRNA aminoacylation. Nat. Struct. Biol..

[53] Murakami H, Saito H, Suga H (2003). A versatile tRNA aminoacylation catalyst based on RNA. Chem. Biol..

[54] Ramaswamy K, Saito H, Murakami H, Shiba K, Suga H (2004). Designer ribozymes: programming the tRNA specificity into flexizyme. J. Am. Chem. Soc..

[55] Murakami H, Ohta A, Ashigai H, Suga H (2006). A highly flexible tRNA acylation method for non-natural polypeptide synthesis. Nat. Methods.

[56] Chen X, Li N, Ellington AD (2007). Ribozyme catalysis of metabolism in the RNA world. Chem. Biodivers..

[57] Higgs PG, Lehman N (2015). The RNA World: molecular cooperation at the origins of life. Nat. Rev. Genet..

[58] Xiao H, Murakami H, Suga H, Ferre-D’Amare AR (2008). Structural basis of specific tRNA aminoacylation by a small in vitro selected ribozyme. Nature.

[59] Passioura T, Suga H (2013). Flexizyme-mediated genetic reprogramming as a tool for noncanonical peptide synthesis and drug discovery. Chemistry.

[60] Niwa N, Yamagishi Y, Murakami H, Suga H (2009). A flexizyme that selectively charges amino acids activated by a water-friendly leaving group. Bioorg. Med. Chem. Lett..

[61] Carrico IS, Carlson BL, Bertozzi CR (2007). Introducing genetically encoded aldehydes into proteins. Nat. Chem. Biol..

[62] Wu P (2009). Site-specific chemical modification of recombinant proteins produced in mammalian cells by using the genetically encoded aldehyde tag. Proc. Natl Acad. Sci. USA.

[63] Dirksen A, Dawson PE (2008). Rapid oxime and hydrazone ligations with aromatic aldehydes for biomolecular labeling. Bioconjug. Chem..

[64] Saito H, Watanabe K, Suga H (2001). Concurrent molecular recognition of the amino acid and tRNA by a ribozyme. RNA.

[65] Goto Y (2008). Reprogramming the translation initiation for the synthesis of physiologically stable cyclic peptides. ACS Chem. Biol..

[66] Saito H, Kourouklis D, Suga H (2001). An in vitro evolved precursor tRNA with aminoacylation activity. EMBO J..

[67] Goto Y, Katoh T, Suga H (2011). Flexizymes for genetic code reprogramming. Nat. Protoc..

[68] Das R, Baker D (2008). Macromolecular modeling with Rosetta. Annu. Rev. Biochem..

[69] Carlson ED, Gan R, Hodgman CE, Jewett MC (2012). Cell-free protein synthesis: applications come of age. Biotechnol. Adv..

[70] Kwon YC, Jewett MC (2015). High-throughput preparation methods of crude extract for robust cell-free protein synthesis. Sci. Rep..

[71] Jaroentomeechai, T. et al. Single-pot glycoprotein biosynthesis using a cell-free transcription-translation system enriched with glycosylation machinery. *Nat. Commun.* **9**, 2686 (2018). 10.1038/s41467-018-05110-x PMC6043479 30002445

[72] Kightlinger W (2018). Design of glycosylation sites by rapid synthesis and analysis of glycosyltransferases. Nat. Chem. Biol..

[73] Oza, J. P. et al. Robust production of recombinant phosphoproteins using cell-free protein synthesis. *Nat. Commun.* **6**, 8168 (2015). 10.1038/ncomms9168 PMC4566161 26350765

[74] Shimizu Y (2001). Cell-free translation reconstituted with purified components. Nat. Biotechnol..

[75] Iwane Y, Katoh T, Goto Y, Suga H (2018). Artificial division of codon boxes for expansion of the amino acid repertoire of ribosomal polypeptide synthesis. Methods Mol. Biol..

[76] Udagawa T, Shimizu Y, Ueda T (2004). Evidence for the translation initiation of leaderless mRNAs by the intact 70 S ribosome without its dissociation into subunits in eubacteria. J. Biol. Chem..

[77] Uttamapinant C (2012). Fast, cell-compatible click chemistry with copper-chelating azides for biomolecular labeling. Angew. Chem. Int Ed. Engl..

[78] Rabuka D, Rush JS, deHart GW, Wu P, Bertozzi CR (2012). Site-specific chemical protein conjugation using genetically encoded aldehyde tags. Nat. Protoc..

[79] Rosen CB, Francis MB (2017). Targeting the N terminus for site-selective protein modification. Nat. Chem. Biol..

[80] Kolmel DK, Kool ET (2017). Oximes and hydrazones in bioconjugation: mechanism and catalysis. Chem. Rev..

[81] Tsiamantas C, Kwon S, Douat C, Huc I, Suga H (2019). Optimizing aromatic oligoamide foldamer side-chains for ribosomal translation initiation. Chem. Commun..

[82] Ad O (2019). Translation of diverse aramid- and 1,3-dicarbonyl-peptides by wild type ribosomes in vitro. ACS Cent. Sci..

[83] Orelle C (2015). Protein synthesis by ribosomes with tethered subunits. Nature.

[84] Fried SD, Schmied WH, Uttamapinant C, Chin JW (2015). Ribosome subunit stapling for orthogonal translation in E. coli. Angew. Chem. Weinh. Bergstr. Ger..

[85] Liu Y, Kim DS, Jewett MC (2017). Repurposing ribosomes for synthetic biology. Curr. Opin. Chem. Biol..

[86] d’Aquino AE, Kim DS, Jewett MC (2018). Engineered ribosomes for basic science and synthetic biology. Annu. Rev. Chem. Biomol..

[87] Schmied WH (2018). Controlling orthogonal ribosome subunit interactions enables evolution of new function. Nature.

[88] Carlson ED (2019). Engineered ribosomes with tethered subunits for expanding biological function. Nat. Commun..

[89] Aleksashin NA (2019). Assembly and functionality of the ribosome with tethered subunits. Nat. Commun..

[90] Hammerling, M. J. et al. In vitro ribosome synthesis and evolution through ribosome display. *bioRxiv*, 692111 (2019). 10.1038/s41467-020-14705-2 PMC7048773 32111839

