# Supplementary material for: Expanding the limits of the second genetic code with ribozymes
Source: Nat Commun. 2019 Nov 8;10:5097. doi: 10.1038/s41467-019-12916-w (PMC6841967; doi:10.1038/s41467-019-12916-w)
Supplement: Supplementary file 1 — Supplementary Information [file 41467_2019_12916_MOESM1_ESM.pdf]

Supplementary Information

Expanding the limits of the second genetic code with ribozymes

Joongoo Lee[1,*], Kenneth E. Schwieter[2,*], Andrew M. Watkins[3], Do Soon Kim[1], Hao Yu[4], Kevin J. Schwarz[2], Jongdoo Lim[5], Jaime Coronado[5], Michelle Byrom[6], Eric V. Anslyn[5], Andrew D. Ellington[6], Jeffrey S. Moore[2,7], Michael C. Jewett[1]

1. Department of Chemical and Biological Engineering, Northwestern University, Evanston, Illinois 60208
2. Department of Chemistry, University of Illinois at Urbana−Champaign, Urbana, Illinois 61801
3. Departments of Biochemistry and Physics, Stanford University, Stanford, California 94305
4. Departments of Chemical and Biomolecular Engineering, University of Illinois at Urbana−Champaign, Urbana, Illinois 61801
5. Department of Chemistry, University of Texas at Austin, Austin, Texas, 78712
6. Department of Chemistry and Biochemistry, Institute for Cellular and Molecular Biology, University of Texas at Austin, Austin, Texas 78712
7. The Beckman Institute for Advanced Science and Technology, University of Illinois at Urbana-Champaign, Urbana, IL 61801

*These authors contributed equally to this work.

To whom correspondence should be addressed. E-mail: jsmoore@illinois.edu or m-jewett@northwestern.edu

Contents

Supplementary Methods 6

Materials and Methods 6

Characterizations of substrates 6

Preparation of DNA templates for RNAs 21

1) Extension (Generation of Fx derivatives by extending different 3'-ends) 21

2) PCR amplification 21

3) DNA precipitation 22

In-vitro transcription 22

Digestion of DNA templates 22

Purification of *in-vitro* transcribed RNA 22

Precipitation of tRNA 23

Peptide purification 23

Characterization of peptides 24

Supplementary Tables and Figures 25

Supplementary Figure 1. Acylation of microhelix with substrates A-G. 25

Supplementary Figure 2. Undesired hydrolysis of acylated microhelix. 26

Supplementary Figure 3. Numerical acylation yields of microhelix obtained using the expanded substrates. 27

Supplementary Figure 4. Analysis of acylation with 1-6. 28

Supplementary Figure 5. Analysis of acylation with 7-21. 29

Supplementary Figure 6. Analysis of acylation with 22-32. 30

Supplementary Figure 7. Acylation test of pyrrole-ABT and thiophene-ABT 31

Supplementary Figure 8. Results of computational modeling. 32

Supplementary Figure 9. Reprogramming fMet^{tRNA} with noncanonical substrates. 33

Supplementary Figure 10. Characterization of the N-terminus functionalized peptide with the Phe analogues 34

Supplementary Figure 11. Characterization of the N-terminus functionalized peptide with the EWG-containing benzoic acid derivatives. 35

Supplementary Figure 12. Characterization of the N-terminus functionalized peptide with the EDG-containing benzoic acids. 36

Supplementary Figure 13. Characterization of the N-terminus functionalized peptide with the benzoic acid derivatives containing both EWG and EDG 37

Supplementary Figure 14. Characterization of the N-terminus functionalized peptide with the heteroaromatic substrates. 38

Supplementary Figure 15. Characterization of the N-terminus functionalized peptide with the aliphatic substrates. 39
Supplementary Figure 16. Translation efficiency for the noncanonical substrates. 40
Supplementary Figure 17. Percent yield of peptides containing the noncanonical chemical substrates. 41
Supplementary Figure 18. Characterization of the N-terminus functionalized peptide with the aldehyde and hydrazine substrates. 43
Supplementary Figure 19. ^{1}H NMR (500 MHz, $CDCl_3$) of B. 44
Supplementary Figure 20. ^{13}C NMR (125 MHz, $CDCl_3$) of B. 44
Supplementary Figure 21. ^{1}H NMR (500 MHz, $CDCl_3$) of C. 45
Supplementary Figure 22. ^{13}C NMR (125 MHz, $CDCl_3$) of C. 45
Supplementary Figure 23. ^{1}H NMR (500 MHz, $CDCl_3$) of D. 46
Supplementary Figure 24. ^{13}C NMR (125 MHz, $CDCl_3$) of D. 46
Supplementary Figure 25. ^{1}H NMR (500 MHz, $CDCl_3$) of E. 47
Supplementary Figure 26. ^{13}C NMR (125 MHz, $CDCl_3$) of E. 47
Supplementary Figure 27. ^{1}H NMR (500 MHz, $CDCl_3$) of F. 48
Supplementary Figure 28. ^{13}C NMR (125 MHz, $CDCl_3$) of F. 48
Supplementary Figure 29. ^{1}H NMR (500 MHz, DMSO-d_6) of 1. 49
Supplementary Figure 30. ^{13}C NMR (125 MHz, DMSO-d_6) of 1. 49
Supplementary Figure 31. ^{1}H NMR (500 MHz, DMSO-d_6) of 2. 50
Supplementary Figure 32. ^{13}C NMR (125 MHz, DMSO-d_6) of 2. 50
Supplementary Figure 33. ^{1}H NMR (500 MHz, $CDCl_3$) of 3. 51
Supplementary Figure 34. ^{13}C NMR (125 MHz, $CDCl_3$) of 3. 51
Supplementary Figure 35. ^{1}H NMR (500 MHz, CD_3CN) of 4. 52
Supplementary Figure 36. ^{13}C NMR (125 MHz, CD_3CN) of 4. 52
Supplementary Figure 37. ^{1}H NMR (500 MHz, DMSO-d_6) of 5. 53
Supplementary Figure 38. ^{13}C NMR (125 MHz, DMSO-d_6) of 5. 53
Supplementary Figure 39. ^{1}H NMR (500 MHz, DMSO-d_6) of 6. 54
Supplementary Figure 40. ^{13}C NMR (125 MHz, DMSO-d_6) of 6. 54
Supplementary Figure 41. ^{1}H NMR (500 MHz, $CDCl_3$) of 7. 55
Supplementary Figure 42. ^{13}C NMR (125 MHz, $CDCl_3$) of 7. 55
Supplementary Figure 43. ^{1}H NMR (500 MHz, $CDCl_3$) of 8. 56
Supplementary Figure 44. ^{13}C NMR (125 MHz, $CDCl_3$) of 8. 56
Supplementary Figure 45. ^{1}H NMR (500 MHz, CD_3CN) of 9. 57
Supplementary Figure 46. ^{13}C NMR (125 MHz, CD_3CN) of 9. 57

Supplementary Figure 47. ^{1}H NMR (500 MHz, $CDCl_3$) of 10. 58
Supplementary Figure 48. ^{13}C NMR (125 MHz, $CDCl_3$) of 10. 58
Supplementary Figure 49. ^{1}H NMR (500 MHz, $CDCl_3$) of 11. 59
Supplementary Figure 50. ^{13}C NMR (125 MHz, $CDCl_3$) of 11. 59
Supplementary Figure 51. ^{1}H NMR (500 MHz, $CDCl_3$) of 12. 60
Supplementary Figure 52. ^{13}C NMR (125 MHz, $CDCl_3$) of 12. 60
Supplementary Figure 53. ^{1}H NMR (500 MHz, $CDCl_3$) of 13. 61
Supplementary Figure 54. ^{13}C NMR (125 MHz, $CDCl_3$) of 13. 61
Supplementary Figure 55. ^{1}H NMR (500 MHz, $CDCl_3$) of 14. 62
Supplementary Figure 56. ^{13}C NMR (125 MHz, $CDCl_3$) of 14. 62
Supplementary Figure 57. ^{1}H NMR (500 MHz, $CDCl_3$) of 15. 63
Supplementary Figure 58 ^{13}C NMR (125 MHz, $CDCl_3$) of 15. 63
Supplementary Figure 59. ^{1}H NMR (500 MHz, $CDCl_3$) of 16. 64
Supplementary Figure 60. ^{13}C NMR (125 MHz, $CDCl_3$) of 16. 64
Supplementary Figure 61. ^{1}H NMR (500 MHz, $CDCl_3$) of 17. 65
Supplementary Figure 62. ^{13}C NMR (125 MHz, $CDCl_3$) of 17. 65
Supplementary Figure 63. ^{1}H NMR (500 MHz, DMSO-d_6) of 18. 66
Supplementary Figure 64. ^{13}C NMR (125 MHz, DMSO-d_6) of 18. 66
Supplementary Figure 65. ^{1}H NMR (500 MHz, $CDCl_3$) of 19. 67
Supplementary Figure 66. ^{13}C NMR (125 MHz, $CDCl_3$) of 19. 67
Supplementary Figure 67. ^{1}H NMR (500 MHz, DMSO-d_6) of 20. 68
Supplementary Figure 68. ^{13}C NMR (125 MHz, DMSO-d_6) of 20. 68
Supplementary Figure 69. ^{1}H NMR (500 MHz, acetone-d_6) of 21. 69
Supplementary Figure 70. ^{13}C NMR (125 MHz, acetone-d_6) of 21. 69
Supplementary Figure 71. ^{1}H NMR (500 MHz, $CDCl_3$) of 22. 70
Supplementary Figure 72. ^{13}C NMR (125 MHz, $CDCl_3$) of 22. 70
Supplementary Figure 73. ^{1}H NMR (500 MHz, $CDCl_3$) of 23. 71
Supplementary Figure 74. ^{13}C NMR (125 MHz, $CDCl_3$) of 23. 71
Supplementary Figure 75. ^{1}H NMR (500 MHz, $CDCl_3$) of 24. 72
Supplementary Figure 76. ^{13}C NMR (125 MHz, $CDCl_3$) of 24. 72
Supplementary Figure 77. ^{1}H NMR (500 MHz, DMSO-d_6) of 25. 73
Supplementary Figure 78. ^{13}C NMR (125 MHz, DMSO-d_6) of 25. 73
Supplementary Figure 79. ^{1}H NMR (500 MHz, $CDCl_3$) of Boc-25a. 74
Supplementary Figure 80. ^{13}C NMR (500 MHz, $CDCl_3$) of Boc-25a. 74

Supplementary Figure 81. ^{1}H NMR (500 MHz, $CDCl_3$) of 26. 75
Supplementary Figure 82. ^{13}C NMR (125 MHz, $CDCl_3$) of 26. 75
Supplementary Figure 83. ^{1}H NMR (500 MHz, $CDCl_3$) of Boc-26a. 76
Supplementary Figure 84. ^{13}C NMR (500 MHz, $CDCl_3$) of Boc-26a. 76
Supplementary Figure 85. ^{1}H NMR (500 MHz, $CDCl_3$) of Boc-G. 77
Supplementary Figure 86. ^{13}C NMR (125 MHz, $CDCl_3$) of Boc-G. 77
Supplementary Figure 87. ^{1}H NMR (500 MHz, $CDCl_3$) of Boc-27. 78
Supplementary Figure 88. ^{13}C NMR (125 MHz, $CDCl_3$) of Boc-27. 78
Supplementary Figure 89. ^{1}H NMR (500 MHz, $CDCl_3$) of Boc-28. 79
Supplementary Figure 90. ^{13}C NMR (125 MHz, $CDCl_3$) of Boc-28. 79
Supplementary Figure 91. ^{1}H NMR (500 MHz, $CDCl_3$) of Boc-29. 80
Supplementary Figure 92. ^{13}C NMR (125 MHz, $CDCl_3$) of Boc-29. 80
Supplementary Figure 93. ^{1}H NMR (500 MHz, $CDCl_3$) of Boc-30. 81
Supplementary Figure 94. ^{13}C NMR (125 MHz, $CDCl_3$) of Boc-30. 81
Supplementary Figure 95. ^{1}H NMR (500 MHz, $CDCl_3$) of Boc-31. 82
Supplementary Figure 96. ^{13}C NMR (125 MHz, $CDCl_3$) of Boc-31. 82
Supplementary Figure 97. ^{1}H NMR (500 MHz, $CDCl_3$) of Boc-32. 83
Supplementary Figure 98. ^{13}C NMR (125 MHz, $CDCl_3$) of Boc-32. 83
Supplementary Figure 99. ^{1}H NMR (500 MHz, DMSO-d_6) of 33. 84
Supplementary Figure 100. ^{13}C NMR (125 MHz, DMSO-d_6) of 33. 84
Supplementary Figure 101. ^{1}H NMR (500 MHz, $CDCl_3$) of 34. 85
Supplementary Figure 102. ^{13}C NMR (125 MHz, $CDCl_3$) of 34. 85
Supplementary Figure 103. ^{1}H NMR (500 MHz, DMSO-d_6) of 35. 86
Supplementary Figure 104. ^{13}C NMR (125 MHz, DMSO-d_6) of 35. 86
Supplementary Figure 105. ^{1}H NMR (500 MHz, DMSO-d_6) of 36. 87
Supplementary Figure 106. ^{13}C NMR (125 MHz, DMSO-d_6) of 36. 87
Supplementary Figure 107. ^{1}H NMR (400 MHz, $CDCl_3$) of Boc-37. 88
Supplementary Figure 108. ^{13}C NMR (100 MHz, $CDCl_3$) of Boc-37. 88
Supplementary Figure 109. ^{1}H NMR (400 MHz, $CDCl_3$) of Boc-38. 89
Supplementary Figure 110. ^{13}C NMR (100 MHz, $CDCl_3$) of Boc-38. 89
Plasmid map 90
References 91

Supplementary Methods

Materials and Methods

All reagents and solvents were commercial grade and purified prior to use when necessary. Dichloromethane was dried by passage through a column of activated alumina as described by Grubbs.[1] Phenylalanine cyanomethyl ester (**A**) was prepared as recently described.[2]

Tert-butyl (2-(4-(mercaptomethyl)benzamido)ethyl) carbamate (ABT) was prepared according to the standard procedure.[3] All organic solutions were dried over $MgSO_4$. Thin layer chromatography (TLC) was performed using glass-backed silica gel (250 μm) plates. Flash chromatography was performed on a Biotage Isolera One automated purification system. UV light, and/or the use of $KMnO_4$ were used to visualize products.

Nuclear magnetic resonance spectra (NMR) were acquired on a Bruker Advance III-500 (500 MHz) or Varian Unity 500 (500 MHz) instrument. Chemical shifts are measured relative to residual solvent peaks as an internal standard set to δ 7.26 and δ 77.0 ($CDCl_3$), and δ 2.50 and δ 39.5 (DMSO-d_6). Mass spectra were recorded on a Bruker AmaZon SL or Waters Q-TOF Ultima (ESI) and Impact-II or Waters 70-VSE (EI), spectrometers by use of the ionization method noted.

Characterizations of substrates

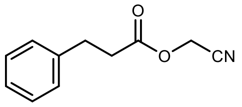

Cyanomethyl 3-phenylpropanoate (B). Prepared according to the general procedure using 3-phenylpropanoic acid (100 mg, 0.66 mmol), triethylamine (140 μL, 0.99 mmol), chloroacetonitrile (53 μL, 0.79 mmol) and dichloromethane (0.7 mL). The product was obtained as a clear oil (95 mg, 77%). ^{1}H NMR (500 MHz, $CDCl_3$) δ 7.33 (t, J = 7.6 Hz, 2H), 7.28-7.21 (m, 3H), 4.72 (s, 2H), 3.01 (t, J = 7.8 Hz, 2H), 2.76 (t, J = 7.8 Hz, 2H); ^{13}C NMR (125 MHz, $CDCl_3$) ppm 171.2, 139.5, 128.6, 128.2, 126.6, 114.3, 48.2, 35.1, 30.5; HRMS (EI): Exact mass calcd for $C_{11}H_{11}NO_2$ $[M]^+$ 189.07898, found 189.07881.

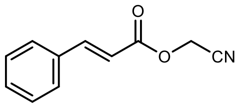

Cyanomethyl *trans*-cinnamate (C). Prepared according to the general procedure using *trans*-cinnamic acid (98 mg, 0.66 mmol), triethylamine (140 μL, 0.99 mmol), chloroacetonitrile (53 μL, 0.79 mmol) and dichloromethane (0.7 mL). The product was obtained as a white solid (78 mg, 63%). ^{1}H NMR (500 MHz, $CDCl_3$) δ 7.80 (d, J = 16.0 Hz, 1H), 7.57-7.53 (m, 2H), 7.44-7.40 (m, 3H), 6.46 (d, J = 16.1 Hz, 1H), 4.86 (s, 2H); ^{13}C NMR (125 MHz, $CDCl_3$) ppm 165.1, 147.7, 133.6, 131.1, 129.0,

128.4, 115.2, 114.5, 48.4; HRMS (EI): Exact mass calcd for $C_{11}H_9NO_2$ $[M]^+$ 187.0633, found 187.0633.

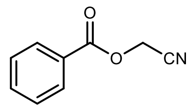

Cyanomethyl benzoate (D). Prepared according to the general procedure using benzoic acid (81 mg, 0.66 mmol), triethylamine (140 µL, 0.99 mmol), chloroacetonitrile (53 µL, 0.79 mmol) and dichloromethane (0.7 mL). The product was obtained as a clear oil (87 mg, 82%). ^{1}H NMR (500 MHz, $CDCl_3$) δ 8.06 (dd, J = 8.3, 1.4 Hz, 2H), 7.67-7.59 (m, 1H), 7.49 (t, J = 7.8 Hz, 2H), 4.97 (s, 2H); ^{13}C NMR (125 MHz, $CDCl_3$) ppm 164.9, 134.1, 130.0, 128.7, 127.8, 114.4, 48.8; HRMS (EI): Exact mass calcd for $C_9H_7NO_2$ $[M]^+$ 161.0477, found 161.0475.

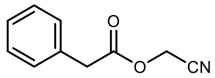

Cyanomethyl 2-phenylacetate (E). Prepared according to the general procedure using phenylacetic acid (90 mg, 0.66 mmol), triethylamine (140 µL, 0.99 mmol), chloroacetonitrile (53 µL, 0.79 mmol) and dichloromethane (0.7 mL). The product was obtained as a white solid (79 mg, 68%). ^{1}H NMR (500 MHz, $CDCl_3$) δ 7.35-7.23 (m, 5H), 4.70 (s, 2H), 3.70 (s, 2H); ^{13}C NMR (125 MHz, $CDCl_3$) ppm 169.9, 132.2, 129.2, 128.8, 127.6, 114.2, 48.6, 40.4; HRMS (EI): Exact mass calcd for $C_{10}H_9NO_2$ $[M]^+$ 175.0633, found 175.0634.

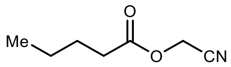

Cyanomethyl pentanoate (F). Prepared according to the general procedure using valeric acid (72 µL, 0.66 mmol), triethylamine (140 µL, 0.99 mmol), chloroacetonitrile (53 µL, 0.79 mmol) and dichloromethane (0.7 mL). The product was obtained as a clear oil (65 mg, 70%). ^{1}H NMR (500 MHz, $CDCl_3$) δ 4.71 (s, 2H), 2.41 (t, J = 7.5 Hz, 2H), 1.67-1.60 (m, 2H), 1.41-1.30 (m, 2H), 0.92 (t, J = 7.4 Hz, 3H); ^{13}C NMR (125 MHz, $CDCl_3$) ppm 172.1, 114.5, 48.1, 33.1, 26.6, 22.1, 13.6; HRMS (CI): Exact mass calcd for $C_7H_{12}NO_2$ $[M+H]^+$ 142.0868, found 142.0867.

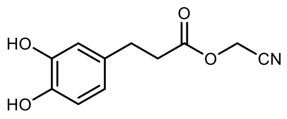

Cyanomethyl 3-(3,4-dihydroxyphenyl)propanoate (1). Prepared according to the general procedure using 3-(3,4-dihydroxyphenyl)propanoic acid (60 mg, 0.33 mmol), triethylamine (70 µL, 0.5 mmol), chloroacetonitrile (26.5 µL, 0.4 mmol) and dichloromethane (0.2 mL). The product was obtained as a brown solid (40 mg, 55%). ^{1}H-NMR (500 MHz, DMSO-d_6) δ 8.73 (s, 1H), 8.67 (s, 1H), 6.61 (d, J = 8.1 Hz, 1H), 6.58 (d, J = 1.9 Hz, 1H), 6.46-6.44 (m, 1H), 4.94 (s, 2H), 2.69-

2.68 (m, 2H), 2.66-2.64 (m, 2H); ^{13}C NMR (125 MHz, DMSO-d_6) ppm 171.9, 145.5, 144.0, 131.3, 119.2, 116.4, 116.1, 115.9, 49.3, 35.2, 29.8; HRMS (EI): Exact mass calcd for $C_{11}H_{11}NO_4$: $[M]^+$ 221.0688, found 221.0690.

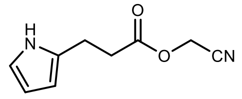

Cyanomethyl 3-(1*H*-pyrrol-2-yl)propanoate (2). Prepared according to the general procedure using 3-(1*H*-pyrrol-2-yl)propanoic acid (46 mg, 0.33 mmol), triethylamine (70 µL, 0.5 mmol), chloroacetonitrile (26.5 µL, 0.4 mmol) and dichloromethane (0.2 mL). The product was obtained as a brown solid (45 mg, 77%). ^1H-NMR (500 MHz, DMSO-d_6) δ 10.54 (s, 1H), 6.58 (d, J = 2.0 Hz, 1H), 5.88 (q, J = 2.7, 3.0, 2.6 Hz, 1H), 5.74 (m, 1H), 4.96 (s, 2H), 2.81 (t, J = 8 Hz, 2H), 2.70 (t, J = 7 Hz, 2H); ^{13}C NMR (125 MHz, DMSO-d_6) ppm 171.9, 130.0, 116.8, 116.5, 107.6, 105.0, 49.4, 33.6, 22.8; HRMS (EI): Exact mass calcd for $C_9H_{10}N_2O_2$: $[M]^+$178.0742, found 178.0743.

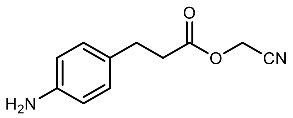

Cyanomethyl 3-(4-aminophenyl)propanoate (3). Prepared according to the general procedure using 3-(4-aminophenyl)propanoic acid (109 mg, 0.66 mmol), triethylamine (140 µL, 0.99 mmol), chloroacetonitrile (53 µL, 0.79 mmol) and dichloromethane (0.7 mL). The product was obtained as a white solid (123 mg, 55%). ^1H NMR (500 MHz, $CDCl_3$) δ 6.98 (d, J = 8.2 Hz, 2H), 6.63 (d, J = 8.2 Hz, 2H), 4.68 (s, 2H), 3.48 (br s, 2H), 2.87 (t, J = 7.7 Hz, 2H), 2.67 (t, J = 7.7 Hz, 2H); ^{13}C NMR (125 MHz, $CDCl_3$) ppm 171.4, 144.8, 129.5, 129.0, 115.3, 114.4, 48.1, 35.5, 29.8; HRMS (EI): Exact mass calcd for $C_{11}H_{12}N_2O_2$ $[M]^+$ 204.0899, found 204.0897.

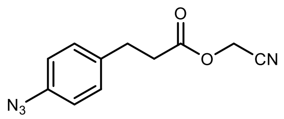

Cyanomethyl 3-(4-azidophenyl)propanoate (4). Prepared according to the general procedure using 3-(4-azidophenyl)propanoic acid (126 mg, 0.66 mmol), triethylamine (140 µL, 0.99 mmol), chloroacetonitrile (53 µL, 0.79 mmol) and dichloromethane (0.7 mL). The product was obtained as a red oil (123 mg, 81%). ^1H NMR (500 MHz, CD_3CN) δ 7.25 (d, J = 8.5 Hz, 2H), 7.00 (d, J = 8.4 Hz, 2H), 4.72 (s, 2H), 2.91 (t, J = 7.6 Hz, 2H), 2.70 (t, J = 7.6 Hz, 2H); ^{13}C NMR (125 MHz, CD_3CN) ppm 172.4, 139.0, 138.1, 130.8, 119.9, 116.2, 49.6, 35.4, 30.3; HRMS (EI): Exact mass calcd for $C_{11}H_{10}N_4O_2$ $[M]^+$ 230.0804, found 230.0794.

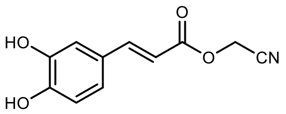

Cyanomethyl (*E*)-3-(3,4-dihydroxyphenyl)acrylate (5). Prepared according to the general procedure using (*E*)-3-(3,4-

dihydroxyphenyl)acrylic acid (59 mg, 0.33 mmol), triethylamine (70 µL, 0.5 mmol), chloroacetonitrile (26.5 µL, 0.4 mmol) and dichloromethane (0.2 mL). The product was obtained as a pink solid (41 mg, 57%). ^{1}H-NMR (500 MHz, DMSO-*d*$_6$) δ 9.71 (s, 1H), 9.20 (s, 1H), 7.61 (m, 1H), 7.10 (d, *J* = 1.8 Hz, 1H), 7.07 (dd, *J* = 8.3, 1.7 Hz, 1H), 6.78 (d, J = 8.4 Hz, 1H), 6.35 (d, *J* = 16.3 Hz, 1H), 5.06 (s, 2H); ^{13}C NMR (125 MHz, DMSO-*d*$_6$) ppm 165.9, 149.5, 147.9, 146.1, 125.6, 122.5, 116.7, 116.2, 115.6, 112.0, 49.3; HRMS (EI): Exact mass calcd for $C_{11}H_9NO_4$: $[M]^+$ 219.0532, found 219.0531.

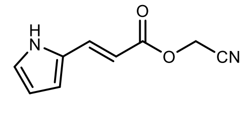

Cyanomethyl (*E*)-3-(1H-pyrrol-2yl)acrylate (6). Prepared according to the general procedure using (*E*)-3-(1*H*-pyrrol-2-yl)acrylic acid (45 mg, 0.33 mmol), triethylamine (70 µL, 0.5 mmol), chloroacetonitrile (26.5 µL, 0.4 mmol) and dichloromethane (0.2 mL). The product was obtained as a brown solid (24 mg, 43%). ^{1}H-NMR (500 MHz, DMSO-*d*$_6$) δ 11.65 (s, 1H), 7.56 (d, *J* = 15.6 Hz, 1H), 7.11 (m, 1H), 6.67 (m, 1H), 6.24 (d, *J* = 15.8 Hz, 1H), 6.22-6.20 (m, 1H), 5.02 (s, 2H); ^{13}C NMR (125 MHz, DMSO-*d*$_6$) ppm 166.2, 137.3, 128.4, 125.0, 116.8, 116.7, 110.9, 107.8, 49.2; HRMS (EI): Exact mass calcd for $C_9H_8N_2O_2$: $[M]^+$ 176.0586, found 176.0586.

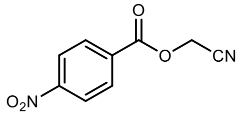

Cyanomethyl 4-nitrobenzoate (7). Prepared according to the general procedure using 4-nitrobenzoic acid (110 mg, 0.66 mmol), triethylamine (140 µL, 0.99 mmol), chloroacetonitrile (53 µL, 0.79 mmol) and dichloromethane (0.7 mL). The product was obtained as a beige solid (69 mg, 51%). ^{1}H NMR (500 MHz, $CDCl_3$) δ 8.34 (d, *J* = 8.9 Hz, 2H), 8.26 (d, *J* = 9.0 Hz, 2H), 5.03 (s, 2H); ^{13}C NMR (125 MHz, $CDCl_3$) ppm 163.2, 151.2, 133.1, 131.2, 123.9, 113.8, 49.5; HRMS (EI): Exact mass calcd for $C_9H_6N_2O_4$ $[M]^+$ 206.03276, found 206.03188.

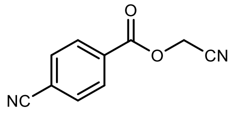

Cyanomethyl 4-cyanobenzoate (8). Prepared according to the general procedure using 4-cyanobenzoic acid (97 mg, 0.66 mmol), triethylamine (140 µL, 0.99 mmol), chloroacetonitrile (53 µL, 0.79 mmol) and dichloromethane (0.7 mL). The product was obtained as a white solid (101 mg, 82%). ^{1}H NMR (500 MHz, $CDCl_3$) δ 8.18 (d, *J* = 8.5 Hz, 2H), 7.80 (d, *J* = 8.5 Hz, 2H), 5.01 (s, 2H); ^{13}C NMR (125 MHz, $CDCl_3$) ppm 163.4, 132.5, 131.6, 130.5, 124.8, 117.6, 113.9, 49.4; HRMS (EI): Exact mass calcd for $C_{10}H_6N_2O_2$ $[M]^+$ 186.0429, found 186.0426.

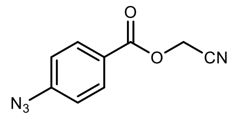

Cyanomethyl 4-azidobenzoate (9). Prepared according to the general procedure using 4-azidobenzoic acid (108 mg, 0.66 mmol), triethylamine (140 µL, 0.99 mmol), chloroacetonitrile (53 µL, 0.79 mmol) and dichloromethane (0.7 mL). The product was obtained as a red oil (89 mg, 67%). ^{1}H NMR (500 MHz, CD_3CN) δ 8.02 (d, *J* = 8.7 Hz, 2H), 7.17 (d, *J* = 8.7 Hz, 2H), 4.97 (s, 2H); ^{13}C NMR (125 MHz, CD_3CN) ppm 165.2, 146.8, 132.4, 125.6, 120.2, 116.2, 50.3; HRMS (EI): Exact mass calcd for $C_9H_6N_4O_2$ $[M]^+$ 202.0491, found 202.0487.

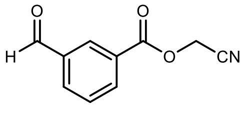

Cyanomethyl 3-formylbenzoate (10). Prepared according to the general procedure using 3-formylbenzoic acid (99 mg, 0.66 mmol), triethylamine (140 µL, 0.99 mmol), chloroacetonitrile (53 µL, 0.79 mmol) and dichloromethane (0.7 mL). The product was obtained as a clear oil (95 mg, 69%). ^{1}H NMR (500 MHz, $CDCl_3$) δ 10.09 (s, 1H), 8.55 (t, *J* = 1.7 Hz, 1H), 8.32 (d, *J* = 7.8 Hz, 1H), 8.16 (d, *J* = 7.7 Hz, 1H), 7.69 (t, *J* = 7.7 Hz, 1H), 5.02 (s, 2H); ^{13}C NMR (125 MHz, $CDCl_3$) ppm 190.9, 163.9, 136.7, 135.4, 134.3, 131.4, 129.7, 129.0, 114.1, 49.2; HRMS (EI): Exact mass calcd for $C_{10}H_6NO_3$ $[M]^+$ 189.0347, found 189.0344.

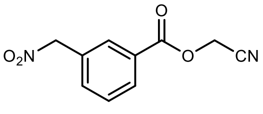

Cyanomethyl 3-(nitromethyl)benzoate (11). Prepared according to the general procedure using 3-bromobenzoic acid (500 mg, 2.49 mmol), triethylamine (520 µL, 3.74 mmol), chloroacetonitrile (188 µL, 2.99 mmol) and dichloromethane (2.5 mL). The product was obtained as a white oily solid (579 mg, 97%). ^{1}H NMR (500 MHz, $CDCl_3$) δ 8.20 (dd, *J* = 1.8, 1.8 Hz, 1H), 8.00 (ddd, *J* = 7.8, 1.7, 1.1 Hz, 1H), 7.76 (ddd, *J* = 8.0, 2.0, 1.1 Hz, 1H), 7.38 (dd, *J* = 7.9, 7.9 Hz, 1H), 4.97 (s, 2H); ^{13}C NMR (125 MHz, $CDCl_3$) ppm 163.5, 136.9, 132.7, 130.2, 129.6, 128.4, 122.6, 114.2, 49.0; HRMS (EI): Exact mass calcd for $C_9H_6NO_2Br$ $[M]^+$ 238.95818, found 238.95761.According to literature procedure, to a flame-dried glass vial under an argon atmosphere was added cyanomethyl 3-bromobenzoate (192 mg, 0.80 mmol), K_3PO_4 (204 mg, 0.96 mmol), XPhos (23.9 mg, 0.05 mmol), Pd_2dba_3 (18.3 mg, 0.02 mmol), nitromethane (430 µL, 8.0 mmol) and dioxane (3.6 mL). The reaction mixture was stirred at 70 °C for 24 h. After cooling to room temperature, the mixture was diluted with CH_2Cl_2 and washed with 1 M HCl. The organic phase was dried ($MgSO_4$) and concentrated. Flash column chromatography (SiO_2, 10-35% ethyl acetate in hexanes) yielded the product as a yellow oil (120 mg, 68%). ^{1}H NMR (500 MHz, $CDCl_3$) δ 8.16 (s, 1H), 8.15 (d, *J* = 8.7 Hz, 1H), 7.74 (d, *J* = 7.8 Hz, 1H), 7.59 (dd, *J* = 7.7, 7.7 Hz, 1H), 5.51 (s,

2H), 4.99 (s, 2H); ^{13}C NMR (125 MHz, $CDCl_3$) ppm 164.0, 135.5, 131.6, 131.5, 130.3, 129.7, 128.9, 114.2, 79.1, 49.1; HRMS (CI): Exact mass calcd for $C_{10}H_9N_2O_4$ $[M+H]^+$ 221.0562, found 221.0558.

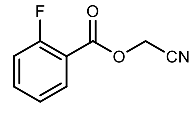

Cyanomethyl 2-fluorobenzoate (12). Prepared according to the general procedure using 2-fluorobenzoic acid (92 mg, 0.66 mmol), triethylamine (140 μL, 0.99 mmol), chloroacetonitrile (53 μL, 0.79 mmol) and dichloromethane (0.7 mL). The product was obtained as a red oil (66 mg, 56%). ^1H NMR (500 MHz, $CDCl_3$) δ 7.98 (td, J = 7.5, 1.8 Hz, 1H), 7.61 (tdd, J = 7.0, 5.9, 3.3 Hz, 1H), 7.26 (td, J = 7.7, 1.1 Hz, 1H), 7.19 (ddd, J = 10.7, 8.4, 1.1 Hz, 1H), 4.98 (s, 2H); ^{13}C NMR (125 MHz, $CDCl_3$) ppm 162.6 (d, $^3J_{CF}$ = 3.6 Hz), 162.2 (d, $^1J_{CF}$ = 262.4 Hz), 135.9 (d, $^3J_{CF}$ = 9.1 Hz), 132.3, 124.2 (d, $^3J_{CF}$ = 4.0 Hz), 117.2 (d, $^2J_{CF}$ = 21.9 Hz), 116.3 (d, $^2J_{CF}$ = 9.3 Hz), 114.2, 48.8; HRMS (EI): Exact mass calcd for $C_9H_6FNO_2$ $[M]^+$ 179.0383, found 179.0383.

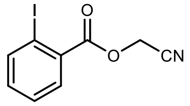

Cyanomethyl 2-iodobenzoate (13). Prepared according to the general procedure using 2-iodobenzoic acid (164 mg, 0.66 mmol), triethylamine (140 μL, 0.99 mmol), chloroacetonitrile (53 μL, 0.79 mmol) and dichloromethane (0.7 mL). The product was obtained as a red oil (129 mg, 68%). ^1H NMR (500 MHz, $CDCl_3$) δ 8.05 (dd, J = 8.0, 1.2 Hz, 1H), 7.88 (dd, J = 7.8, 1.7 Hz, 1H), 7.45 (td, J = 7.6, 1.2 Hz, 1H), 7.23 (td, J = 7.7, 1.7 Hz, 1H), 4.97 (s, 2H); ^{13}C NMR (125 MHz, $CDCl_3$) ppm 164.4, 141.9, 133.8, 132.2, 131.6, 128.1, 114.1, 94.7, 49.1; HRMS (EI): Exact mass calcd for $C_9H_6INO_2$ $[M]^+$ 286.9443, found 286.9448.

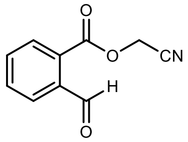

Cyanomethyl 2-formylbenzoate (14). Prepared according to the general procedure using 2-formylbenzoic acid (150 mg, 1.00 mmol), triethylamine (153 μL, 1.10 mmol), chloroacetonitrile (191 μL, 3.00 mmol) and dichloromethane (2.0 mL). The product was obtained as a clear oil (146 mg, 77%). ^1H NMR (500 MHz, $CDCl_3$) δ 10.58 (s, 1H), 7.99 (d, J = 7.5 Hz, 2H), 7.73 (m, 2H), 5.01 (s, 2H); ^{13}C NMR (125 MHz, $CDCl_3$) ppm 191.2, 164.7, 137.2, 133.5, 133.2, 130.5, 129.4, 124.7, 114.0, 49.3; HRMS (EI): Exact mass calcd for $C_{10}H_6NO_3$ $[M]^+$ 189.0348, found 189.0363.

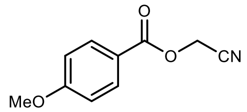

Cyanomethyl 4-methoxybenzoate (15). Prepared according to the general procedure using 4-methoxybenzoic acid (100 mg, 0.66 mmol), triethylamine (140 µL, 0.99 mmol), chloroacetonitrile (53 µL, 0.79 mmol) and dichloromethane (0.7 mL). The product was obtained as a white solid (102 mg, 81%). ^{1}H NMR (500 MHz, $CDCl_3$) δ 8.01 (d, J = 9.0 Hz, 2H), 6.95 (d, J = 8.9 Hz, 2H), 4.93 (s, 2H), 3.88 (s, 3H); ^{13}C NMR (125 MHz, $CDCl_3$) ppm 164.6, 164.3, 132.2, 120.1, 114.7, 114.0, 55.5, 48.6; HRMS (EI): Exact mass calcd for $C_{10}H_9NO_3$ [M]$^+$ 191.0582, found 191.0581.

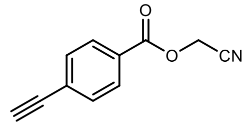

Cyanomethyl 4-ethynylbenzoate (16). Prepared according to the general procedure using 4-ethynylbenzoic acid (96 mg, 0.66 mmol), triethylamine (140 µL, 0.99 mmol), chloroacetonitrile (53 µL, 0.79 mmol) and dichloromethane (0.7 mL). The product was obtained as a white solid (87 mg, 76%). ^{1}H NMR (500 MHz, $CDCl_3$) δ 8.02 (d, J = 8.5 Hz, 2H), 7.59 (d, J = 8.4 Hz, 2H), 4.97 (s, 2H), 3.29 (s, 1H); ^{13}C NMR (125 MHz, $CDCl_3$) ppm 164.3, 132.4, 129.9, 128.1, 127.7, 114.3, 82.4, 81.0, 49.0; HRMS (EI): Exact mass calcd for $C_{11}H_7NO_2$ [M]$^+$ 185.0477, found 185.0476.

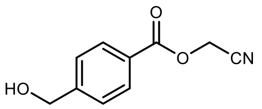

Cyanomethyl 4-(hydroxymethyl)benzoate (17). Prepared according to the general procedure using 4-(hydroxymethyl)benzoic acid (500 mg, 3.29 mmol), triethylamine (700 µL, 4.94 mmol), chloroacetonitrile (266 µL, 3.95 mmol) and dichloromethane (1.2 mL). The product was obtained as a white solid (470 mg, 75%). ^{1}H NMR (500 MHz, $CDCl_3$) δ 8.03 (d, J = 8.0 Hz, 1H), 7.47 (d, J = 7.9 Hz, 1H), 4.96 (s, 2H), 4.79 (s, 2H), 2.10 (br s, 1H); ^{13}C NMR (125 MHz, $CDCl_3$) ppm 164.8, 147.4, 130.3, 126.9, 126.6, 114.5, 64.4, 48.8; HRMS (ESI): Exact mass calcd for $C_{10}H_9NNaO_3$ [M+Na]$^+$ 214.0480, found 214.0486.

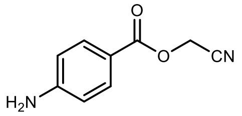

Cyanomethyl 4-aminobenzoate (18). Prepared according to the general procedure using 4-(Boc-amino)benzoic acid (78 mg, 0.33 mmol), triethylamine (70 µL, 0.5 mmol), chloroacetonitrile (26.5 µL, 0.4 mmol) in DMF (0.4 mL). The product was obtained as a white solid (39 mg, 68%) ^{1}H-NMR (500 MHz, DMSO-d_6) δ 7.66 (td, J = 8.7 Hz, 2H), 6.59 (td, J = 8.6 Hz, 2H), 6.18 (s, 2H), 5.08 (s, 2H); ^{13}C NMR (125 MHz, DMSO-d_6) ppm 165.1, 154.9, 132.2, 117.0, 113.9, 113.3, 49.3; Exact mass calcd for $C_9H_8N_2O_2$ [M]$^+$ 176.0586, found 176.0585.

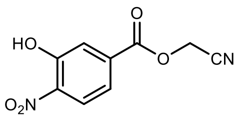

Cyanomethyl 3-hydroxy-4-nitrobenzoate (19). Prepared according to the general procedure using 3-hydroxy-4-nitrobenzoic acid (200 mg, 1.09 mmol), triethylamine (232 μL, 1.64 mmol), chloroacetonitrile (88 μL, 1.31 mmol) and dichloromethane (1.2 mL). The product was obtained as a yellow solid (92 mg, 38%). ^{1}H NMR (500 MHz, $CDCl_3$) δ 10.51 (s, 1H), 8.23 (d, *J* = 8.8 Hz, 1H), 7.87 (d, *J* = 1.9 Hz, 1H), 7.65 (dd, *J* = 8.8, 1.8 Hz, 1H), 5.00 (s, 2H); ^{13}C NMR (125 MHz, $CDCl_3$) ppm 162.9, 154.7, 136.4, 135.4, 125.7, 122.3, 120.8, 113.7, 49.5; HRMS (EI): Exact mass calcd for $C_9H_6N_2O_5$ $[M]^+$ 222.0276, found 222.0272.

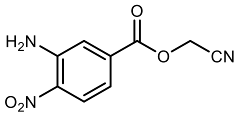

Cyanomethyl 3-amino-4-nitrobenzoate (20). Prepared according to the general procedure using 3-amino-4-nitrobenzoic acid (198 mg, 1.09 mmol), triethylamine (232 μL, 1.64 mmol), chloroacetonitrile (88 μL, 1.31 mmol) and dichloromethane (1.2 mL). The product was obtained as a yellow solid (210 mg, 87%). ^{1}H NMR (500 MHz, d_6-DMSO) δ 8.10 (dd, *J* = 9.0, 1.0 Hz, 1H), 7.74 (d, *J* = 1.9 Hz, 1H), 7.65 (s, 2H), 7.09 (dd, *J* = 8.9, 1.9 Hz, 1H), 5.24 (s, 2H); ^{13}C NMR (125 MHz, d_6-DMSO) ppm 163.7, 145.7, 133.6, 132.5, 126.5, 121.5, 115.9, 114.5, 50.4; HRMS (ESI): Exact mass calcd for $C_9H_7N_3NaO_4$ $[M+Na]^+$ 244.0334, found 244.0335.

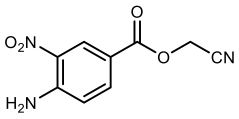

Cyanomethyl 4-amino-3-nitrobenzoate (21). Prepared according to the general procedure using 4-amino-3-nitrobenzoic acid (198 mg, 1.09 mmol), triethylamine (232 μL, 1.64 mmol), chloroacetonitrile (88 μL, 1.31 mmol) and dichloromethane (1.2 mL). The product was obtained as a yellow solid (120 mg, 49%). ^{1}H NMR (500 MHz, d_6-acetone) δ 8.74 (d, *J* = 1.9 Hz, 1H), 7.96 (dd, *J* = 8.9, 2.0 Hz, 1H), 7.68 (s, 2H), 7.19 (d, *J* = 9.0 Hz, 1H), 5.17 (s, 2H); ^{13}C NMR (125 MHz, d_6-acetone) ppm 164.3, 150.2, 136.0, 129.9, 120.3, 120.2, 116.3, 116.2, 49.9; HRMS (ESI): Exact mass calcd for $C_9H_7N_3NaO_4$ $[M+Na]^+$ 244.0334, found 244.0329.

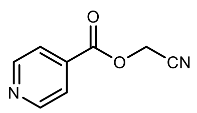

Cyanomethyl isonicotinate (22). Prepared according to the general procedure using isonicotinic acid (81 mg, 0.66 mmol), triethylamine (140 μL, 0.99 mmol), chloroacetonitrile (53 μL, 0.79 mmol) and dichloromethane (0.7 mL). The product was obtained as a red oil (50 mg, 47%). ^{1}H NMR (500 MHz, $CDCl_3$) δ 8.85 (d, *J* = 3.9 Hz, 2H), 7.87 (d, *J* = 6.1 Hz, 2H), 5.01 (s, 2H); ^{13}C NMR (125 MHz,

CDCl$_3$) ppm 163.7, 150.9, 135.0, 122.9, 113.8, 49.4; HRMS (EI): Exact mass calcd for $C_8H_6N_2O_4$ $[M]^+$ 162.0429, found 162.0430.

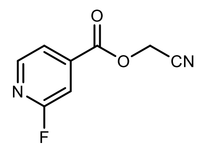

Cyanomethyl 2-fluoroisonicotinate (23). Prepared according to the general procedure using 2-fluoroisonicotinic acid (93 mg, 0.66 mmol), triethylamine (140 µL, 0.99 mmol), chloroacetonitrile (53 µL, 0.79 mmol) and dichloromethane (0.7 mL). The product was obtained as a white solid (102 mg, 86%). ^{1}H NMR (500 MHz, CDCl$_3$) δ 8.43 (d, *J* = 5.1 Hz, 1H), 7.77 (m, 1H), 7.52 (dd, *J* = 2.6, 1.2 Hz, 1H), 5.02 (s, 2H); ^{13}C NMR (125 MHz, CDCl$_3$) ppm 164.4 (d, $^1J_{CF}$ = 241.1 Hz), 162.7 (d, $^4J_{CF}$ = 4.5 Hz), 149.4 (d, $^3J_{CF}$ = 14.6 Hz), 140.6 (d, $^3J_{CF}$ = 7.8 Hz), 121.1 (d, $^4J_{CF}$ = 4.9 Hz), 113.8, 110.4 (d, $^2J_{CF}$ = 39.7 Hz), 49.9; HRMS (EI): Exact mass calcd for $C_8H_5FN_2O_2$ $[M]^+$ 180.0335, found 180.0332.

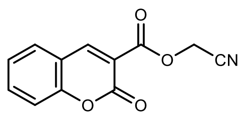

Cyanomethyl 2-oxo-2*H*-chromene-3-carboxylate (24). Prepared according to the general procedure using 2-oxo-2*H*-chromene-3-carboxylic acid (125 mg, 0.66 mmol), triethylamine (140 µL, 0.99 mmol), chloroacetonitrile (53 µL, 0.79 mmol) and dichloromethane (0.7 mL). The product was obtained as a white solid (118 mg, 78%). ^{1}H NMR (500 MHz, CDCl$_3$) δ 8.67 (s, 1H), 7.72 (dd, *J* = 8.0, 7.5 Hz, 1H), 7.67 (d, *J* = 7.2 Hz, 1H), 7.40 (d, *J* = 8.0 Hz, 1H), 7.39 (dd, J = 8.0, 7.5 Hz, 1H), 4.99 (s, 2H); ^{13}C NMR (125 MHz, CDCl$_3$) ppm 161.5, 156.0, 155.5, 150.9, 135.5, 130.0, 125.2, 117.5, 117.0, 115.7, 113.9, 49.3; HRMS (EI): Exact mass calcd for $C_{12}H_7NNO_4$ $[M]^+$ 229.0375, found 229.0382.

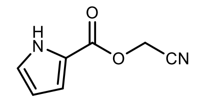

Cyanomethyl 1*H*-pyrrole-2-carboxylate (25). Prepared according to the general procedure using 1*H*-pyrrole-2-carboxylic acid (37 mg, 0.33 mmol), triethylamine (70 µL, 0.5 mmol), chloroacetonitrile (26.5 µL, 0.4 mmol) and dichloromethane (0.2 mL). The product was obtained as a white solid (24 mg, 49%). ^{1}H-NMR (500 MHz, DMSO-*d$_6$*) δ 12.15 (s, 1H), 7.13 (m, 1H), 6.91 (m, 1H), 6.23 (m, 1H), 5.12 (s, 2H); ^{13}C NMR (125 MHz, DMSO-*d$_6$*) ppm 159.4, 126.2, 120.3, 117.2, 116.7, 110.6, 49.2; ESI-MS; calculated mass for $C_7H_6N_2O_2$: $[M]^+$ 150.0429, found 150.0432.

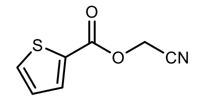

Cyanomethyl thiophene-2-carboxylate (26). Prepared according to the general procedure using thiophene-2-carboxylic acid (84 mg, 0.66 mmol), triethylamine (140 µL, 0.99 mmol), chloroacetonitrile (53 µL, 0.79 mmol) and dichloromethane (0.7 mL). The product was obtained as a brown oil (72 mg, 79%). ^{1}H NMR (500 MHz, $CDCl_3$) δ 7.89 (dd, *J* = 3.8, 1.3 Hz, 1H), 7.67 (dd, *J* = 5.0, 1.3 Hz, 1H), 7.15 (dd, *J* = 4.9, 3.8 Hz, 1H), 4.94 (s, 2H); ^{13}C NMR (125 MHz, $CDCl_3$) ppm 160.4, 135.2, 134.3, 130.7, 128.2, 114.2, 48.7; HRMS (EI): Exact mass calcd for $C_7H_5NO_2S$ $[M]^+$ 167.0041, found 167.0038.

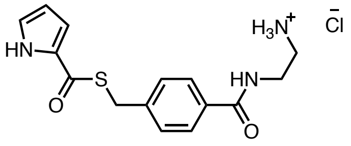

2-(4-(((1H-pyrrole-2-carbonyl)thio)methyl)benzamido)ethan-1-aminium chloride (25a). Prepared according to the general procedure using 1H-pyrrole-2-carboxylic acid (50 mg, 0.45 mmol), ABT (100 mg, 0.32 mmol), DMAP (109 mg, 0.9 mmol), EDC•HCl (171 mg, 0.9 mmol) and dichloromethane (2.0 mL). Flash column chromatography (SiO_2 30%-50% ethyl acetate in hexanes) yielded the Boc-protected product as a white solid (60 mg, 15%). Boc-deprotection with 4M HCl•dioxane provided the product, which was used without further purification and characterization. Boc-**25a**: ^{1}H NMR (500 MHz, $CDCl_3$) δ 9.26 (s, 1H), 7.77 (d, J = 7.9 Hz, 2H), 7.43 (d, J = 8.1 Hz, 2H), 7.14 (s, 1H), 7.03 (d, J = 11.4 Hz, 2H), 6.29 (d, J = 3.0 Hz, 1H), 4.97 (s, 1H), 4.31 (s, 2H), 3.57 (q, J = 5.1 Hz, 2H), 3.45 – 3.38 (m, 2H), 1.44 (s, 9H). ^{13}C NMR (125 MHz, $CDCl_3$) ppm 180.48, 167.37, 133.06, 129.71, 129.02, 127.32, 123.84, 115.37, 110.92, 42.09, 40.00, 31.91, 28.34. HRMS (ESI): Exact mass calcd for $C_{20}H_{26}N_3O_4S$ $[M+H]^+$ 404.1644, found 404.1632.

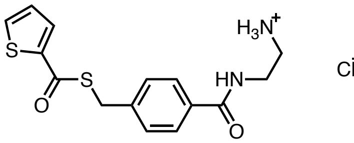

2-(4-(((thiophene-2-carbonyl)thio)methyl)benzamido)ethan-1-aminium chloride (26a). Prepared according to the general procedure using thiophene-2-carboxylic acid (57 mg, 0.45 mmol), ABT (100 mg, 0.32 mmol), DMAP (109 mg, 0.9 mmol), EDC•HCl (171 mg, 0.9 mmol) and dichloromethane (2.0 mL). Flash column chromatography (SiO_2 30%-50% ethyl acetate in hexanes) yielded the Boc-protected product as a white solid (150 mg, 76%). Boc-deprotection with 4M HCl•dioxane provided the product, which was used without further purification and characterization. Boc-**26a**: ^{1}H NMR (500 MHz, $CDCl_3$) δ 7.84 – 7.75 (m, 3H), 7.65 (dd, J = 4.9, 1.1 Hz, 1H), 7.44 (d, J = 8.1 Hz, 2H), 7.22 (br, 1H), 7.13 (dd, J = 4.9, 3.9 Hz, 1H), 5.00 (s, 1H), 4.35 (s, 2H), 3.56 (q, J = 5.1 Hz, 2H), 3.45 – 3.37 (m, 2H), 1.44 (s, 9H). ^{13}C NMR (125 MHz,

$CDCl_3$) ppm 182.92, 167.32, 157.50, 141.52, 141.06, 133.22, 132.98, 131.34, 129.11, 128.38, 128.32, 127.96, 127.39, 126.09, 42.12, 39.99, 32.99, 28.34. HRMS (ESI): Exact mass calcd for $C_{20}H_{25}N_2O_4S_2$ $[M+H]^+$ 421.1256, found 421.1249.

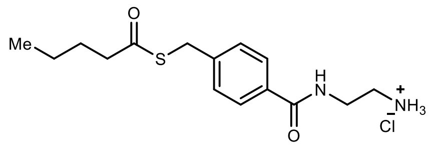

2-(4-((Pentanoylthio)methyl)benzamido)ethan-1-aminium chloride (G). Prepared according to the general procedure using valeric acid (47 µL, 0.43 mmol), ABT (93 mg, 0.30 mmol), DMAP (105 mg, 0.86 mmol), EDC•HCl (165 mg, 0.86 mmol) and dichloromethane (1.0 mL). Flash column chromatography (SiO_2 30%-50% ethyl acetate in hexanes) yielded the Boc-protected product as a white solid (66 mg, 56%). Boc-deprotection with 4M HCl•dioxane provided the product, which was used without further purification and characterization. Boc-**G**: ^1H NMR (500 MHz, $CDCl_3$) δ 7.73 (d, *J* = 7.9 Hz, 2H), 7.30 (d, *J* = 8.0 Hz, 2H), 7.28 (br s, 1H), 5.14 (br s, 1H), 4.11 (s, 2H), 3.52 (q, 5.3 Hz, 2H), 3.37 (m, 2H), 2.56 (t, *J* = 7.5 Hz, 2H), 1.63 (p, *J* = 7.5 Hz, 2H), 1.40 (s, 9H), 1.33 (p, *J* = 7.5 Hz, 2H), 0.89 (t, *J* = 7.4 Hz, 3H); ^{13}C NMR (125 MHz, $CDCl_3$) ppm 198.6, 167.4, 157.5 141.4, 133.0, 128.8, 127.3, 79.9, 43.5, 42.0, 39.9, 32.7, 28.3, 27.6, 22.0, 13.7; HRMS (ESI): Exact mass calcd for $C_{20}H_{31}N_2O_4S$ $[M+H]^+$ 395.2005, found 395.2009.

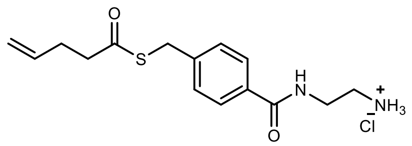

2-(4-((Pent-4-enoylthio)methyl)benzamido)ethan-1-aminium chloride (27). Prepared according to the general procedure using 4-pentenoic acid (44 µL, 0.43 mmol), ABT (93 mg, 0.30 mmol), DMAP (105 mg, 0.86 mmol), EDC•HCl (165 mg, 0.86 mmol) and dichloromethane (1.0 mL). Flash column chromatography (SiO_2 30%-50% ethyl acetate in hexanes) yielded the Boc-protected product as a white solid (61 mg, 52%). Boc-deprotection with 4M HCl•dioxane provided the product, which was used without further purification and characterization. Boc-**15**: ^1H NMR (500 MHz, $CDCl_3$) δ 7.73 (d, *J* = 8.0 Hz, 2H), 7.30 (d, *J* = 8.3 Hz, 2H), 7.29 (br s, 1H), 5.77 (ddt, *J* = 16.8, 10.2, 6.5 Hz, 1H), 5.16 (br s, 1H), 5.04 (dd, J = 17.1, 1.7 Hz, 1H), 4.99 (dd, J = 10.2, 5.1 Hz, 1H), 4.12 (s, 2H), 3.52 (q, 5.2 Hz, 2H), 3.37 (m, 2H), 2.65 (dd, *J* = 8.3, 6.7 Hz, 2H), 2.40 (tdd, *J* = 8.5, 5.9, 3.5 Hz, 2H), 1.40 (s, 9H); ^{13}C NMR (125 MHz, $CDCl_3$) ppm 197.8, 167.4, 157.5, 141.3, 135.9, 133.0, 128.8, 127.3, 115.9, 79.9, 42.8, 42.0, 39.9, 32.7, 29.3, 28.3; HRMS (ESI): Exact mass calcd for $C_{20}H_{29}N_2O_4S$ $[M+H]^+$ 393.1848, found 393.1850.

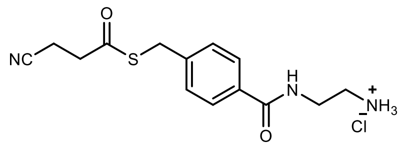

2-(4-(((3-Cyanopropanoyl)thio)methyl)benzamido)ethan-1-aminium chloride (28). Prepared according to the general procedure using 3-cyanopropanoic acid (43 mg, 0.43 mmol), ABT (93 mg, 0.30 mmol), DMAP (105 mg, 0.86 mmol), EDC•HCl (165 mg, 0.86 mmol) and dichloromethane (1.0 mL). Flash column chromatography (SiO_2 30%-50% ethyl acetate in hexanes) yielded the Boc-protected product as a white solid (42 mg, 36%). Boc-deprotection with 4M HCl•dioxane provided the product, which was used without further purification and characterization. Boc-**16**: ^{1}H NMR (500 MHz, $CDCl_3$) δ 7.75 (d, *J* = 7.9 Hz, 2H), 7.32 (d, *J* = 8.2 Hz, 2H), 7.27 (br s, 1H), 5.07 (br s, 1H), 4.18 (s, 2H), 3.53 (q, 5.1 Hz, 2H), 3.38 (q, *J* = 5.8 Hz, 2H), 2.94 (dd, *J* = 7.7, 6.7 Hz, 2H), 2.68 (dd, *J* = 7.7, 6.7 Hz, 2H), 1.42 (s, 9H); ^{13}C NMR (125 MHz, $CDCl_3$) ppm 194.5, 167.2, 157.5, 140.3, 133.4, 128.9, 127.4, 118.0, 80.0, 42.1, 39.9, 38.3, 33.0, 28.3, 12.8; HRMS (ESI): Exact mass calcd for $C_{19}H_{26}N_3O_4S$ $[M+H]^+$ 392.1644, found 392.1658.

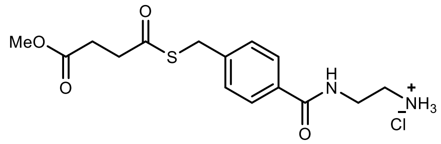

2-(4-(((4-Methoxy-4-oxobutanoyl)thio)methyl)benzamido) ethan-1-aminium chloride (29). Prepared according to the general procedure using monomethyl succinic acid (57 mg, 0.43 mmol), ABT (93 mg, 0.30 mmol), DMAP (105 mg, 0.86 mmol), EDC•HCl (165 mg, 0.86 mmol) and dichloromethane (1.0 mL). Flash column chromatography (SiO_2 30%-50% ethyl acetate in hexanes) yielded the Boc-protected product as a white solid (57 mg, 45%). Boc-deprotection with 4M HCl•dioxane provided the product, which was used without further purification and characterization. Boc-**17**: ^{1}H NMR (500 MHz, $CDCl_3$) δ 7.73 (d, *J* = 7.9 Hz, 2H), 7.30 (d, *J* = 8.0 Hz, 2H), 7.29 (br s, 1H), 5.14 (br s, 1H), 4.13 (s, 2H), 3.67 (s, 3H), 3.51 (q, 5.3 Hz, 2H), 3.37 (m, 2H), 2.89 (t, *J* = 6.9 Hz, 2H), 2.66 (t, *J* = 6.9 Hz, 2H), 1.41 (s, 9H); ^{13}C NMR (125 MHz, $CDCl_3$) ppm 196.8, 172.3, 167.3, 157.5, 141.0, 133.1, 128.9, 127.3, 79.9, 51.9, 42.0, 39.9, 38.1, 32.8, 28.9, 28.3; HRMS (ESI): Exact mass calcd for $C_{20}H_{29}N_2O_6S$ $[M+H]^+$ 425.1746, found 425.1759.

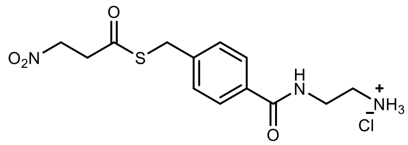

2-(4-(((3-Nitropropanoyl)thio)methyl)benzamido)ethan-1-aminium chloride (30). Prepared according to the general procedure using 3-nitropropionic acid (51 mg, 0.43 mmol), ABT (93 mg, 0.30 mmol), DMAP (105 mg, 0.86 mmol), EDC•HCl (165 mg, 0.86 mmol) and dichloromethane (1.0 mL). Flash column chromatography (SiO_2 30%-50%

ethyl acetate in hexanes) yielded the Boc-protected product as a white solid (57 mg, 46%). Boc-deprotection with 4M HCl•dioxane provided the product, which was used without further purification and characterization. Boc-**13**: ^{1}H NMR (500 MHz, $CDCl_3$) δ 7.76 (d, *J* = 8.0 Hz, 2H), 7.33 (d, *J* = 8.2 Hz, 2H), 7.19 (br s, 1H), 4.97 (br s, 1H), 4.70 (t, *J* = 6.2 Hz, 2H), 4.19 (s, 2H), 3.54 (q, 5.2 Hz, 2H), 3.40 (m, 2H), 3.25 (t, *J* = 6.2 Hz, 2H), 1.43 (s, 9H); ^{13}C NMR (125 MHz, $CDCl_3$) ppm 194.0, 167.2, 157.6, 140.3, 133.4, 129.0, 127.4 80.1, 69.3, 42.2, 39.9, 39.3, 33.0, 28.3; HRMS (ESI): Exact mass calcd for $C_{18}H_{26}N_3O_6S$ $[M+H]^+$ 244.0334, found 412.1531.

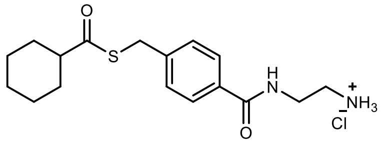

2-(4-(((Cyclohexanecarbonyl)thio)methyl)benzamido)ethan-1-aminium chloride (31). Prepared according to the general procedure using cyclohexanecarboxylic acid (53 µL, 0.43 mmol), ABT (93 mg, 0.30 mmol), DMAP (105 mg, 0.86 mmol), EDC•HCl (165 mg, 0.86 mmol) and dichloromethane (1.0 mL). Flash column chromatography (SiO_2 30%-50% ethyl acetate in hexanes) yielded the Boc-protected product as a white solid (77 mg, 61%). Boc-deprotection with 4M HCl•dioxane provided the product, which was used without further purification and characterization. Boc-**12**: ^{1}H NMR (500 MHz, $CDCl_3$) δ 7.72 (d, *J* = 8.1 Hz, 2H), 7.30 (d, *J* = 8.2 Hz, 2H), 7.29 (br s, 1H), 5.15 (br s, 1H), 4.08 (s, 2H), 3.52 (q, 5.2 Hz, 2H), 3.37 (m, 2H), 2.48 (tt, *J* = 11.5, 3.6 Hz, 1H), 1.90 (dd, *J* = 12.9, 3.3 Hz, 2H), 1.76 (dt, *J* = 12.7, 3.4 Hz, 2H), 1.69-1.57 (m, 1H), 1.45 (qd, *J* = 12.0, 3.1 Hz, 2H), 1.40 (s, 9H), 1.31-1.12 (m, 3H); ^{13}C NMR (125 MHz, $CDCl_3$) ppm 202.0, 167.4, 157.4, 141.6, 132.9, 128.8, 127.3, 79.9, 52.7, 41.9, 39.9, 32.3, 29.5, 28.3, 25.5, 25.4; HRMS (ESI): Exact mass calcd for $C_{22}H_{33}N_2O_4S$ $[M+H]^+$ 421.2161, found 421.2151.

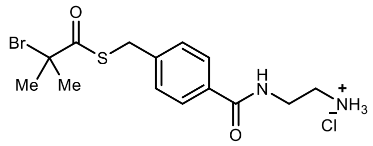

2-(4-(((2-Bromo-2-methylpropanoyl)thio)methyl)benzamido) ethan-1-aminium chloride (32). Prepared according to the general procedure using α-bromoisobutyric acid (72 mg, 0.43 mmol), ABT (93 mg, 0.30 mmol), DMAP (105 mg, 0.86 mmol), EDC•HCl (165 mg, 0.86 mmol) and dichloromethane (1.0 mL). Flash column chromatography (SiO_2 30%-50% ethyl acetate in hexanes) yielded the Boc-protected product as a white solid (93 mg, 68%). Boc-deprotection with 4M HCl•dioxane provided the product, which was used without further purification and characterization. Boc-**14**: ^{1}H NMR (500 MHz, $CDCl_3$) δ 7.74 (d, *J* = 8.0 Hz, 2H), 7.33 (d, *J* = 8.4 Hz, 2H), 7.29 (br s, 1H), 5.16 (br s, 1H), 4.12 (s, 2H), 3.52 (q, 5.3 Hz, 2H), 3.38 (m, 2H), 1.93 (s, 6H), 1.40 (s, 9H); ^{13}C NMR (125 MHz, $CDCl_3$) ppm 199.1, 167.4, 157.5,

140.4, 133.2, 128.9, 127.4, 79.9, 63.9, 42.0, 39.9, 34.2, 31.3 28.3; HRMS (ESI): Exact mass calcd for $C_{19}H_{28}BrN_2O_4S$ $[M+H]^+$ 459.0953, found 459.0964.

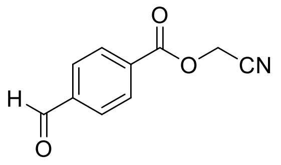

Cyanomethyl 4-formylbenzoate (33). Prepared according to the general procedure using 2-(4-formylphenyl)benzoic acid (150 mg, 1.00 mmol), triethylamine (200 µL, 1.5 mmol), chloroacetonitrile (5000 µL, 75.0 mmol) and dichloromethane (5 mL). The product was obtained as a white powder (168.4 mg, 89%). ^{1}H-NMR (500 MHz, DMSO-d_6) δ 10.14 (s, 1H), 8.20 (d, 2H, J = 8.6 Hz), 8.09 (d, 2H, J = 8.9 Hz), 5.2t (s, 2H); ^{13}C NMR (125 MHz, DMSO-d_6) ppm 193.4, 164.5, 140.1, 133.0, 130.7 (2C), 130.2 (2C), 116.3, 50.7; ESI-MS calculated mass for $C_{10}H_7NO_3$ $[M+H]^+$ 190.0426, found 190.0428.

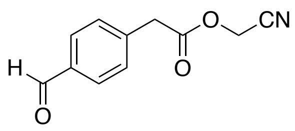

Cyanomethyl 2-(4-formylphenyl)acetate (34). Prepared according to the general procedure using 2-(4-formylphenyl)acetic acid (82 mg, 0.50 mmol), triethylamine (105 µL, 0.75 mmol), chloroacetonitrile (40 µL, 0.6 mmol) and dichloromethane (0.5 mL). The product was obtained as a white oil (52 mg, 52%). ^{1}H-NMR (500 MHz, $CDCl_3$) δ 9.8 (s, 1H), 7.66 (d, 2H, J = 4.8), 7.24 (d, 2H, J = 3.5), 4.54 (s, 2H), 3.60 (s, 2H); ^{13}C NMR (125 MHz, $CDCl_3$) ppm 191.6, 169.0, 138.9, 135.7, 130.1 (2C), 130.0 (2C), 113.9, 48.8, 40.4; ESI-MS calculated mass for $C_{11}H_9NO_3$ $[M-H]^-$ 202.0582, found 202.5122.

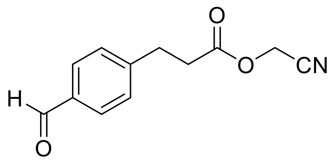

Cyanomethyl 3-(4-formylphenyl)propanoate (35). Prepared according to the general procedure using 3-(4-formylphenyl)propanoic acid (178.2 mg, 1.00 mmol), triethylamine (200 µL, 1.5 mmol), chloroacetonitrile (5000 µL, 75.0 mmol) and dichloromethane (5 mL). The product was obtained as a white powder (167.1 mg, 77%). ^{1}H-NMR (500 MHz, $CDCl_3$) δ 9.97 (s, 1H), 7.84 (d, 2H, J = 8.4 Hz), 7.49 (d, 2H, J = 7.9 Hz), 4.96 (s, 1H), 2.98 (t, 2H, J = 7.0 Hz), 2.83 (t, 2H, J = 6.9 Hz); ^{13}C NMR (125 MHz, $CDCl_3$) ppm 193.1, 171.6, 147.9, 135.0 130.1 (2C), 129.6 (2C), 116.4, 49.4, 34.0, 30.4; ESI-MS calculated mass for $C_{10}H_7NO_3$ $[M+H]^+$ 218.2240, found 218.2242.

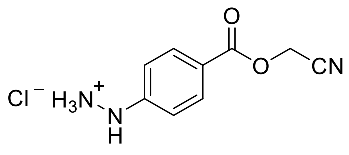

Cyanomethyl 4-hydrazineylbenzoate (36). Prepared according to the general procedure using 4-(2-(tert-butoxycarbonyl)hydrazineyl)benzoic acid (252.27 mg, 1.00 mmol),

triethylamine (200 µL, 1.5 mmol), chloroacetonitrile (5000 µL, 75.0 mmol) and dichloromethane (5 mL). The product was obtained as a yellow powder (146.1 mg, 76%). ^{1}H-NMR (500 MHz, DMSO-d_6) δ 7.90 (d, 2H, J = 9.3 Hz), 6.96 (d, 2H, J = 9.0 Hz), 5.16 (s, 2H), 4.59 (s, 2H); ^{13}C NMR (125 MHz, DMSO-d_6) ppm 164.8, 151.9, 131.6 (2C), 119.1, 116.7, 112.9 (2C), 49.7; ESI-MS; calculated mass for $C_9H_9N_3O_2$ [M+H]$^+$ 192.1900, found 192.0775

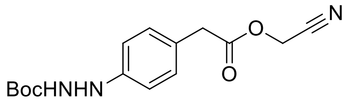

***tert*-Butyl 2-(4-(2-(cyanomethoxy)-2-oxoethyl)phenyl)hydrazinecarboxylate (37).** To a solution of 2-(4-(2-(*tert*-Butoxycarbonyl)hydrazinyl)phenyl)acetic acid (0.31 g, 1.16 mmol) in DMF (1.5 mL) were added chloroacetonitrile (1.5 mL) and triethylamine (0.17 mL, 1.22 mol). The solution was stirred at room temperature for 16 h, diluted with brine (100 mL), and then extracted with ethyl acetate (2 x 30 mL). The combined organic fractions were washed with brine, dried over $MgSO_4$, filtered, and evaporated under vacuum. The crude product was purified by silica gel chromatography (EA:Hex = 2:3) to give the product (0.25 g, 71%) as a soft colorless solid. NMR (400 MHz, $CDCl_3$) δ 7.04 (d, J = 8.4 Hz, 2H), 6.69 (d, J = 8.4 Hz, 2H), 4.58 (s, 2H), 3.55 (s, 2H), 1.43 (s, 9H); ^{13}C NMR (100 MHz, $CDCl_3$) δ 170.5, 156.4, 148.0, 130.0, 124.2, 114.6, 113.1, 81.1, 48.6, 39.6, 28.3; MS (ESI-TOF) calcd for $C_{15}H_{19}N_3O_4$ 305.1376, found 328.1268 [M + Na]$^+$.

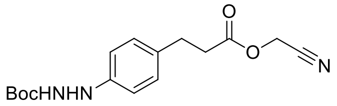

***tert*-Butyl 2-(4-(3-(cyanomethoxy)-3-oxopropyl)phenyl)hydrazinecarboxylate (38).** To a solution of 3-(4-(2-(*tert*-Butoxycarbonyl)hydrazinyl)phenyl)propanoic acid (0.40 g, 1.43 mmol) in DMF (1.5 mL) were added chloroacetonitrile (1.5 mL) and triethylamine (0.20 mL, 1.43 mol). The solution was stirred at room temperature for 16 h, diluted with brine (100 mL), and then extracted with ethyl acetate (2 x 30 mL). The combined organic fractions were washed with brine, dried over $MgSO_4$, filtered, and evaporated under vacuum. The crude product was purified by silica gel chromatography (EA:Hex = 1:2) to give the product (0.35 g, 77%) as a pale yellow oil. NMR (400 MHz, $CDCl_3$) δ 6.99 (d, J = 8.8 Hz, 2H), 6.69 (d, J = 8.4 Hz, 2H), 4.61 (s, 2H), 2.84 (t, J = 7.6 Hz, 2H), 2.62 (t, J = 7.6 Hz, 2H), 1.43 (s, 9H); ^{13}C NMR (100 MHz, $CDCl_3$) δ 171.4, 156.4, 147.1, 131.7, 129.0, 114.6, 113.2, 81.1, 48.2, 35.4, 29.8, 28.3; MS (ESI-TOF) calcd for $C_{16}H_{21}N_3O_4$ 319.1532, found 342.1427 [M+Na]$^+$.

Preparation of DNA templates for RNAs

The DNA templates were synthesized by using the following primers as previously described[4].

1) Extension (Generation of Fx derivatives by extending different 3'-ends)

A. Flexizymes

0.5 μL of 200 μM Fx_F primer and 0.5 μL of 200 μM of Fx_R1 primer (eFx_R1, dFx_R1, and aFx_R1 were used for eFx, dFx, and aFx generation, respectively) were added to 99 μL of a master mix containing 9.9 μL of 10X PCR buffer (500 mM KCl, 100 mM Tris-HCL (pH 9.0), and 1 % of Triton X-100), 0.99 μL of 250 mM $MgCl_2$, 4.95 μL of 5 mM dNTPs, 0.66 μL of Taq DNA polymerase (NEB), and 82.5 μL of water in a PCR tube. The thermocycling conditions were: 1 min at 95 °C followed by 5 cycles of 50 °C for 1 min and 72 °C for 1 min. The sizes of products were checked in 3 % (w/v) agarose gel.

2) PCR amplification

A. Flexizyme

5 μL of of the extension product was used as a PCR template. 200 μL of 5X OneTaq® Standard buffer, 20 μL of 10 mM dNTP, 5 μL of 200 μM Fx_T7F primer and 5 μL of 200 μM Fx_R2 (eFx_R2, dFx_R2, and aFx_R2 were used for eFx, dFx, and aFx generation, respectively), 10 μL of OneTaq® polymerase and 755 μL of nuclease-free water was mixed in a 1.5 mL microcentrifuge tube. The mixture was transferred to 10 PCR tubes and the DNA was amplified by the following thermocycling conditions: 1 min at 95 °C followed by 12 cycles of 95 °C for 40 s and 50 °C for 40 s, and 72 °C for 40 s. Products were checked in 3 % (w/v) agarose gel.

- Sequence of the final DNA templates produced by the PCR reactions

eFx	5'-GTAATACGACTCACTATAGGATCGAAAGATTTCCGCGGCCCCGAAAGGGGATTAGCGTTAGGT-3'
dFx	5'-GTAATACGACTCACTATAGGATCGAAAGATTTCCGCATCCCCGAAAGGGTACATGGCGTTAGGT-3'
aFx	5'-GTAATACGACTCACTATAGGATCGAAAGATTTCCGCACCCCCGAAAGGGGTAAGTGGCGTTAGGT-3'

B. tRNA

The DNA template for tRNA preparation was directly amplified from the full-length oligo by a pair of the primers corresponding to both 5'- and 3'-ends of the template. 5 μL of the DNA template (100 μM) for tRNA was mixed with 5 μL of 200 μM GluE2_fwd and Glu_E2_rev, 200 μL of 5X HF buffer, 10 μL of Phusion polymerase (NEB), 20 μL of 10 mM dNTPs, and 755 μL of water. The

thermocycling conditions were: 1 min at 95 °C followed by 35 cycles of 95 °C for 5 sec, 60 °C for 10 sec, and 72 °C 10 sec, and final elongation at 72 °C for 1 min. The sizes of products were checked in 3 % (w/v) agarose gel.

- Sequence of the final DNA templates produced by the PCR reactions

fMet_CAU	GTAATACGACTCACTATAGGCGGGGTGGAGCAGCCTGGTAGCTCGTCGGGCTCATAACCCGAAGATCGTCGGTTCAAATCCGGCCCCCGCAACCA

3) DNA precipitation

PCR products were combined, extracted using phenol/chloroform/isoamyl alcohol and precipitated and washed with EtOH. Sample were dried at room temperature for 5 min and resuspended in 100 µL nuclease-free water. DNA concentrations were determined spectrophotometrically (Thermo Scientific NanoDrop 2000C spectrophotometer).

In-vitro **transcription**

The microhelix was obtained from Integrated DNA Technologies (IDT) and directly used. Flexizymes and tRNAs were prepared using a HiScribe T7 high yield RNA synthesis kit (NEB). For *in vitro* transcription, 5 µg of DNA template was used with 10 µL of each of 10X T7 Reaction Buffer, ATP, CTP, GTP, UTP, T7 RNA polymerase mix, and nuclease-free water upto 100 µL. The mixture was incubated at 37 °C overnight.

Digestion of DNA templates

The DNA templates were removed by adding 5 µL of DNase I (NEB) and 20 µL of DNase I reaction buffer into the 100 µL of transcription reaction products. The reaction mixture was incubated for 1 h at 37 °C.

Purification of *in-vitro* transcribed RNA

The digested transcription reactions were mixed with 100 µL 2x RNA loading dye[4] , and loaded onto a 15 % TBE-Urea gel (Invitrogen). The gel was run in Tris-Borate-EDTA (89 mM Tris, 89 mM boric acid, 2 mM EDTA, and pH 8.3) buffer at 160 V for 2.5 h at room temperature. The gel was placed on a cling film covering a 20 cm x 20 cm TLC silica gel glass plate (EMD Millipore) coated with a fluorescent indicator and the transcribed RNAs were visualized by irradiating with UV lamp (260 nm). A sheet of cling film was covered on the gel and the band with desired size was marked on the film. The RNA products were excised from the gel and added to 2 mL of water. The gels

were crushed and then shaken in the cold room for 4 h. The gels were transferred to a centrifugal filter (EMD Millipore) and centrifuged at 4,000 g for 2 min. The flow-through was collected and added to the solution of 120 μL of 5 M NaCl and 5 mL of 100% EtOH and. The solution was placed in -20 °C for 16 h and centrifuged at 15,000 g for 45 min at 4 °C. The supernatant was removed and the pellet was dried for 5 min at room temperature. The dried RNA pellet was dissolved in nuclease-free water and the concentration was determined from the absorbance measured on a Thermo Scientific NanoDrop 2000C spectrophotometer.

Precipitation of tRNA

Into a 1.5 mL of microcentrifuge tube containing 100 μL of EtOH and 40 μL of 0.3 M NaOAc (pH 5.2), the mixture from coupling reaction was added and mixed to quench the reaction. The mixture was centrifuged at 21,000 g for 15 min at room temperature and the supernatant was removed. The RNA pellet was washed with 50 μL of 70 % (v/v) ethanol containing 0.1 M NaOAc (pH 5.2) was resuspended into the solution by vortexing and subsequently centrifuged at 21,000 g for 5 min at room temperature. The washing step was repeated twice. After the supernatant was discarded, the pellet was resuspended in 50 μL of 70 % (v/v) ethanol resuspended and centrifuged at 21,000 g for 3 min at room temperature. The supernatant was removed and the pellet was dissolved by 1 μL of 1 mM NaOAc (pH 5.2).

Peptide purification

The peptides produced in the PURExpress were produced by using an affinity tag purification technique. 2 μL of MagStrep (type3) XT beads 5 % suspension (iba) was washed twice with 200 and 100 μL of Strep-Tactin XT Wash buffer (1X) in a 1.5 mL microcentrifuge tube. The buffer was discarded by placing the tube on a magnetic rack. 10 μL of PURExpress reaction material was mixed with the wet magnetic beads and the tube containing the mixture was placed on ice for 30 min. The mixture was vortexed for 5 sec every 10 min. The tube was placed back on a magnetic rack and the supernatant was removed. The beads were washed twice with 200 and 100 μL of the wash buffer and the buffer was discarded. The beads were mixed with 10 μL of 0.1 % SDS solution (v/v in water) and transferred to a PCR tube and heated at 95° C for 2 min. The SDS solution was separated from the beads on a 96-well magnetic rack and further analyzed by mass spectrum.

For calculation of peptide (NH_2-WSHPQFEKST-OH) yield, the his-tagged enzymes resent in the PURExpress were removed using Ni-NTA-coated magnetic beads (His-Select® Nickel magnetic

agarose beads, Sigma). 2 µL of beads suspension (iba) was washed twice with 200 and 100 µL of Strep-Tactin XT Wash buffer (1X) in a 1.5 mL microcentrifuge tube. The reaction mixture was added to the beads and vortexed for 10 min at room temperature. The beads were washed on a magnetic rack and the supernatant was collected. The supernatant was added to a C18 spin column (Pierce C18 columns, Thermo Fisher Scientific) to remove residual nucleic acids and buffers. The column was washed twice with 20 % MeCN/water (5 % TFA) solution. The peptide was eluted using 80 % MeCN/water (5 % TFA) solution.

Characterization of peptides

1.5 µL of the peptides purified by the strep affinity tag was mixed on a MALDI plate with 1 µL of saturated α-cyano-4-hydroxycinnamic acid (CHCA) in THF containing 0.1 % TFA. The samples were dried at room temperature for 30 min. MALDI-TOF mass spectra of the peptides were obtained on a Bruker Autoflex III using the positive reflectron mode.

Primers

Fx_F	5′-GTAATACGACTCACTATAGGATCGAAAGATTTCCGC-3′
eFx_R1	5′-ACCTAACGCTAATCCCCTTTCGGGGCCGCGGAAATCTTTCGATCC-3′
dFx_R1	5′-ACCTAACGCCATGTACCCTTTCGGGGATGCGGAAATCTTTCGATCC-3′
aFx_R1	5′-ACCTAACGCCACTTACCCCTTTCGGGGGTGCGGAAATCTTTCGATCC-3′
Fx_T7F	5′-GGCGTAATACGACTCACTATAG-3′
eFx_R2	5′-ACCTAACGCTAATCCCCT-3′
dFx_R2	5′-ACCTAACGCCATGTACCCT-3′
aFx_R2	5′-ACCTAACGCCACTTACCCC-3′
microhelix	5′-rGrGrCrUrCrUrGrUrUrCrGrCrArGrArGrCrCrGrCrCrA-3′

Supplementary Tables and Figures

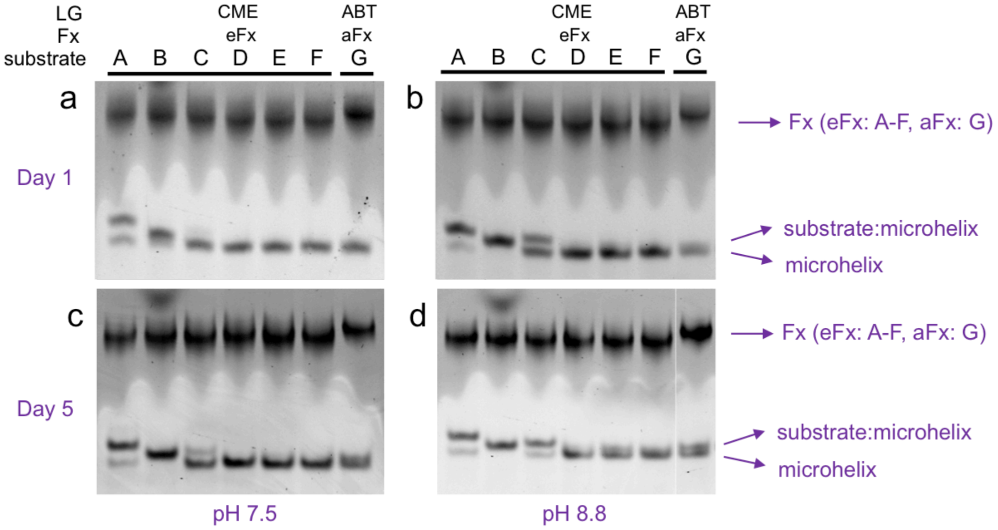

Supplementary Figure 1. Acylation of microhelix with substrates A-G. The Fx-catalyzed acylation reaction using the six representative substrates (Phe-CME (**A**), hcinA-CME (**B**), cinA-CME (**C**), benA-CME (**D**), PhAA-CME (**E**), penA-CME (**F**), penA-ABT (**G**) were monitored at two different pH (7.5 and 8.8) over 120 h. In general, high pH (pH 8.8) and long incubation time (120 h) gives high reaction yield. A part of **Fig. S1a** (lane A-C), **1b** (lane A-C), and **1d** (lane C-G) was used to produce **Fig. 2b**. LG: leaving group, Fx: Flexizyme, CME: cyanomethylester, ABT: (2-aminoethyl)amidocarboxybenzyl thioester.

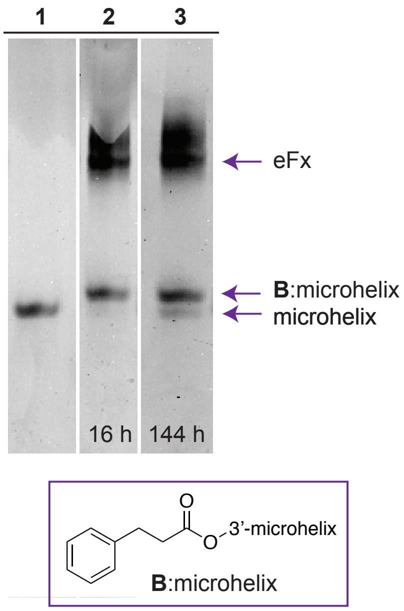

Supplementary Figure 2. Undesired hydrolysis of acylated microhelix. The microhelix charged by hcinA (**B**) was acylated at 16 h in a 100 % yield, however, the acylation yield was found to decrease (76 %) at 144 h, presumably because of unwanted hydrolysis by water on the ester linkage. Lane 1: microhelix; lane 2 and 3: crude acylated product observed at 16 h and 144 h, respectively. We limited the reaction time to 120 h based on this observation.

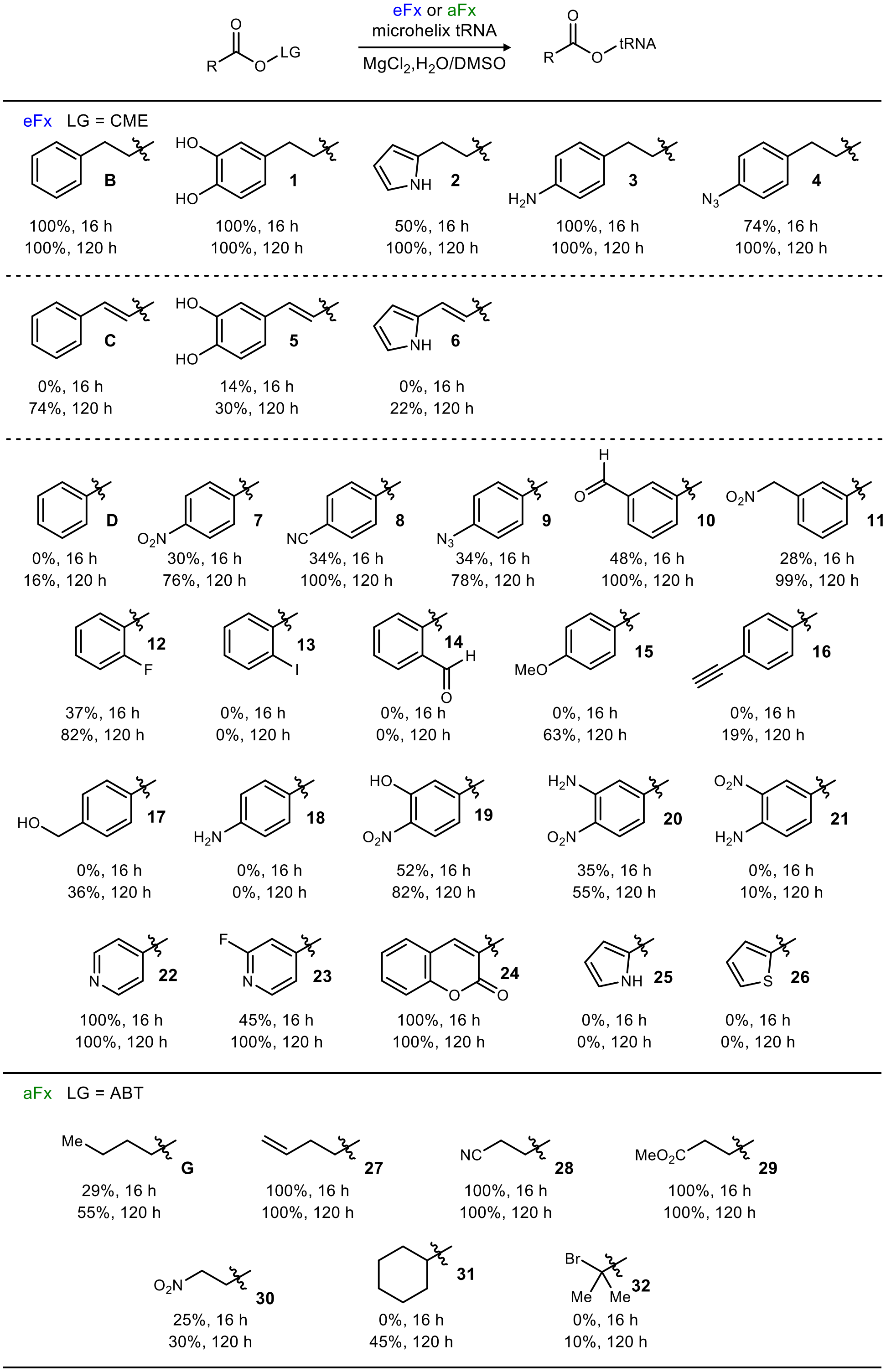

Supplementary Figure 3. Numerical acylation yields of microhelix obtained using the expanded substrates. The acylation reaction yields of microhelix with the 32 noncanonical chemical substrates were determined by quantifying the band intensity on the 20 % polyacrylamide gel (pH 5.2, 50 mM NaOAc, **Fig. S4-S6**). The quantified yields of acylated microhelix were used to produce the blue and green color code gradient and used in **Fig. 2c**.

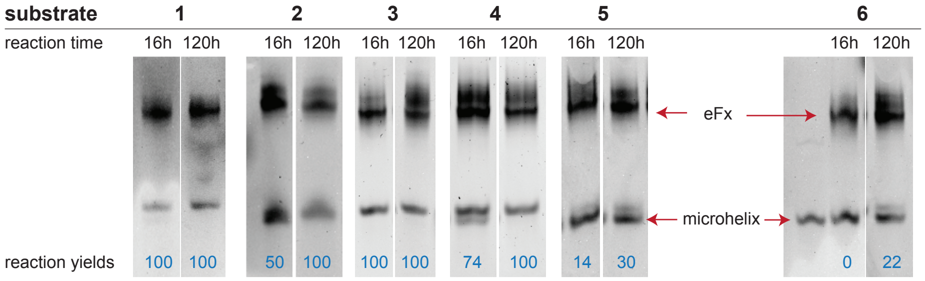

Supplementary Figure 4. Analysis of acylation with 1-6. The acylation yields were analyzed by electrophoresis on 20 % polyacrylamide gel containing 50 mM NaOAc (pH 5.2). The crude products containing the chemical substrates (**1**-**6**) were loaded on the gel and separated by the electrophoretic mobility at 135 mV in cold room over 2-3 h. The reactions were monitored over 120 h and the yields were quantified using densiometric analysis (software: ImageJ).

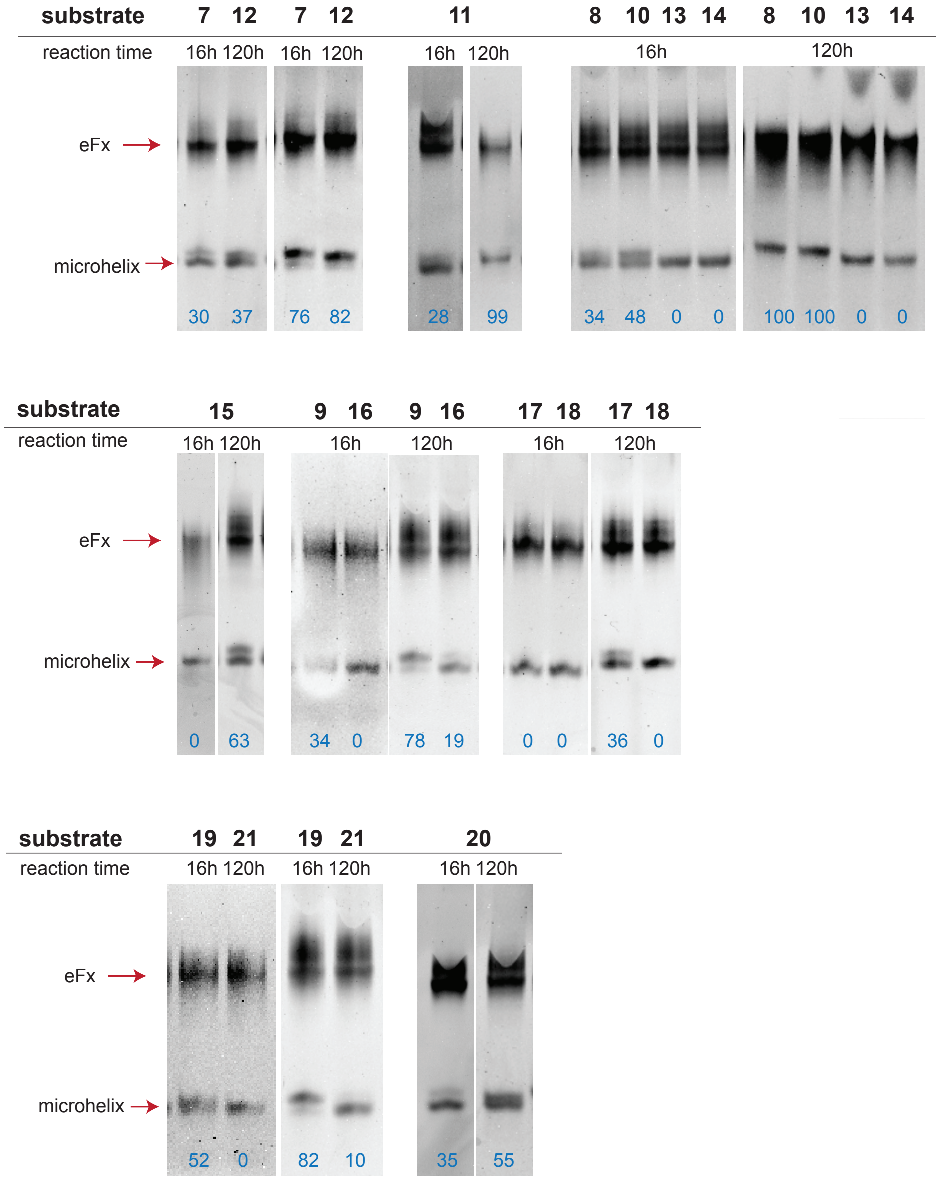

Supplementary Figure 5. Analysis of acylation with 7-21. The crude acylation reaction mixtures charged with the substrates (**7**-**21**) were analyzed by using the same methods described in the legend of **Fig. S3**.

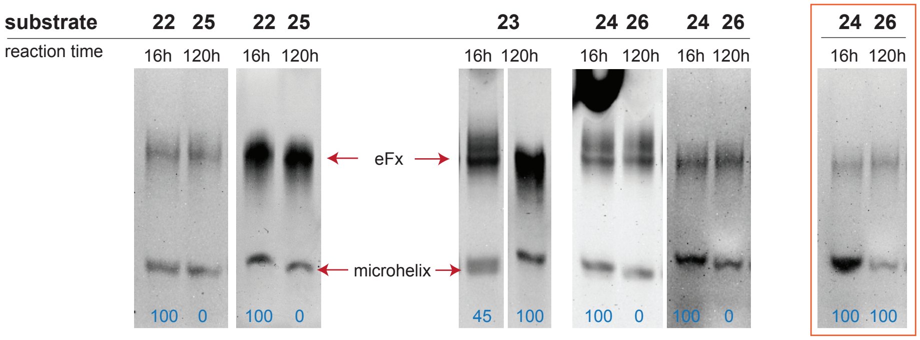

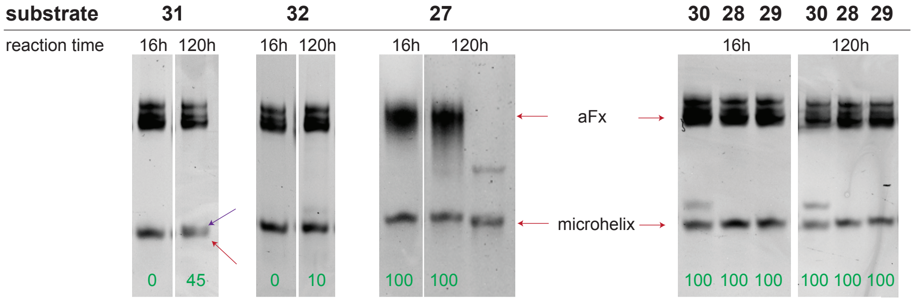

Supplementary Figure 6. Analysis of acylation with 22-32. The crude products charged with the chemical substrates (**22**-**32**) were analyzed. Gels were visualized by staining with GelRed (Biotium) and exposing on a filter of 630 nm for 20 s on a Gel Doc XR+ (Bio-Rad). The band containing the mihx charged with coumarin (**24**) in the orange box shows relatively higher intensity than the other nucleic acid bands when the gel is exposed in lower wavelength (560 nm). Note that the yields in blue and green were obtained from the reaction with the substrate containing an CME and ABT leaving group, respectively. (coumarin excitation/emission wavelength: 380 nm/410-470 nm)

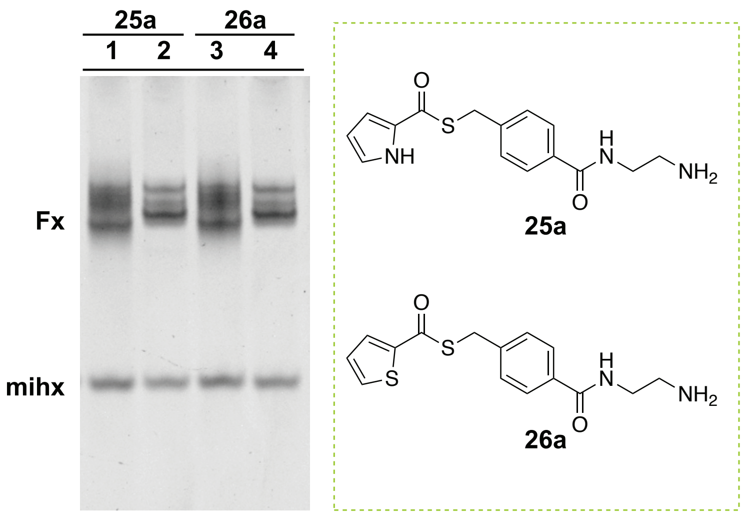

Supplementary Figure 7. Acylation test of pyrrole-ABT and thiophene-ABT. We tested additional substrates for the pyrrole and thiophene substrates (**25a** and **26a** with ABT) in case that eFx did not recognize the small aromatic ring. However, we were not able to find a new band for substrate-charged microhelix in the gel. eFx and aFx was used for lane 1, 3 and 2, 4, respectively. See the characterization section and SI_II for mass and NMR spectroscopic data.

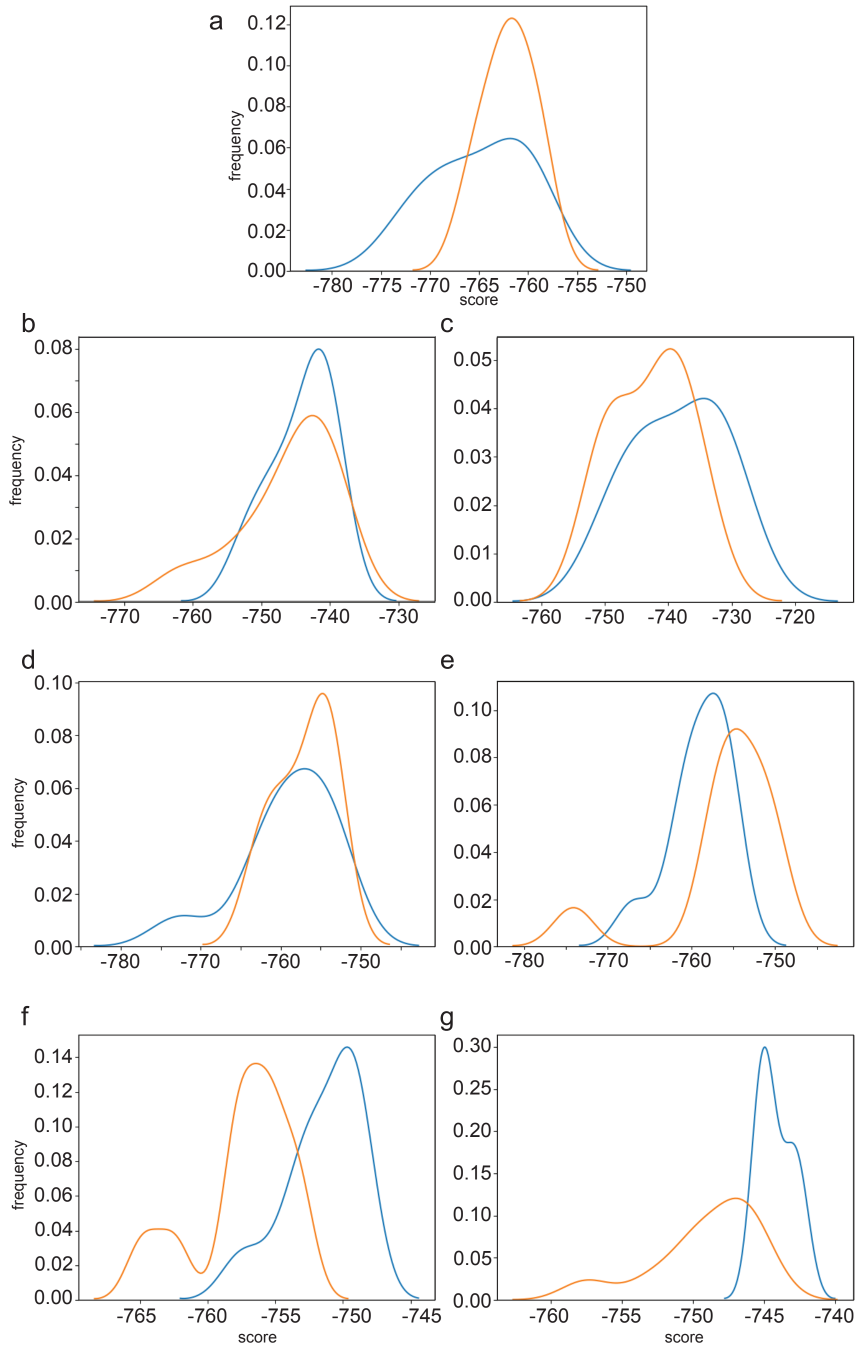

Supplementary Figure 8. Results of computational modeling. Constrained (blue) vs. unconstrained (orange) score distribution for the top 1% of models simulated for each of the same selection of moieties, including successfully incorporated residues like phenylalanine (**a**), 3-phenylpropanoic acid (**b**), hydrocinnamic acid (**c**), benzoic acid (**d**), and phenylacetic acid (**e**). The distributions are most shifted to favor the unconstrained state for the pyrrole-2-carboxylic acid (**f**) and 2-thiophenecarboxylic acid substrates (**g**). Scripts for conducting modeling simulations are available at https://github.com/everyday847/flexizyme.

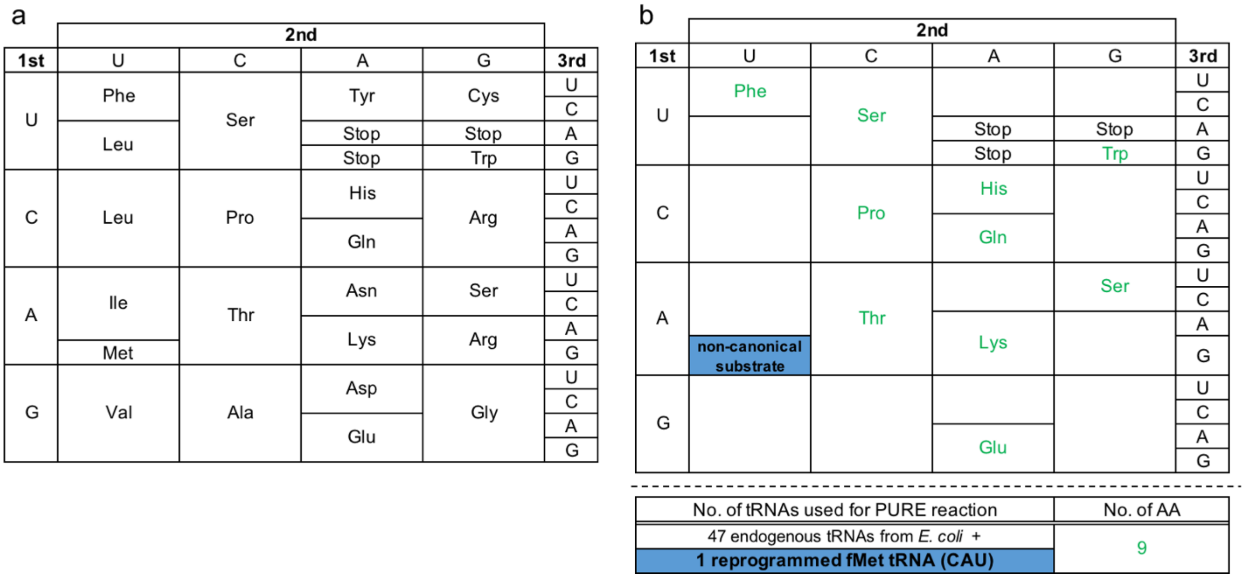

Supplementary Figure 9. Reprogramming fMet^{tRNA} with noncanonical substrates. a) The codon table. b) The noncanonical substrates on mis-acylated tRNA^{fMet} (CAU, blue box) were transferred to the initiating codon (AUG) of mRNA and incorporated into a peptide through the PURE system lacking Met. Only nine amino acids in green were used for the synthesis of a peptide. The synthesized peptides were characterized in **Fig. S10-15** and **18.**

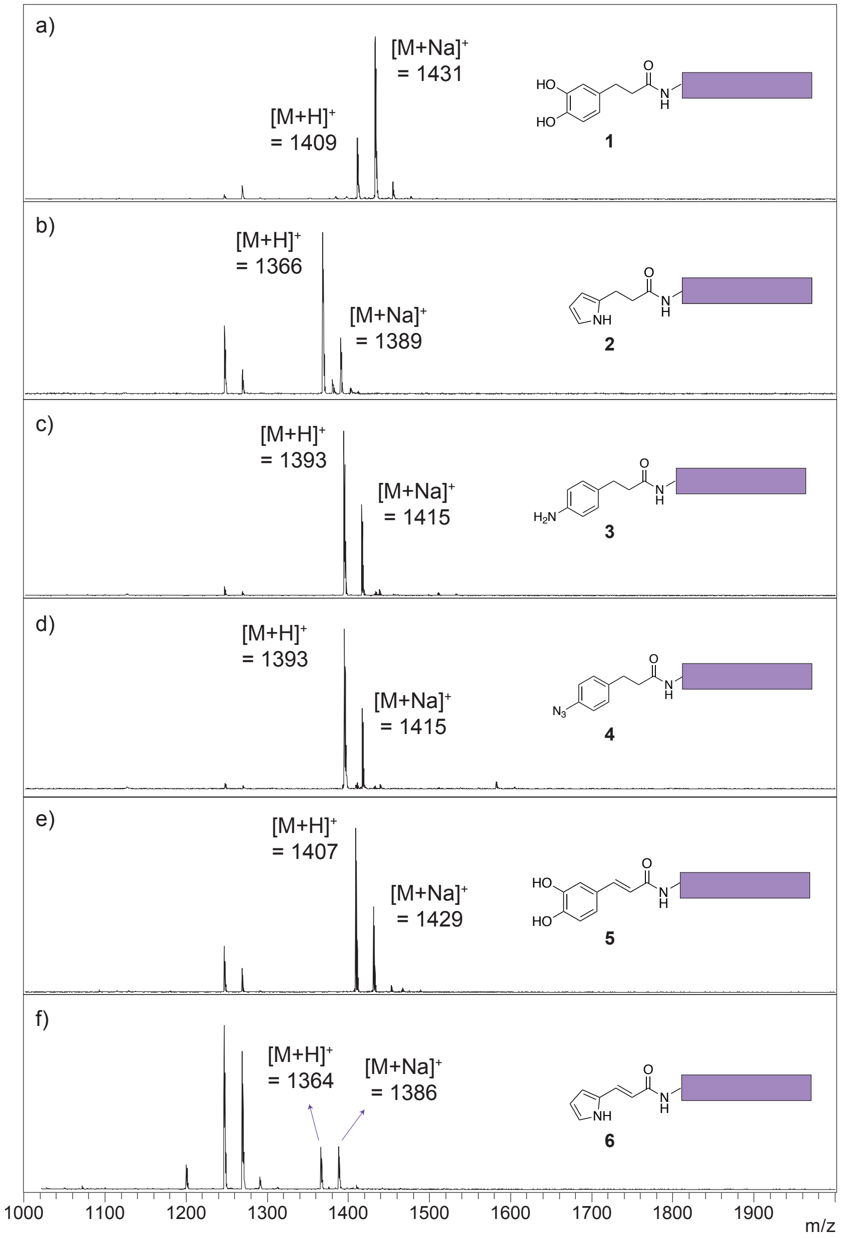

Supplementary Figure 10. Characterization of the N-terminus functionalized peptide with the Phe analogues.

The theoretical mass of each peptides are **a)** [M+H]$^+$ = 1410; [M+Na]$^+$ = 1432, **b)** [M+H]$^+$ = 1367; [M+Na]$^+$ = 1389, **c)** [M+H]$^+$ = 1393; [M+Na]$^+$ = 1415, **d)** [M+H]$^+$ = 1419, [M+H]$^+$ = 1393; [M+Na]$^+$ = 1415, **e)** [M+H]$^+$ = 1408; [M+Na]$^+$ = 1430, **f)** [M+H]$^+$ = 1365; [M+Na]$^+$ = 1387. The azide group in **4** was found to be reduced to an amino group because of 1,4-dithiothreitol (DTT) present in the PURE system[5].

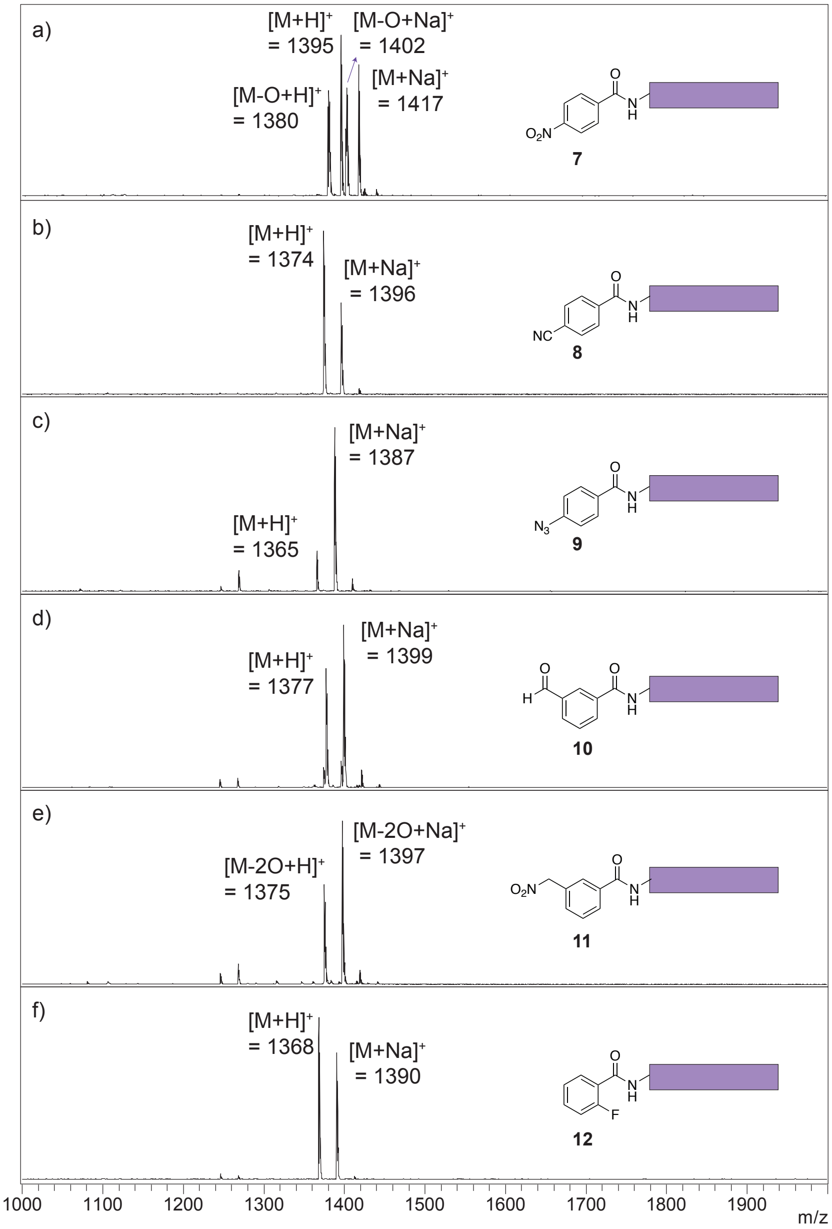

Supplementary Figure 11. Characterization of the N-terminus functionalized peptide with the EWG-containing benzoic acid derivatives. The theoretical mass of each peptides are **a)** $[M\text{-}O+H]^+$ = 1379; $[M+H]^+$ = 1395; $[M+Na]^+$ = 1401; $[M+Na]^+$ = 1417, **b)** $[M+H]^+$ = 1375; $[M+Na]^+$ = 1397, **c)** $[M+H]^+$ = 1391; $[M+H]^+$ = 1365; $[M+Na]^+$ = 1387, **d)** $[M+H]^+$ = 1378; $[M+Na]^+$ = 1400, **e)** $[M+H]^+$ = 1409; $[M\text{-}2O+H]^+$ = 1377; $[M\text{-}2O+Na]^+$ = 1399, **f)** $[M+H]^+$ = 1368; $[M+Na]^+$ = 1390. The azide group in **9** was found to be reduced to an amino group.

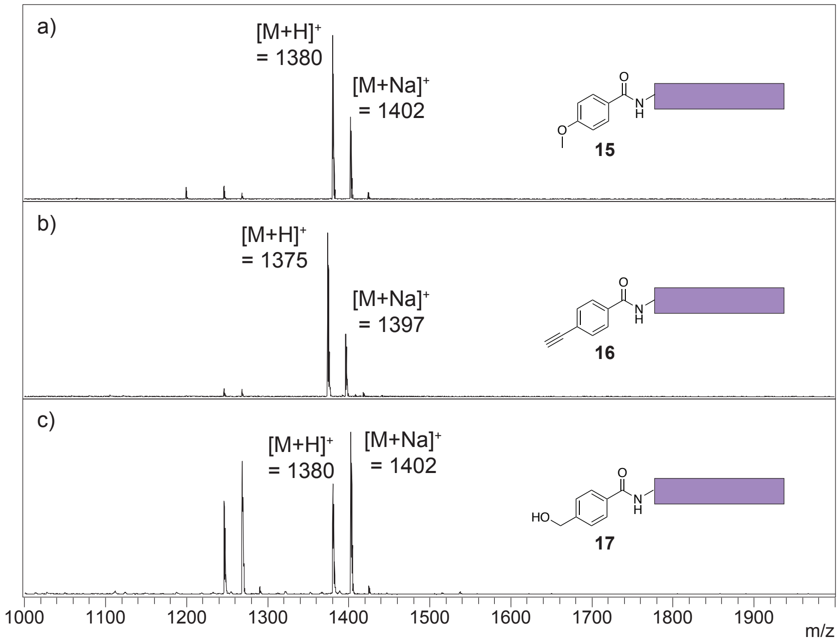

Supplementary Figure 12. Characterization of the N-terminus functionalized peptide with the EDG-containing benzoic acids. The theoretical mass of each peptides are **a)** $[M+H]^+$ = 1380; $[M+Na]^+$ = 1402, **b)** $[M+H]^+$ = 1374; $[M+Na]^+$ = 1396, **c)** $[M+H]^+$ = 1380; $[M+Na]^+$ = 1402.

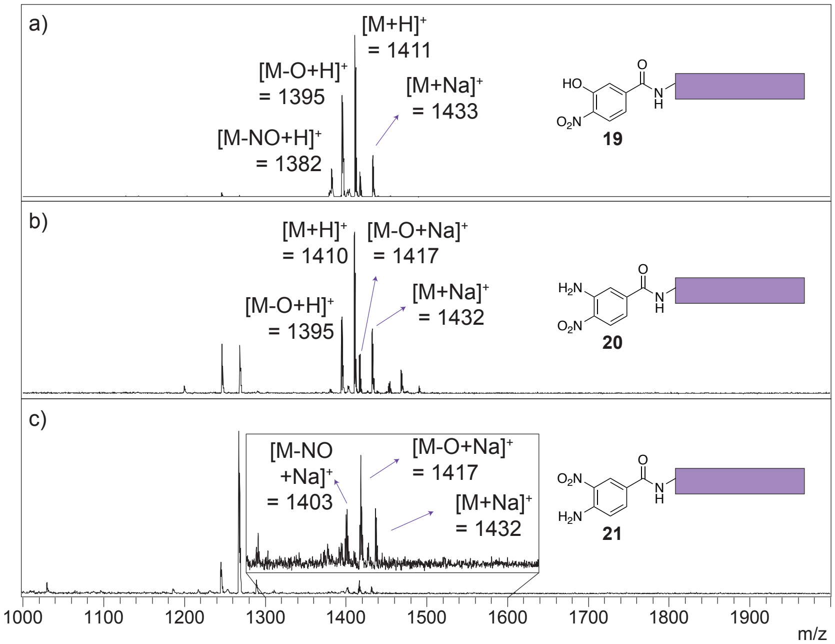

Supplementary Figure 13. Characterization of the N-terminus functionalized peptide with the benzoic acid derivatives containing both EWG and EDG. The theoretical mass of each peptides are **a)** $[M+H]^+$ = 1411, $[M-O+H]^+$ = 1395, $[M-NO+H]^+$ = 1382, $[M+Na]^+$ = 1433, **b)** $[M+H]^+$ = 1410; $[M-O+H]^+$ = 1394; $[M+Na]^+$ = 1416, $[M+Na]^+$ = 1432, **c)** The range of 1300-1600 was expanded for clear peak assignment. $[M-NO+Na]^+$ = 1402; $[M-NO+Na]^+$ = 1416; $[M+Na]^+$ = 1432.

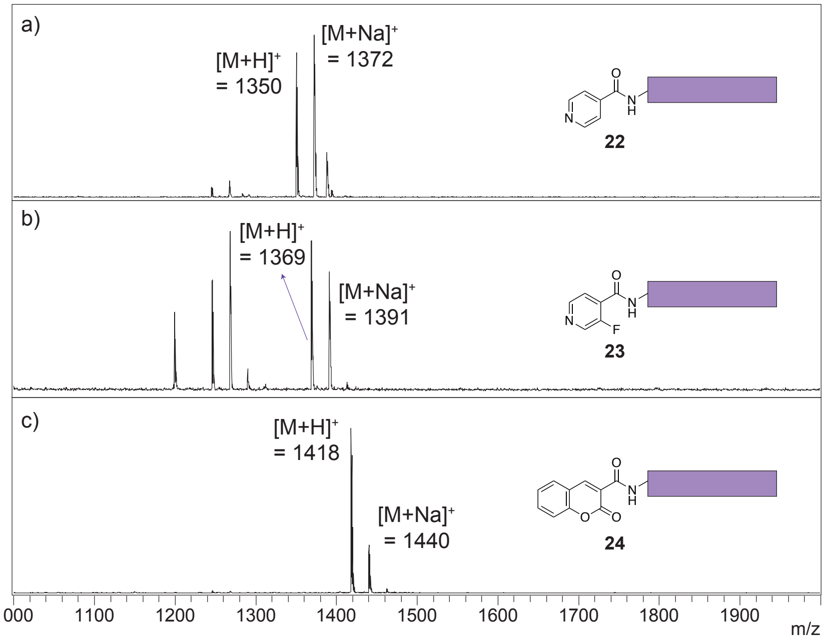

Supplementary Figure 14. Characterization of the N-terminus functionalized peptide with the heteroaromatic substrates. The theoretical mass of each peptides are **a)** $[M+H]^+$ = 1350, $[M+Na]^+$ = 1372, **b)** $[M+H]^+$ = 1369, $[M+Na]^+$ = 1391, **c)** $[M+H]^+$ = 1418, $[M+Na]^+$ = 1440.

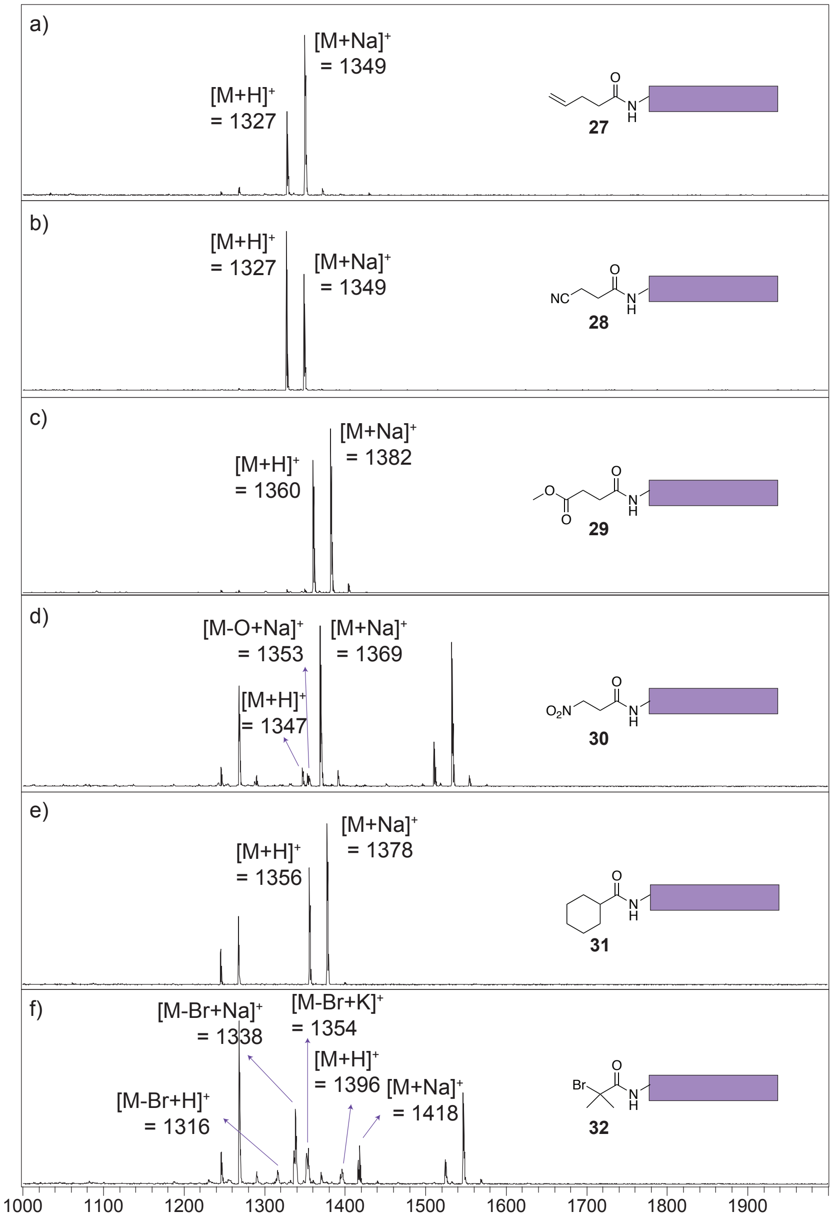

Supplementary Figure 15. Characterization of the N-terminus functionalized peptide with the aliphatic substrates. The theoretical mass of each peptides are **a)** $[M+H]^+$ = 1328; $[M+Na]^+$ = 1350, **b)** $[M+H]^+$ = 1327; $[M+Na]^+$ = 1349, **c)** $[M+H]^+$ = 1360; $[M+Na]^+$ = 1382, **d)** $[M+H]^+$ = 1347; $[M+Na]^+$ = 1369, **e)** $[M+H]^+$ = 1356; $[M+Na]^+$ = 1378, **f)** $[M+H]^+$ = 1395; $[M+Na]^+$ = 1417.

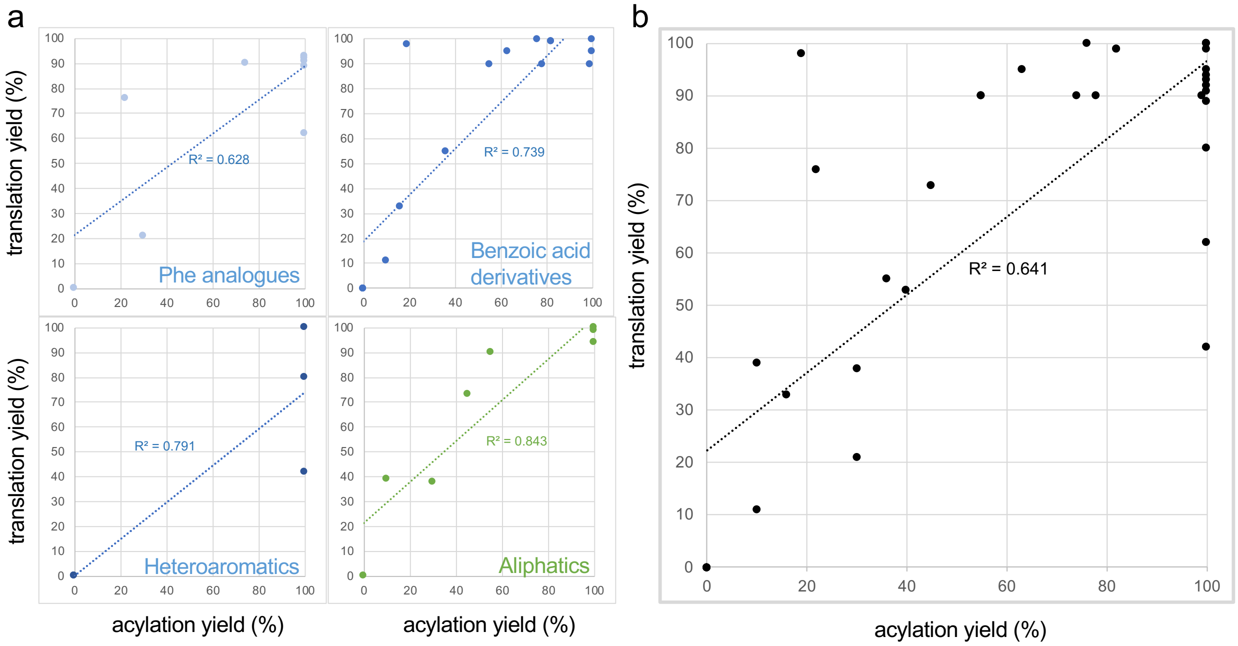

Supplementary Figure 16. Translation efficiency for the noncanonical substrates. Individual value for acylation and protein yield are plotted and fitted to a linear function to show correlation of translation efficiency for mis-acylated tRNAs containing a noncanonical amino acid. The data points grouped in each category of substrates **(a**, r = 0.628, 0.739, 0.791, and 0.843 for Phe analogues, benzoic acid derivatives, heteroaromatics, and aliphatics, respectively**)** and from all 32 noncanonical substrates **(b**, $r_{average}$ = 0.641**)** show a good correlation, suggesting that the substrates charged with higher acylation yield into tRNA tend to yield higher amount of desired peptide containing a noncanonical substrate at the N-terminus. The translation yields were obtained by quantifying the relative ratio of peak area of the desired peptide in the mass spectra shown in **Fig. 5** and **Fig. S10-15**.

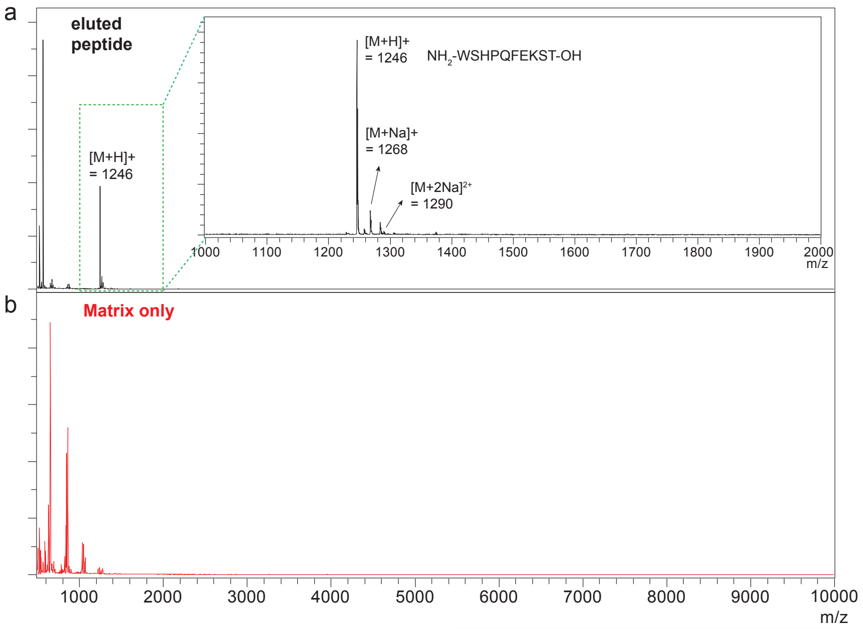

c

m/z	Peak area (au)	
505.9	21	
521.9	29	
522.9	19	
523.9	416	
549.9	45	
565.8	21	
567.9	1904	
586.8	28	
589.8	22	
649.7	23	
655.7	46	
665.7	25	
687.6	36	
860.6	74	
876.6	59	
1245.9	2482	: $[M+H]^+$
1257.9	72	
1268.0	317	: $[M+Na]^+$
1283.9	168	
1289.9	13	: $[M+2Na]^{2+}$
1305.7	4	
1375.0	10	
	51%	: yield of peptide NH$_2$-WSHPQFEKST-OH

d

substrate	peak area (%) of the peptide	yield (%) of target peptide
A	40	20
B	91	46
C	90	46
D	33	17
E	53	27
G	90	46
1	92	47
2	62	32
3	93	47
4	89	45
5	21	11
6	76	39
7	99	50
8	99	50
9	90	46
10	95	48
11	90	46
12	99	50
13	N/A	N/A
14	N/A	N/A
15	95	48
16	98	50
17	55	28
18	N/A	N/A
19	99	50
20	90	46
21	11	6
22	80	41
23	42	21
24	100	51
25	N/A	N/A
26	N/A	N/A
27	94	48
28	100	51
29	100	51
30	38	19
31	73	37
32	39	20

Supplementary Figure 17. Percent yield of peptides containing the noncanonical chemical substrates. The percent yield of each peptide was calculated based on the relative peak area shown in the MALDI spectra (**Figure 5** and **Figure S10-15**) by using the peptide prepared in the absences of Met (NH$_2$-WSHPQFEKST-OH, **Figure 5c**) as an internal standard. **a)** The percent yield of NH$_2$-WSHPQFEKST-OH was calculated based on the relative peak area of the peptides shown in the mass spectrum from 500 to 10,000 Da. The enzymes present in the PURE system were removed using Ni-NTA-coated magnetic

beads. See the experimental sections for details. **b)** The impurity peaks were found in the matrix (CHCA; α-Cyano-4-hydroxycinnamic acid). **c)** The peak info found in a). The peaks (red) found in b) were not included for the calculation of the relative yield of NH_2-WSHPQFEKST-OH (54 %). **d)** The yields of the peptides containing the noncanonical chemical substrates at the N-terminus were obtained by multiplying the relative ratio of the peak area shown in **Figure 5** and **Figure S10-15**.

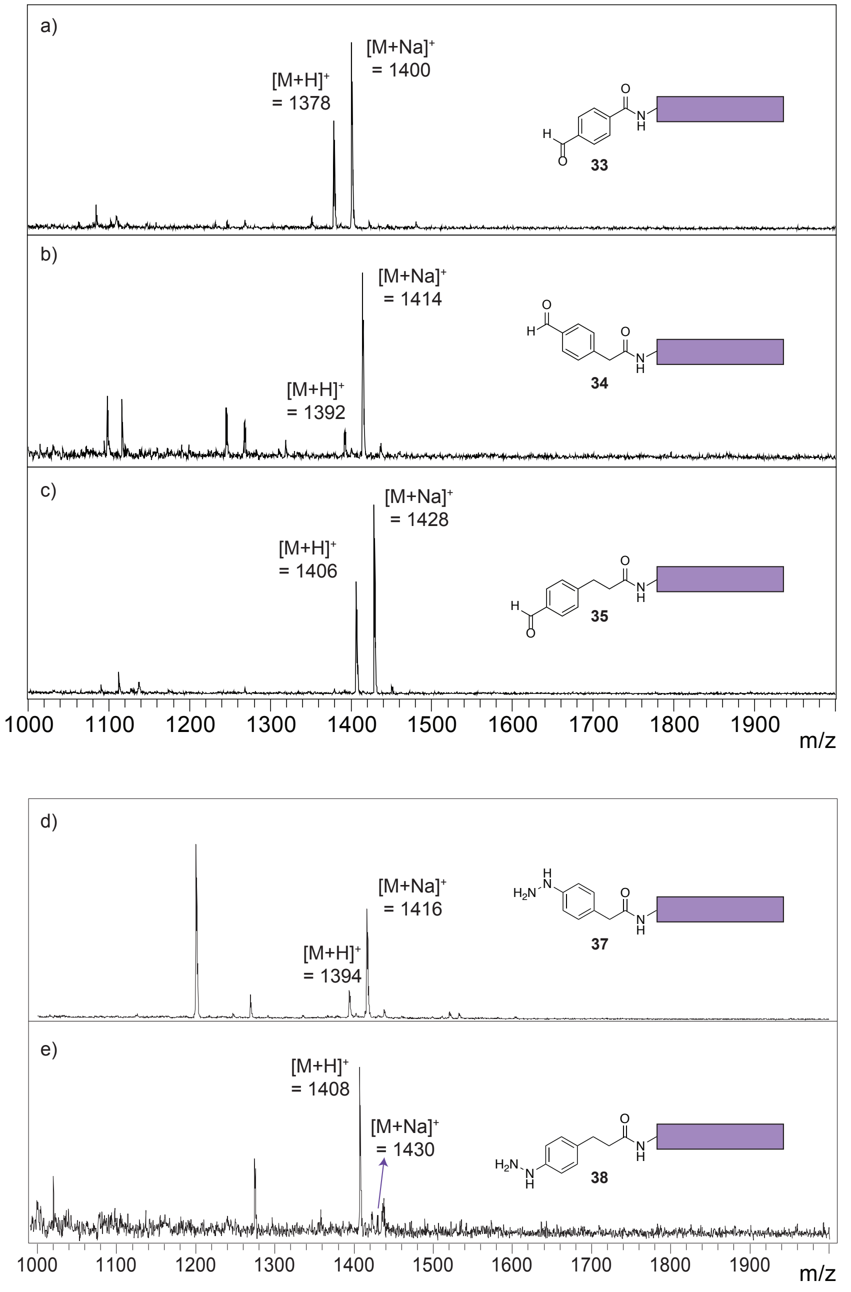

Supplementary Figure 18. Characterization of the N-terminus functionalized peptide with the aldehyde and hydrazine substrates. The theoretical mass of each peptides are **a)** $[M+H]^+$ = 1378, $[M+Na]^+$ = 1400, **b)** $[M+H]^+$ = 1392, $[M+Na]^+$ = 1414, **c)** $[M+H]^+$ = 1406, $[M+Na]^+$ = 1428. **d)** $[M+H]^+$ = 1394, $[M+Na]^+$ = 1416. **e)** $[M+H]^+$ = 1408, $[M+Na]^+$ = 1430.

Supplementary Figure 19. ^{1}H NMR (500 MHz, $CDCl_3$) of **B.**

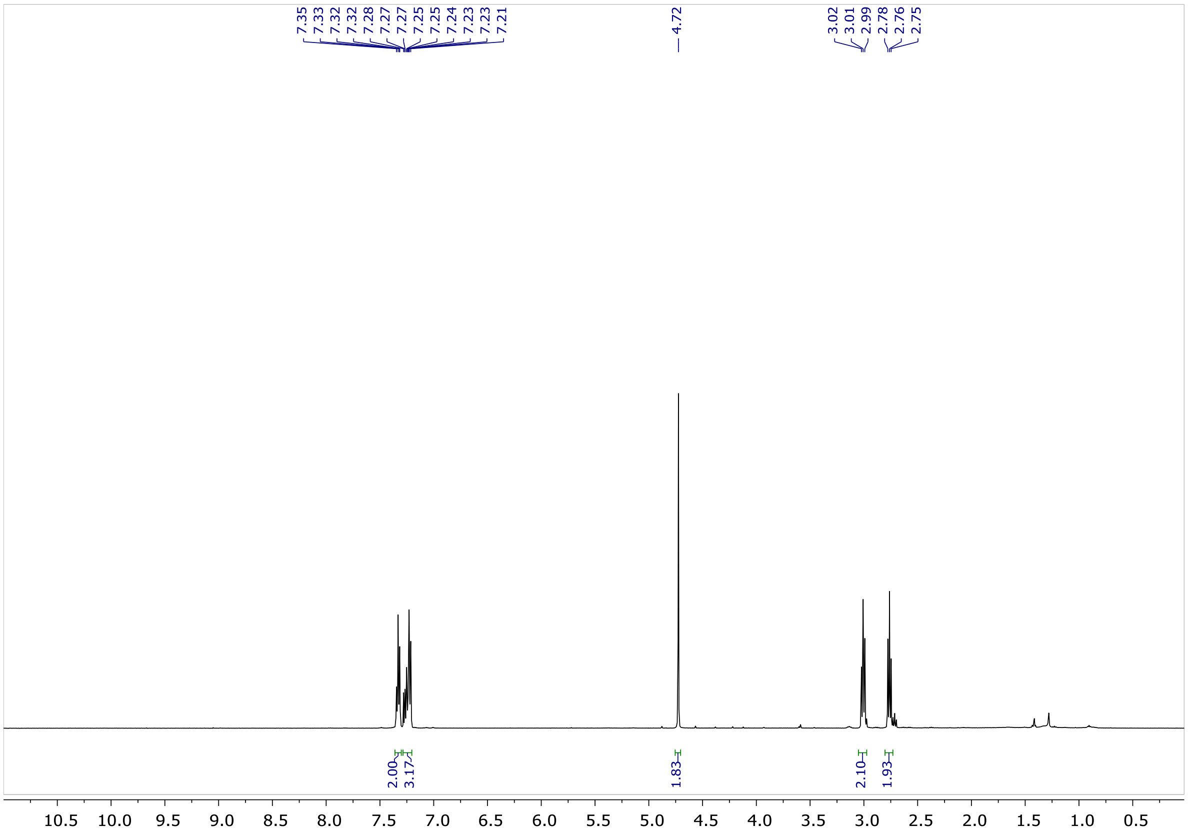

Supplementary Figure 20. ^{13}C NMR (125 MHz, $CDCl_3$) of **B.**

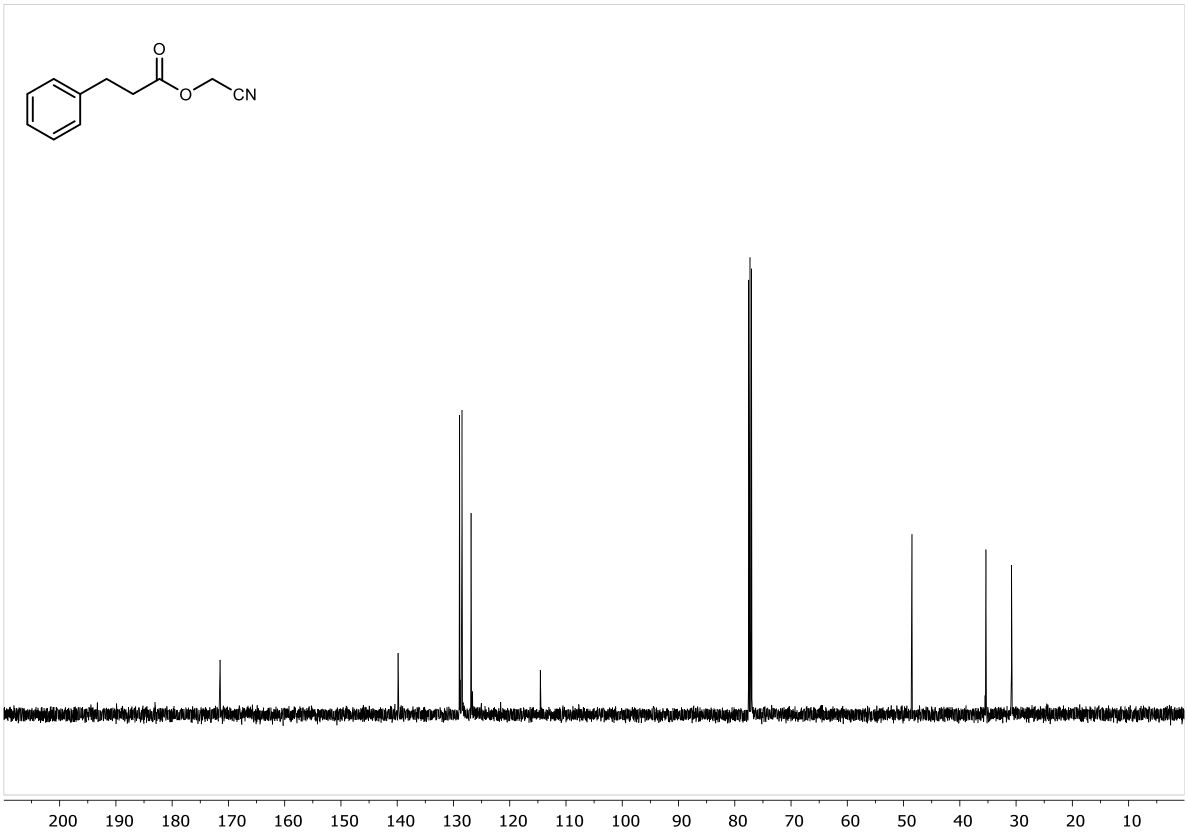

Supplementary Figure 21. ^{1}H NMR (500 MHz, $CDCl_3$) of **C.**

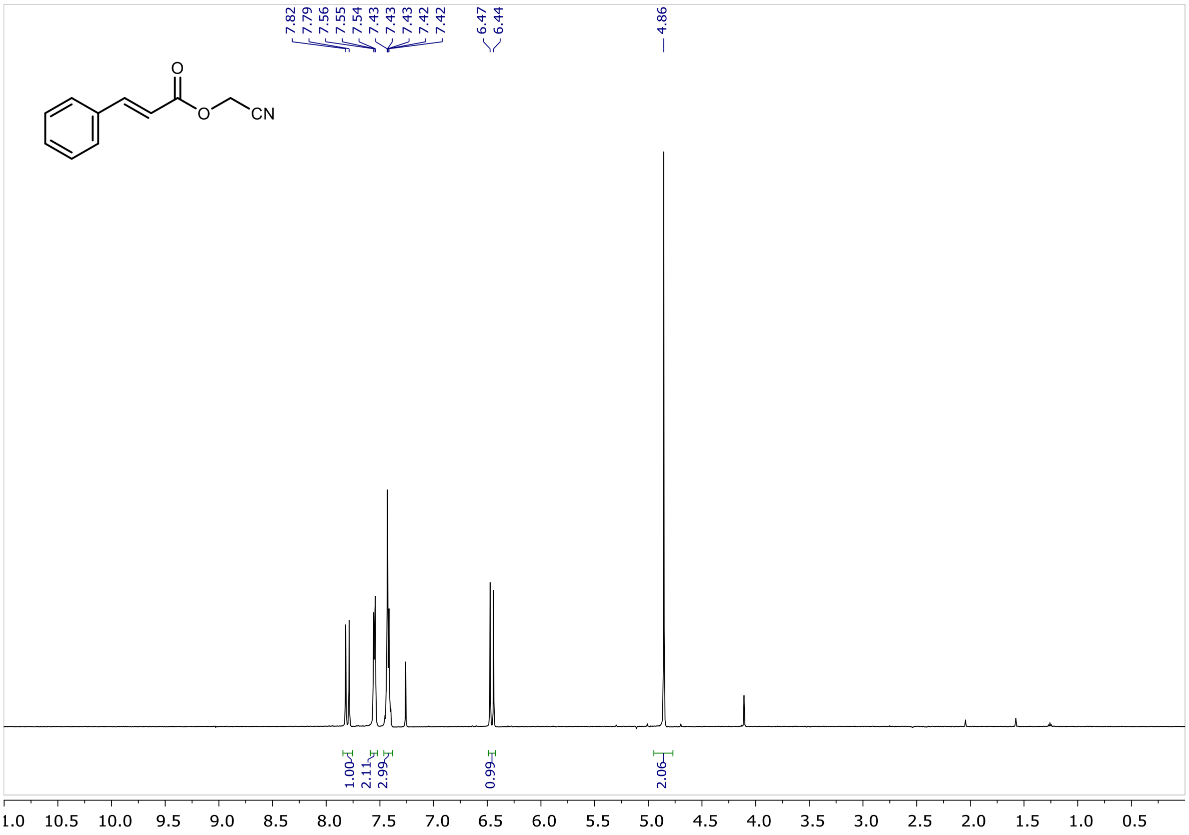

Supplementary Figure 22. ^{13}C NMR (125 MHz, $CDCl_3$) of **C.**

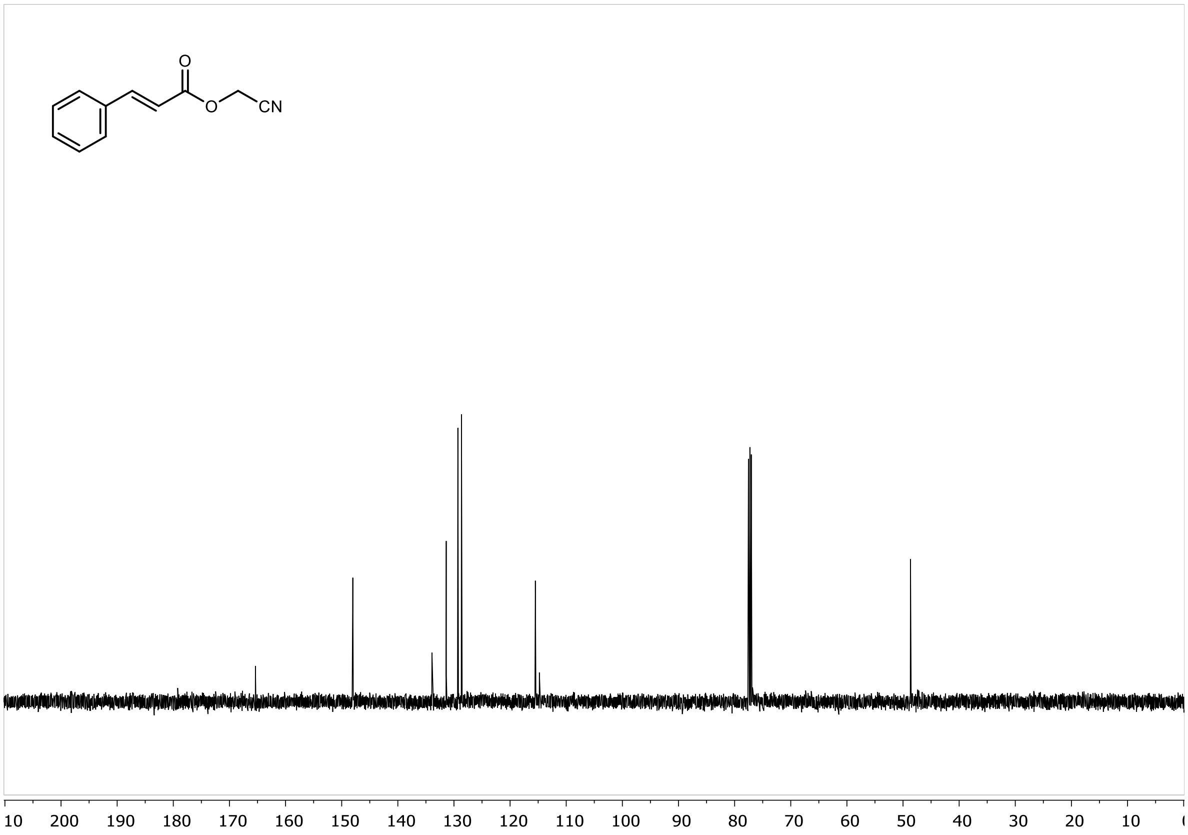

Supplementary Figure 23. ^{1}H NMR (500 MHz, $CDCl_3$) of **D.**

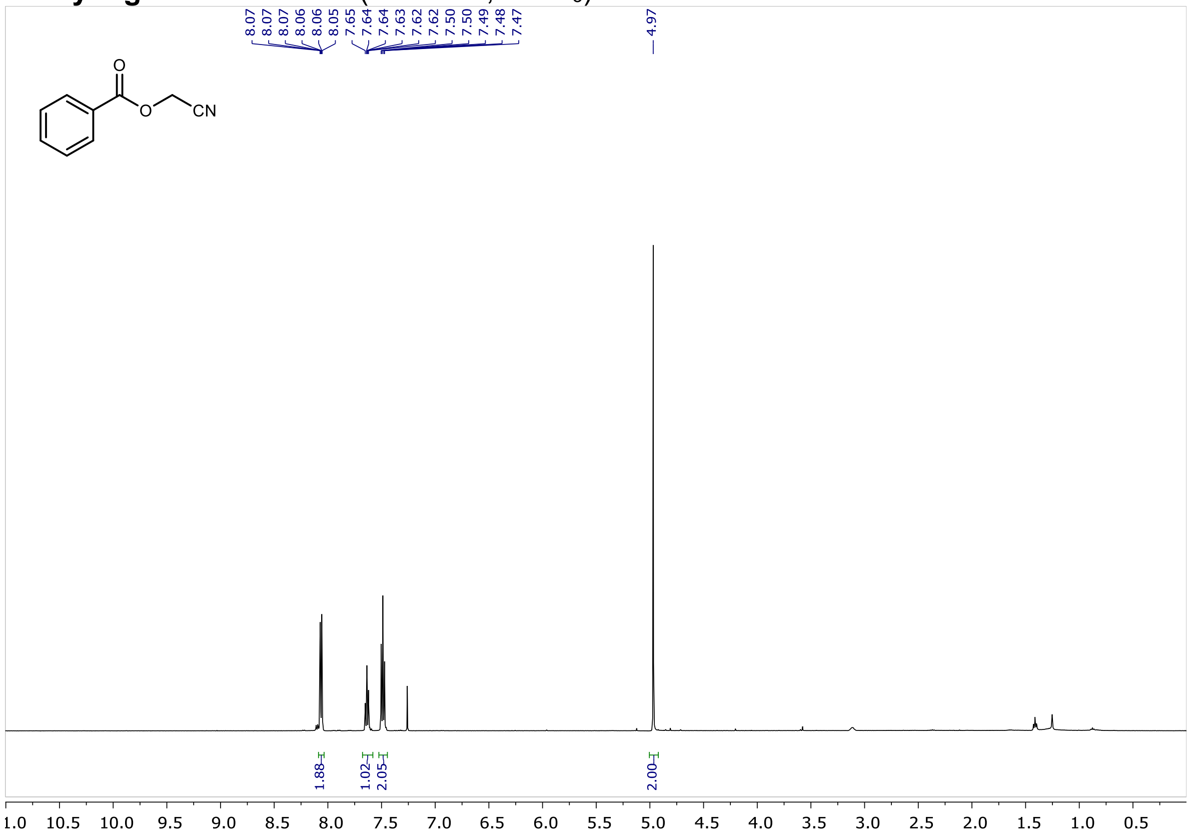

Supplementary Figure 24. ^{13}C NMR (125 MHz, $CDCl_3$) of **D.**

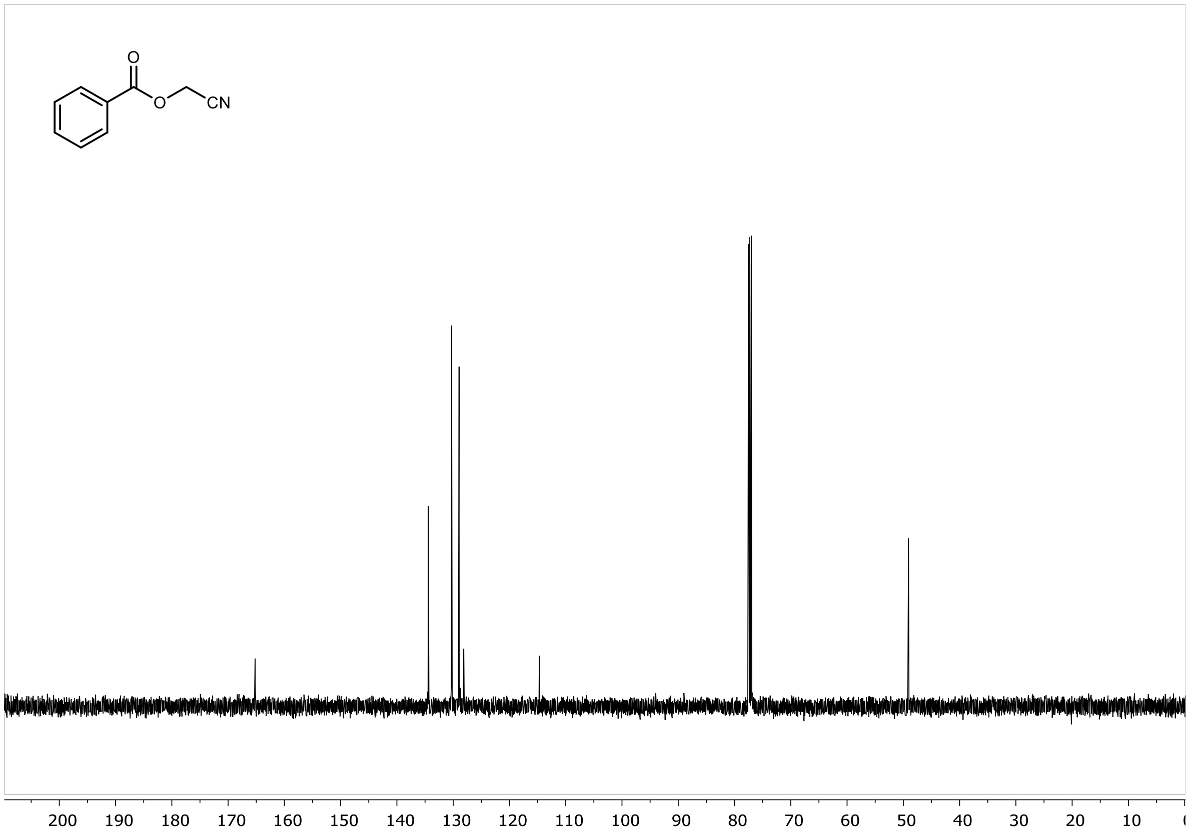

Supplementary Figure 25. ^{1}H NMR (500 MHz, $CDCl_3$) of **E.**

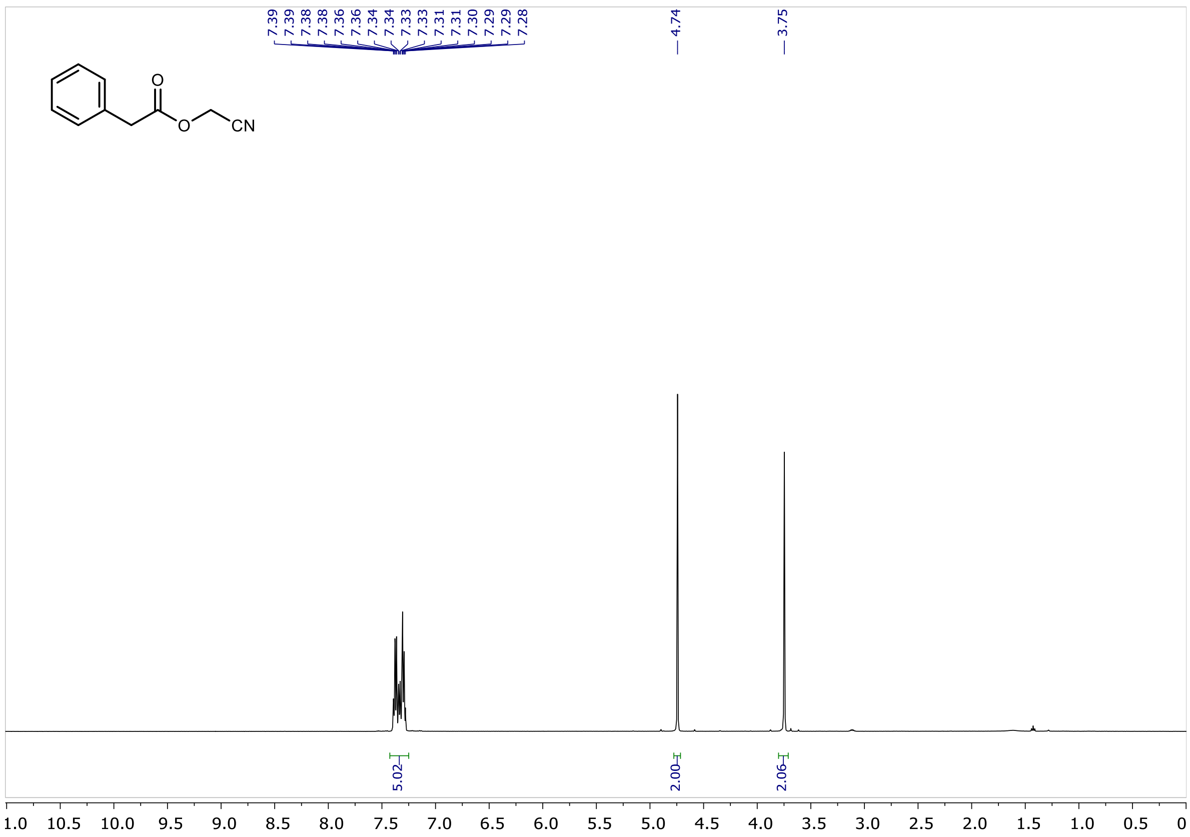

Supplementary Figure 26. ^{13}C NMR (125 MHz, $CDCl_3$) of **E.**

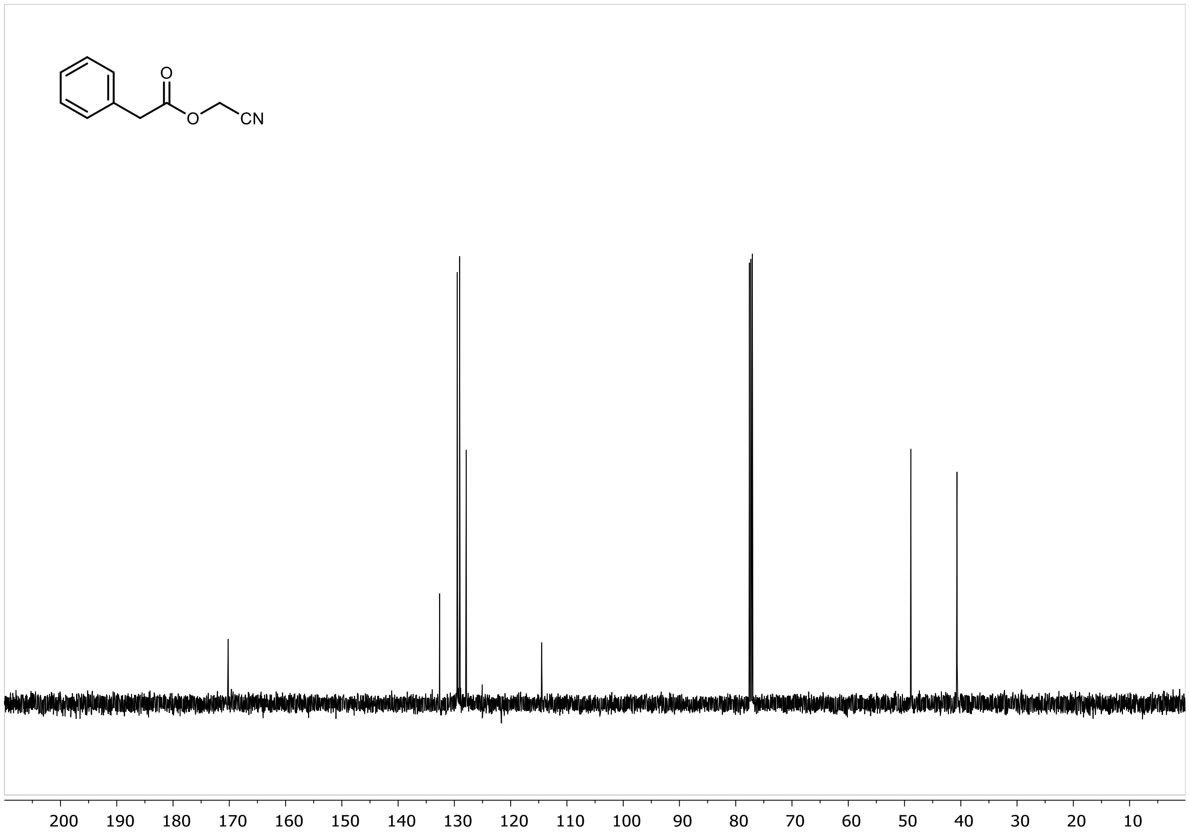

Supplementary Figure 27. ^{1}H NMR (500 MHz, $CDCl_3$) of **F.**

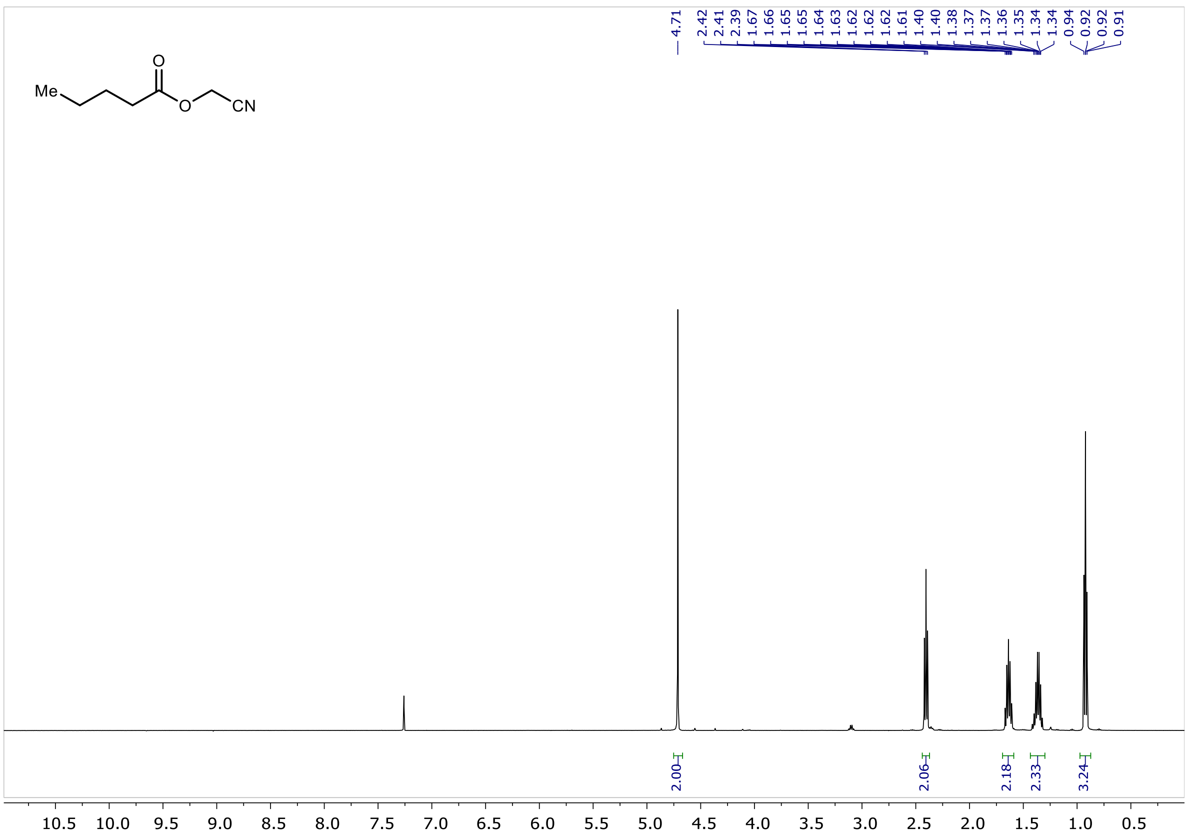

Supplementary Figure 28. ^{13}C NMR (125 MHz, $CDCl_3$) of **F.**

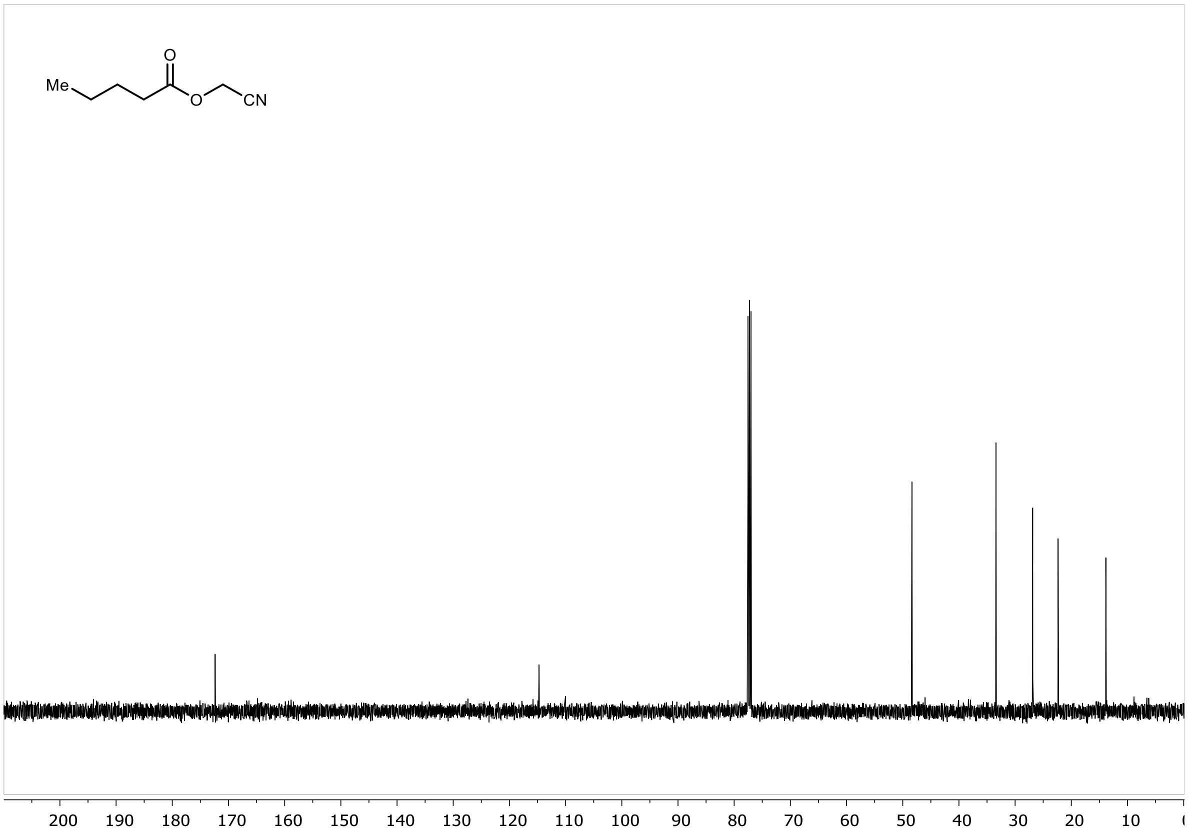

Supplementary Figure 29. ^{1}H NMR (500 MHz, DMSO-d_6) of **1.**

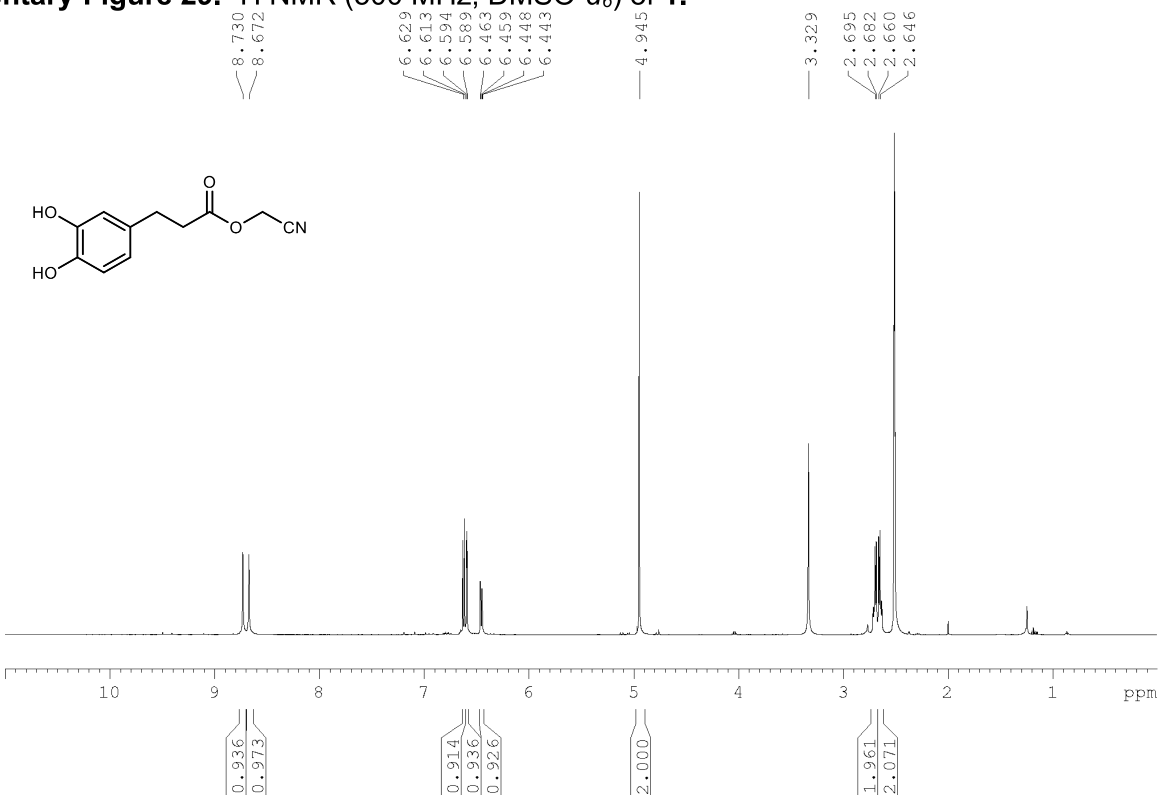

Supplementary Figure 30. ^{13}C NMR (125 MHz, DMSO-d_6) of **1.**

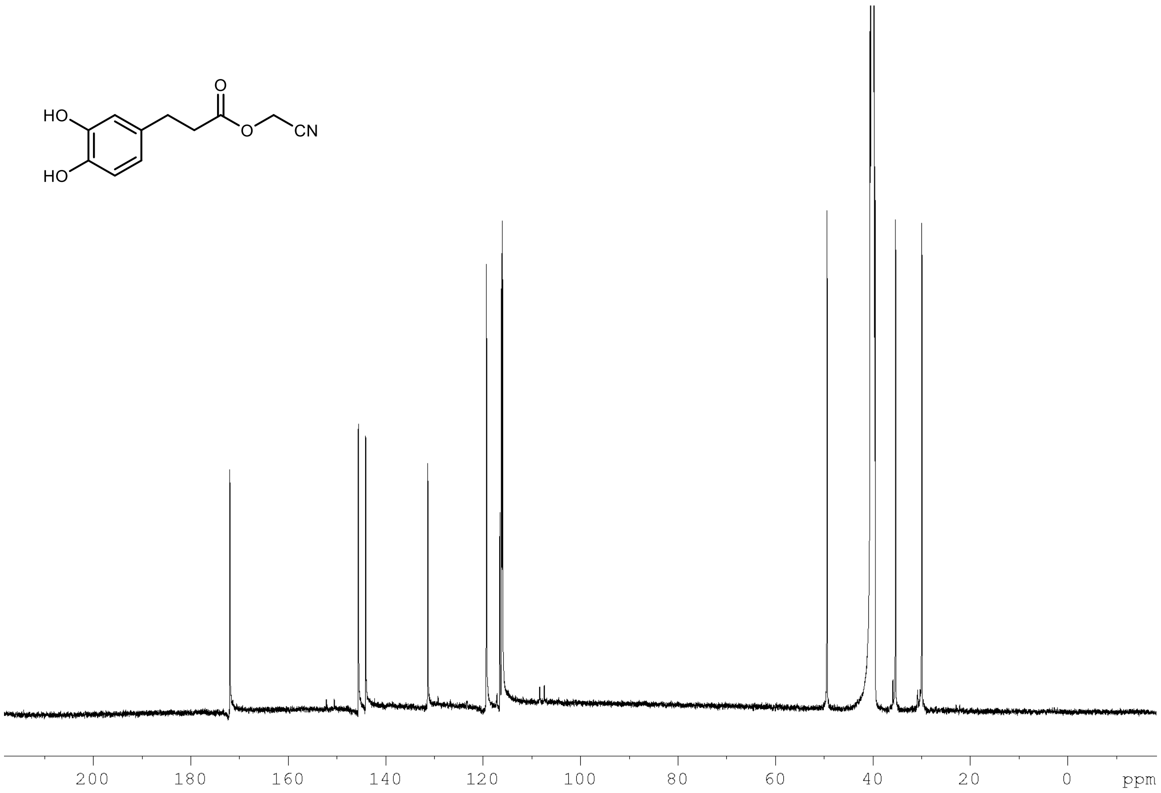

Supplementary Figure 31. ^{1}H NMR (500 MHz, DMSO-d_6) of **2.**

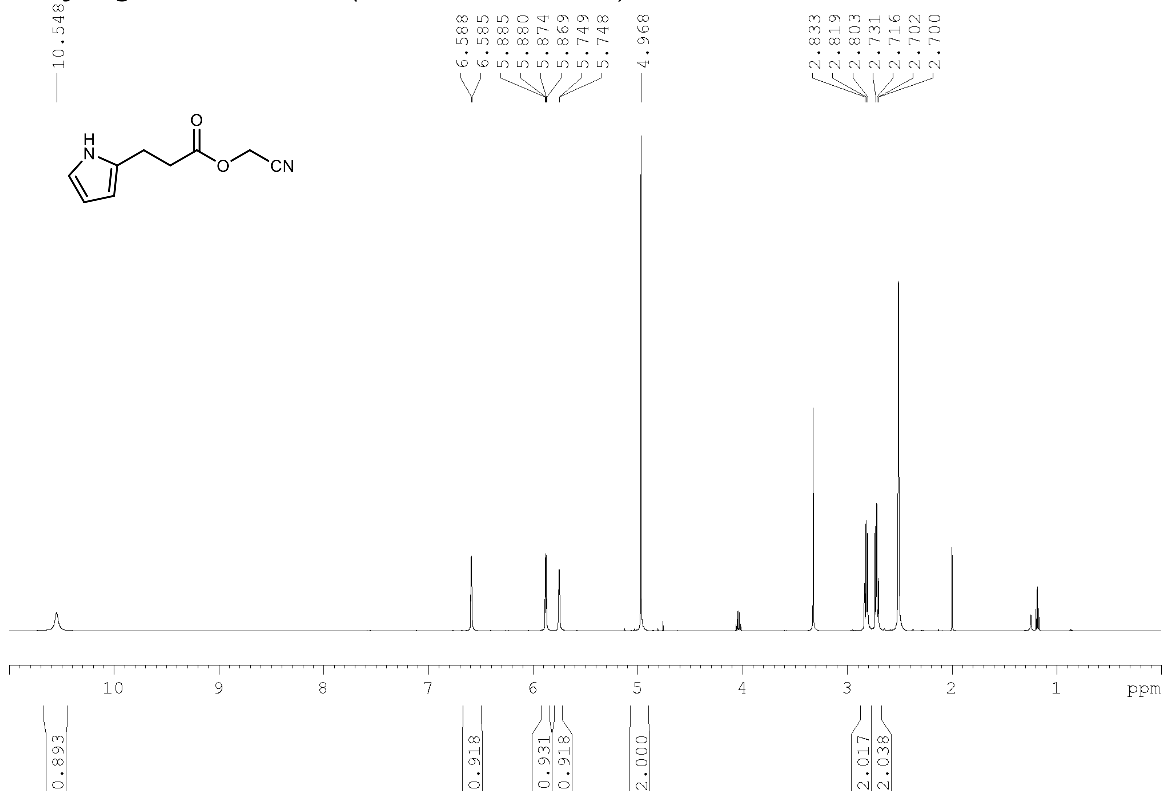

Supplementary Figure 32. ^{13}C NMR (125 MHz, DMSO-d_6) of **2.**

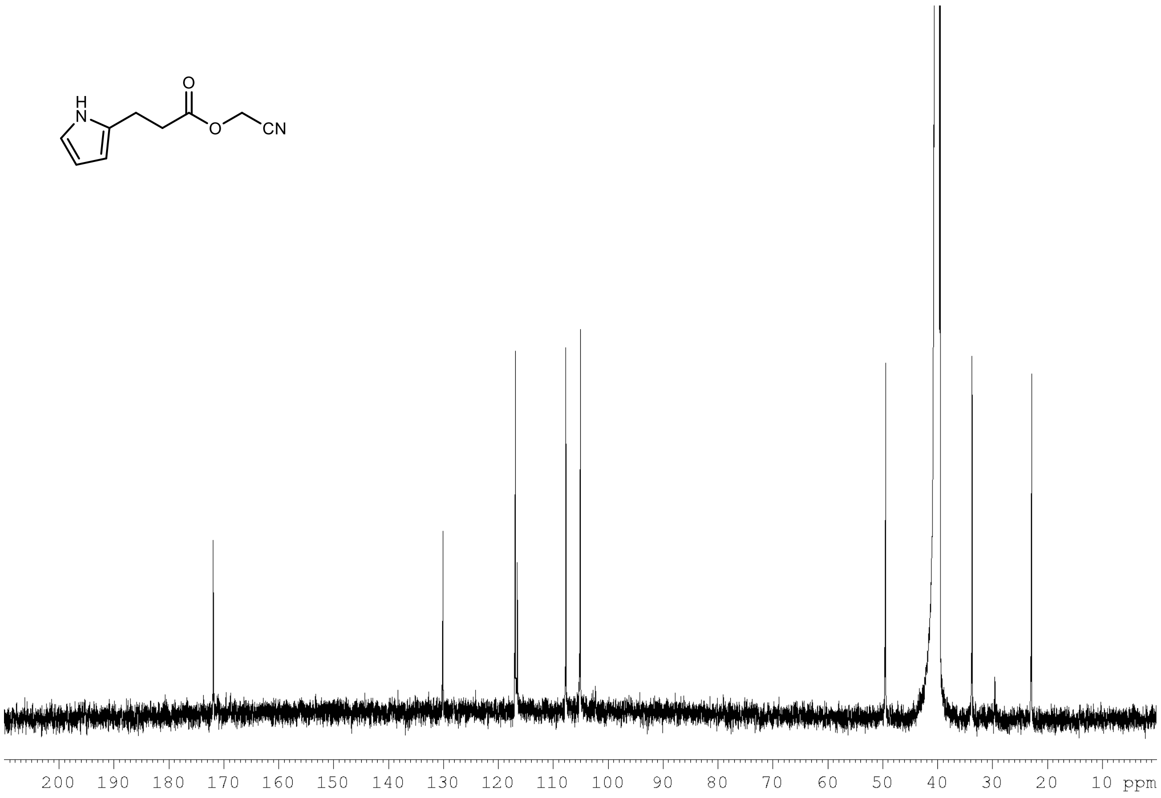

Supplementary Figure 33. ^{1}H NMR (500 MHz, $CDCl_3$) of **3.**

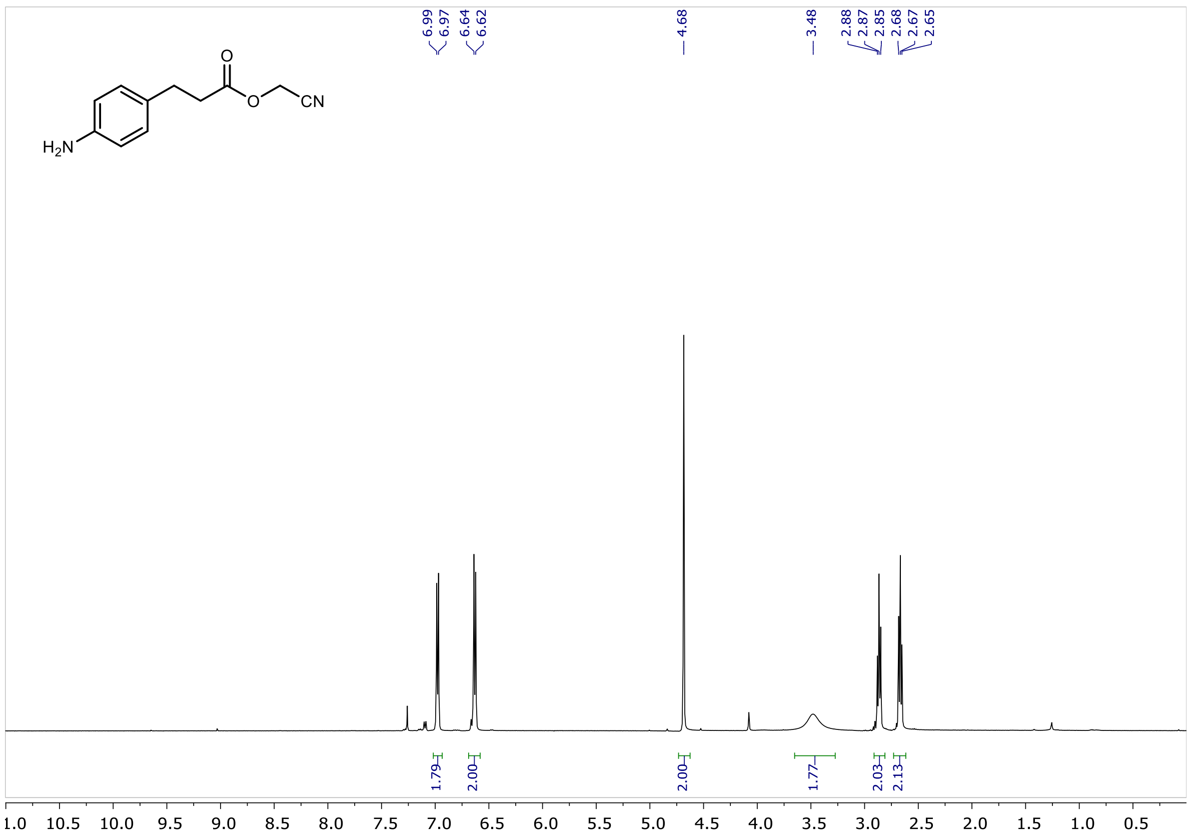

Supplementary Figure 34. ^{13}C NMR (125 MHz, $CDCl_3$) of **3.**

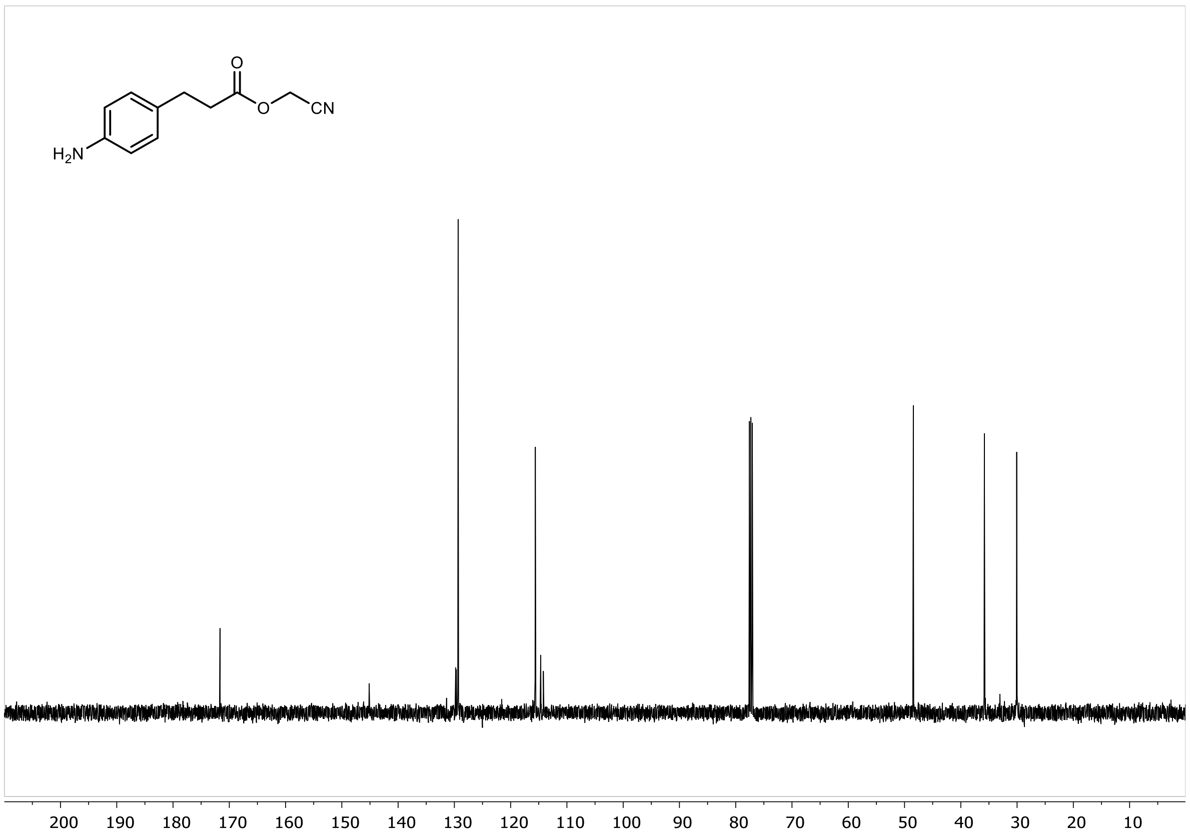

Supplementary Figure 35. ^{1}H NMR (500 MHz, CD_3CN) of **4.**

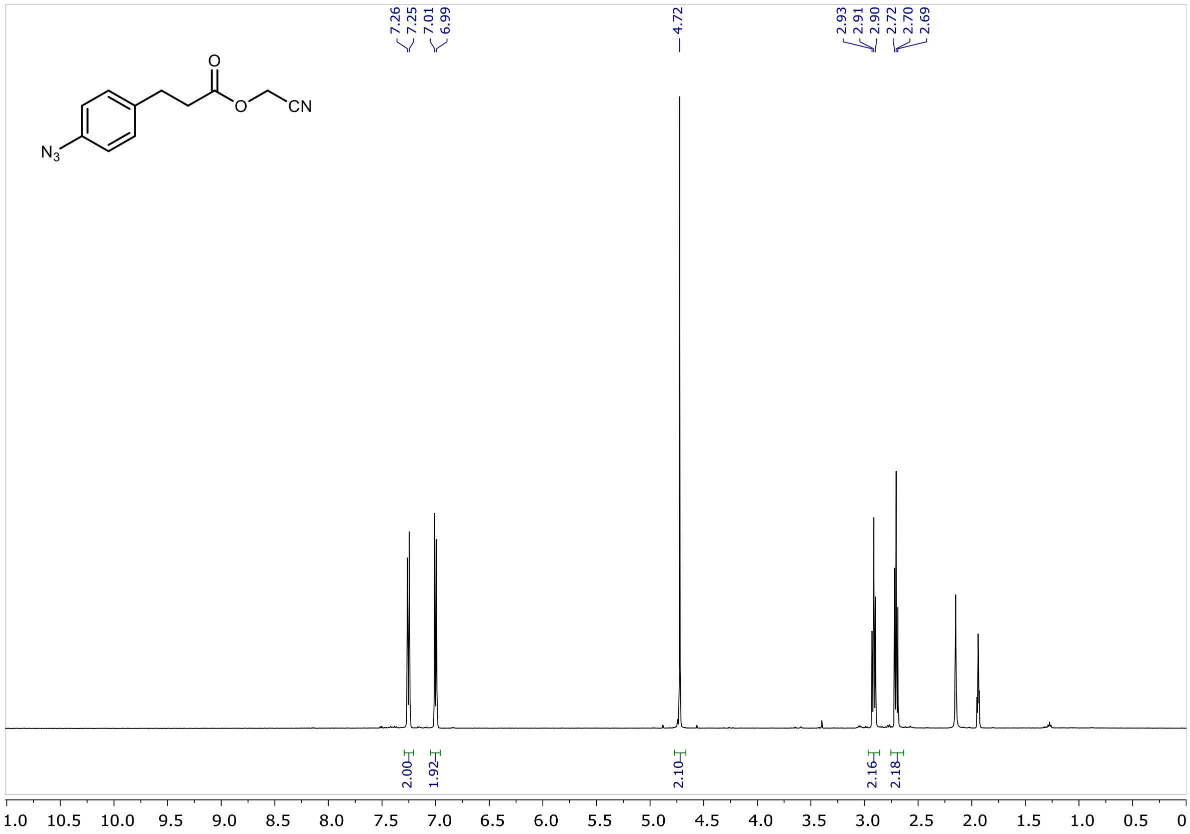

Supplementary Figure 36. ^{13}C NMR (125 MHz, CD_3CN) of **4.**

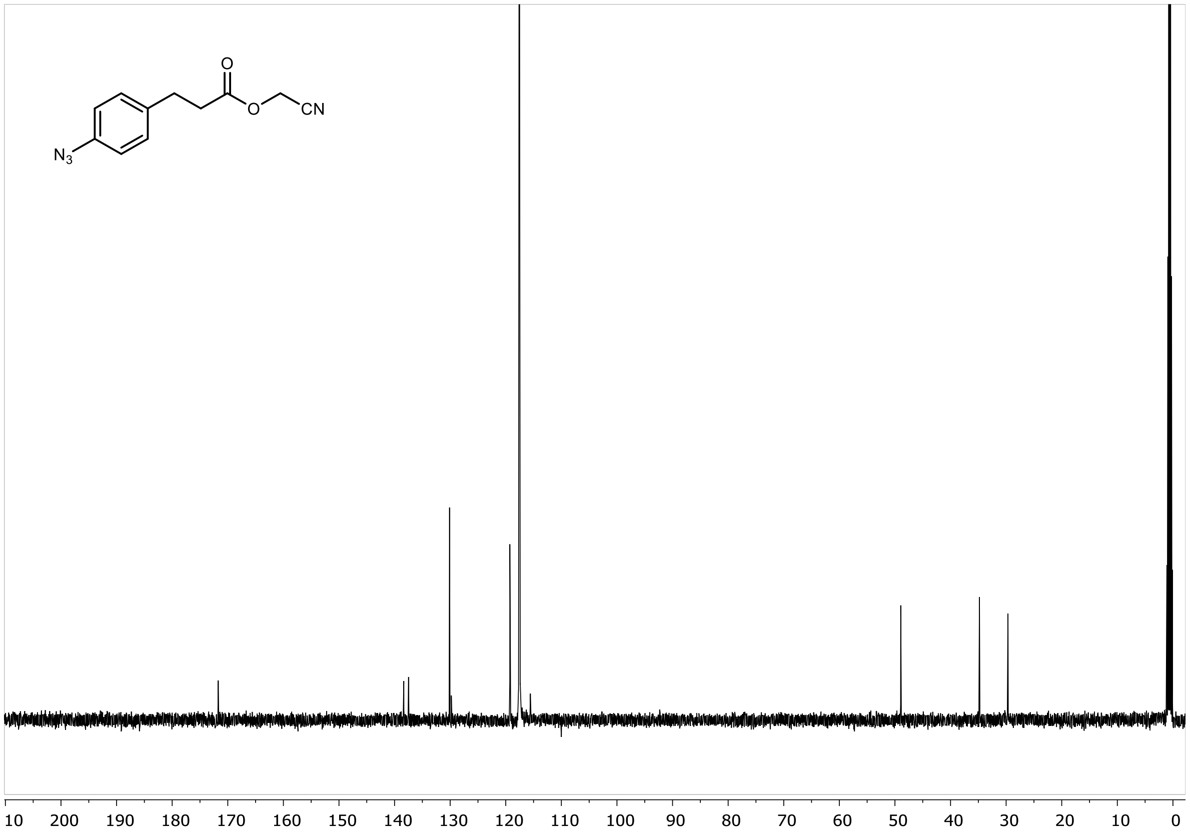

Supplementary Figure 37. ^{1}H NMR (500 MHz, DMSO-d_6) of **5.**

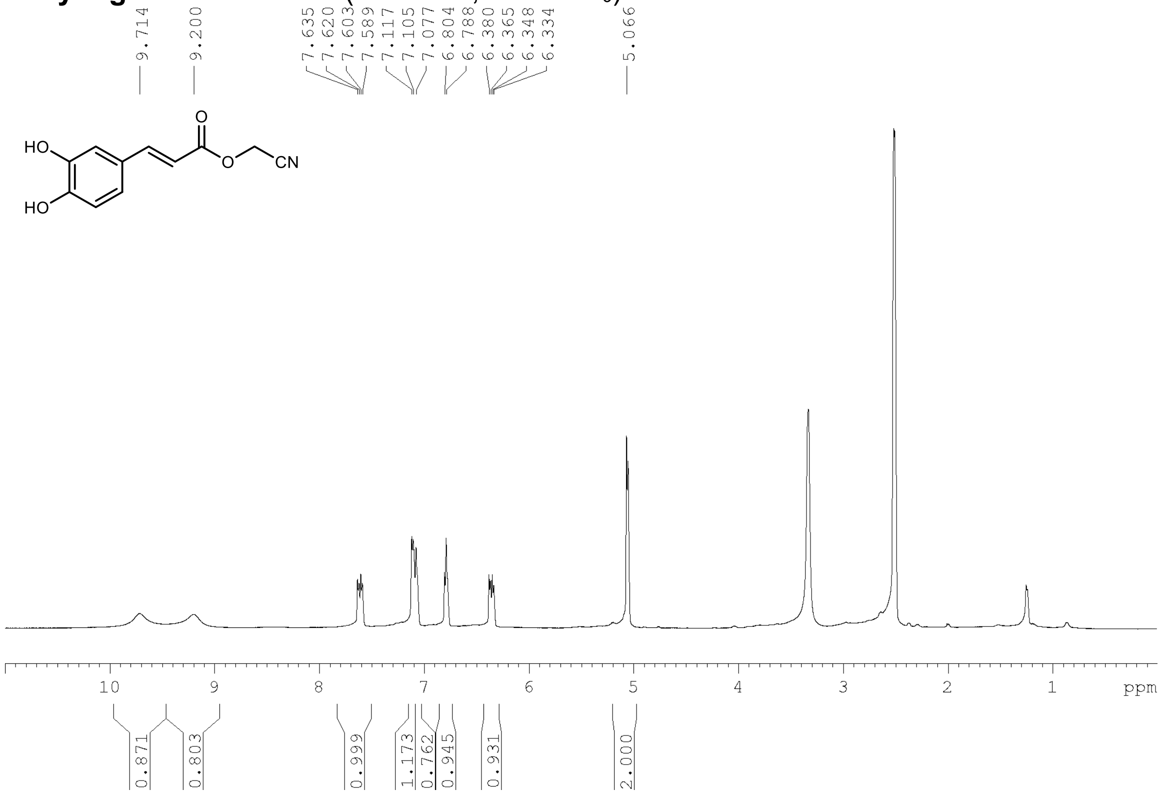

Supplementary Figure 38. ^{13}C NMR (125 MHz, DMSO-d_6) of **5.**

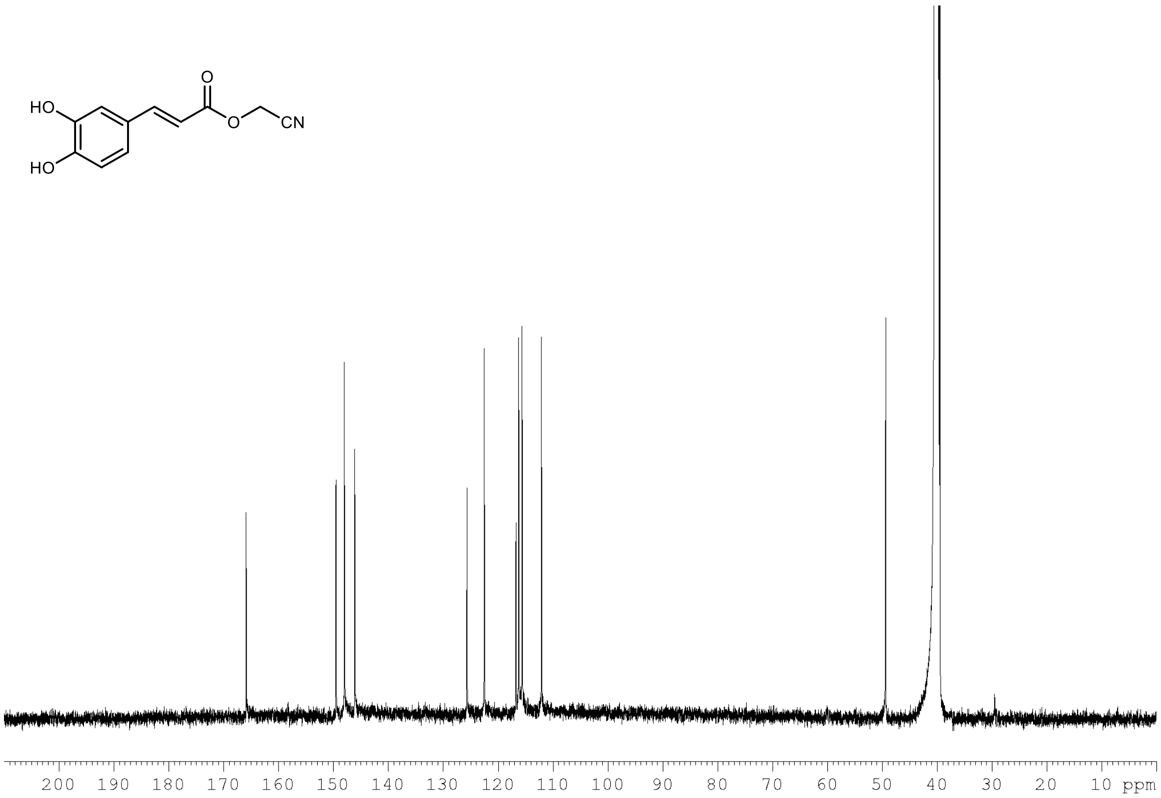

Supplementary Figure 39. ^{1}H NMR (500 MHz, DMSO-d_6) of **6.**

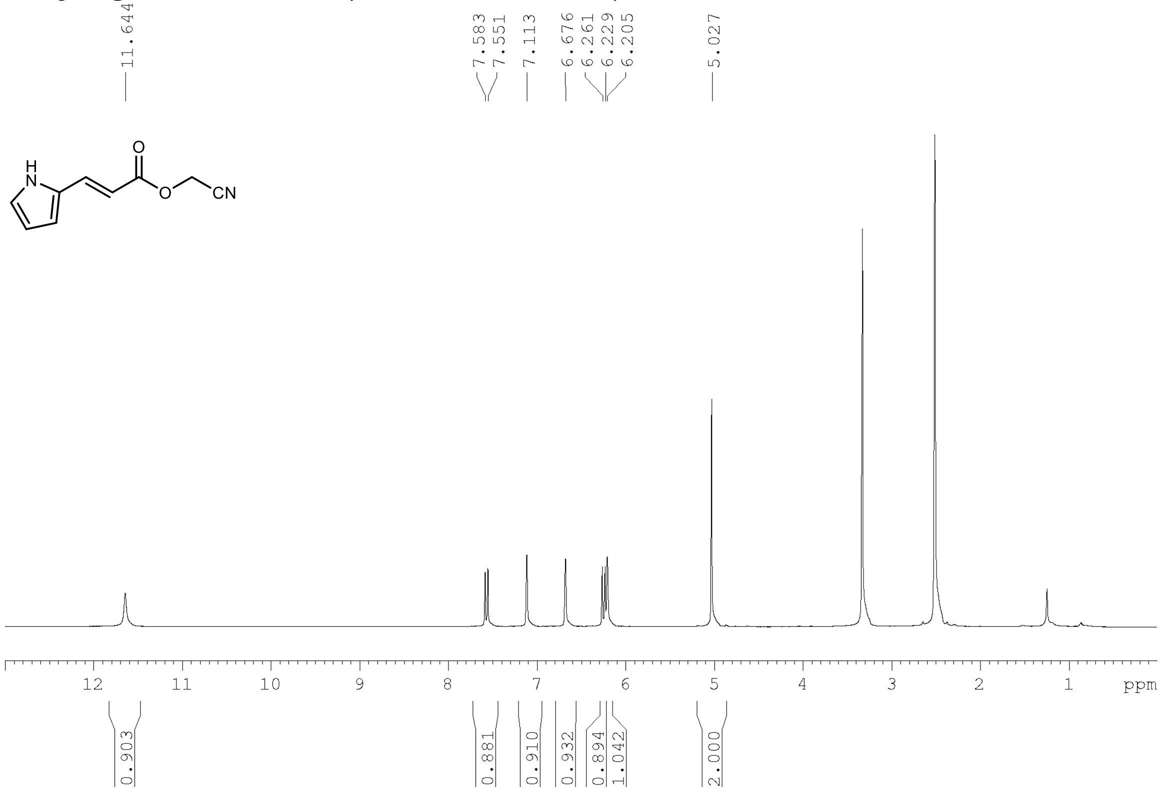

Supplementary Figure 40. ^{13}C NMR (125 MHz, DMSO-d_6) of **6.**

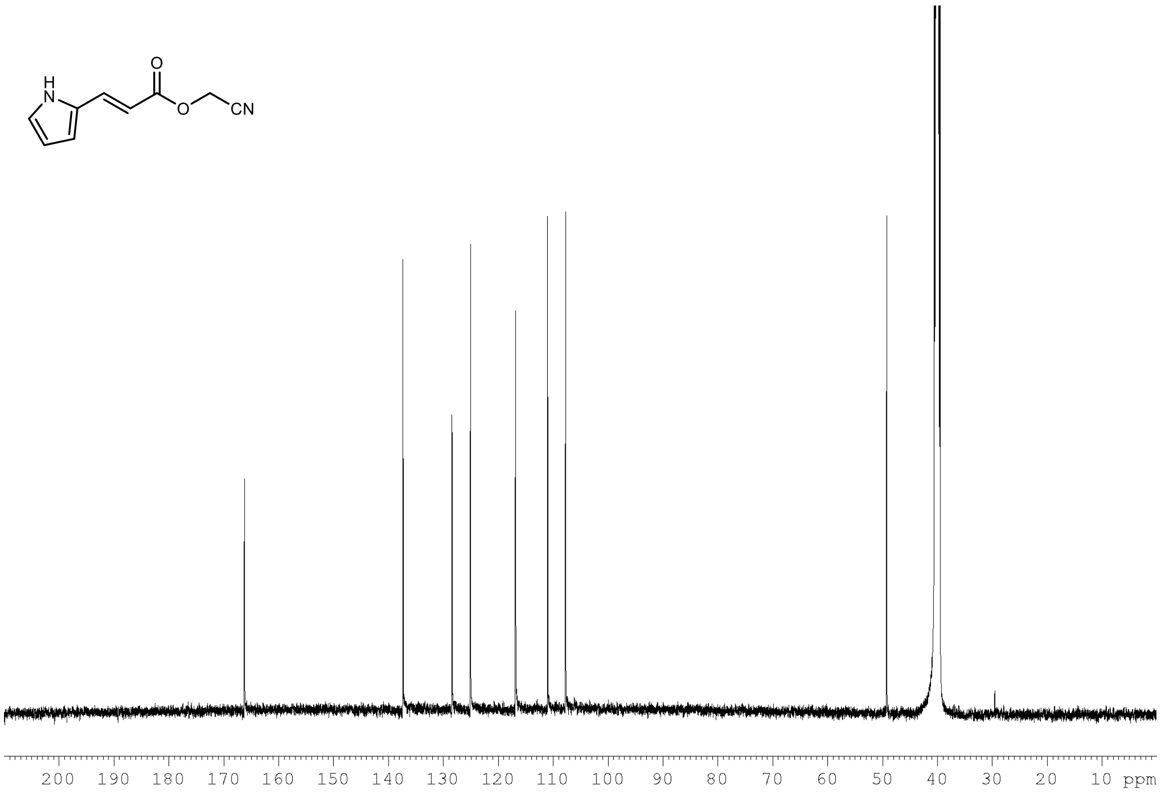

Supplementary Figure 41. ^{1}H NMR (500 MHz, $CDCl_3$) of **7.**

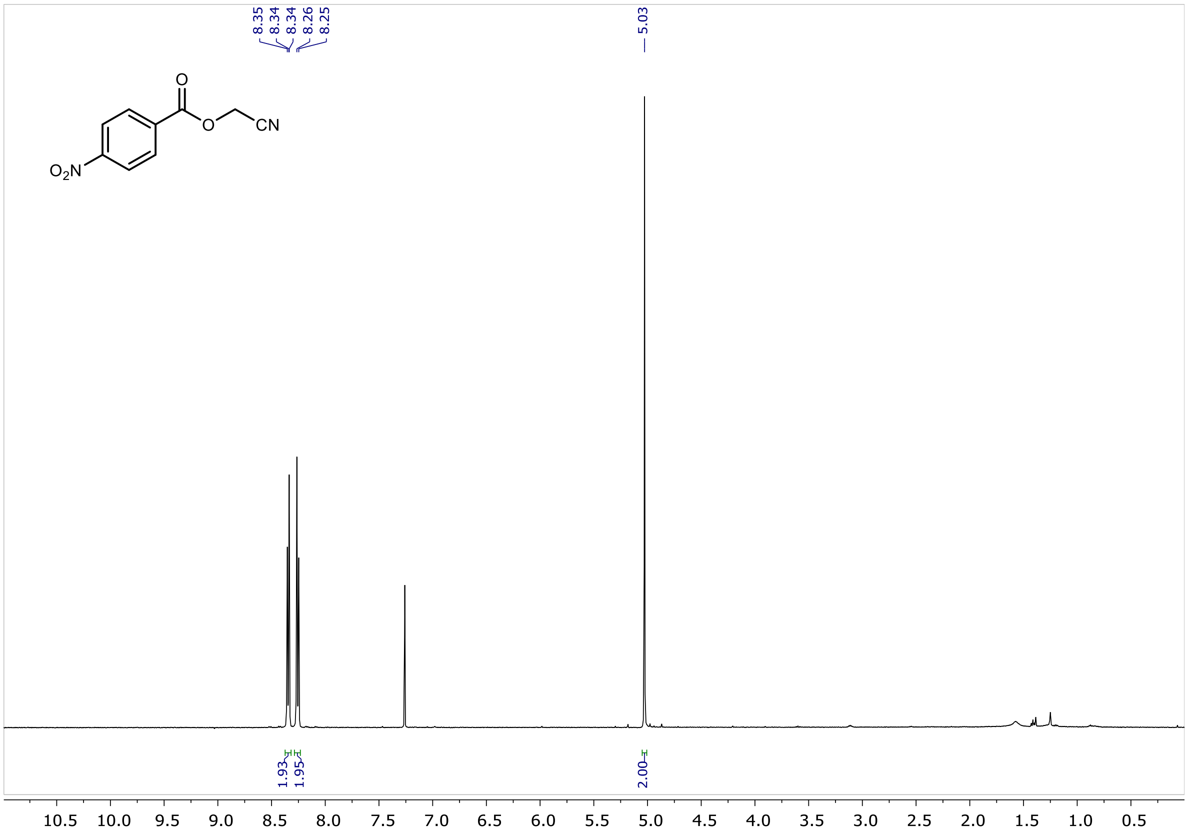

Supplementary Figure 42. ^{13}C NMR (125 MHz, $CDCl_3$) of **7.**

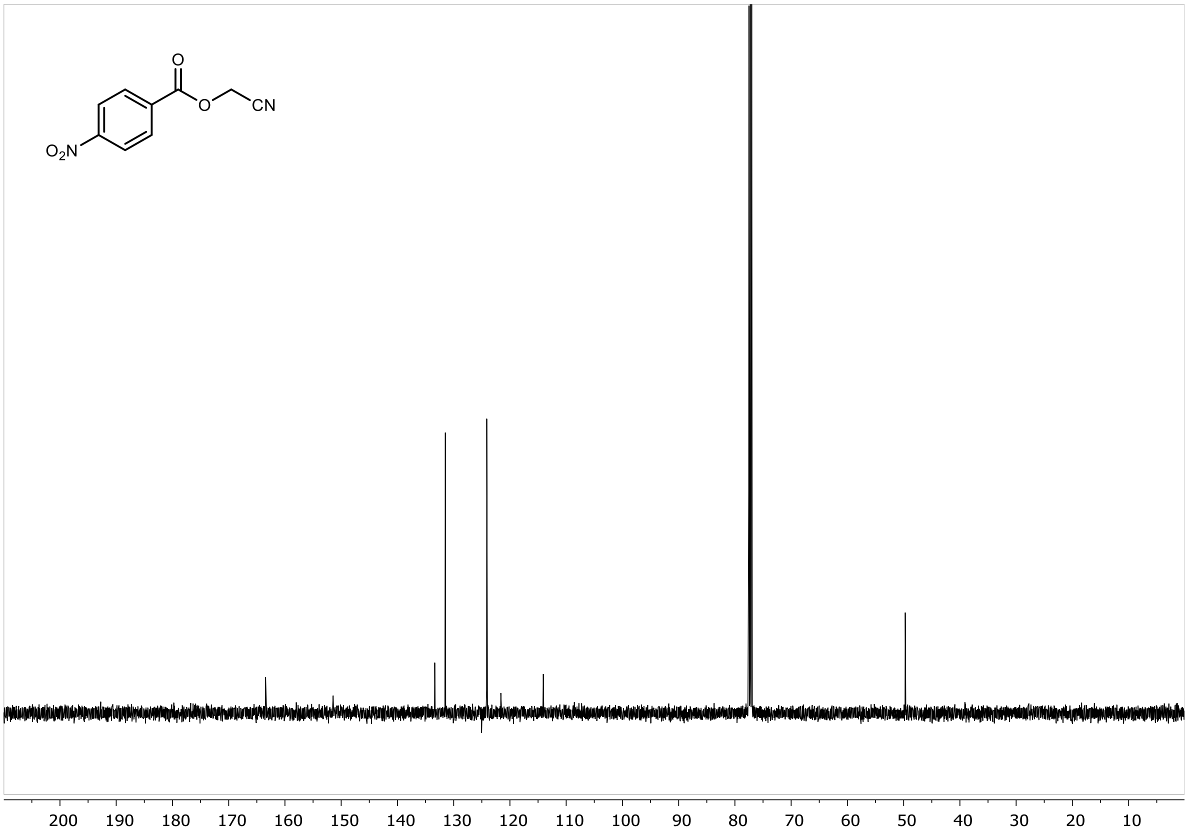

Supplementary Figure 43. ^{1}H NMR (500 MHz, $CDCl_3$) of **8.**

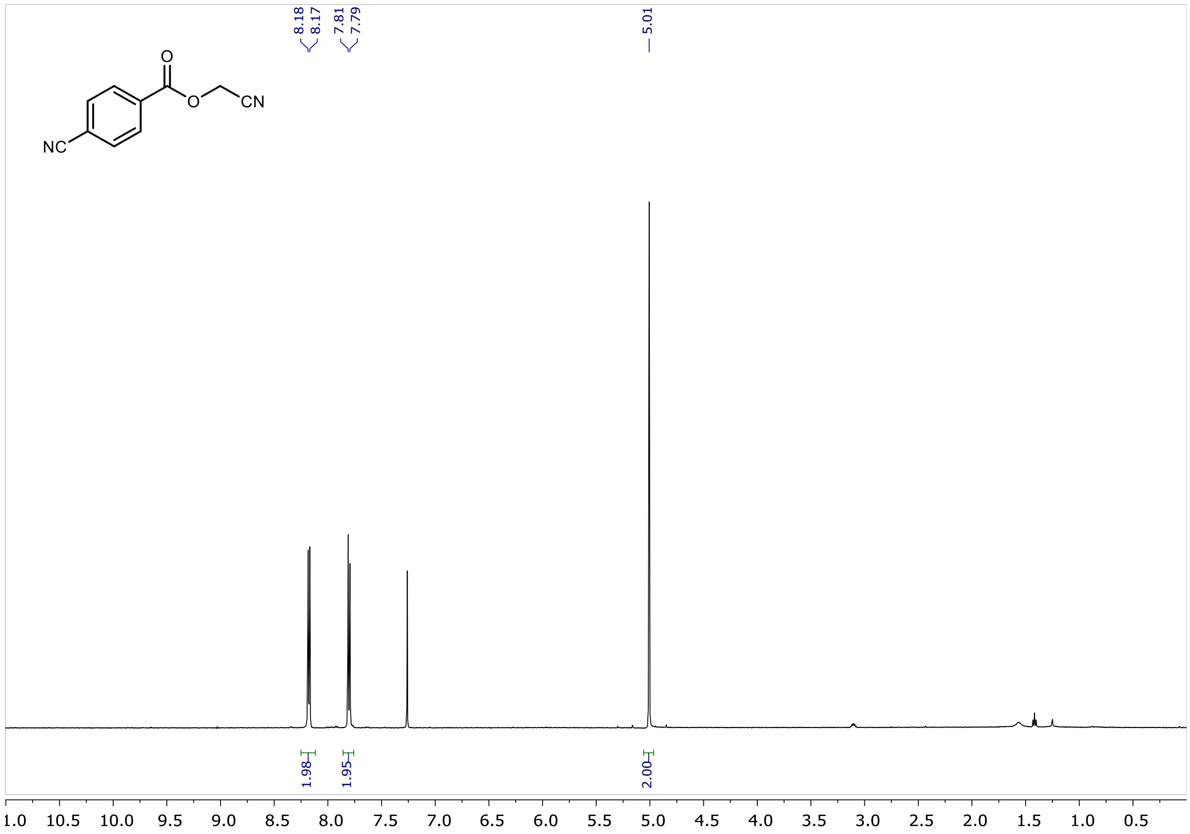

Supplementary Figure 44. ^{13}C NMR (125 MHz, $CDCl_3$) of **8.**

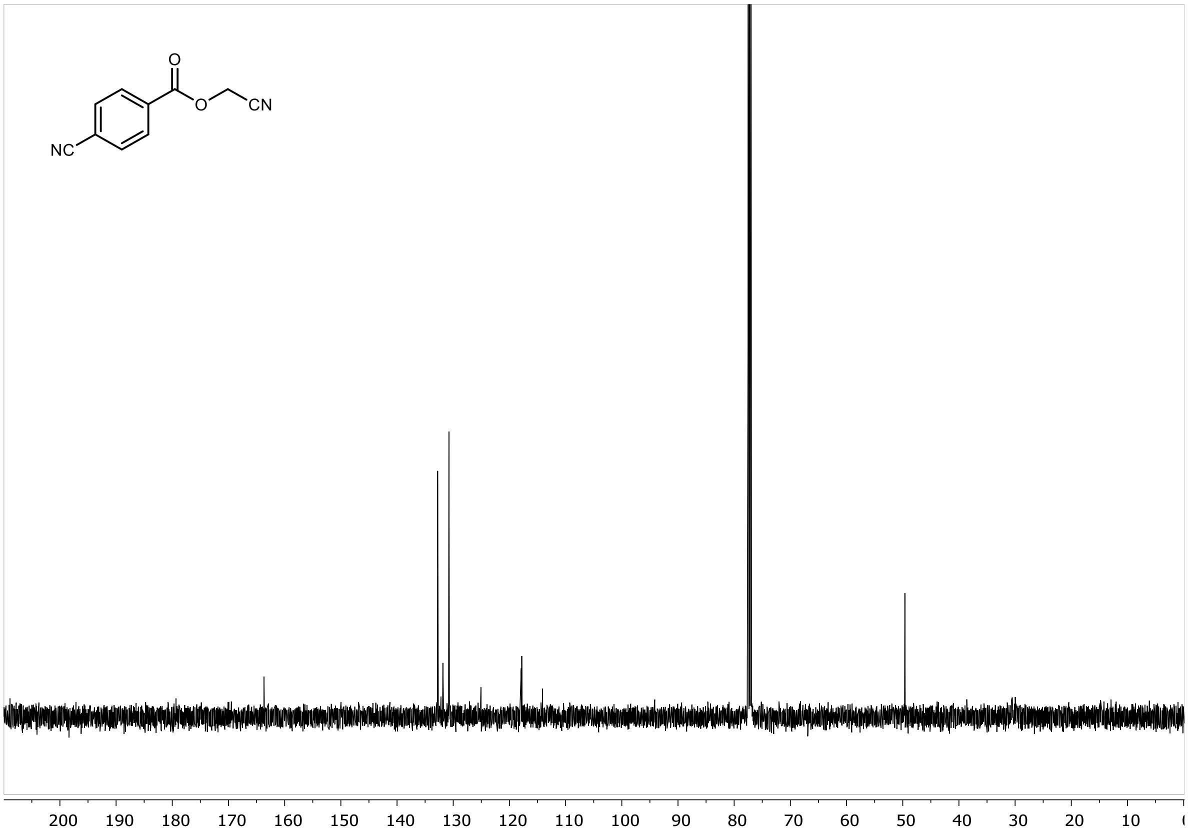

Supplementary Figure 45 ^{1}H NMR (500 MHz, CD_3CN) of **9.**

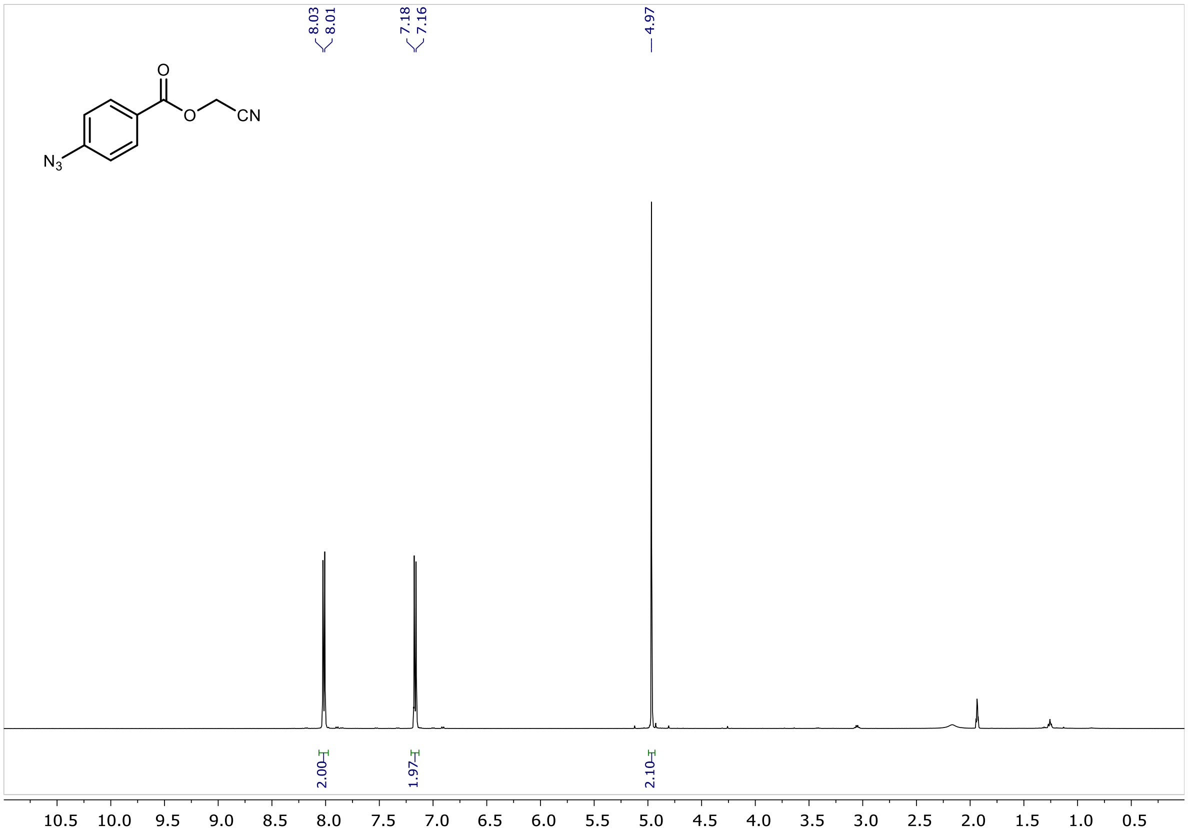

Supplementary Figure 46. ^{13}C NMR (125 MHz, CD_3CN) of **9.**

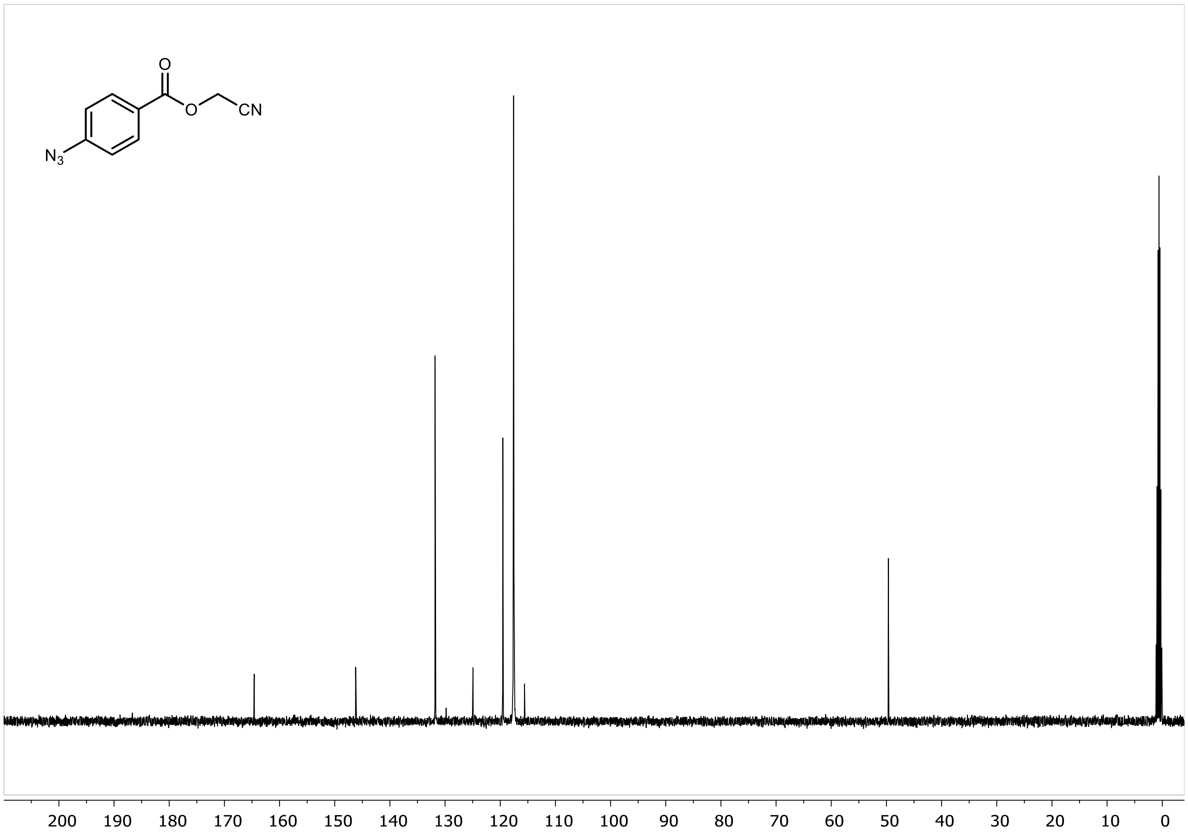

Supplementary Figure 47. ^{1}H NMR (500 MHz, $CDCl_3$) of **10.**

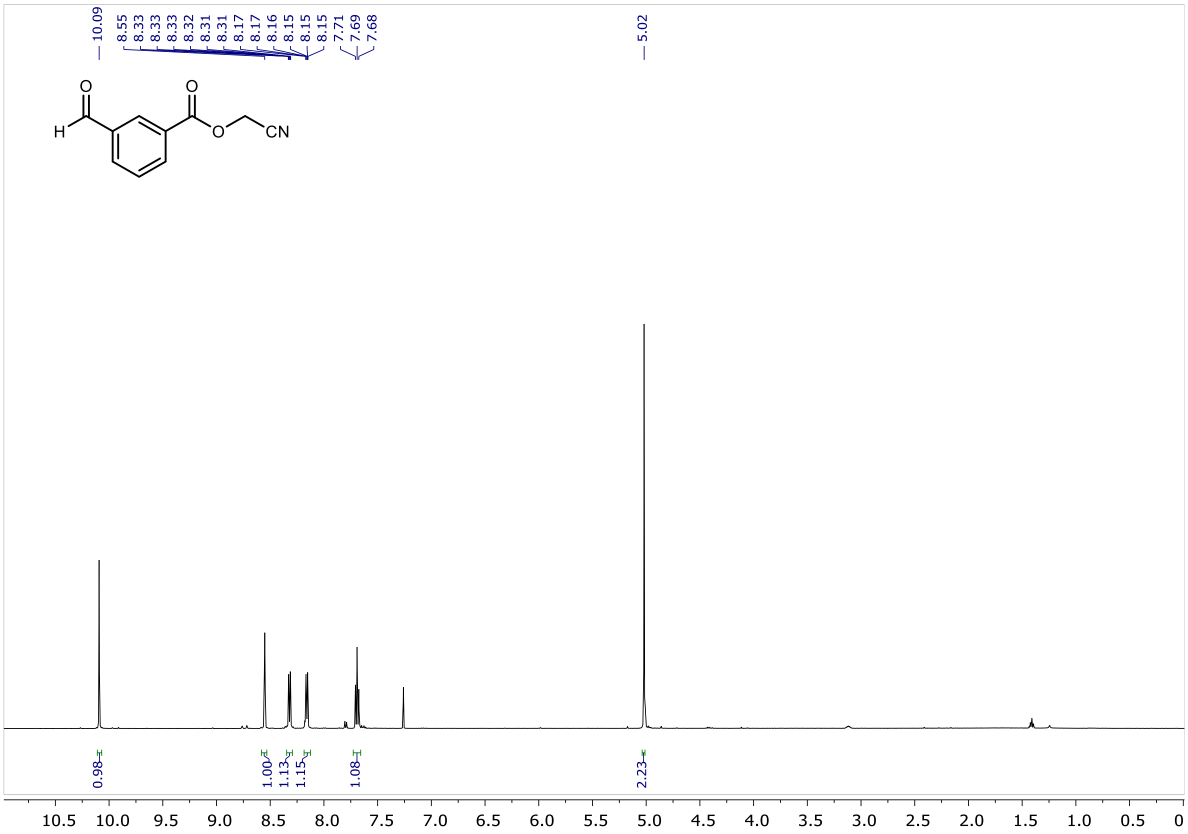

Supplementary Figure 48. ^{13}C NMR (125 MHz, $CDCl_3$) of **10.**

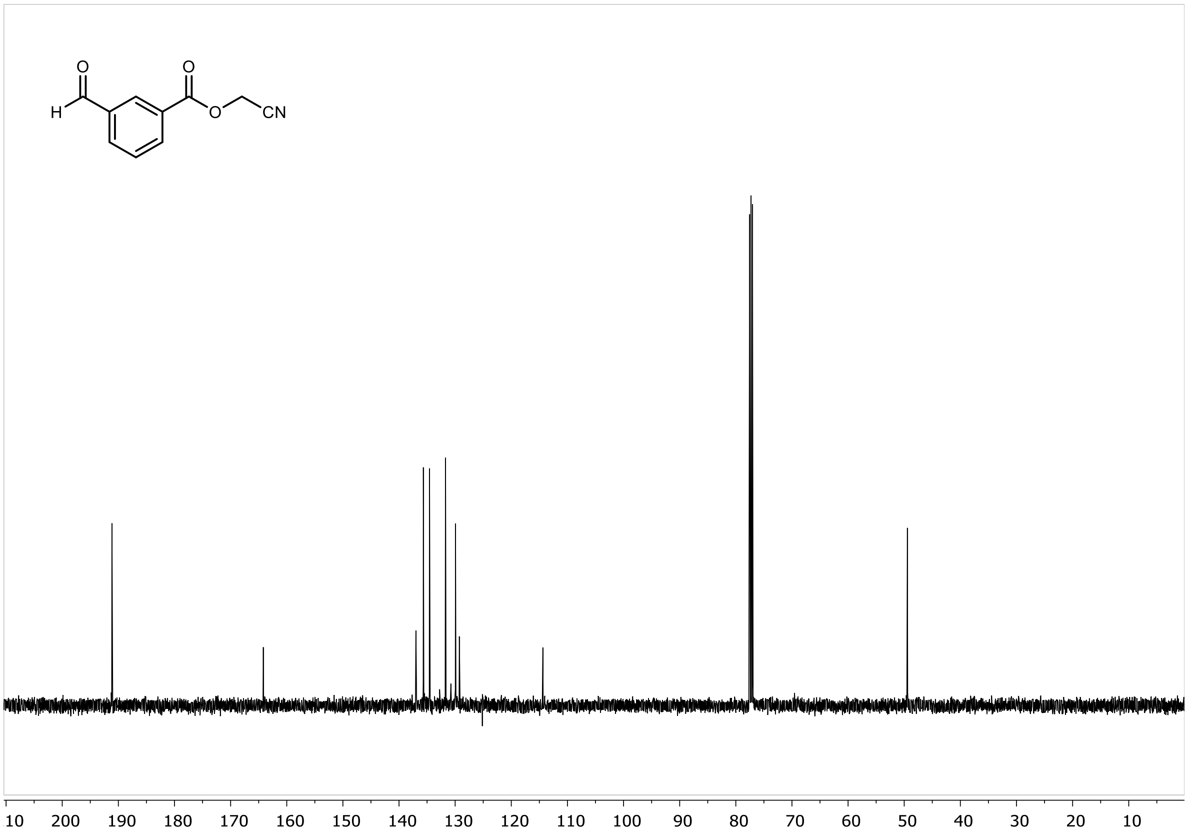

Supplementary Figure 49. ^{1}H NMR (500 MHz, $CDCl_3$) of **11.**

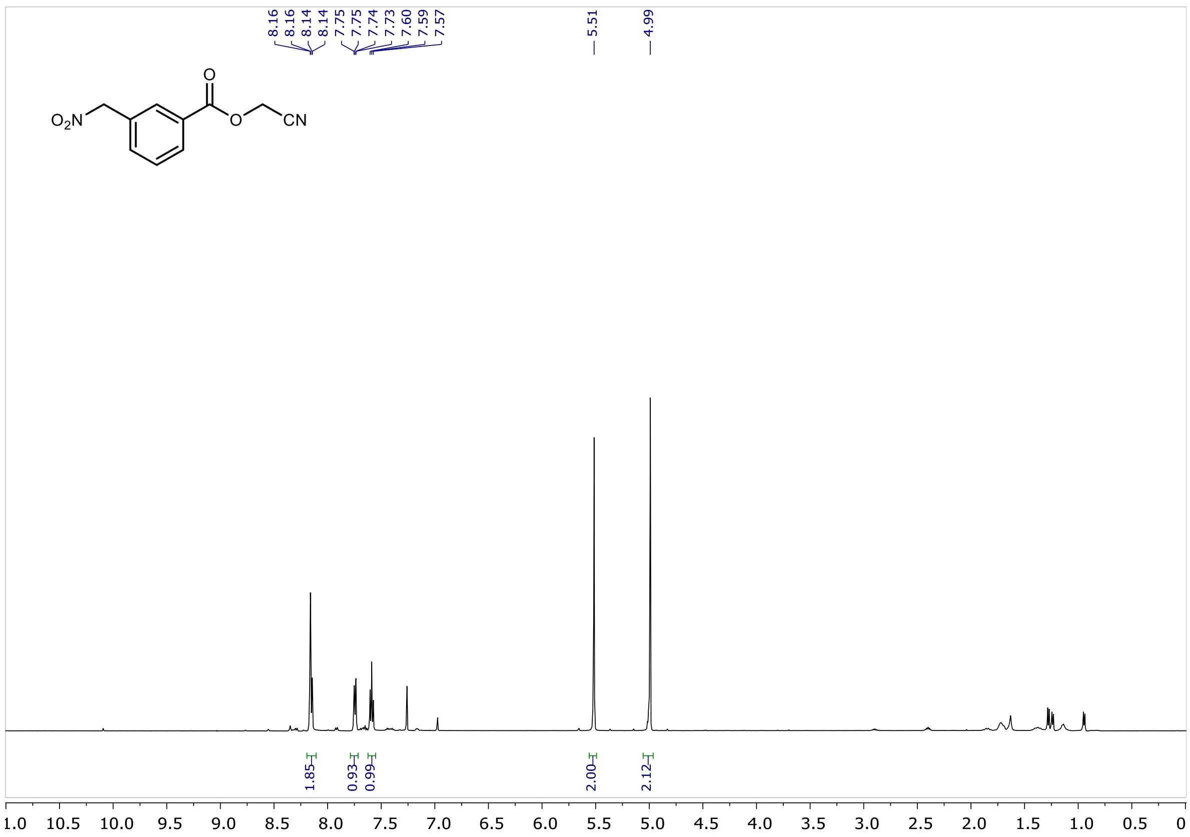

Supplementary Figure 50. ^{13}C NMR (125 MHz, $CDCl_3$) of **11.**

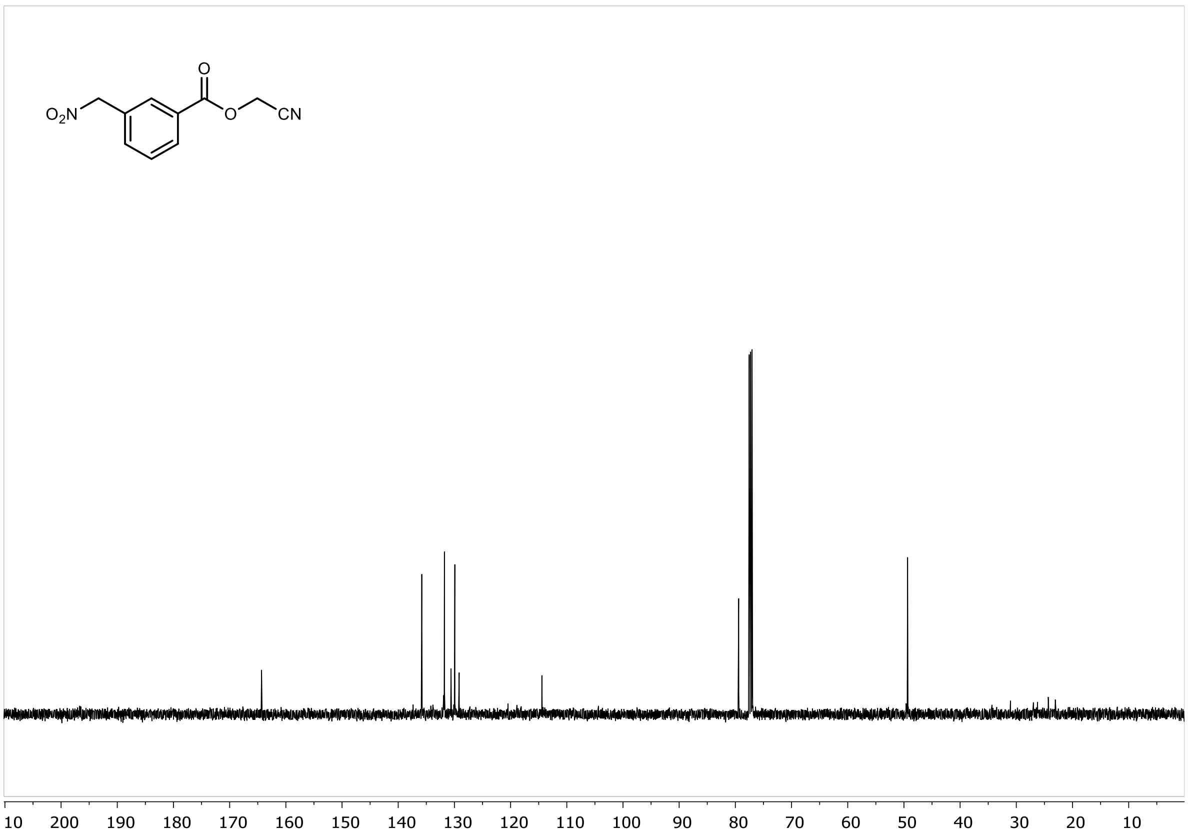

Supplementary Figure 51. ^{1}H NMR (500 MHz, $CDCl_3$) of **12.**

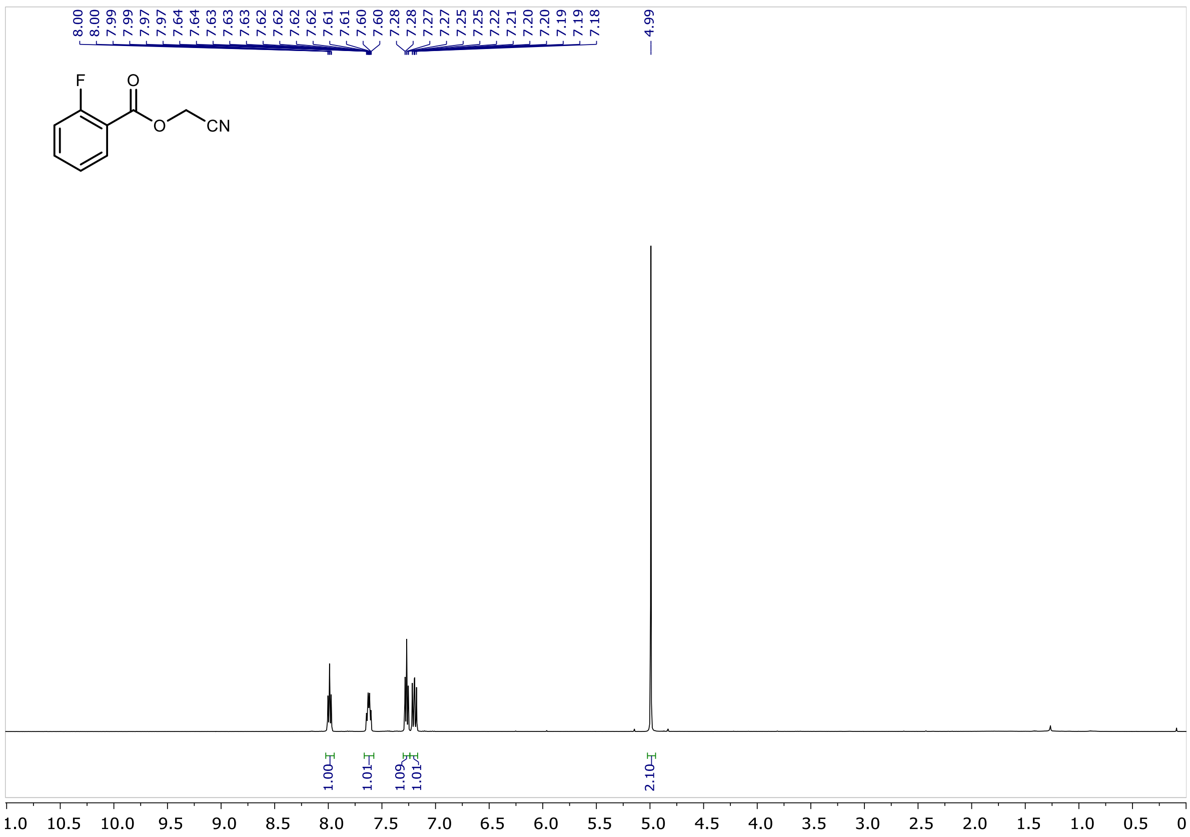

Supplementary Figure 52. ^{13}C NMR (125 MHz, $CDCl_3$) of **12.**

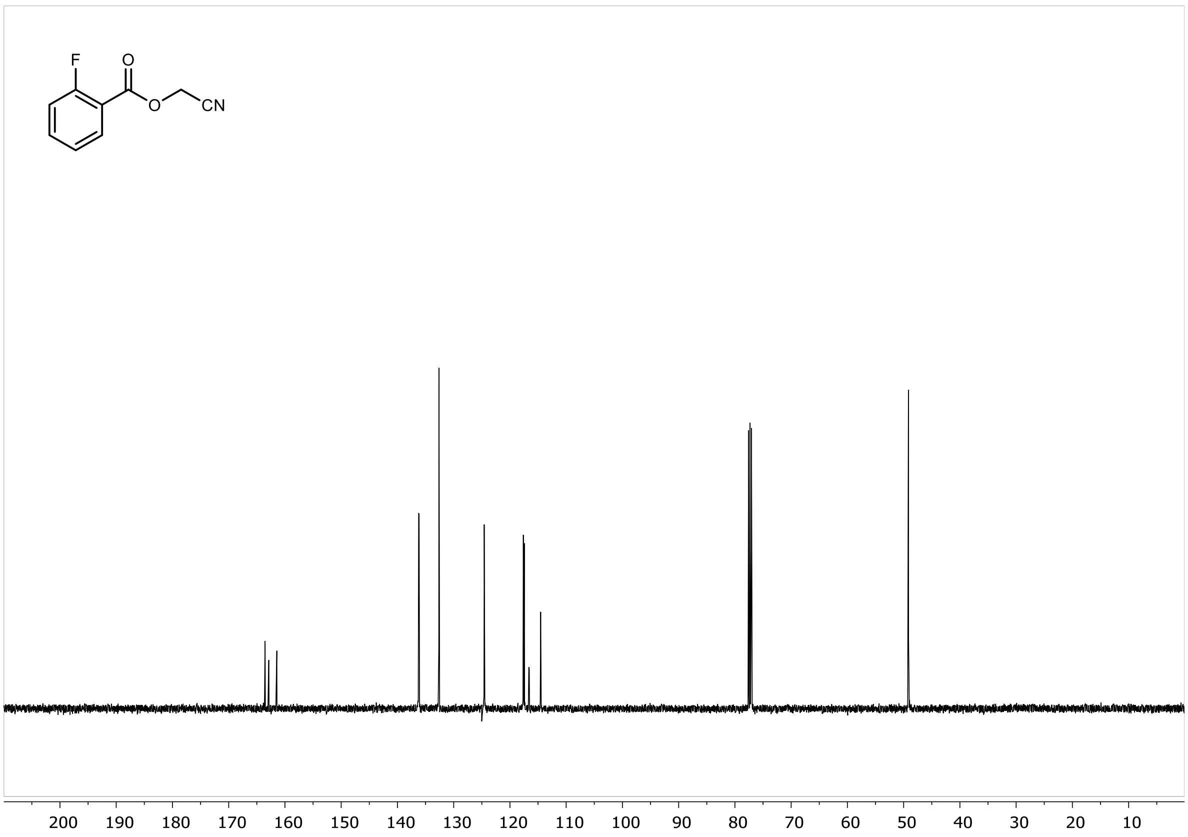

Supplementary Figure 53. ^{1}H NMR (500 MHz, $CDCl_3$) of **13.**

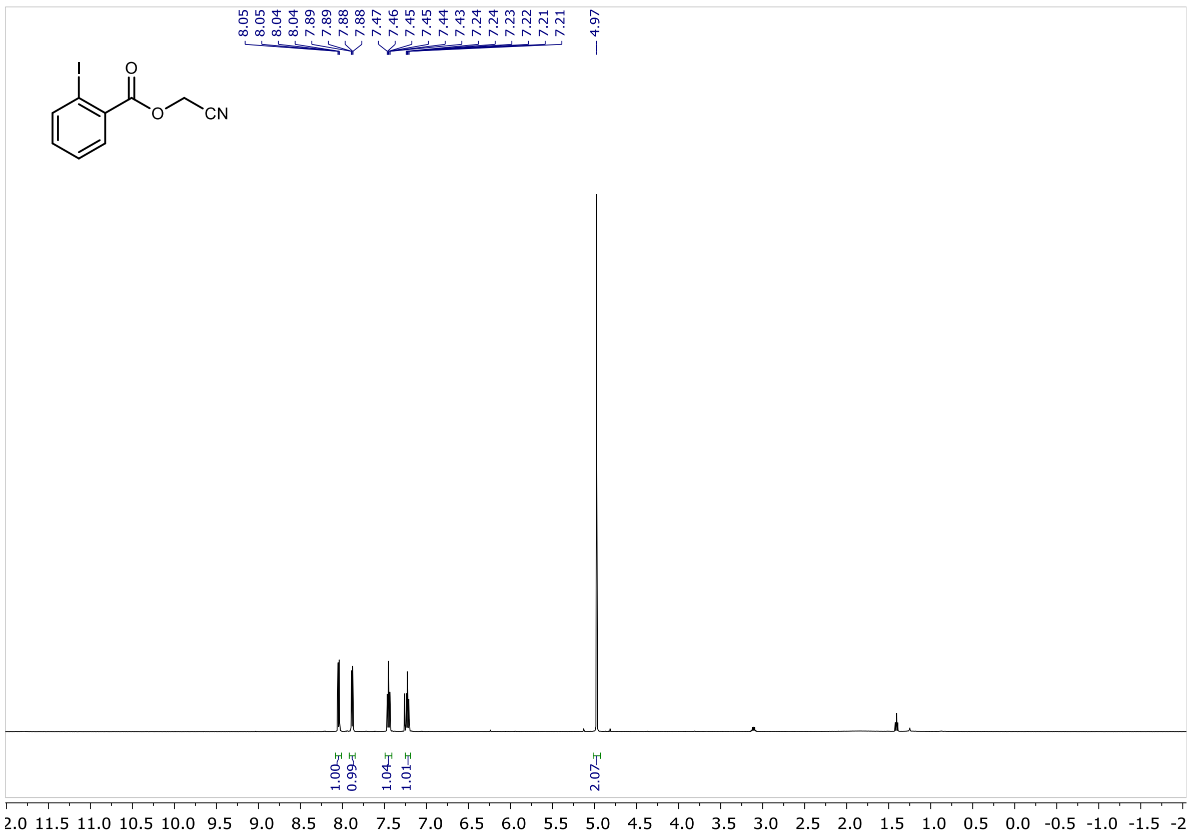

Supplementary Figure 54. ^{13}C NMR (125 MHz, $CDCl_3$) of **13.**

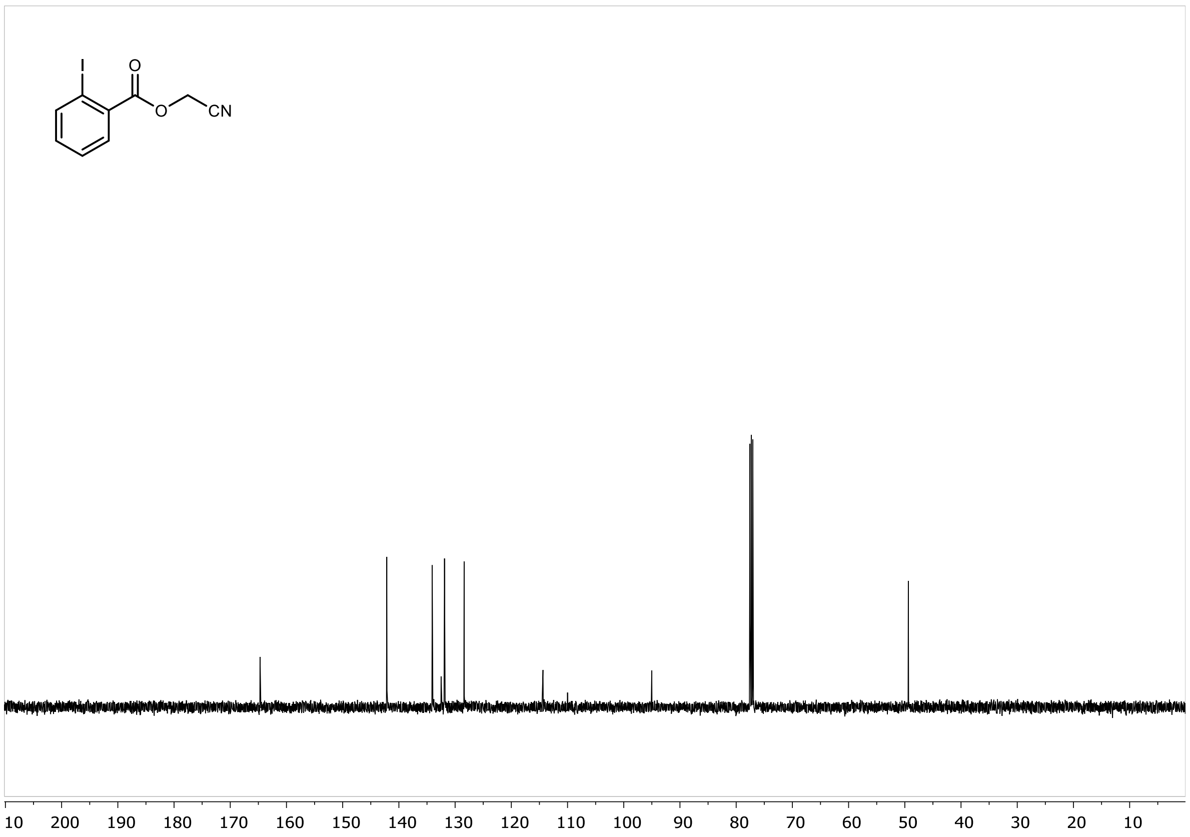

Supplementary Figure 55. ^{1}H NMR (500 MHz, $CDCl_3$) of **14.**

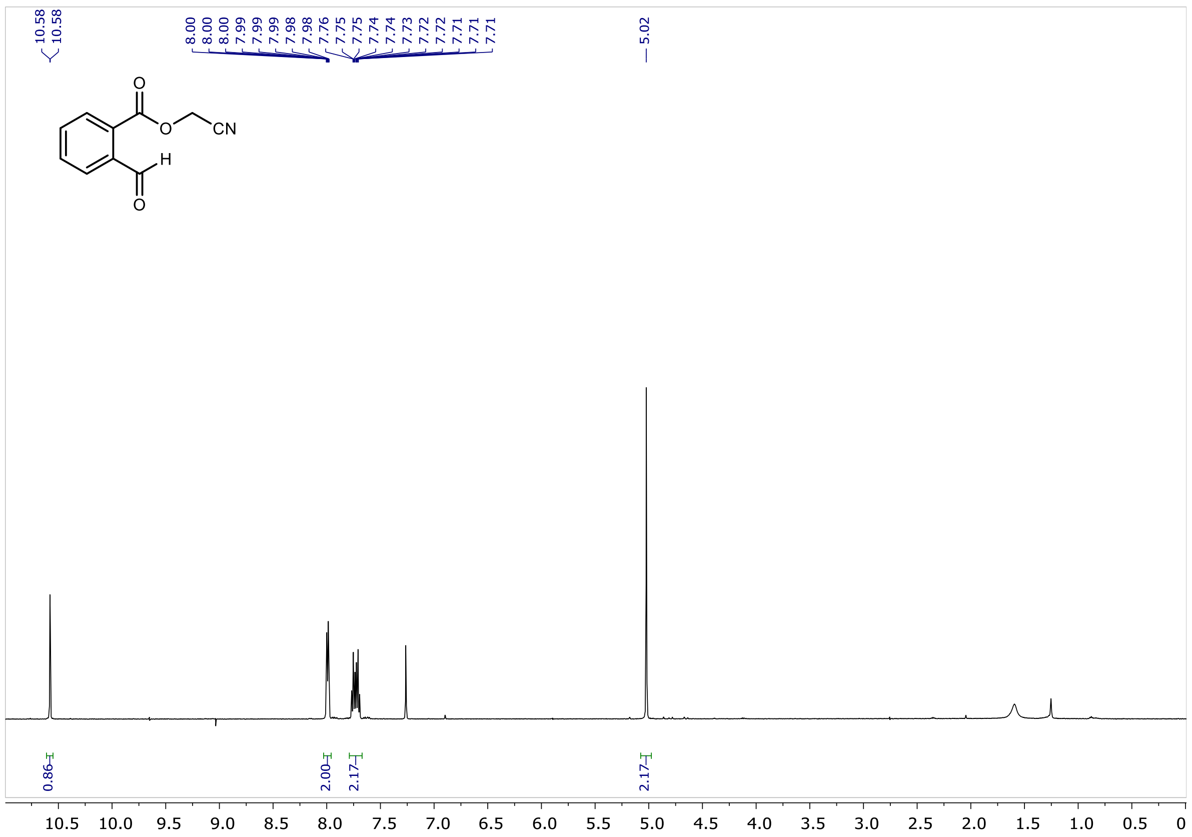

Supplementary Figure 56. ^{13}C NMR (125 MHz, $CDCl_3$) of **14.**

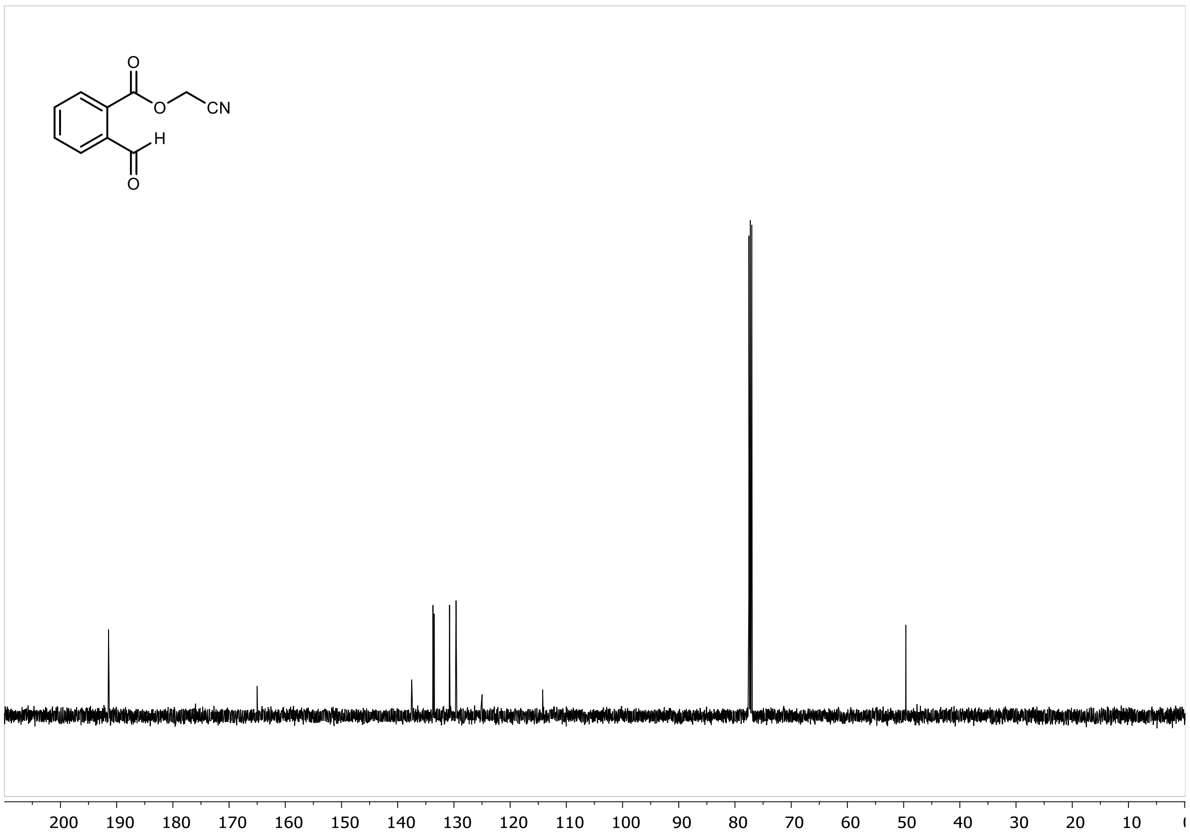

Supplementary Figure 57. ^{1}H NMR (500 MHz, $CDCl_3$) of **15.**

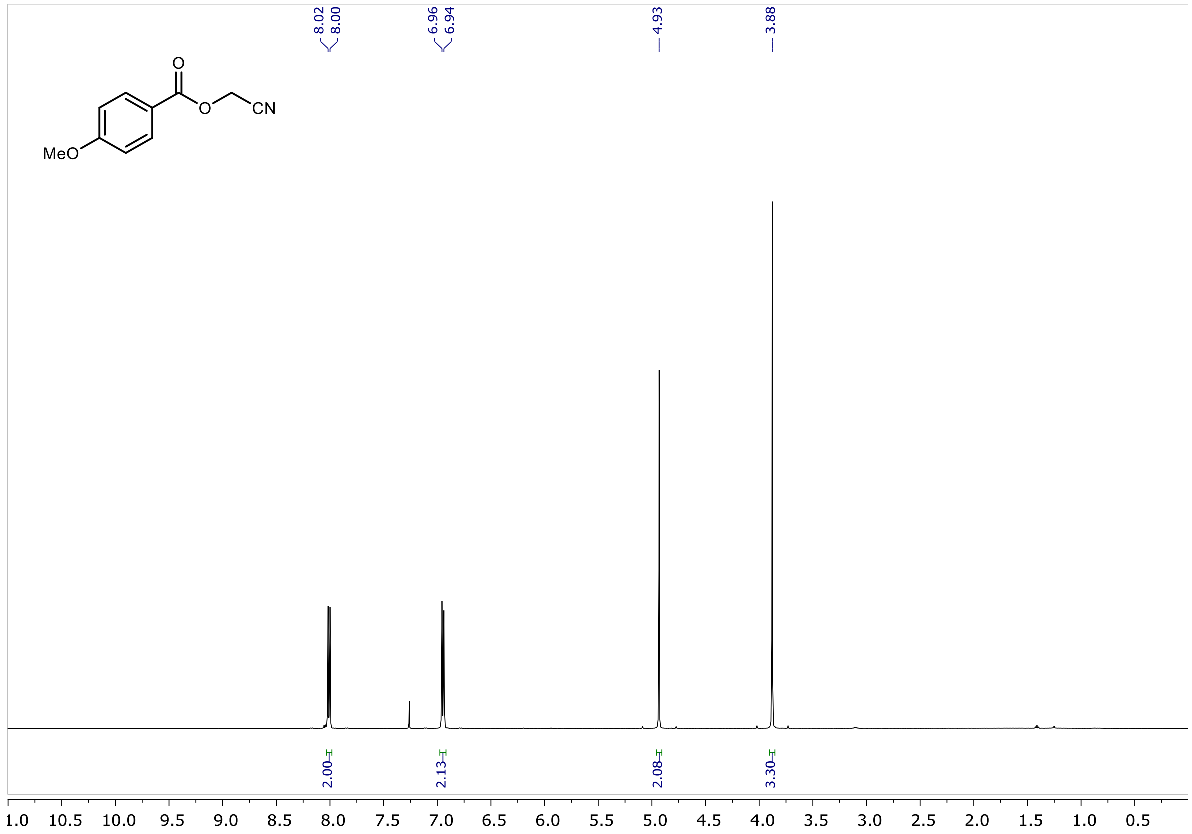

Supplementary Figure 58 ^{13}C NMR (125 MHz, $CDCl_3$) of **15.**

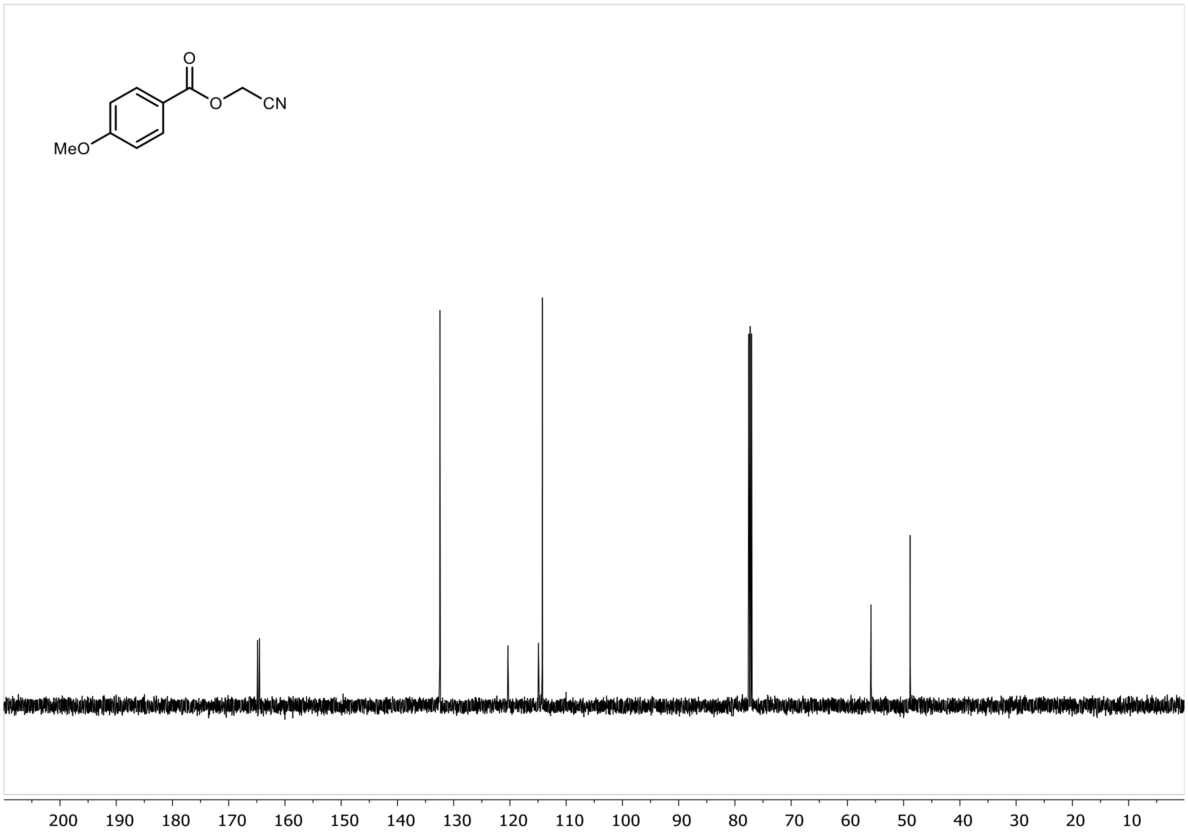

Supplementary Figure 59. ^{1}H NMR (500 MHz, $CDCl_3$) of **16.**

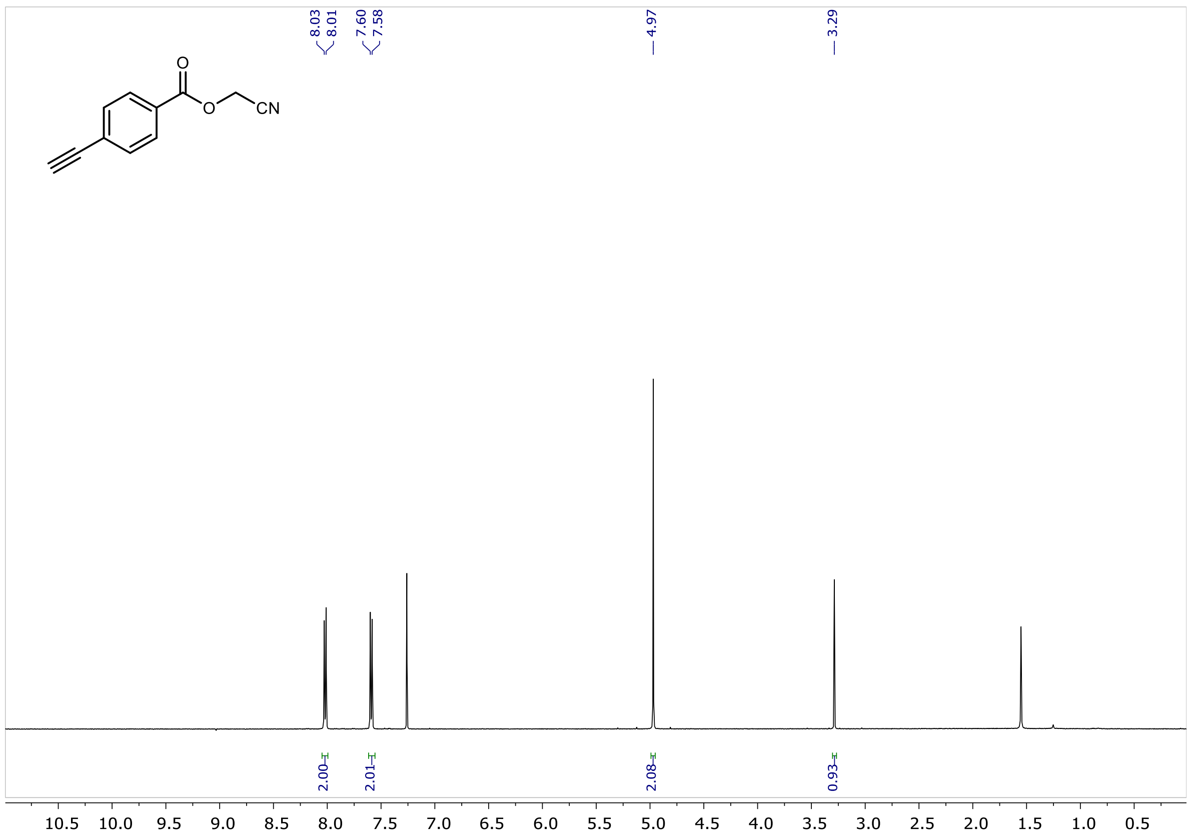

Supplementary Figure 60. ^{13}C NMR (125 MHz, $CDCl_3$) of **16.**

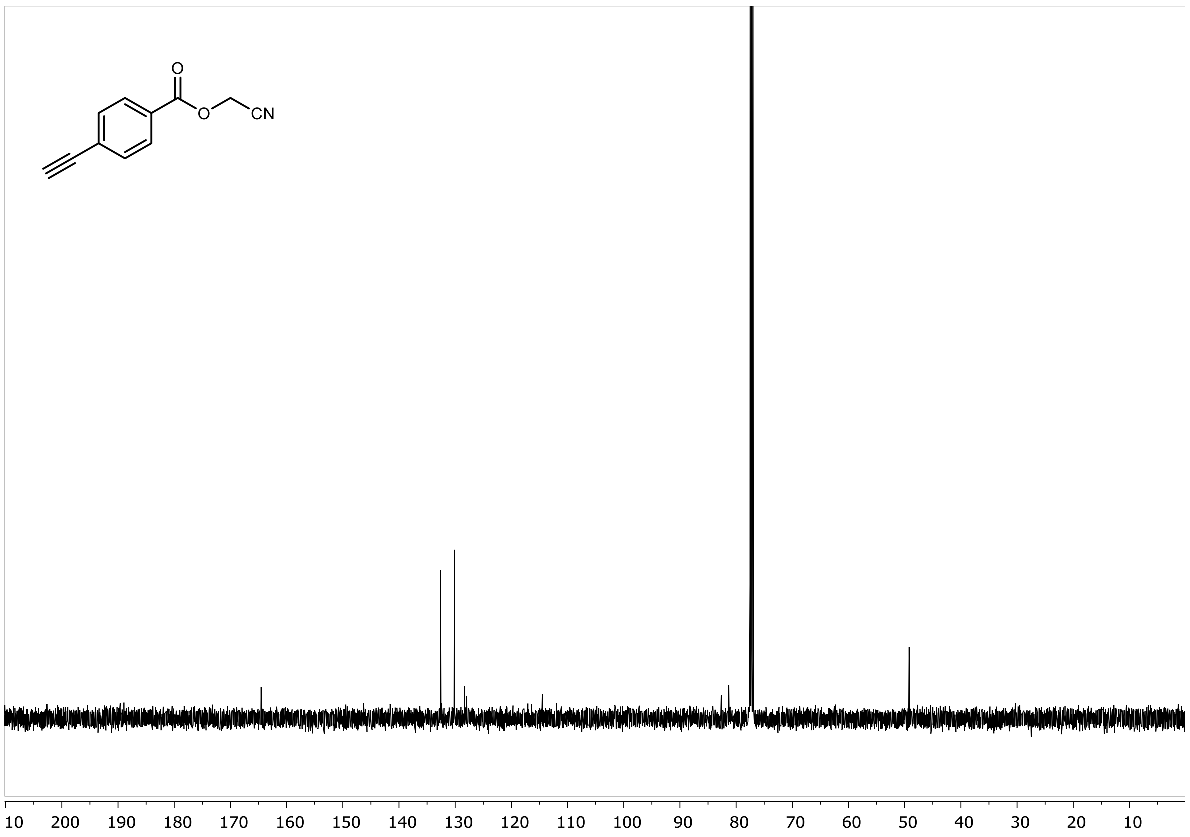

Supplementary Figure 61. ^{1}H NMR (500 MHz, $CDCl_3$) of **17.**

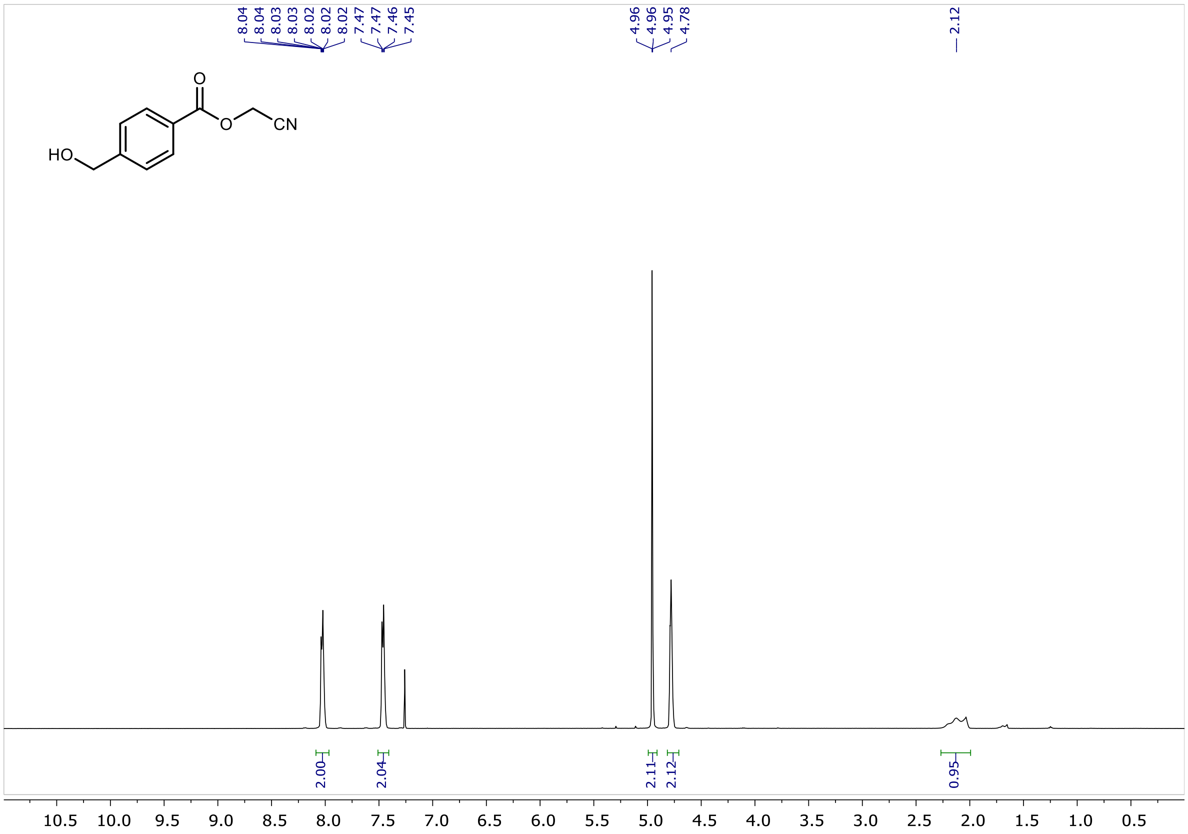

Supplementary Figure 62. ^{13}C NMR (125 MHz, $CDCl_3$) of **17.**

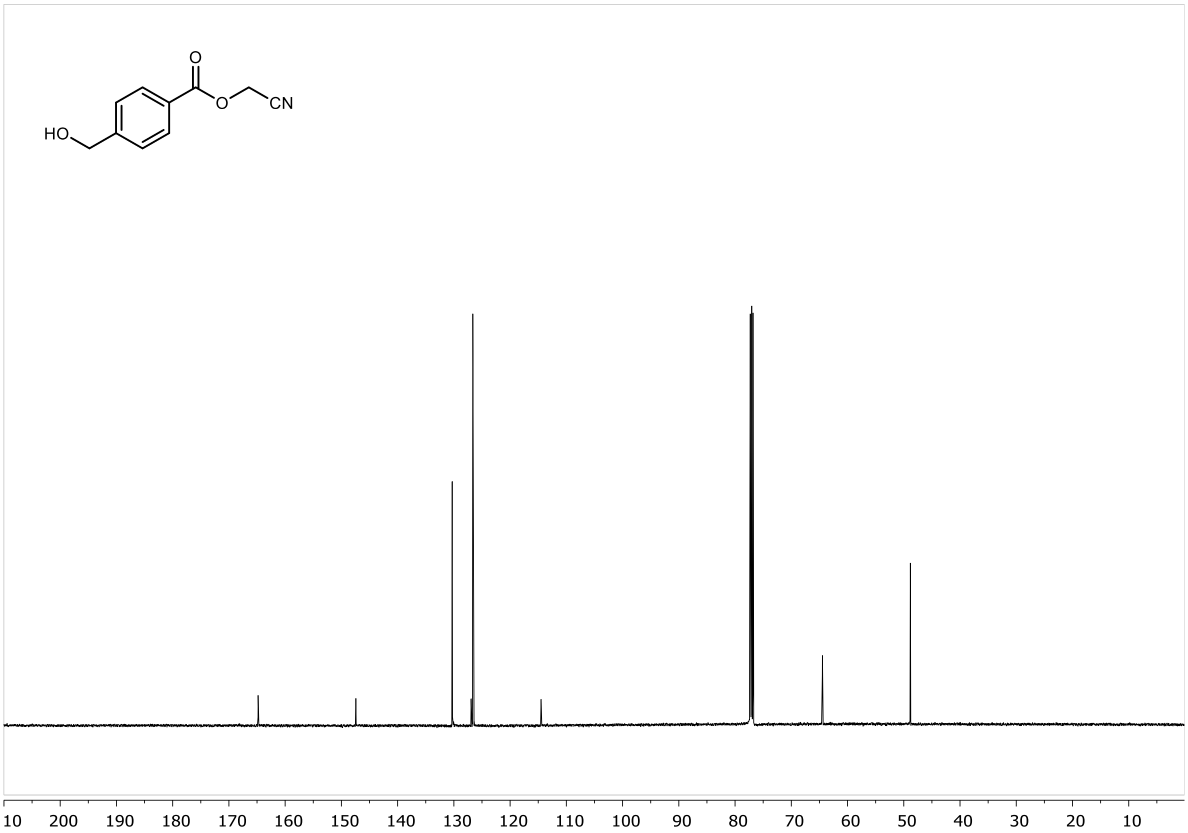

Supplementary Figure 63. ^{1}H NMR (500 MHz, DMSO-d_6) of **18.**

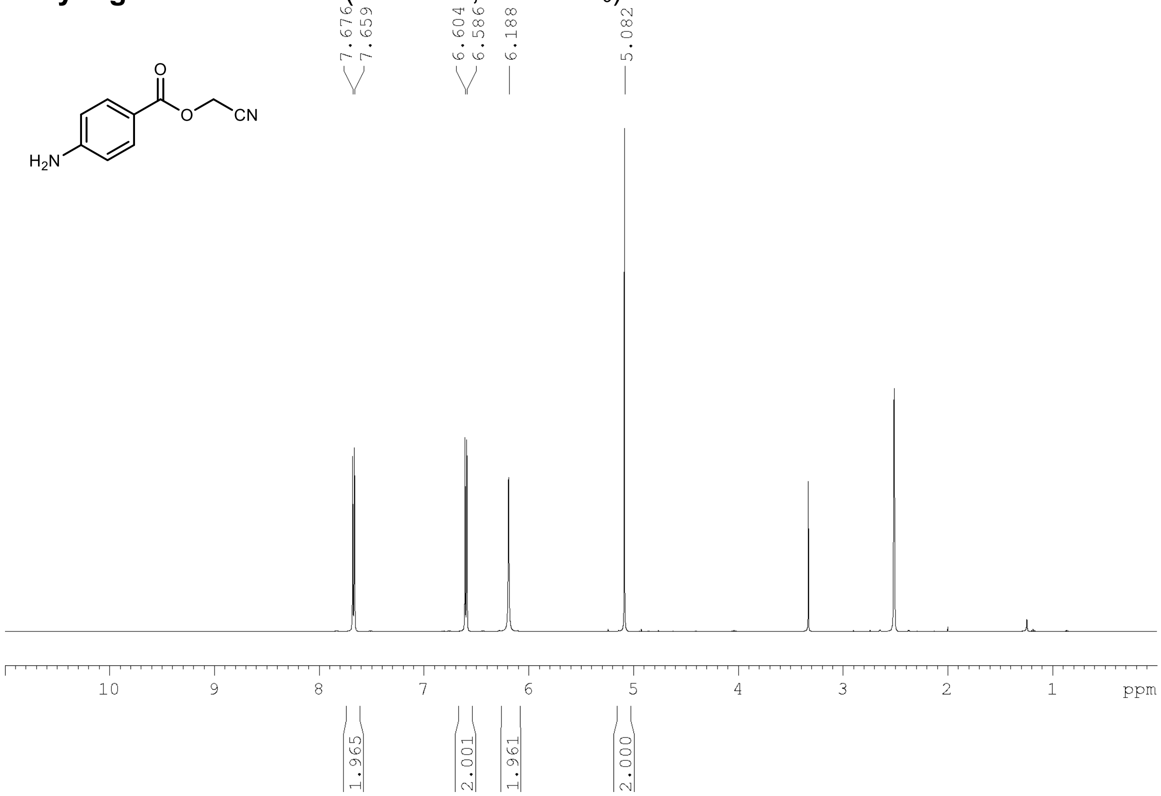

Supplementary Figure 64. ^{13}C NMR (125 MHz, DMSO-d_6) of **18.**

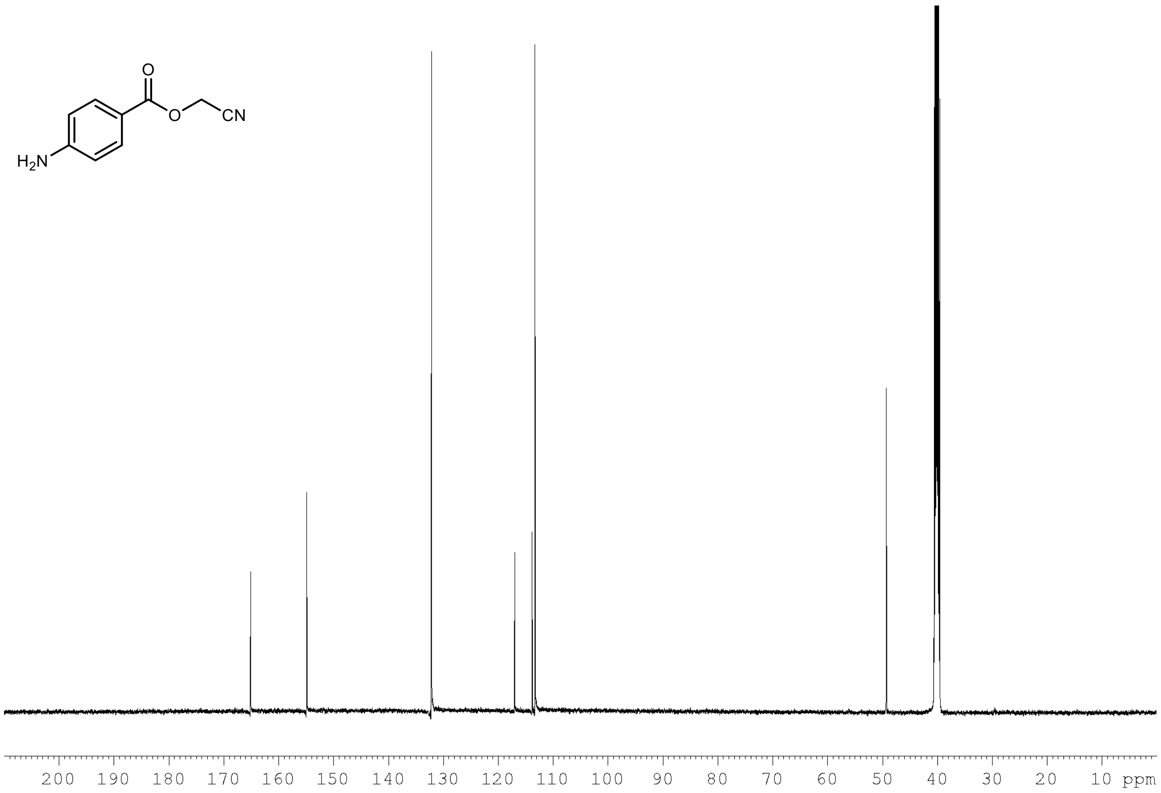

Supplementary Figure 65. ^{1}H NMR (500 MHz, $CDCl_3$) of **19.**

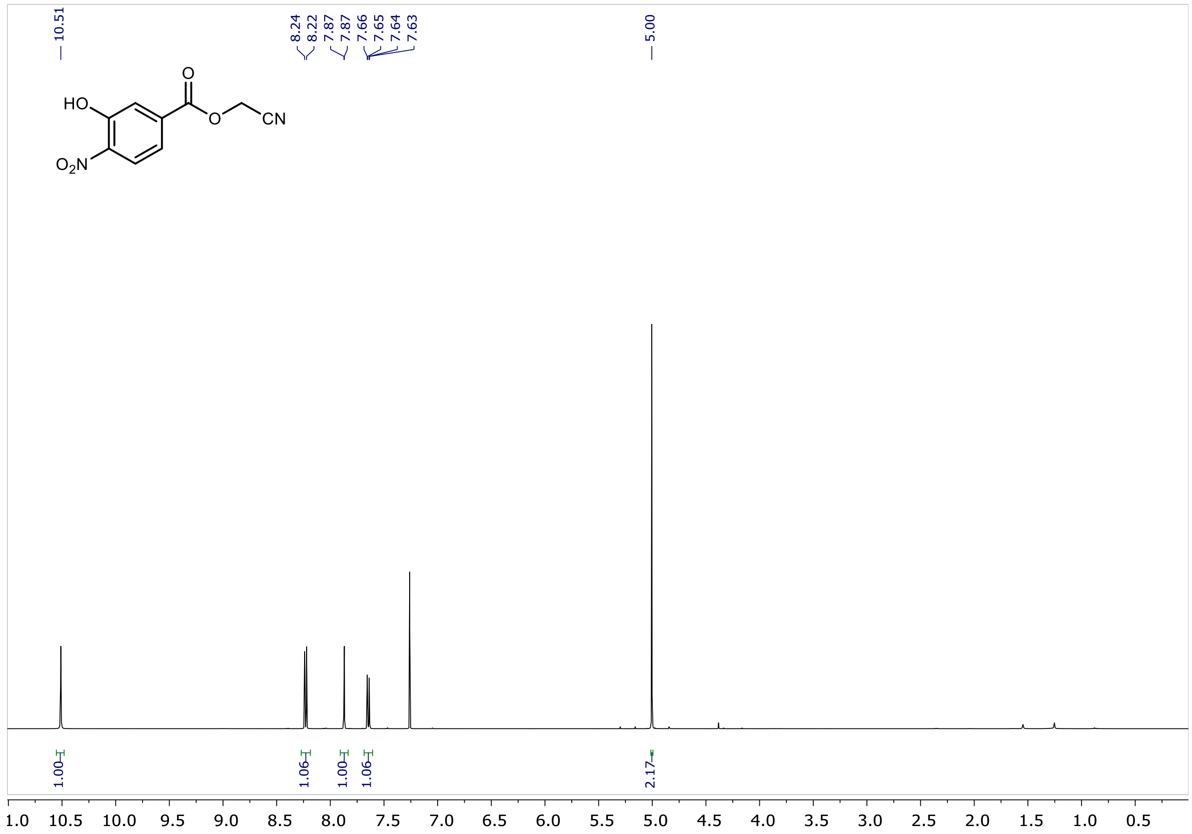

Supplementary Figure 66. ^{13}C NMR (125 MHz, $CDCl_3$) of **19.**

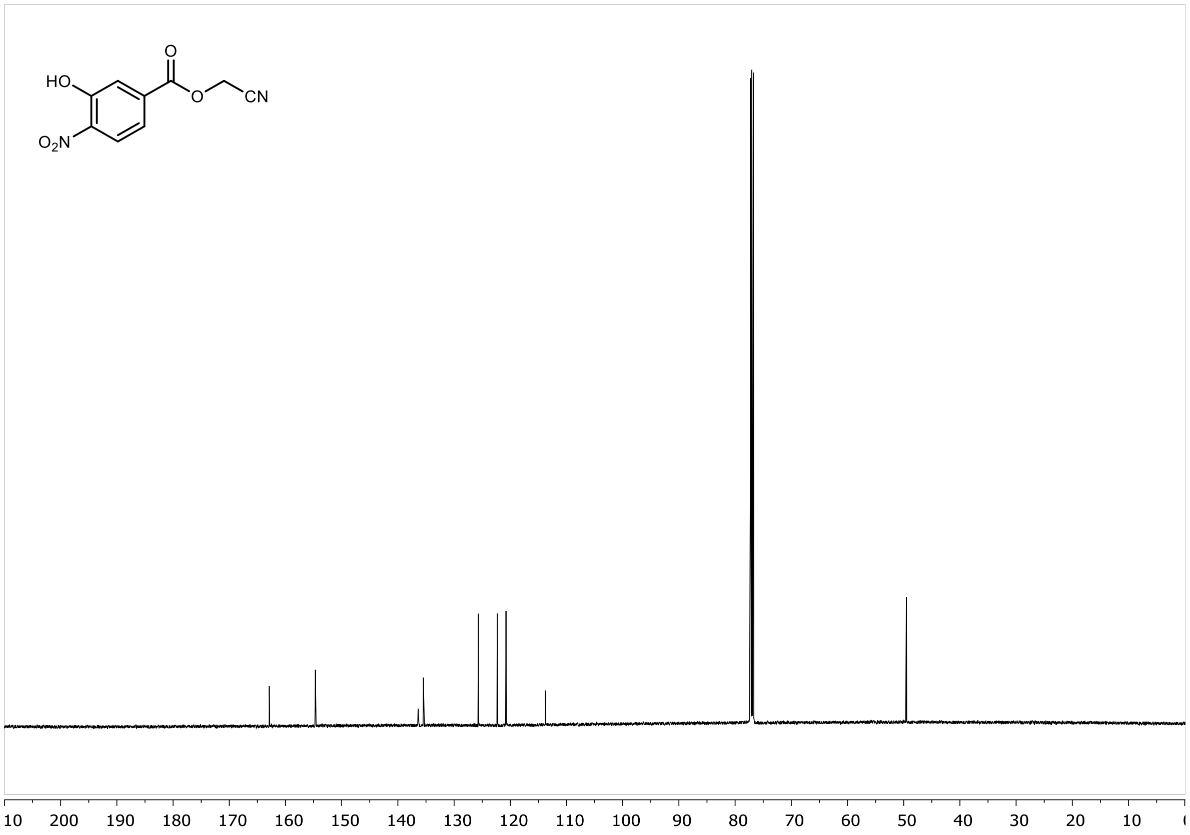

Supplementary Figure 67. ^{1}H NMR (500 MHz, DMSO-d_6) of **20.**

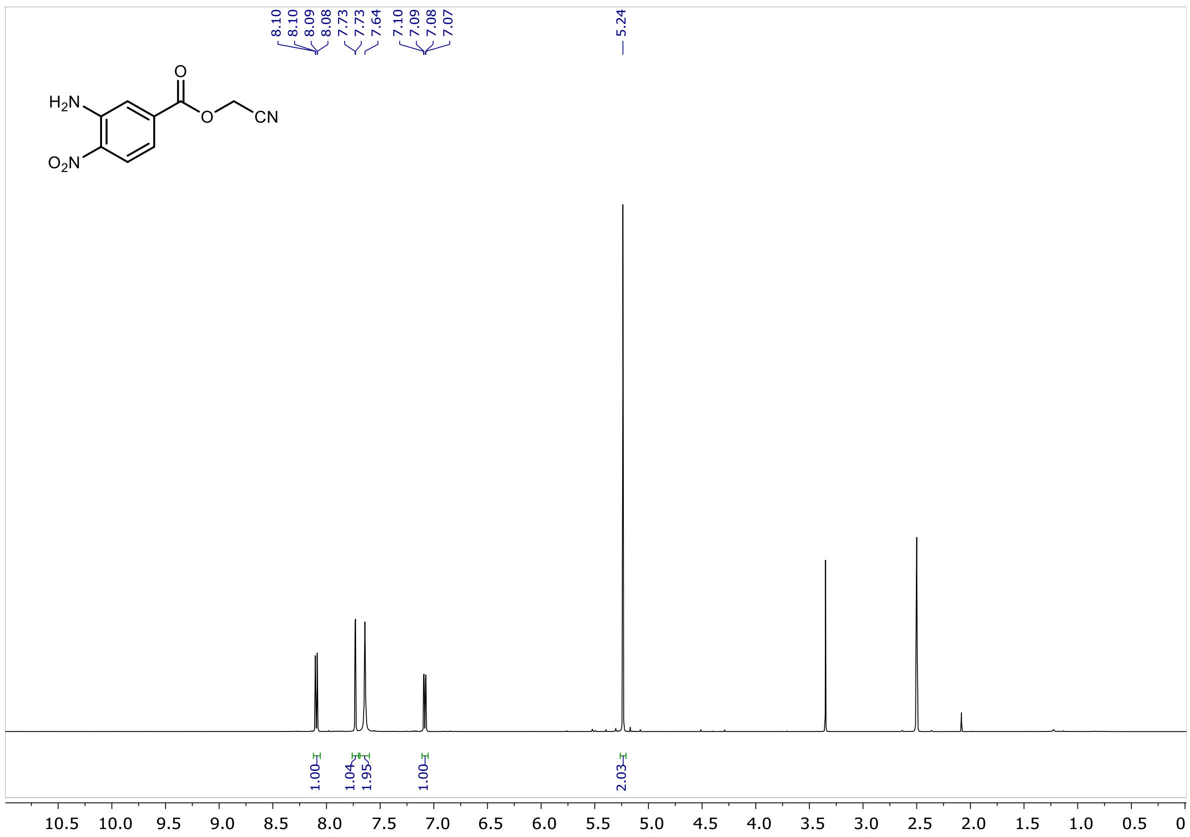

Supplementary Figure 68. ^{13}C NMR (125 MHz, DMSO-d_6) of **20.**

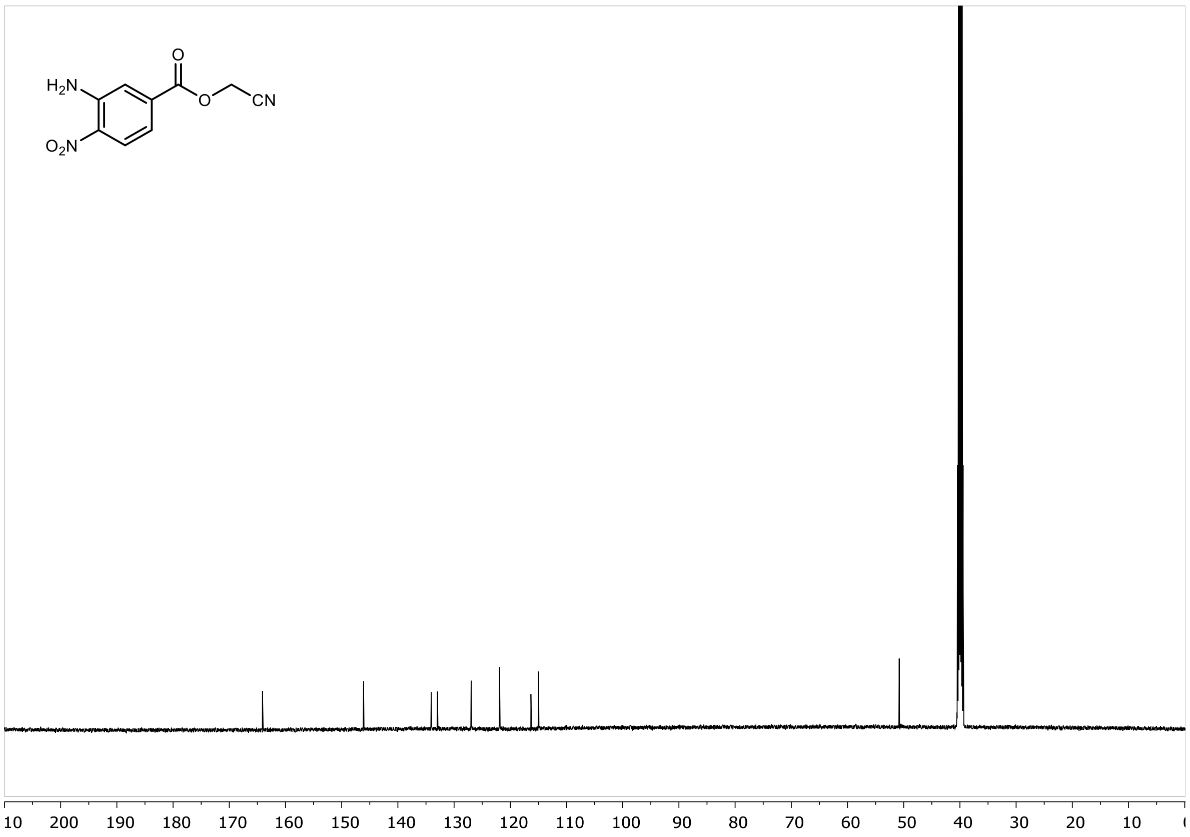

Supplementary Figure 69. ^{1}H NMR (500 MHz, acetone-d_6) of **21.**

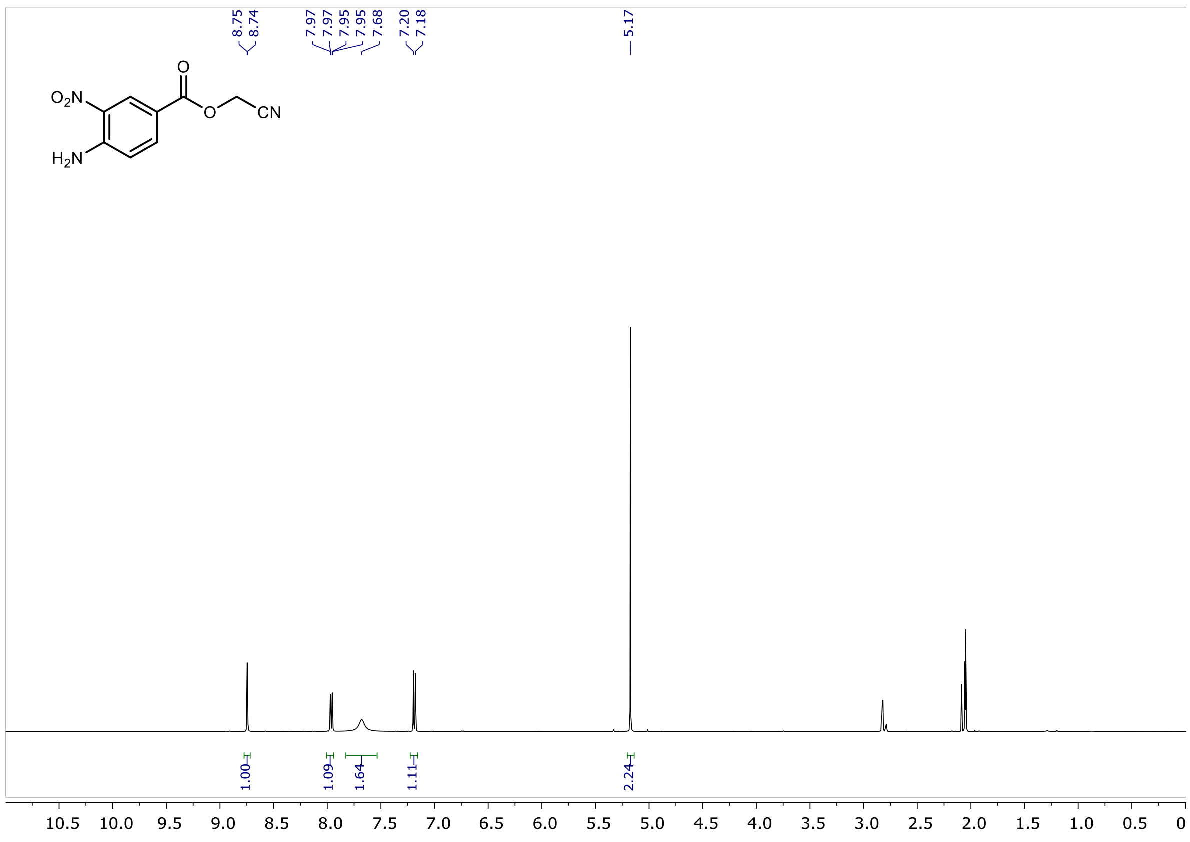

Supplementary Figure 70. ^{13}C NMR (125 MHz, acetone-d_6) of **21.**

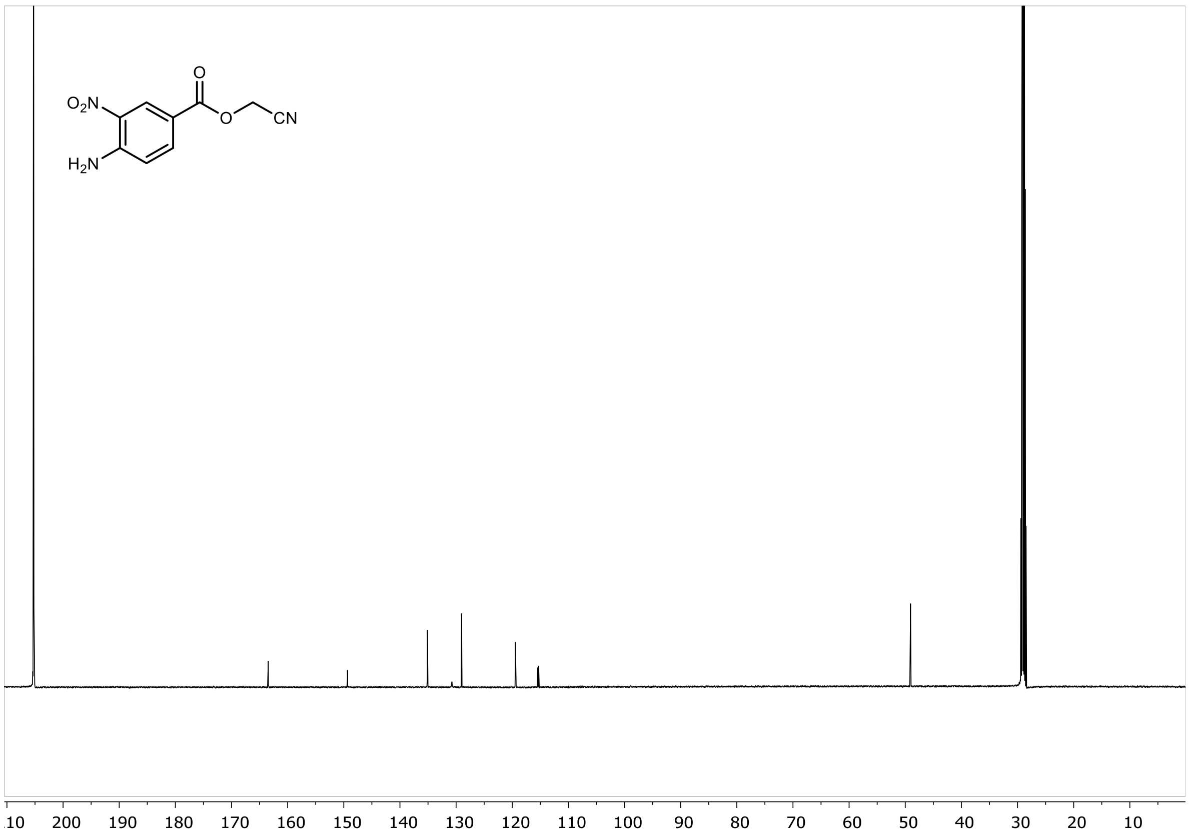

Supplementary Figure 71. ^{1}H NMR (500 MHz, $CDCl_3$) of **22.**

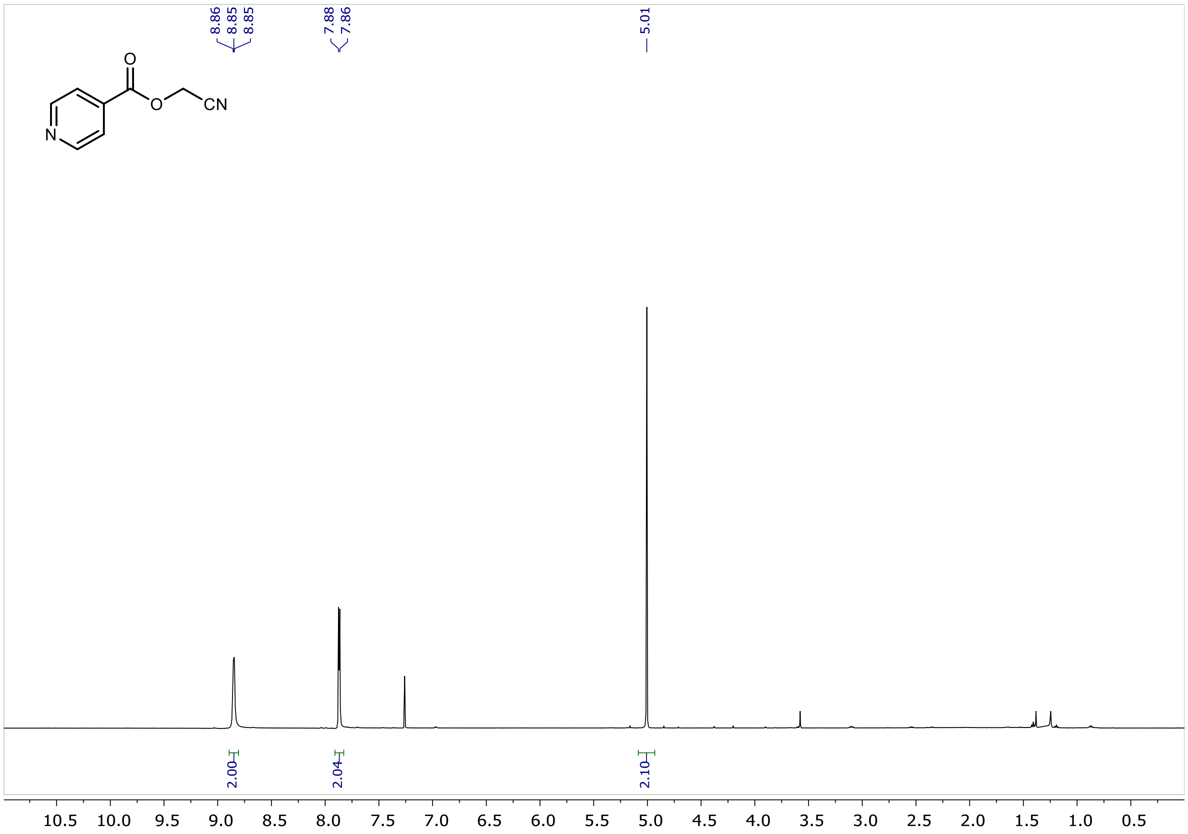

Supplementary Figure 3. ^{13}C NMR (125 MHz, $CDCl_3$) of **22.**

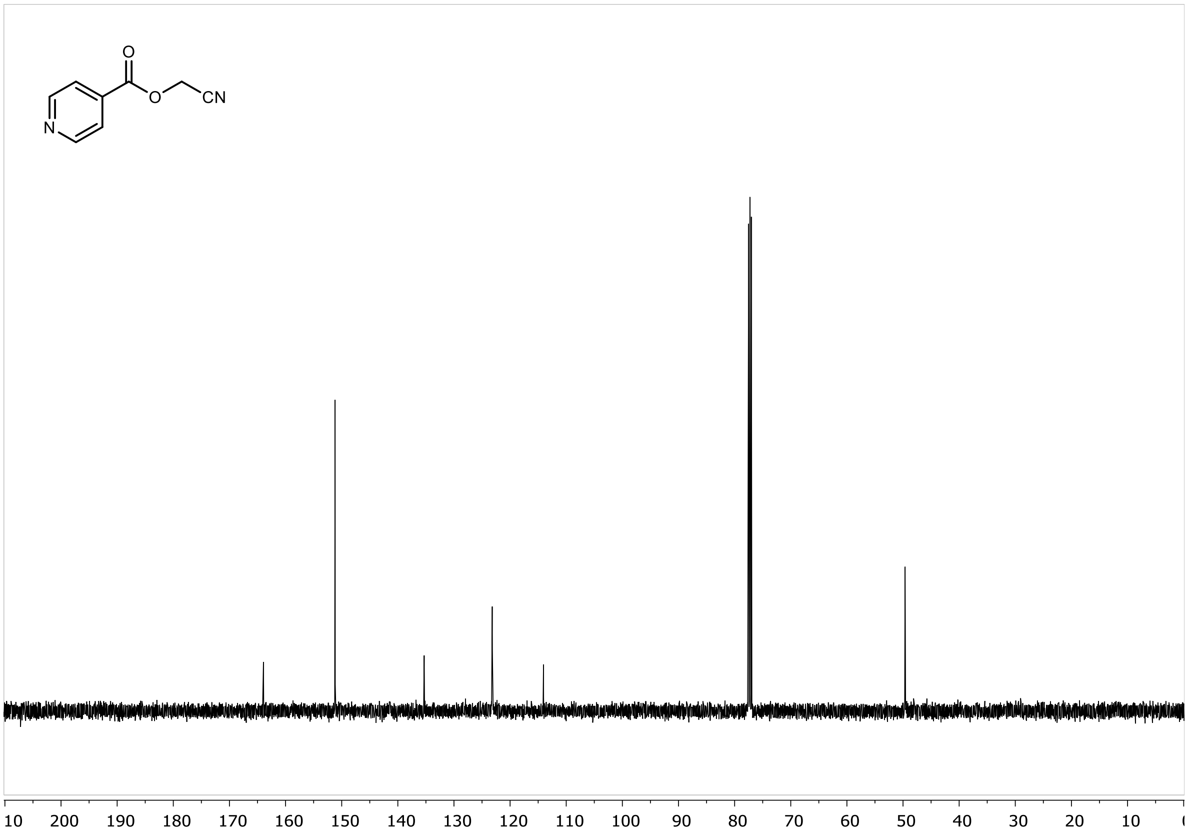

Supplementary Figure 73. ^{1}H NMR (500 MHz, $CDCl_3$) of **23.**

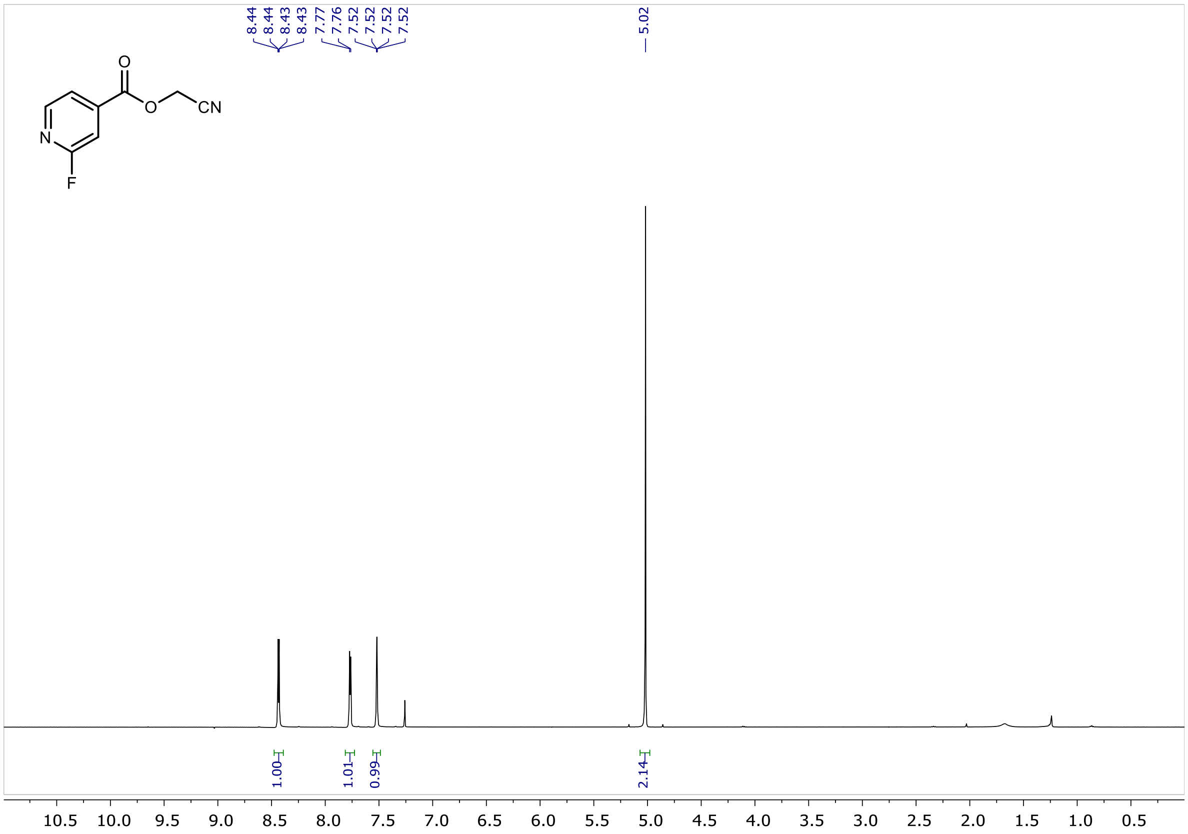

Supplementary Figure 74. ^{13}C NMR (125 MHz, $CDCl_3$) of **23.**

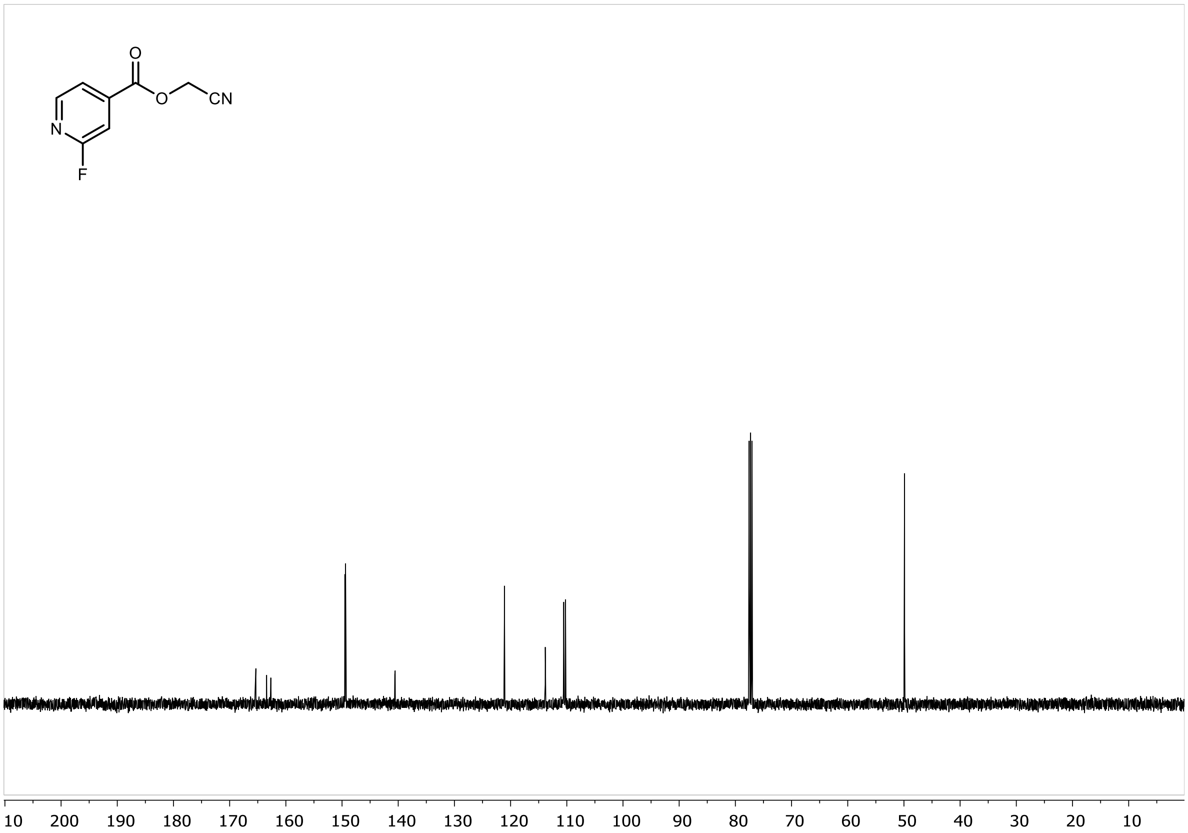

Supplementary Figure 75. ^{1}H NMR (500 MHz, $CDCl_3$) of **24.**

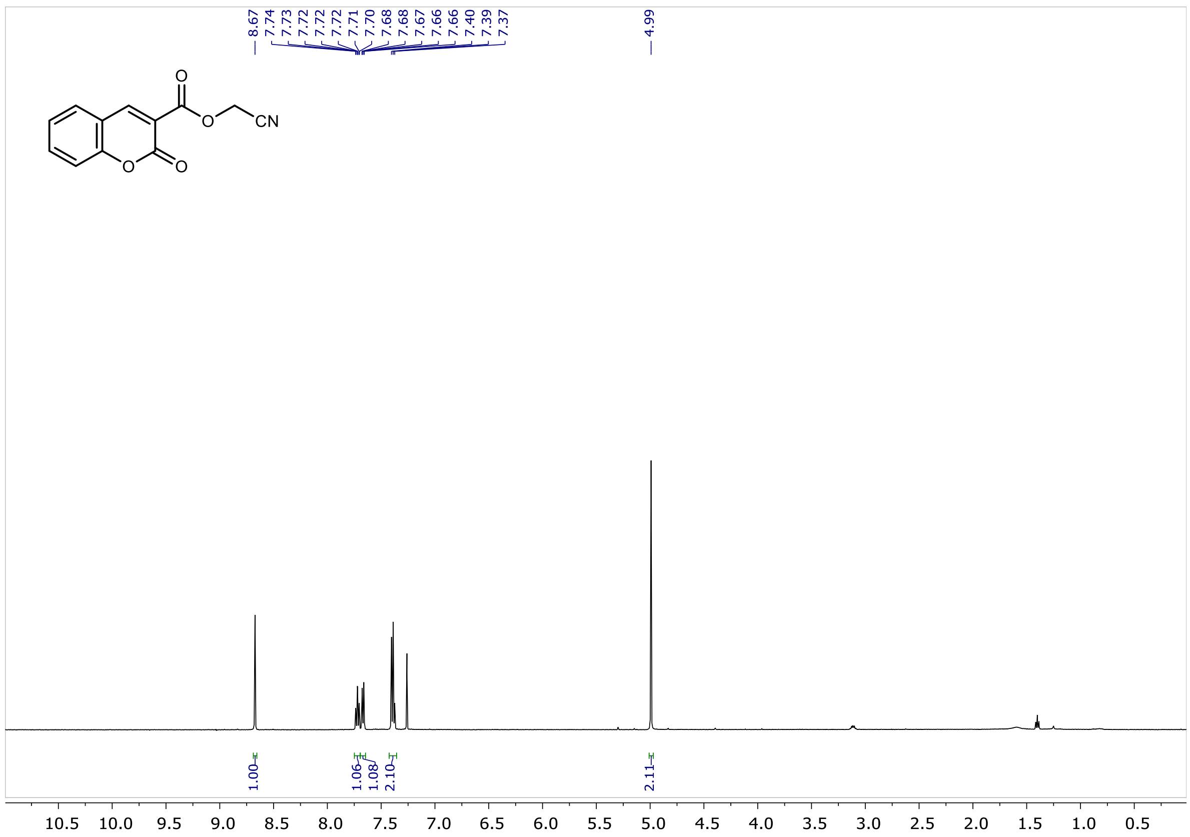

Supplementary Figure 76. ^{13}C NMR (125 MHz, $CDCl_3$) of **24.**

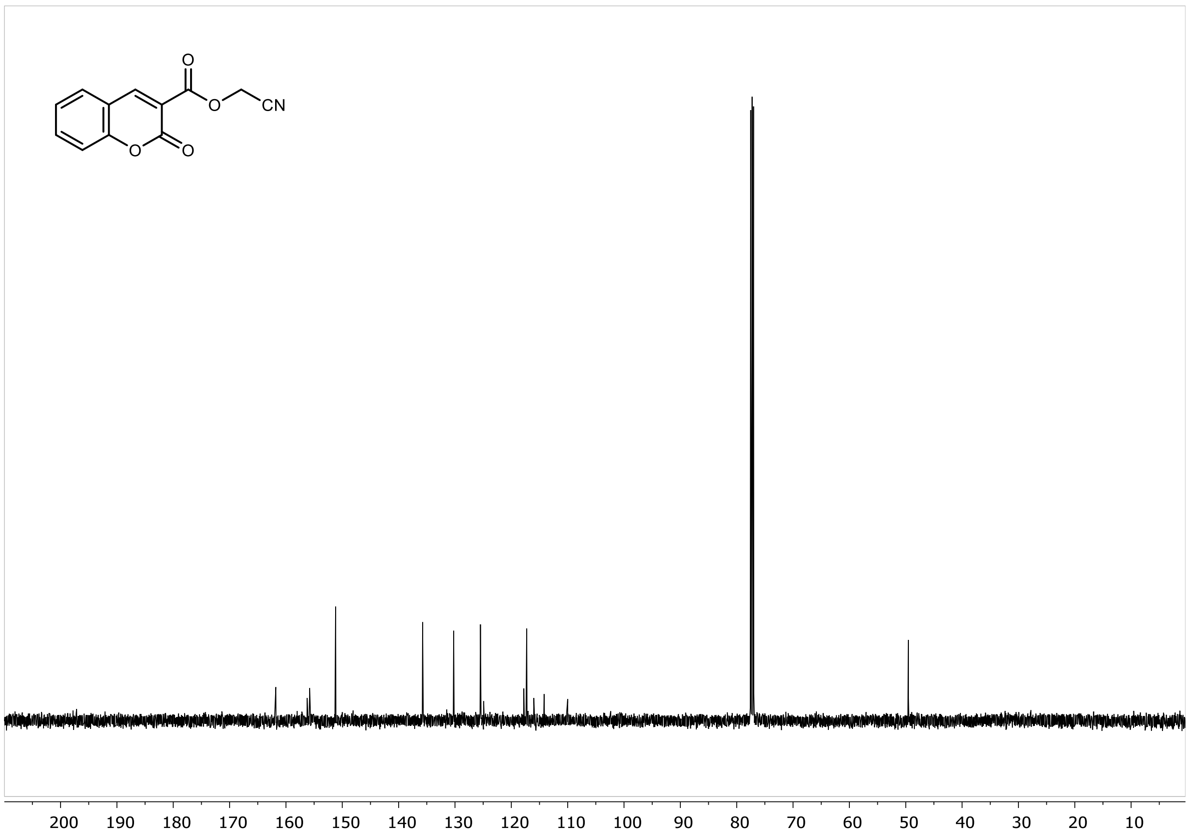

Supplementary Figure 77. ^{1}H NMR (500 MHz, DMSO-d_6) of **25.**

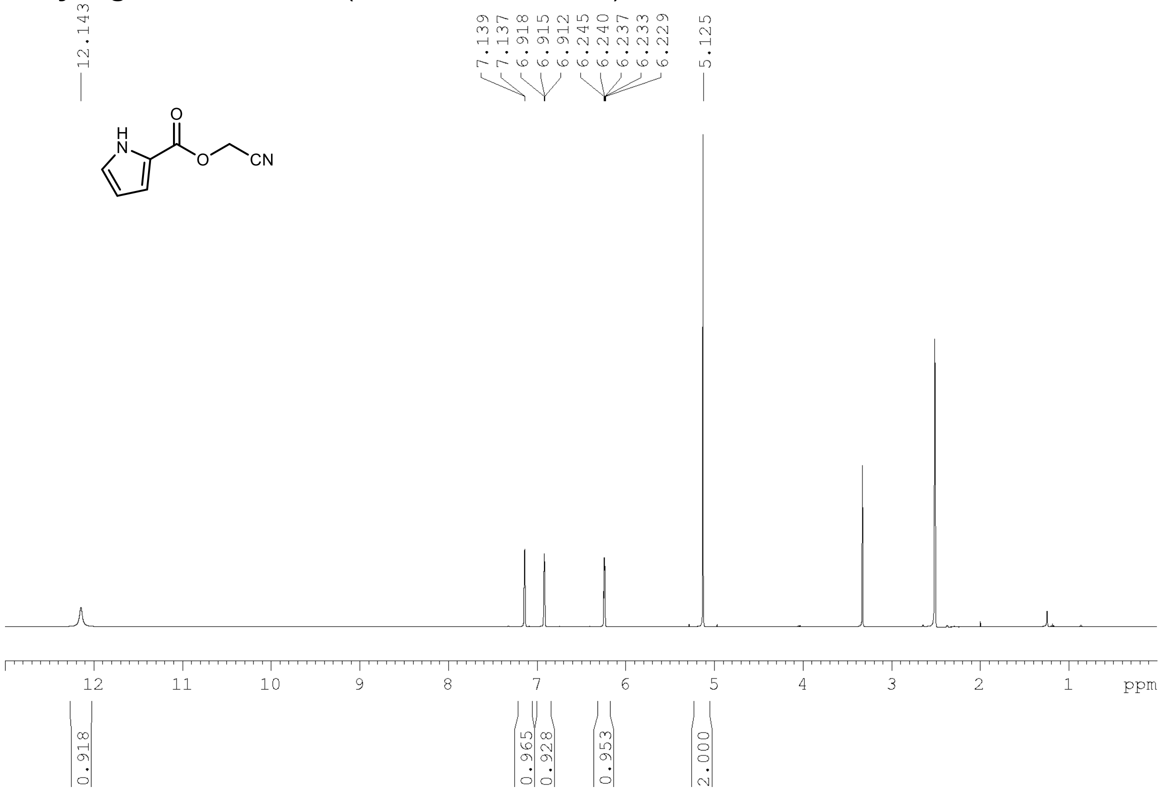

Supplementary Figure 78. ^{13}C NMR (125 MHz, DMSO-d_6) of **25.**

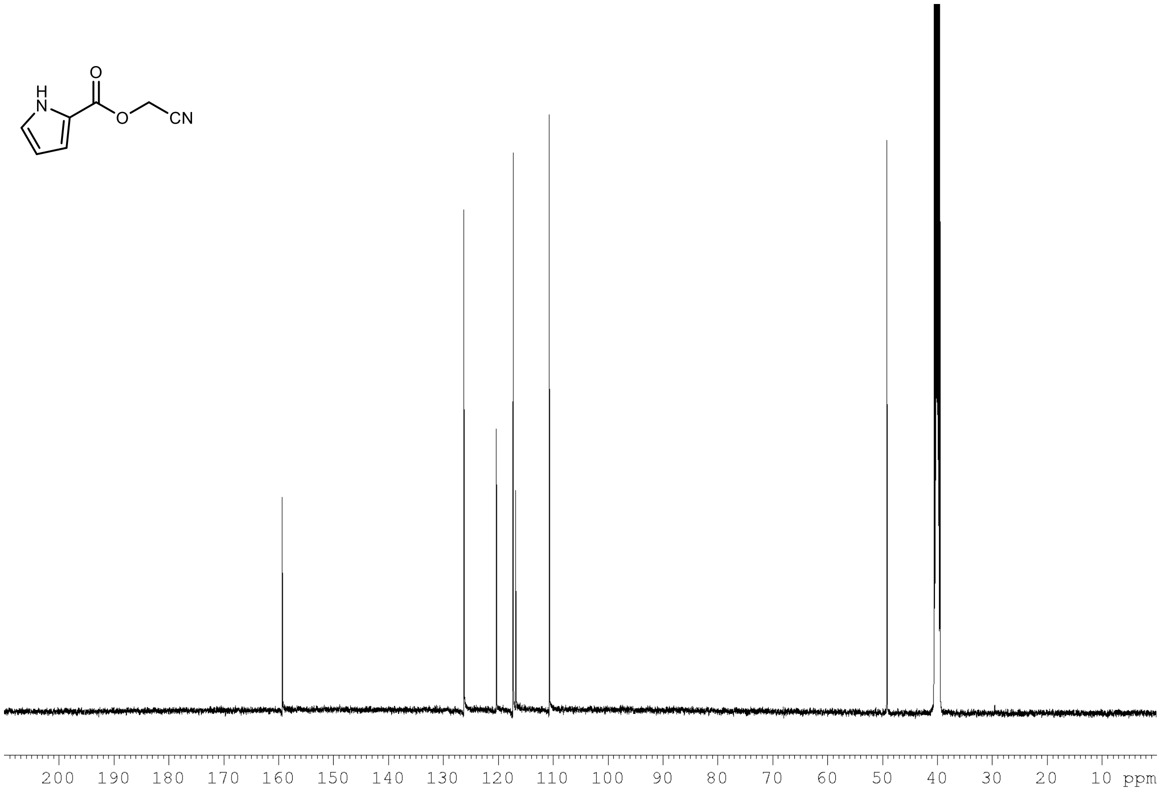

Supplementary Figure 79. ^{1}H NMR (500 MHz, $CDCl_3$) of Boc-**25a.**

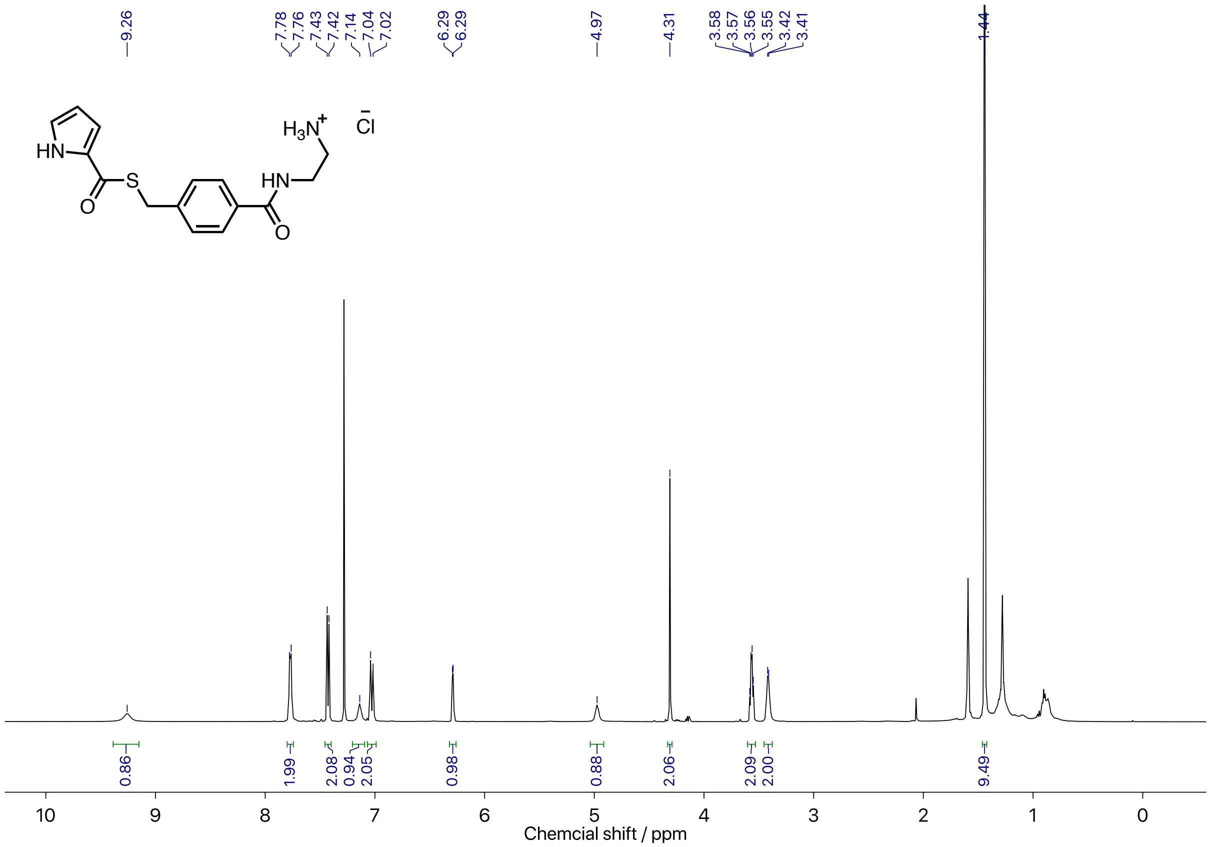

Supplementary Figure 80. ^{13}C NMR (500 MHz, $CDCl_3$) of Boc-**25a.**

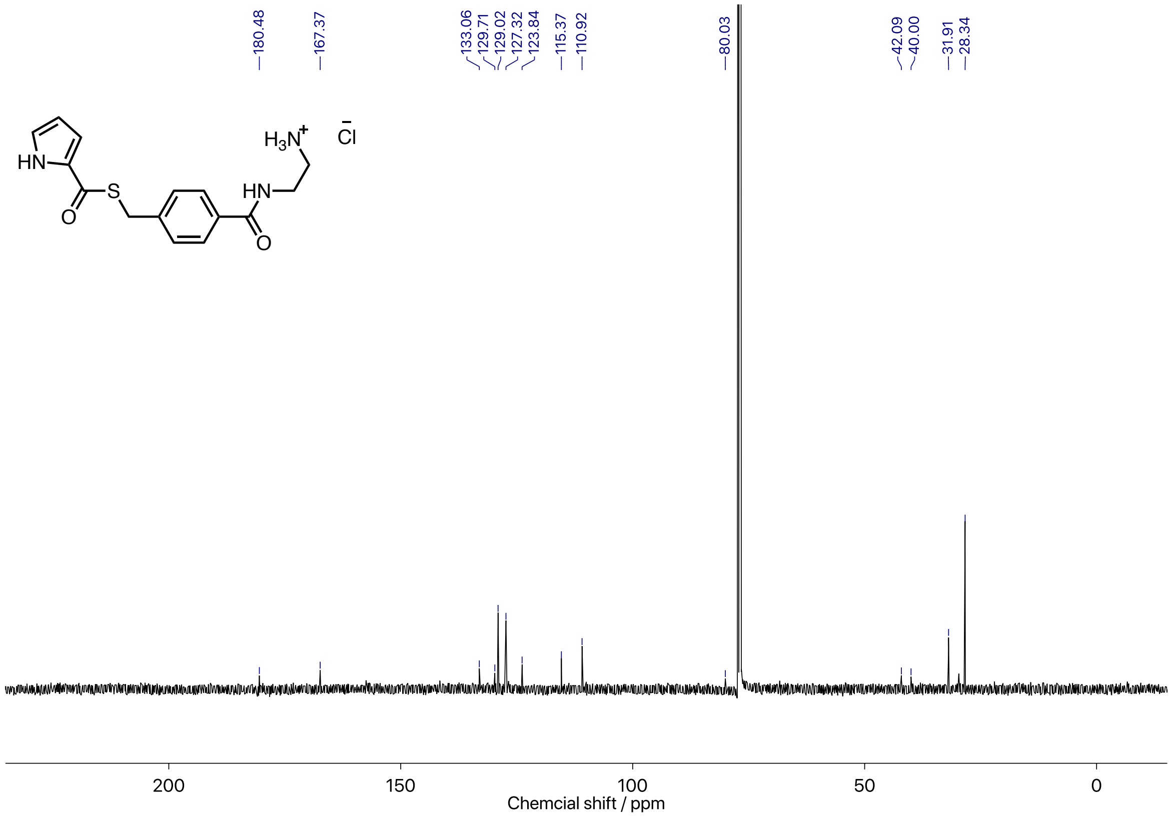

Supplementary Figure 81. ^{1}H NMR (500 MHz, $CDCl_3$) of **26.**

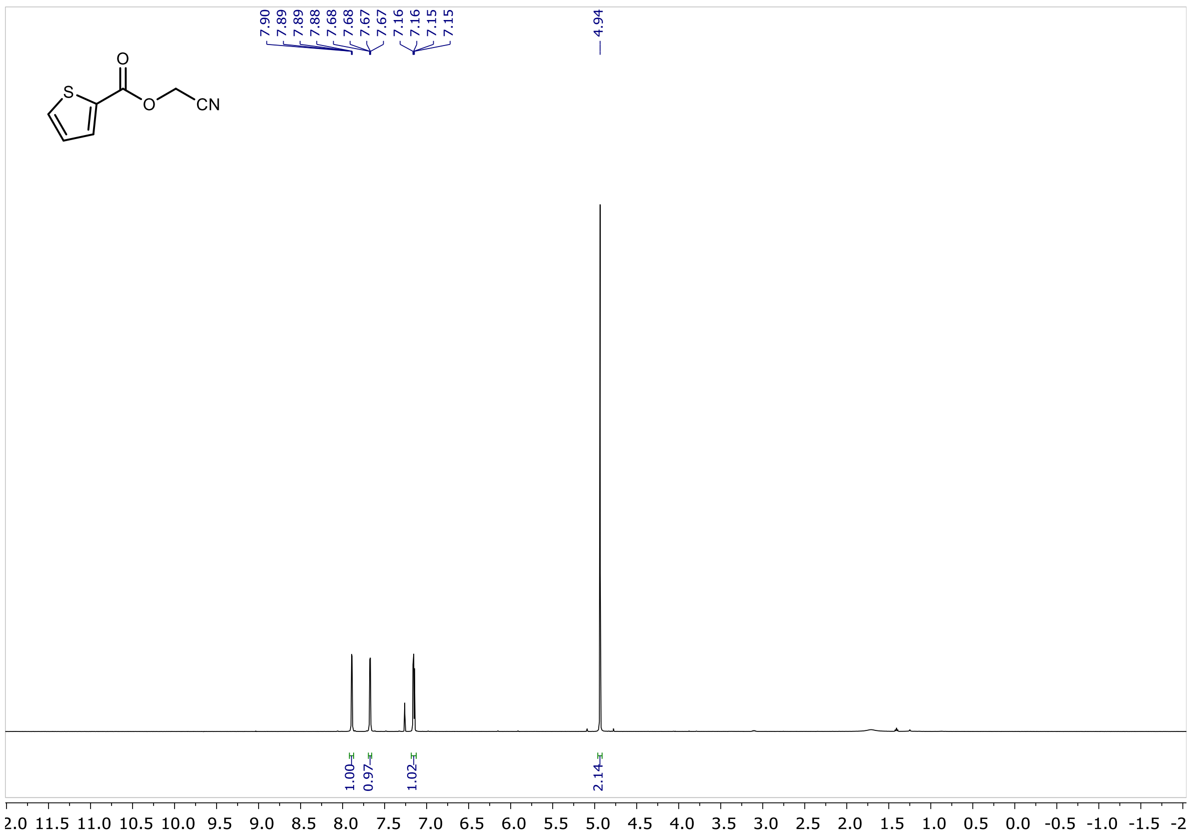

Supplementary Figure 82. ^{13}C NMR (125 MHz, $CDCl_3$) of **26.**

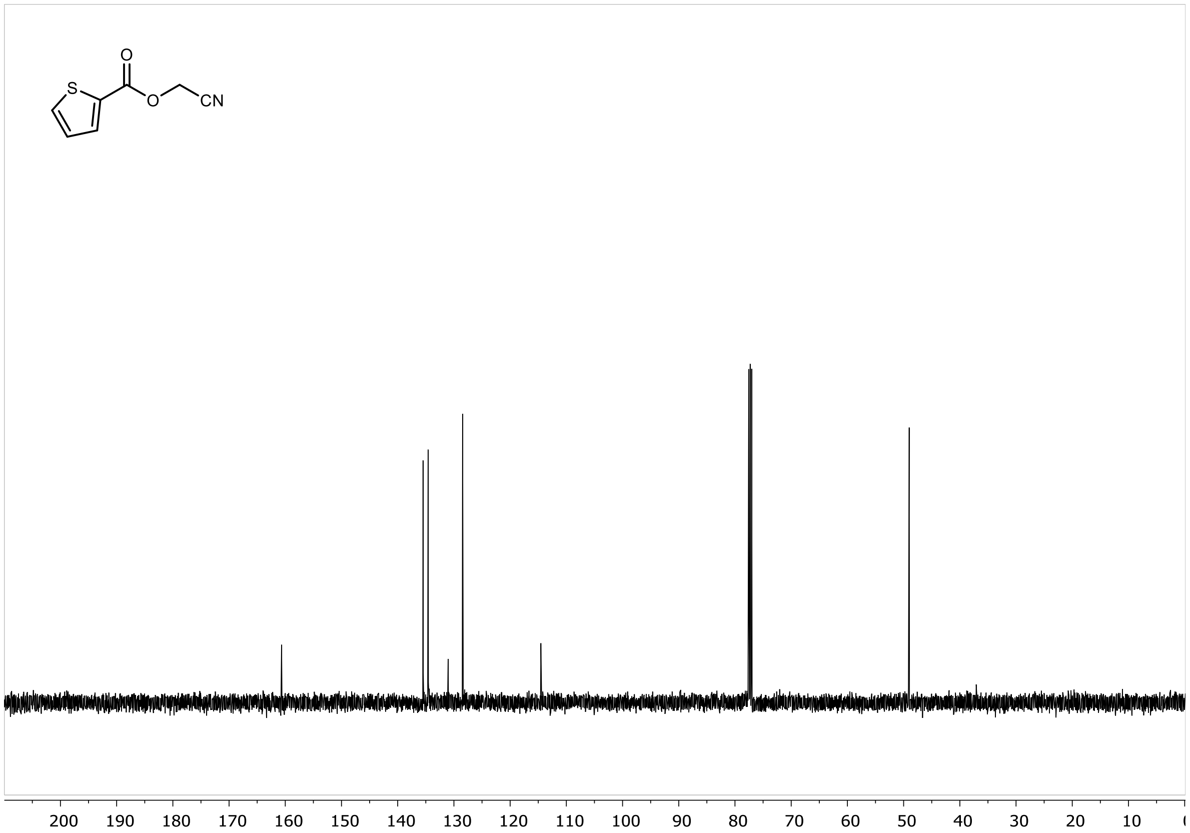

Supplementary Figure 83. ^{1}H NMR (500 MHz, $CDCl_3$) of Boc-**26a**.

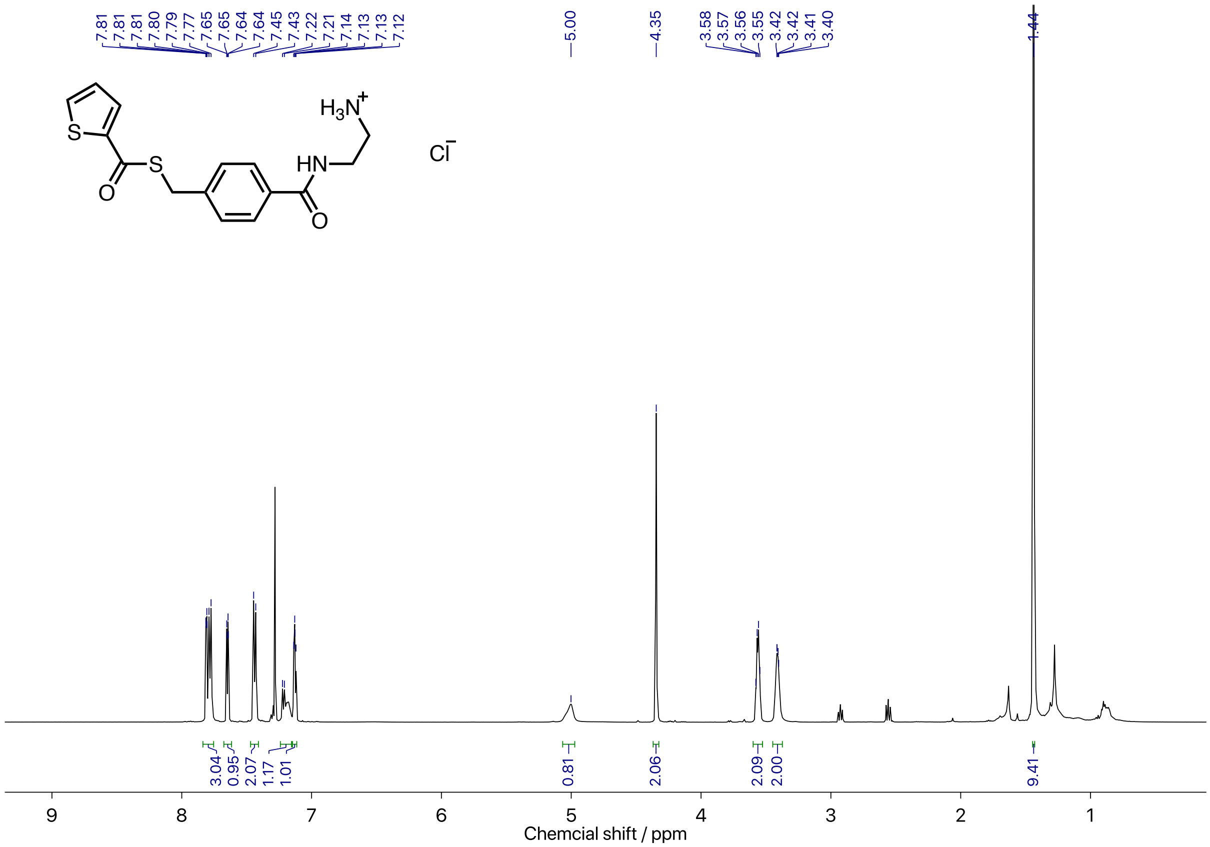

Supplementary Figure 84. ^{13}C NMR (500 MHz, $CDCl_3$) of Boc-**26a**.

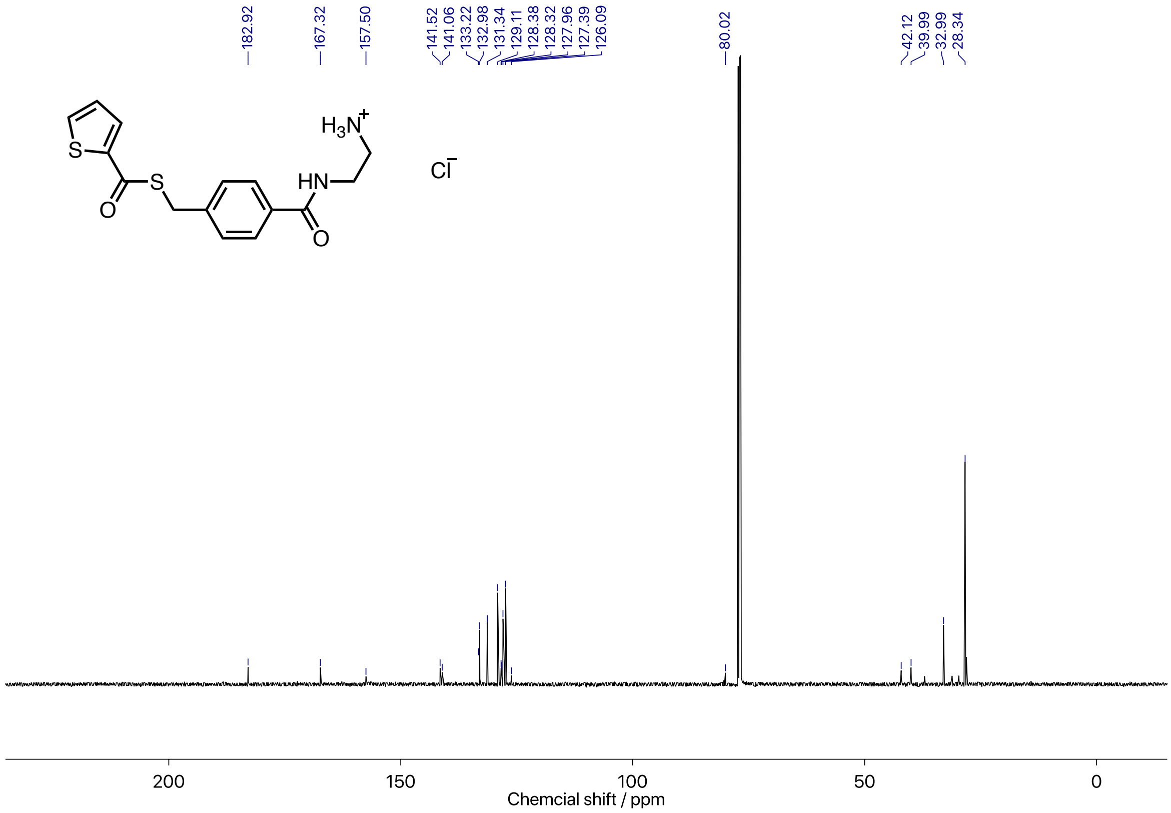

Supplementary Figure 85. ^{1}H NMR (500 MHz, $CDCl_3$) of Boc-**G**.

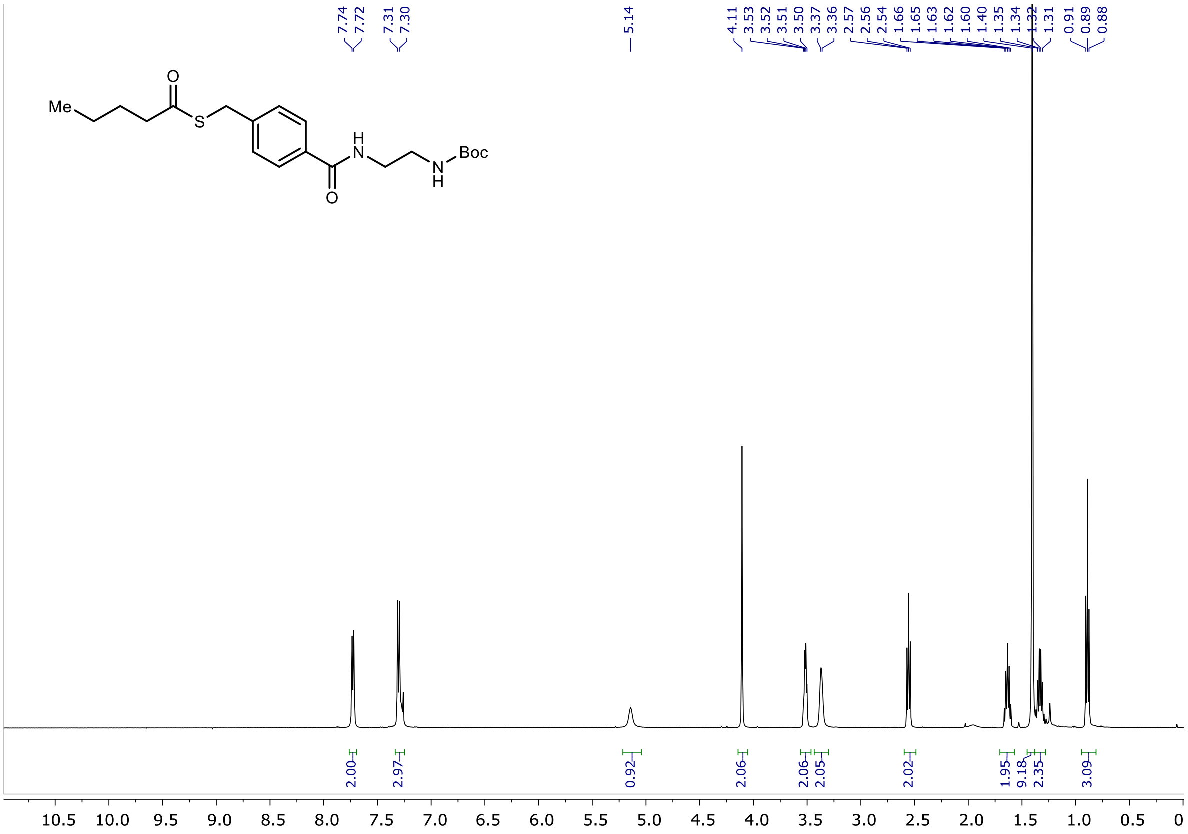

Supplementary Figure 86. ^{13}C NMR (125 MHz, $CDCl_3$) of Boc-**G.**

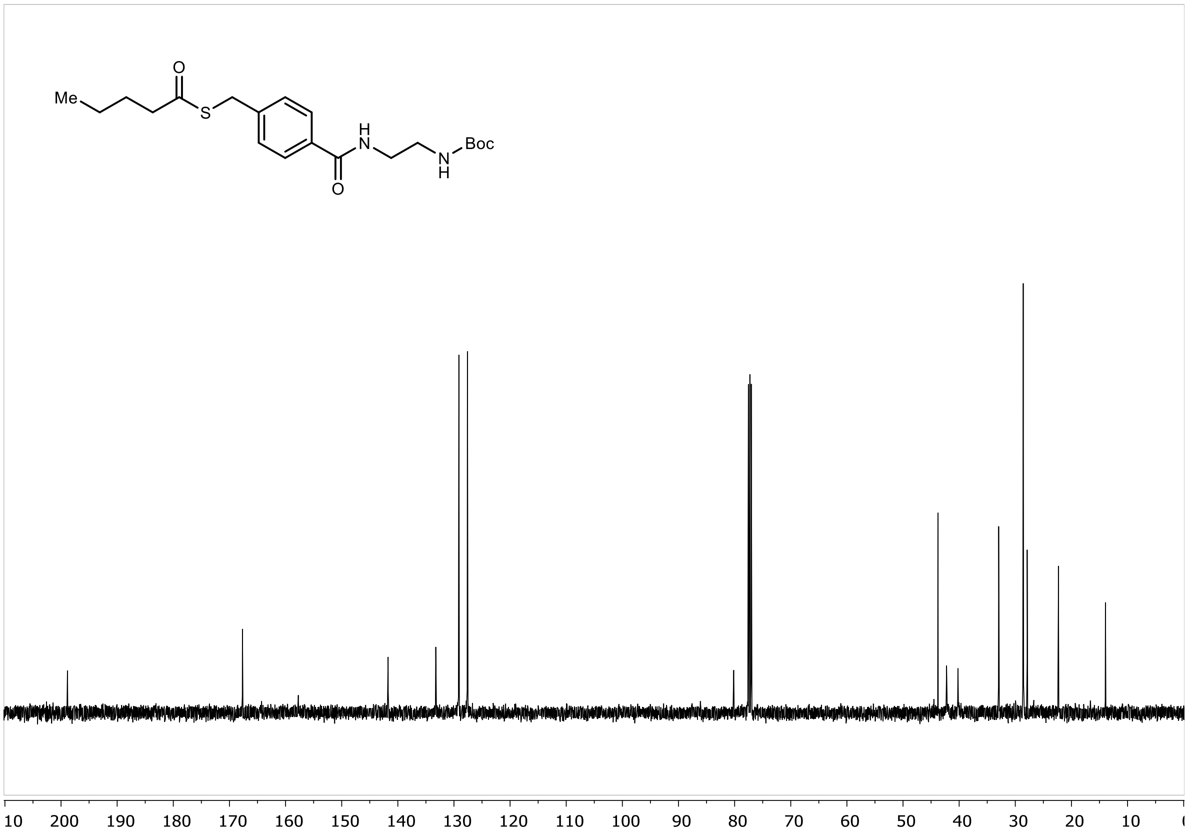

Supplementary Figure 87. ^{1}H NMR (500 MHz, $CDCl_3$) of Boc-**27.**

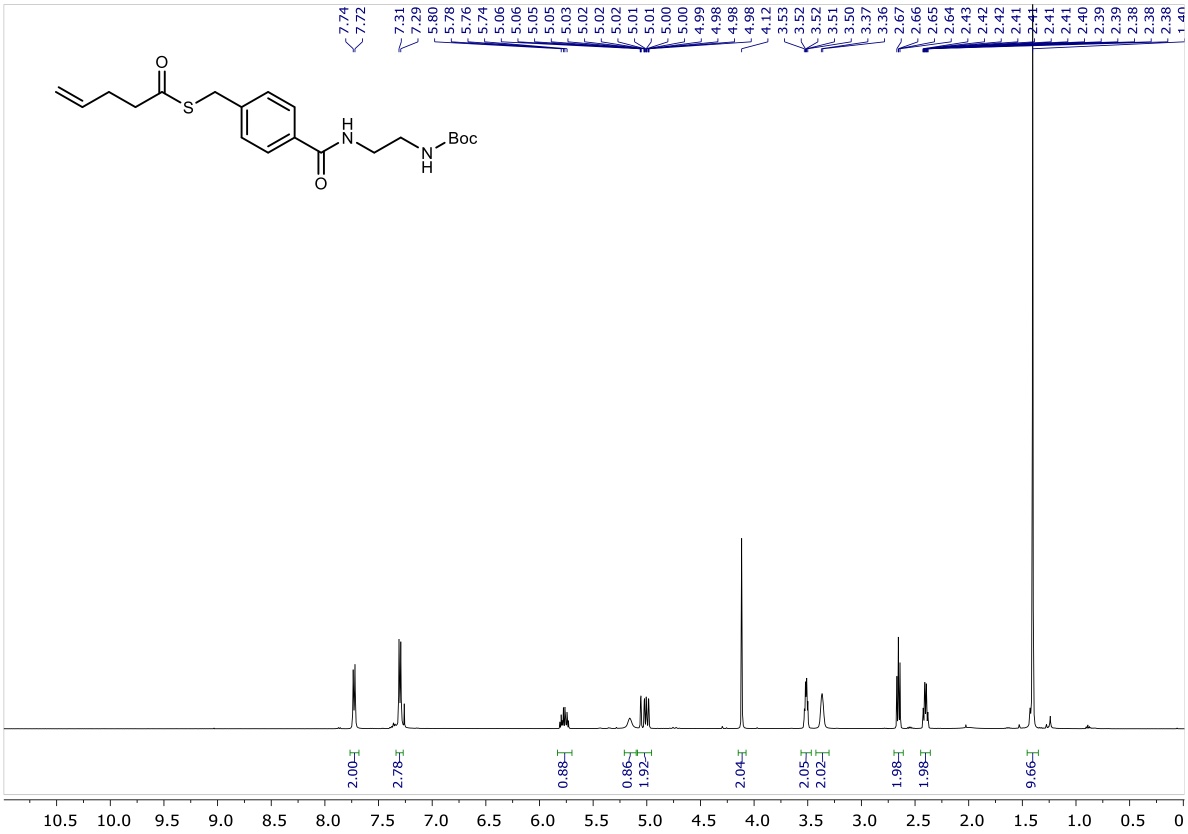

Supplementary Figure 88. ^{13}C NMR (125 MHz, $CDCl_3$) of Boc-**27.**

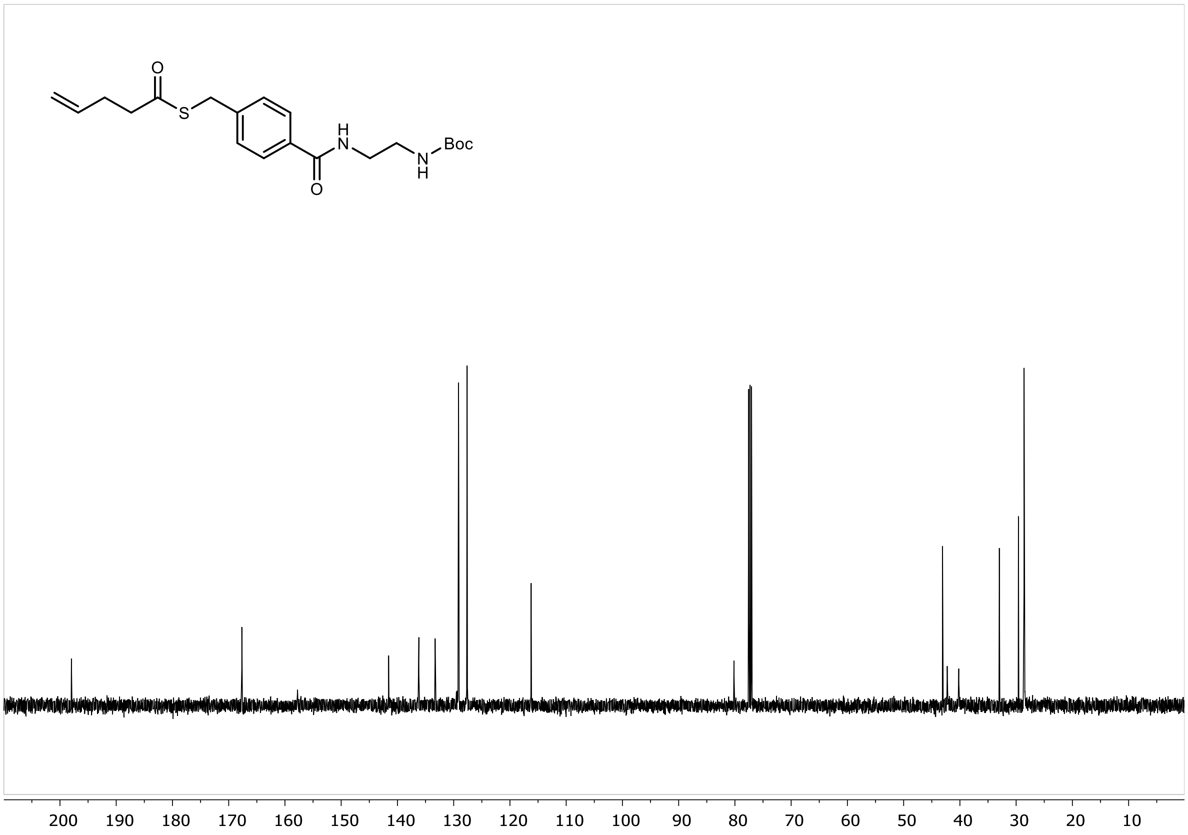

Supplementary Figure 89. ^{1}H NMR (500 MHz, $CDCl_3$) of Boc-**28.**

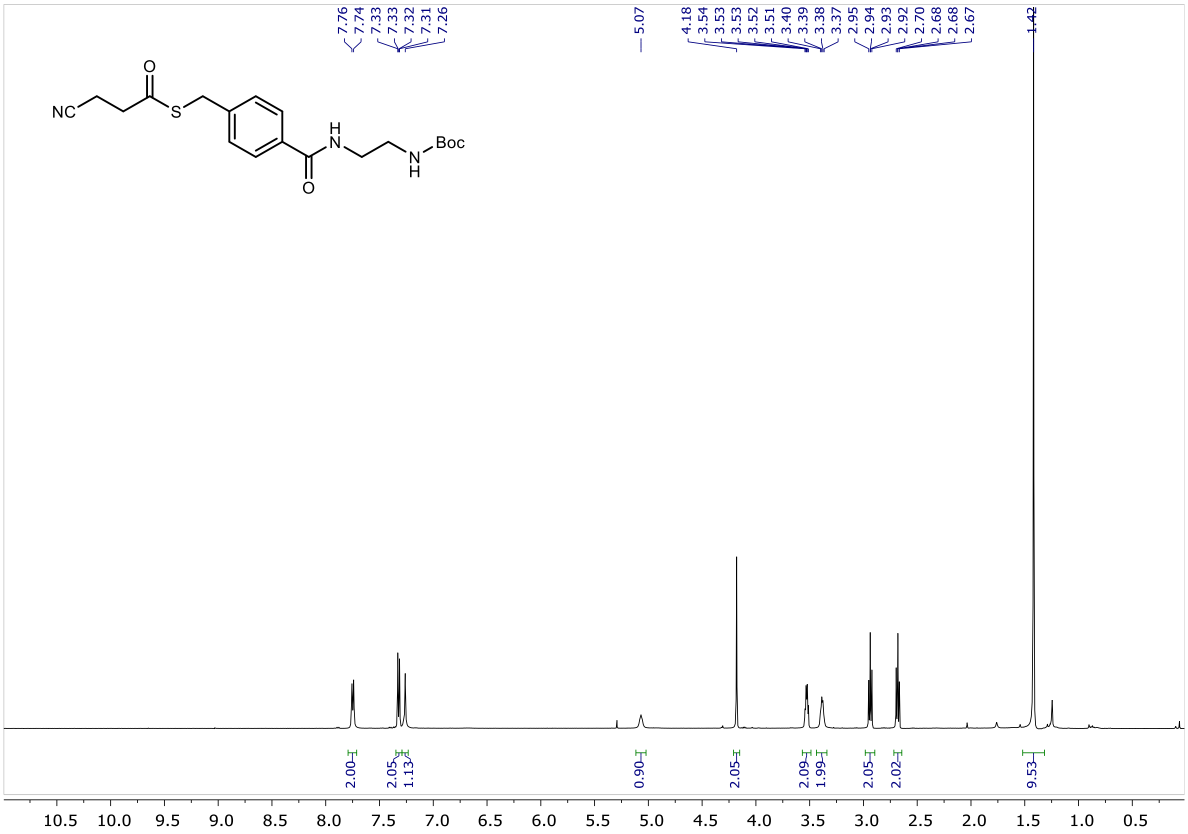

Supplementary Figure 90. ^{13}C NMR (125 MHz, $CDCl_3$) of Boc-**28.**

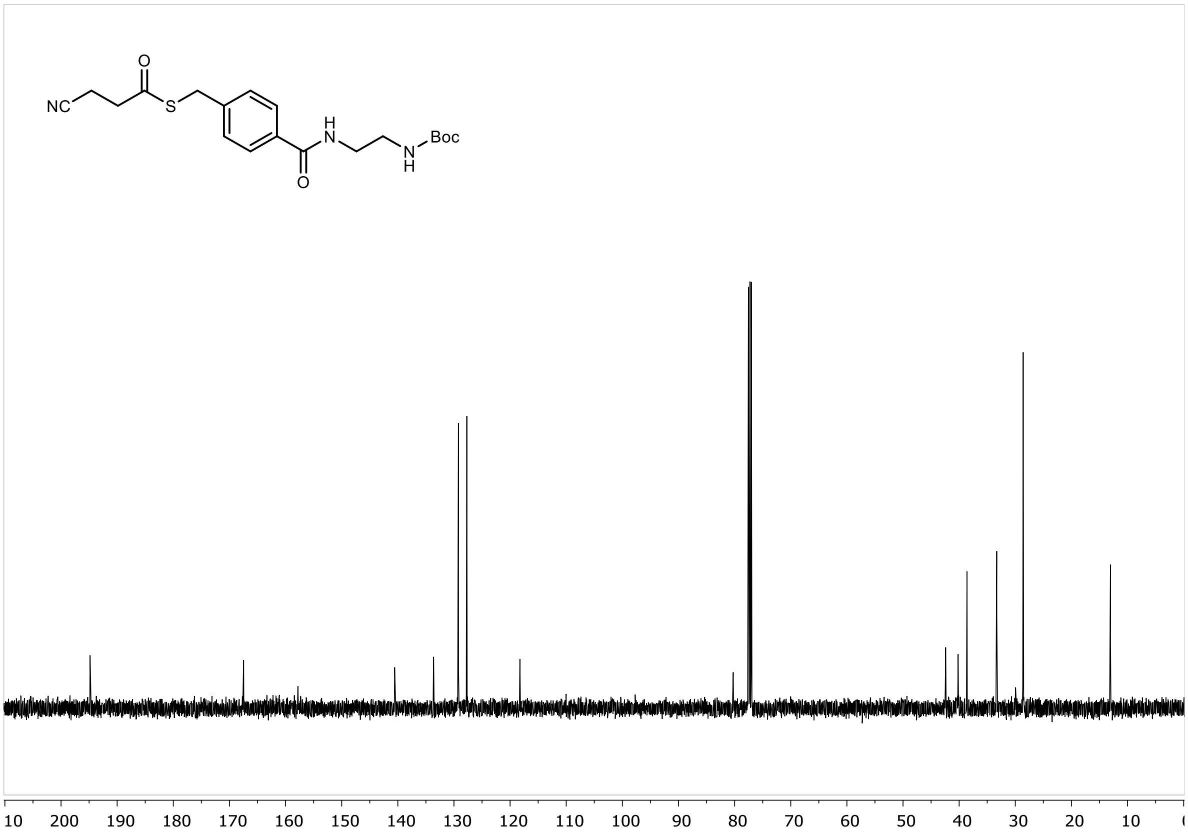

Supplementary Figure 91. ^{1}H NMR (500 MHz, $CDCl_3$) of Boc-**29.**

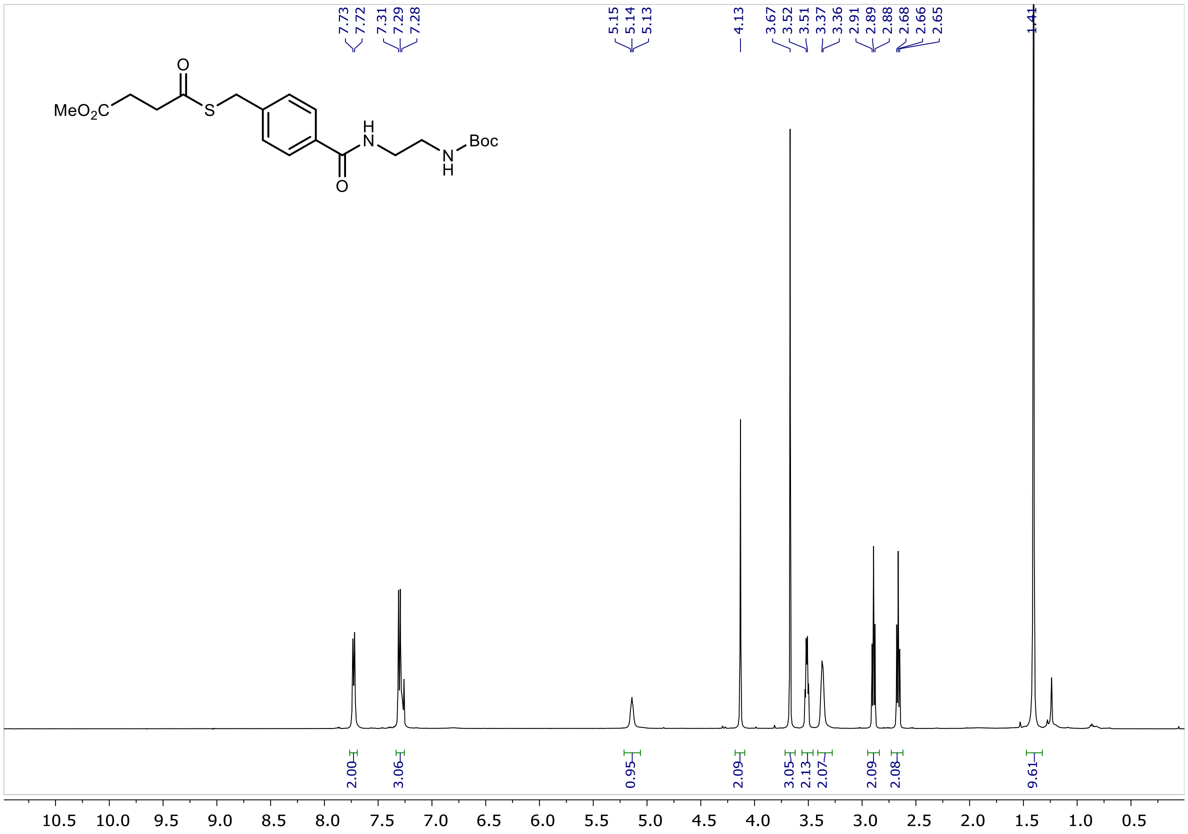

Supplementary Figure 92. ^{13}C NMR (125 MHz, $CDCl_3$) of Boc-**29.**

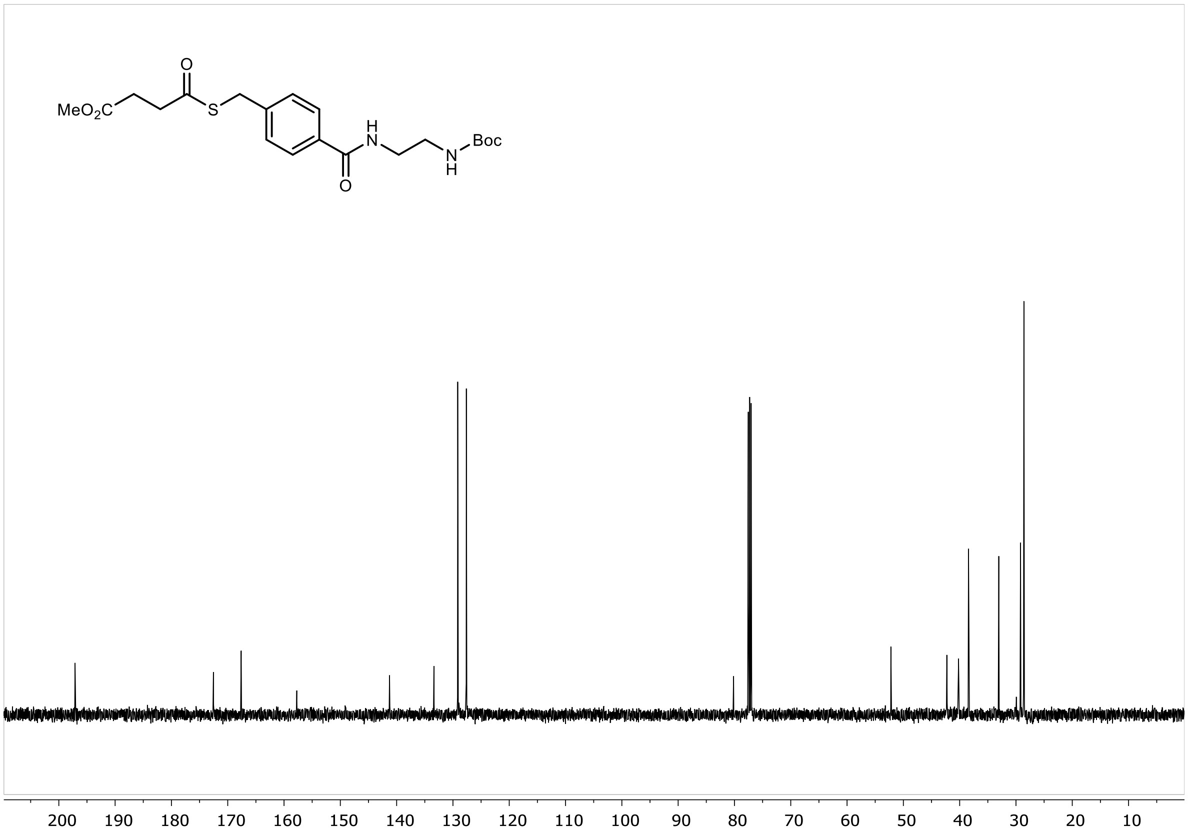

Supplementary Figure 93. ^{1}H NMR (500 MHz, $CDCl_3$) of Boc-**30.**

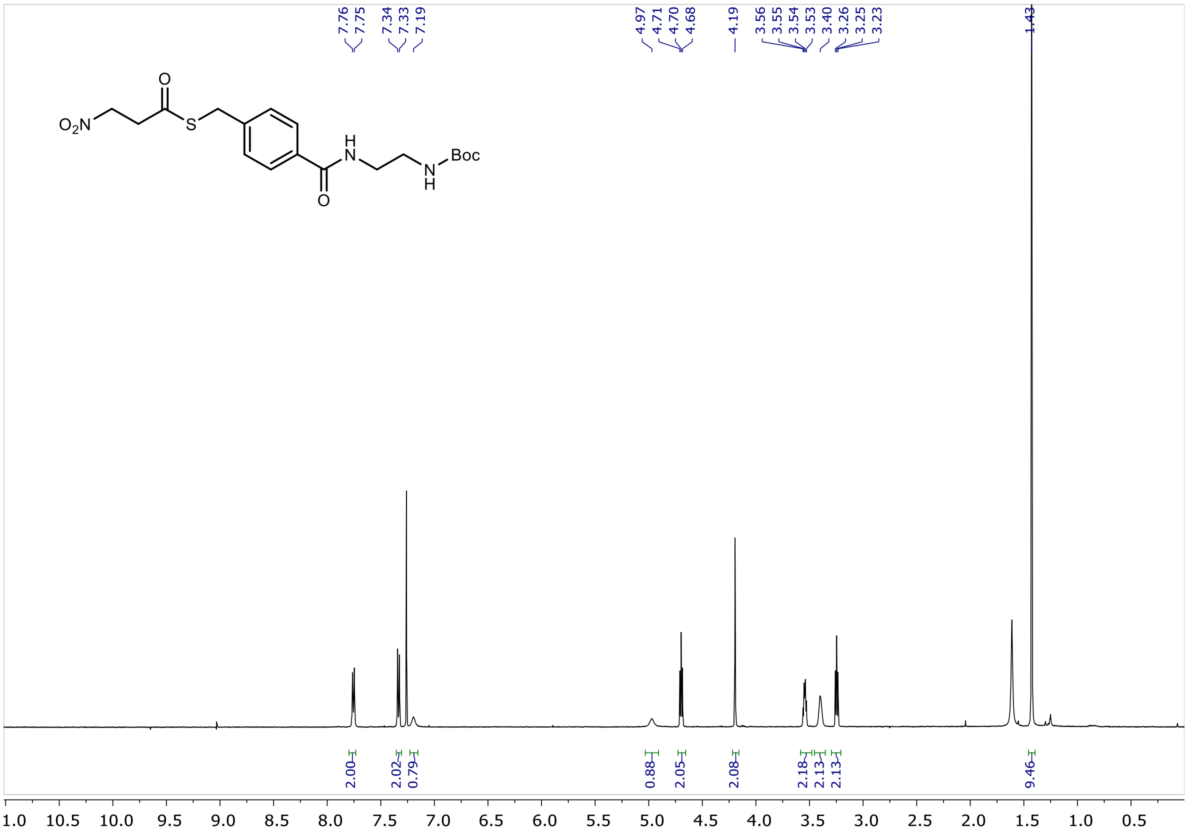

Supplementary Figure 94. ^{13}C NMR (125 MHz, $CDCl_3$) of Boc-**30.**

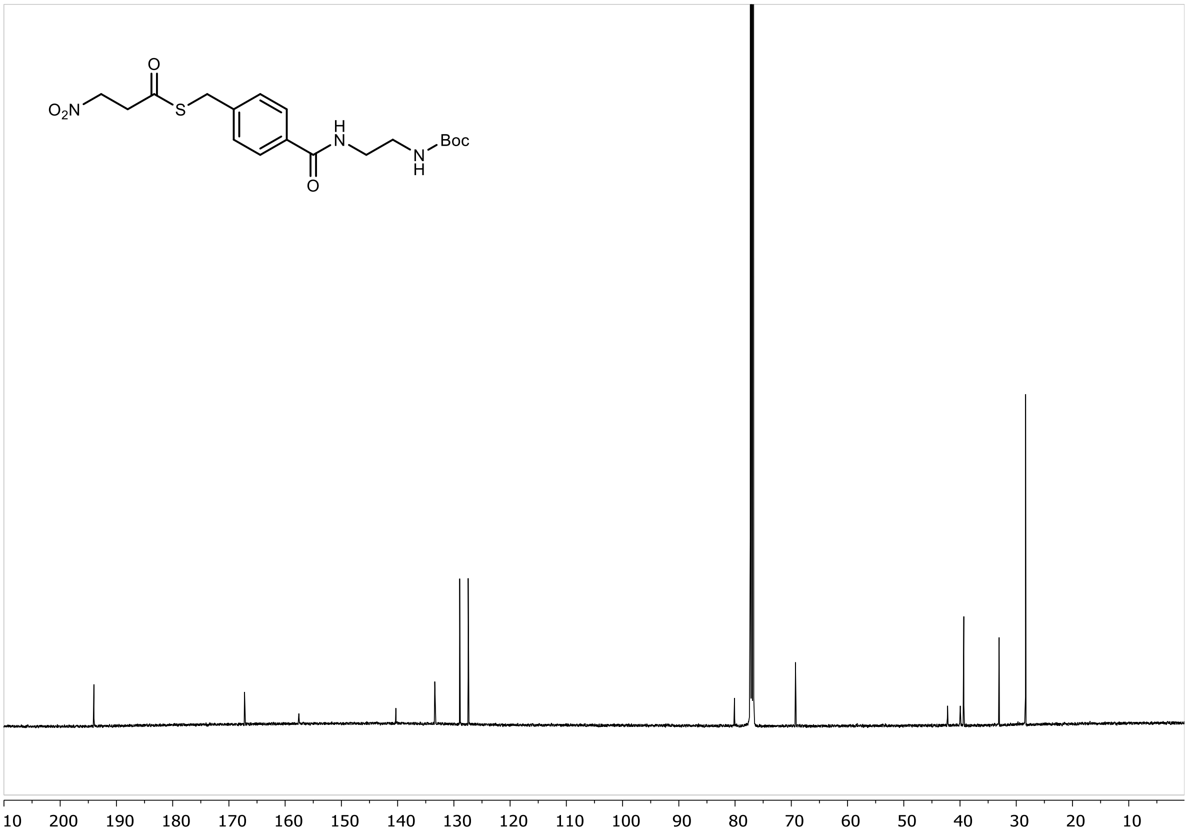

Supplementary Figure 95. ^{1}H NMR (500 MHz, $CDCl_3$) of Boc-**31.**

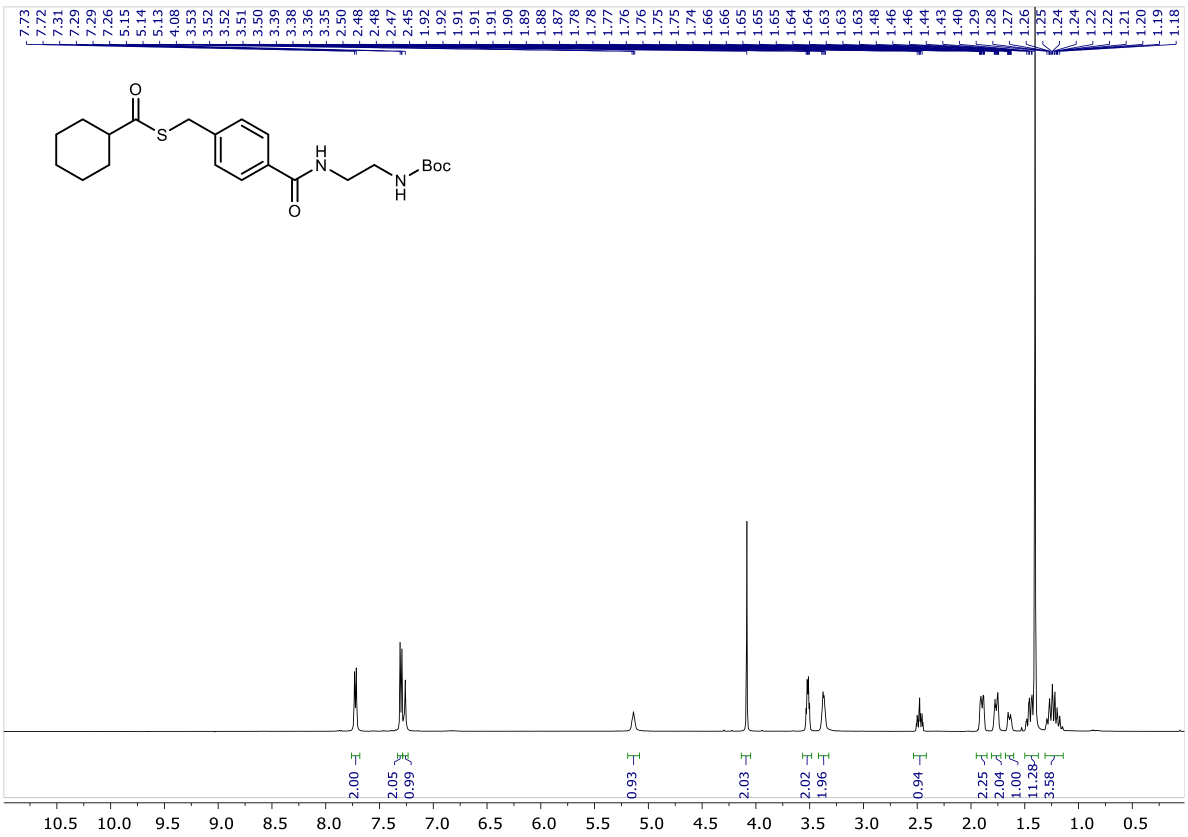

Supplementary Figure 96. ^{13}C NMR (125 MHz, $CDCl_3$) of Boc-**31.**

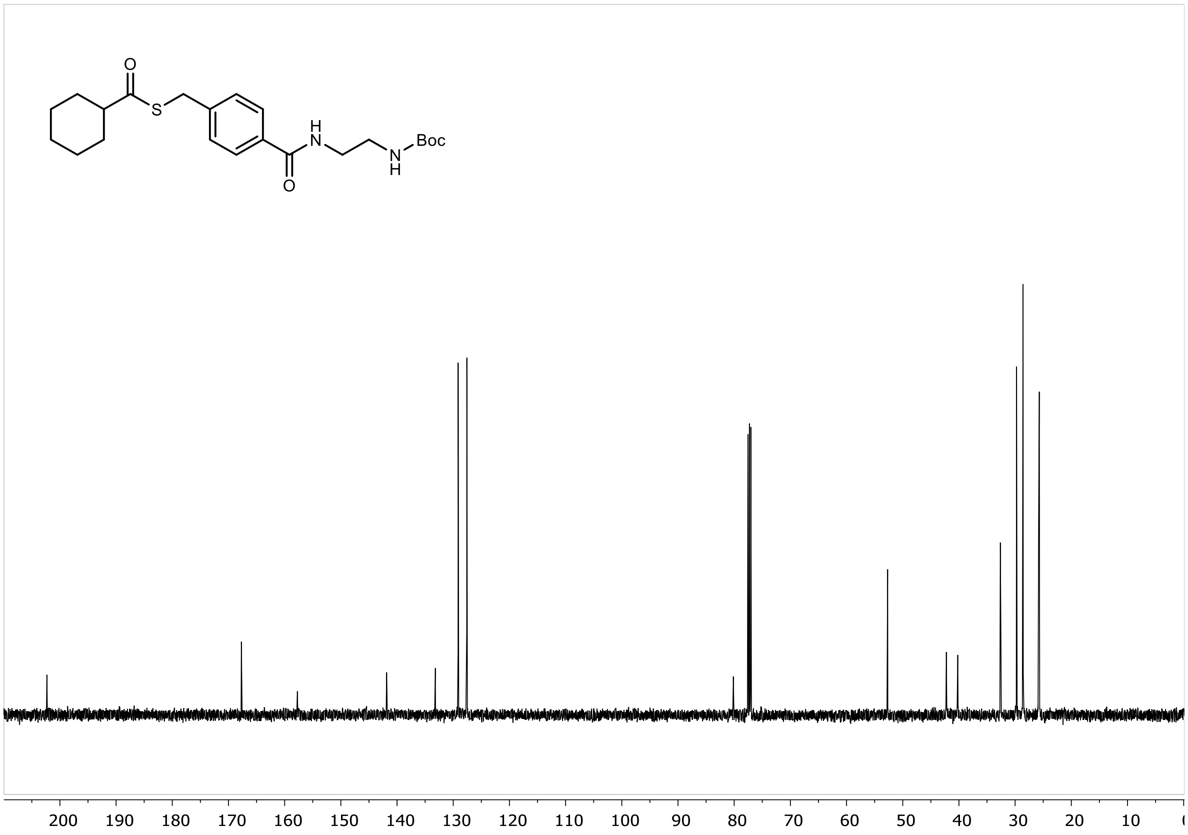

Supplementary Figure 97. ^{1}H NMR (500 MHz, $CDCl_3$) of Boc-**32.**

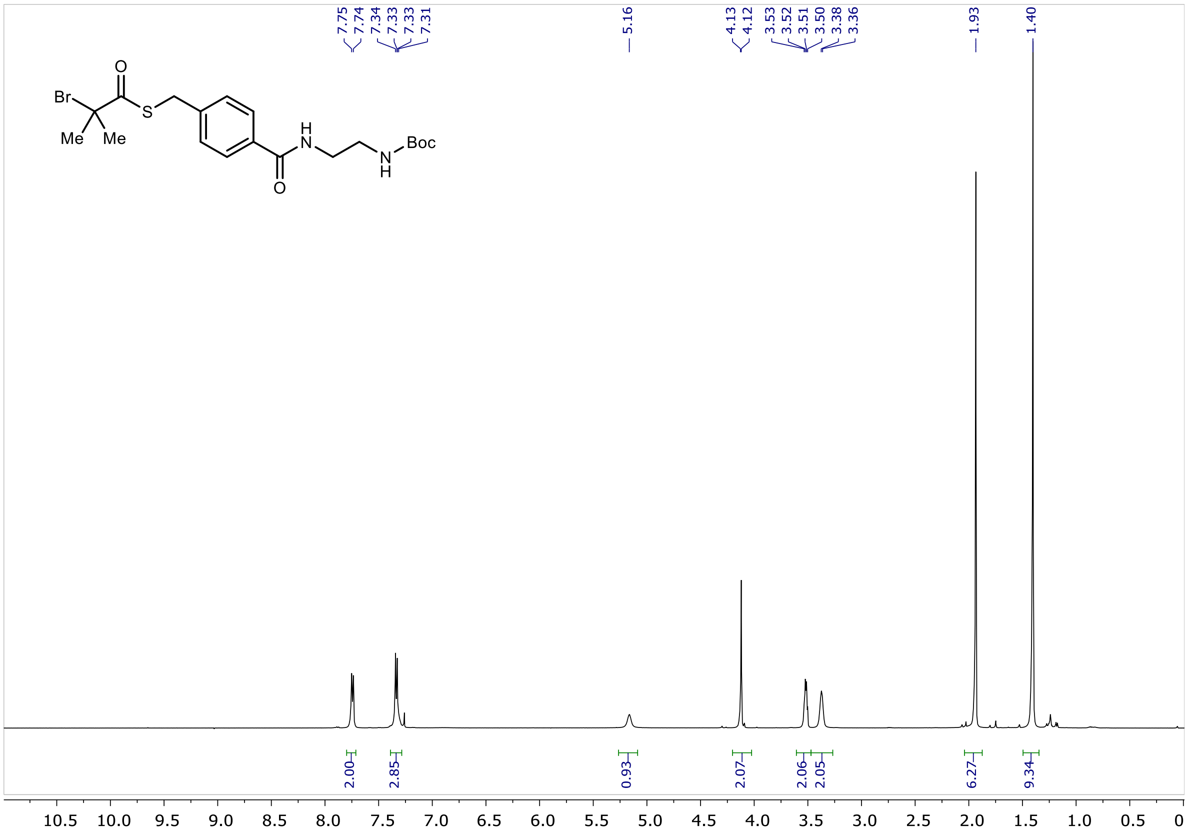

Supplementary Figure 98. ^{13}C NMR (125 MHz, $CDCl_3$) of Boc-**32.**

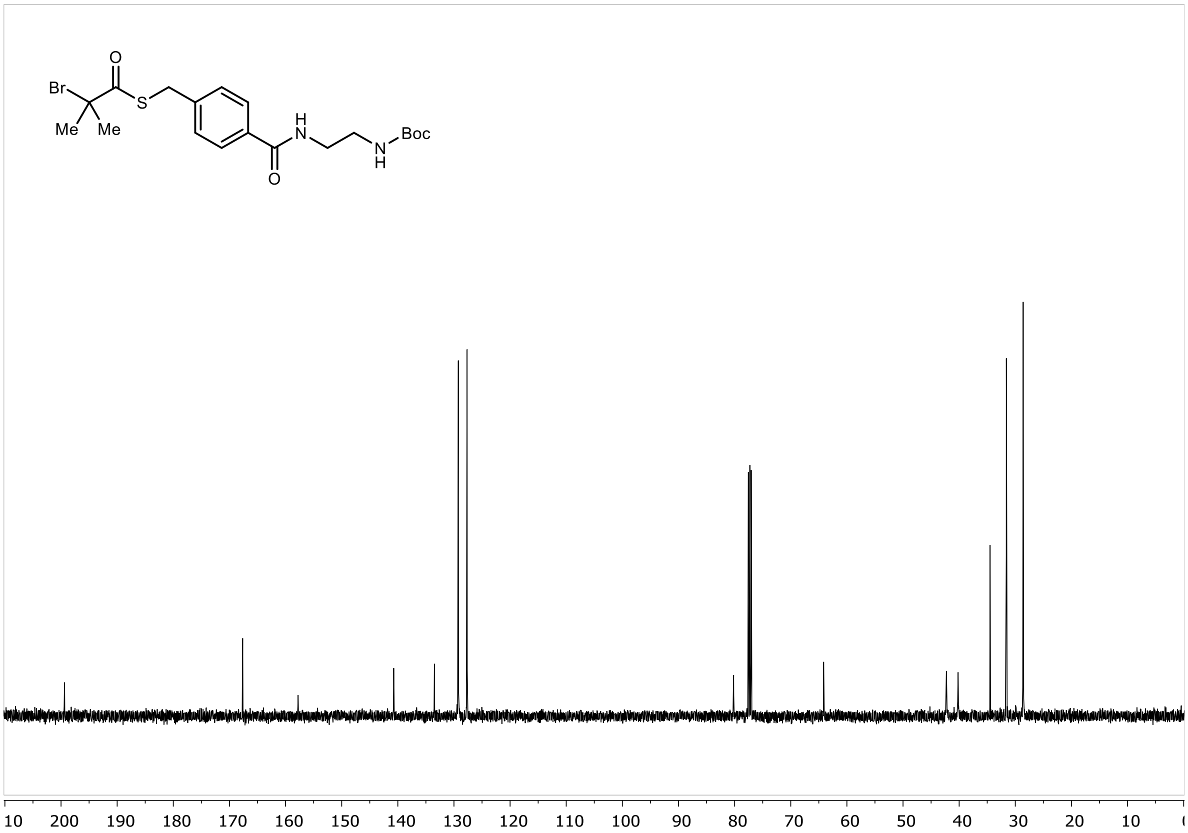

Supplementary Figure 99. ^{1}H NMR (500 MHz, DMSO-d_6) of **33.**

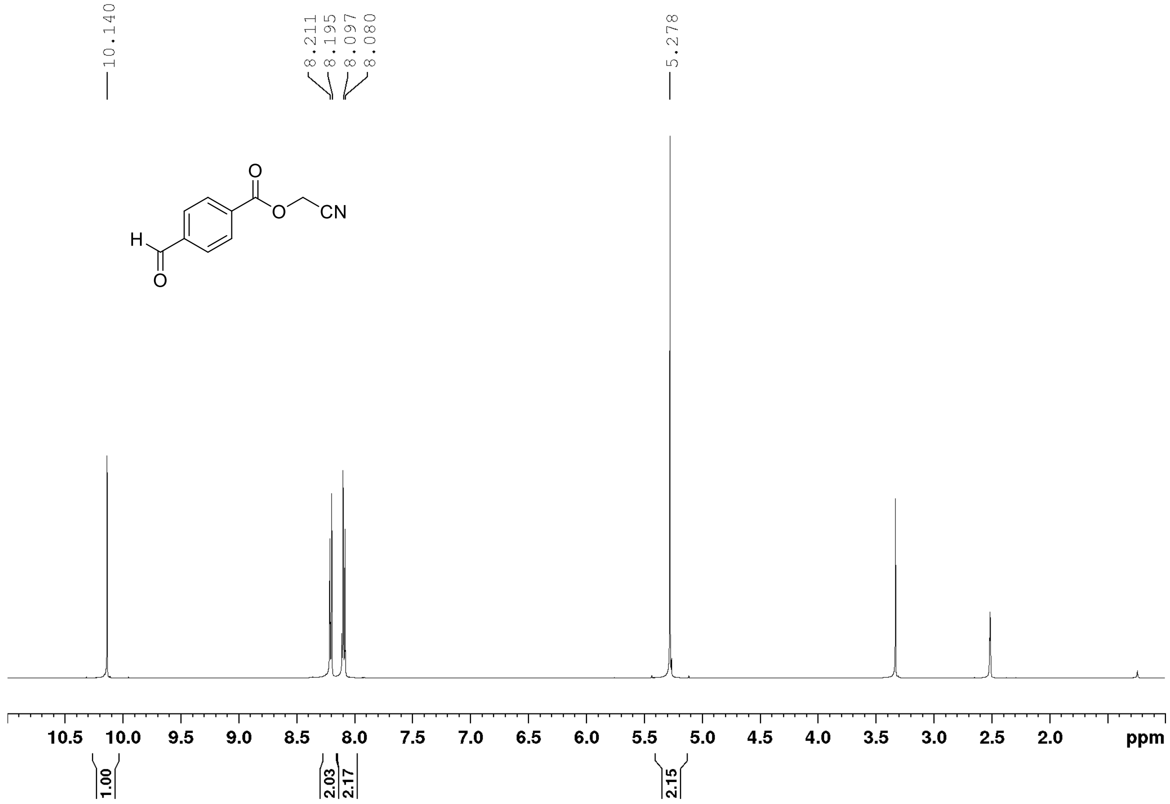

Supplementary Figure 100. ^{13}C NMR (125 MHz, DMSO-d_6) of **33.**

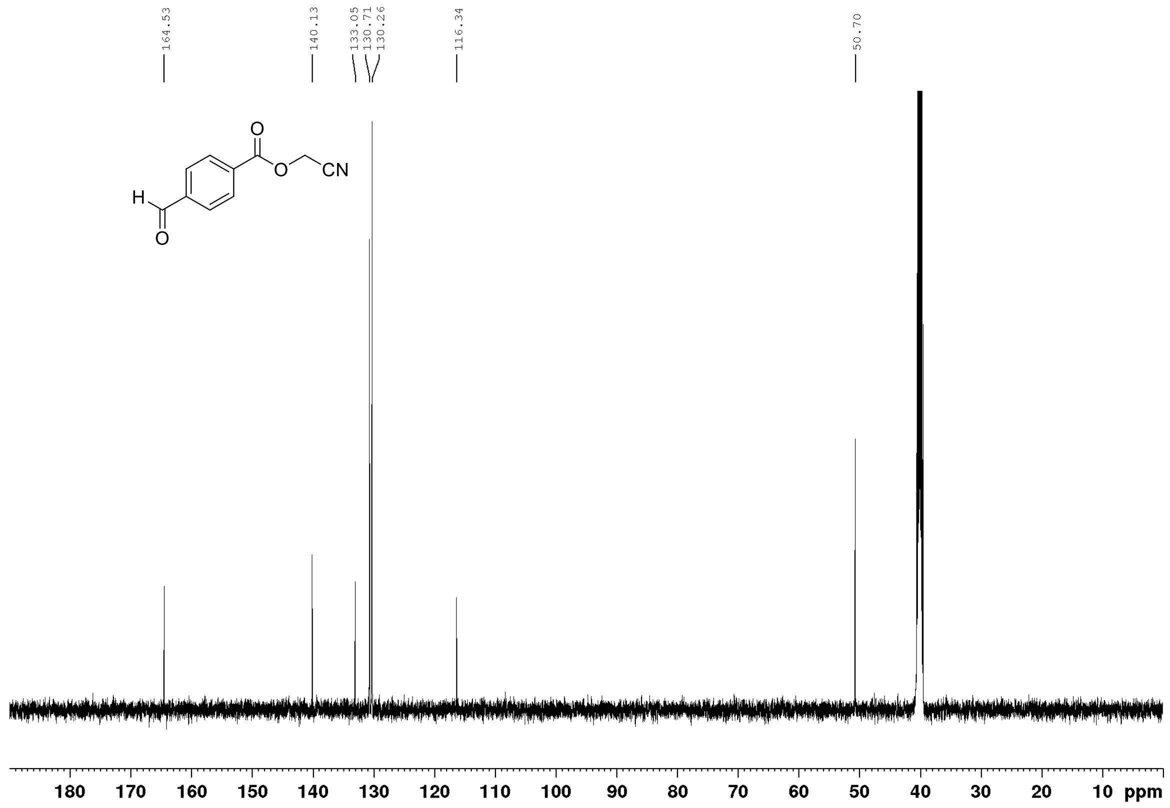

Supplementary Figure 101. ^{1}H NMR (500 MHz, $CDCl_3$) of **34.**

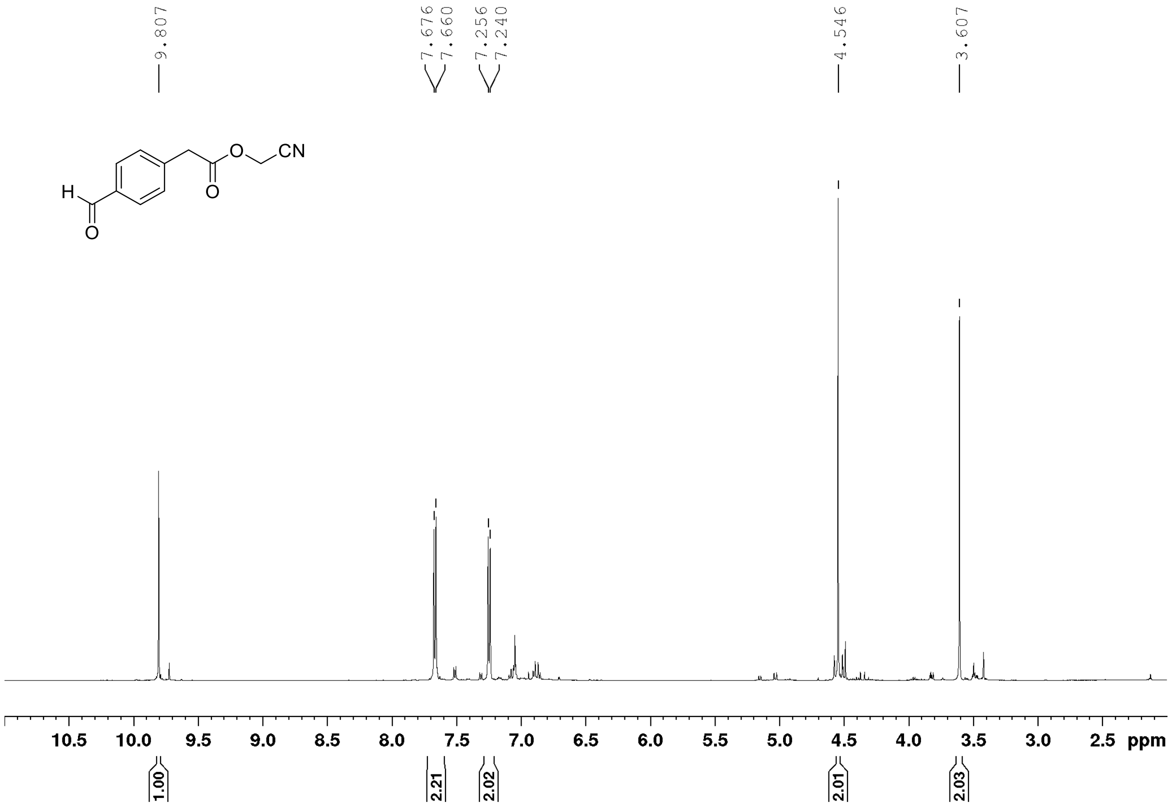

Supplementary Figure 102. ^{13}C NMR (125 MHz, $CDCl_3$) of **34.**

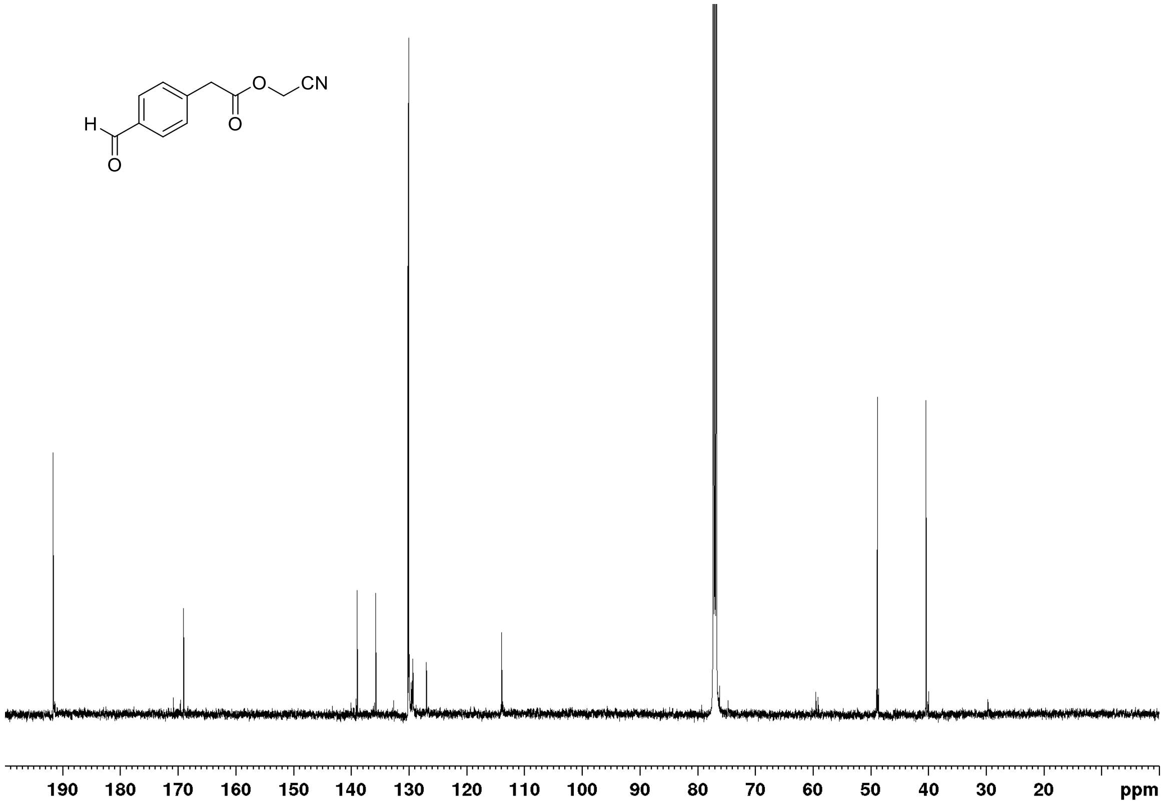

Supplementary Figure 103. ^{1}H NMR (500 MHz, DMSO-d_6) of **35.**

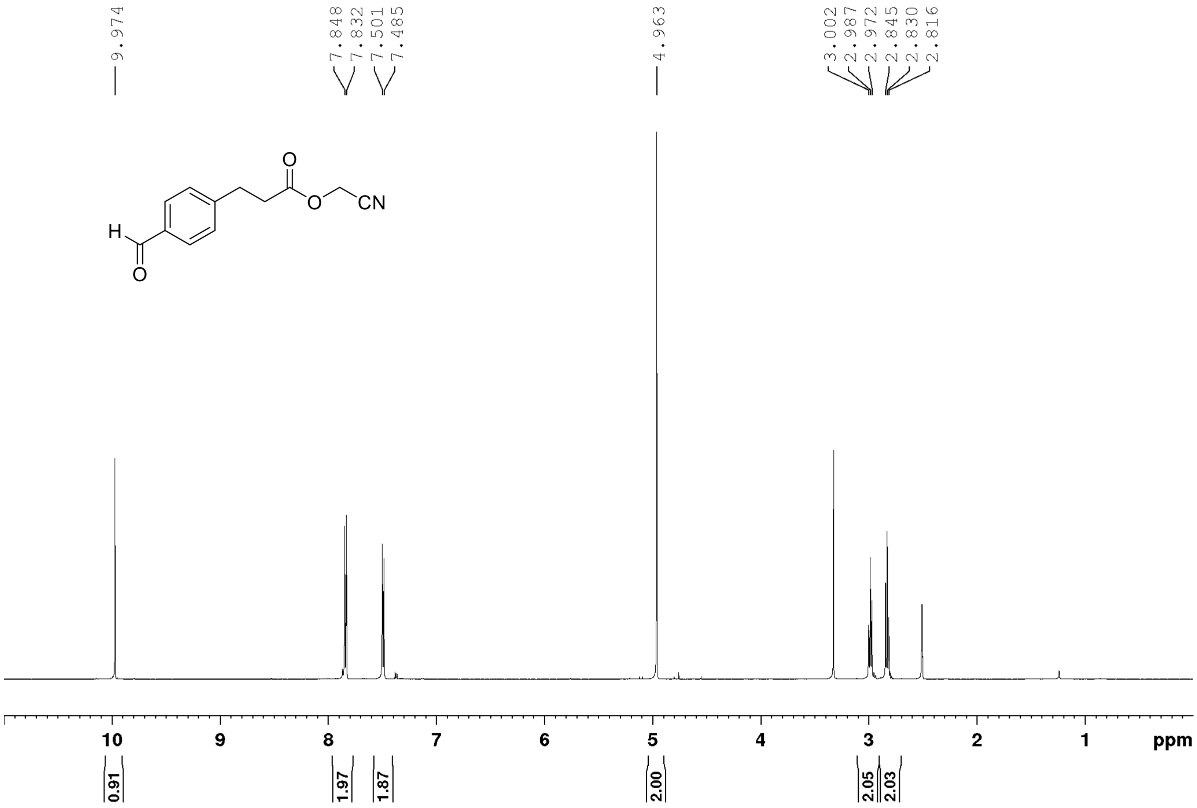

Supplementary Figure 104. ^{13}C NMR (125 MHz, DMSO-d_6) of **35.**

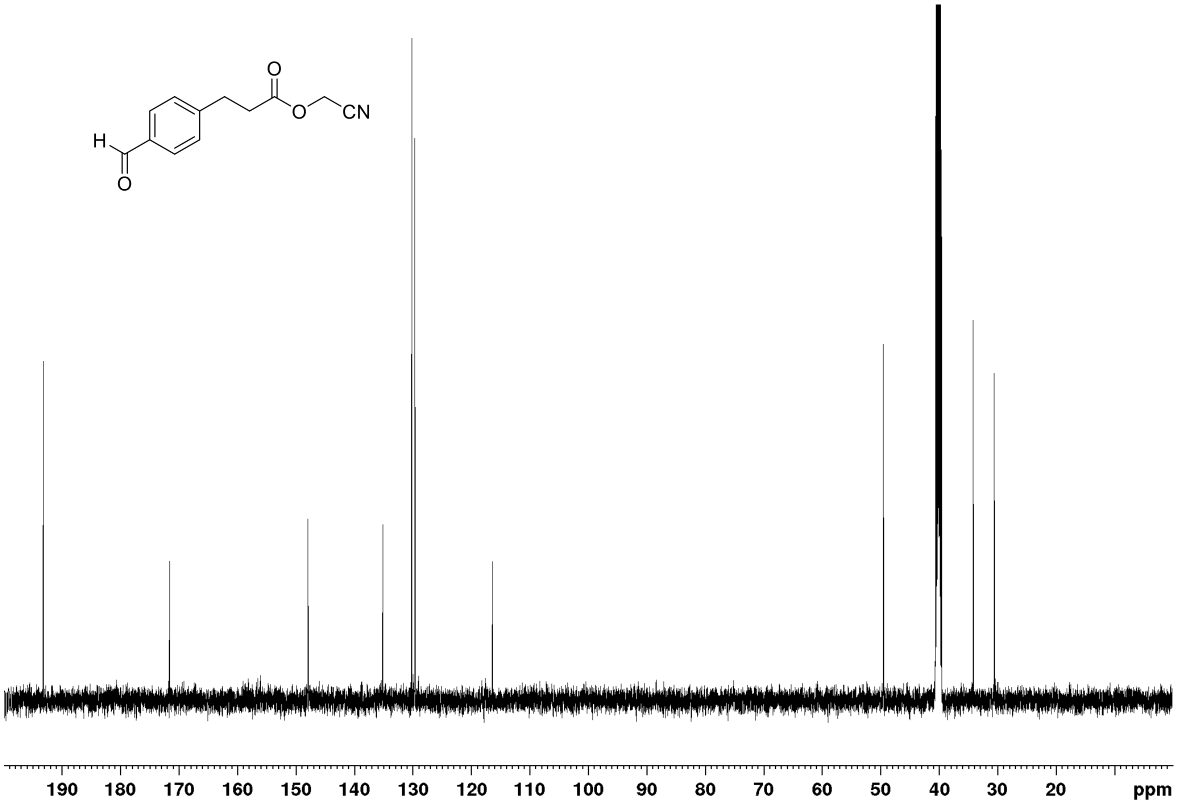

Supplementary Figure 105. ^{1}H NMR (500 MHz, DMSO-d_6) of **36.**

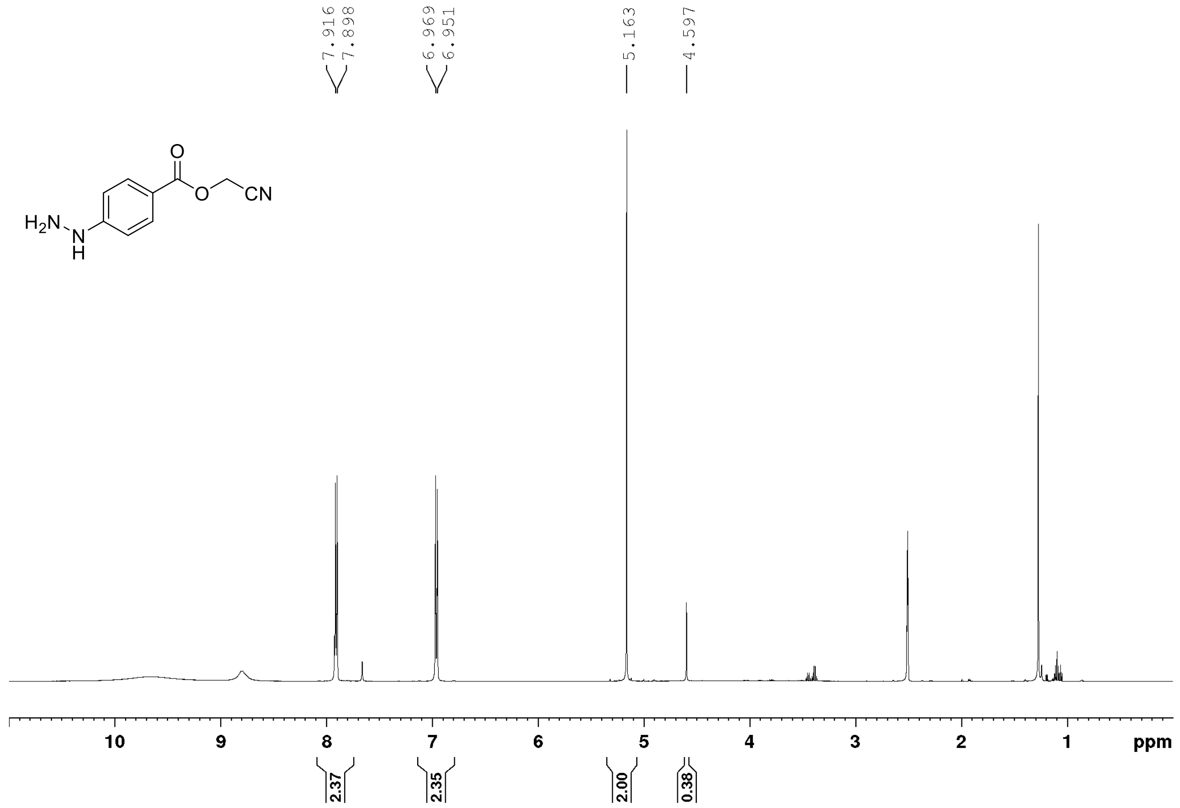

Supplementary Figure 106. ^{13}C NMR (125 MHz, DMSO-d_6) of **36.**

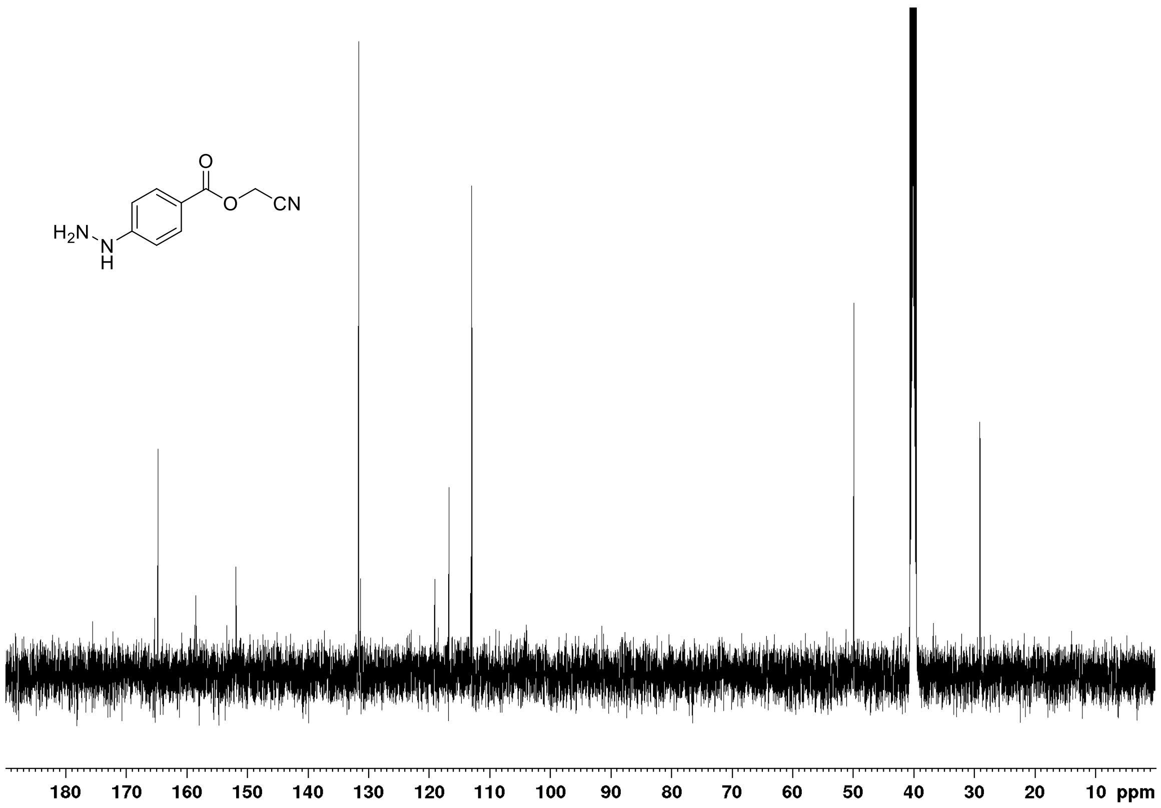

Supplementary Figure 107. ^{1}H NMR (400 MHz, $CDCl_3$) of Boc-**37.**

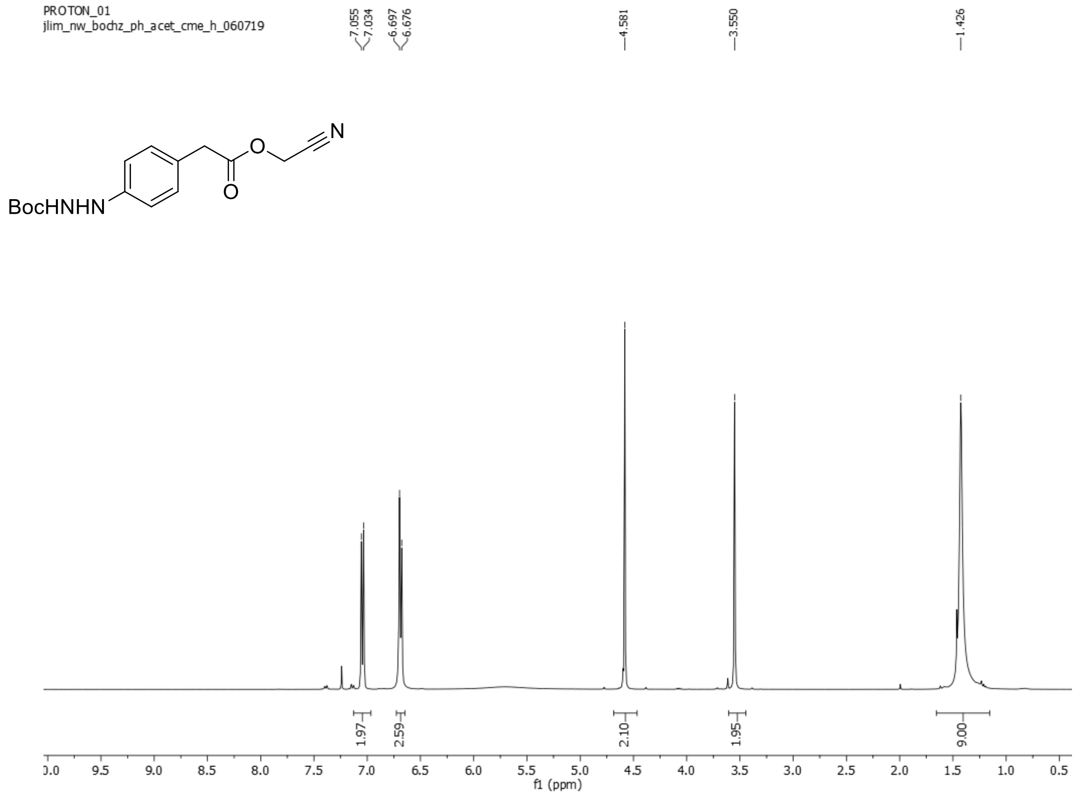

Supplementary Figure 108. ^{13}C NMR (100 MHz, $CDCl_3$) of Boc-**37.**

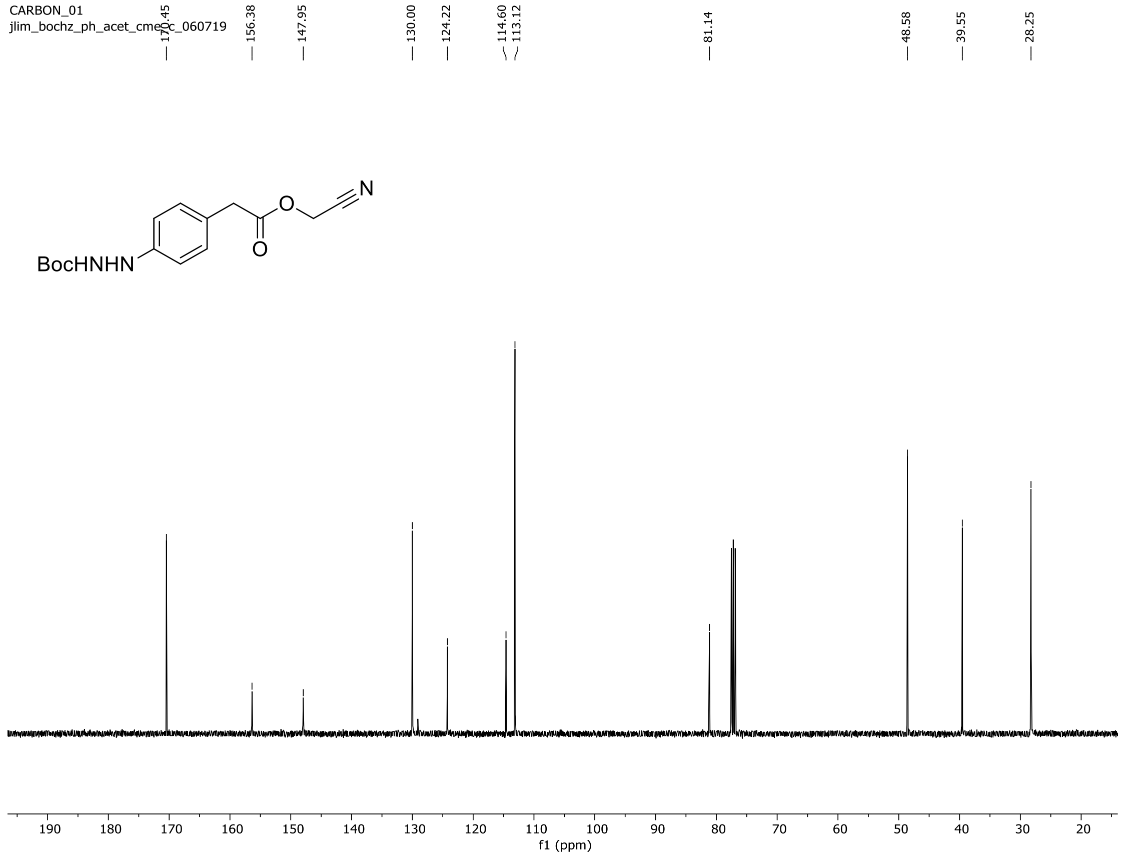

Supplementary Figure 109. ¹H NMR (400 MHz, $CDCl_3$) of Boc-**38.**

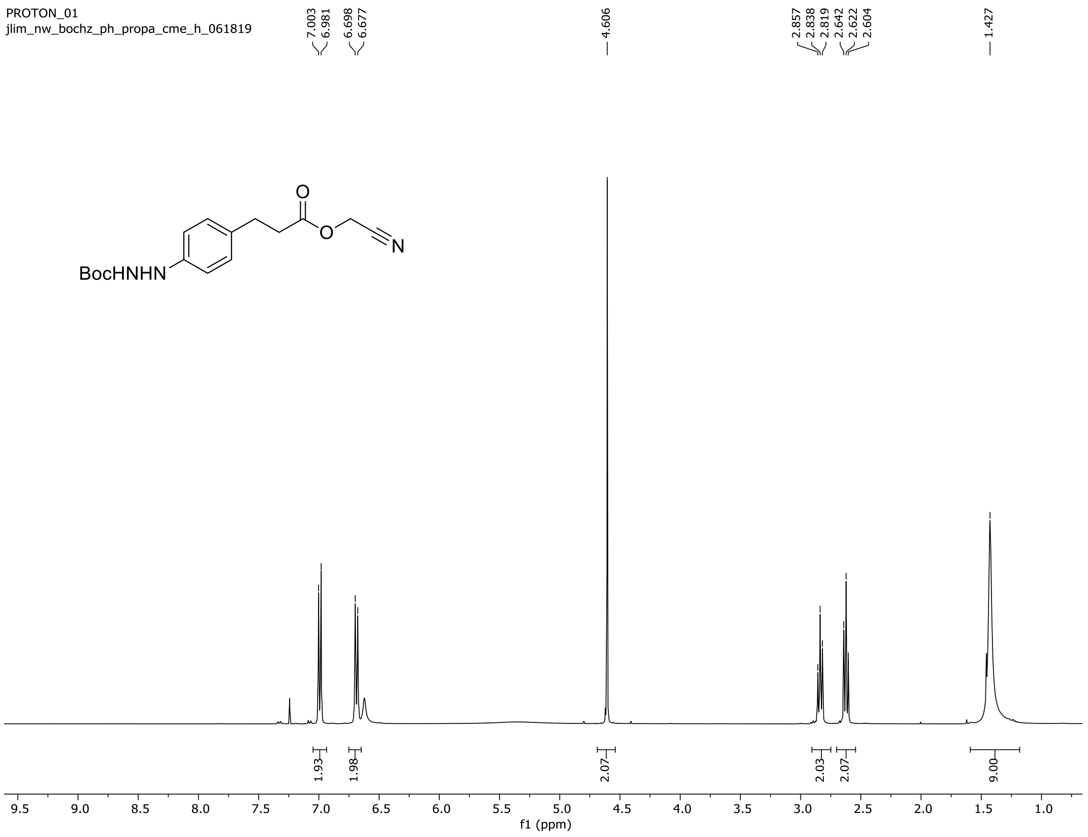

Supplementary Figure 110. ¹³C NMR (100 MHz, $CDCl_3$) of Boc-**38.**

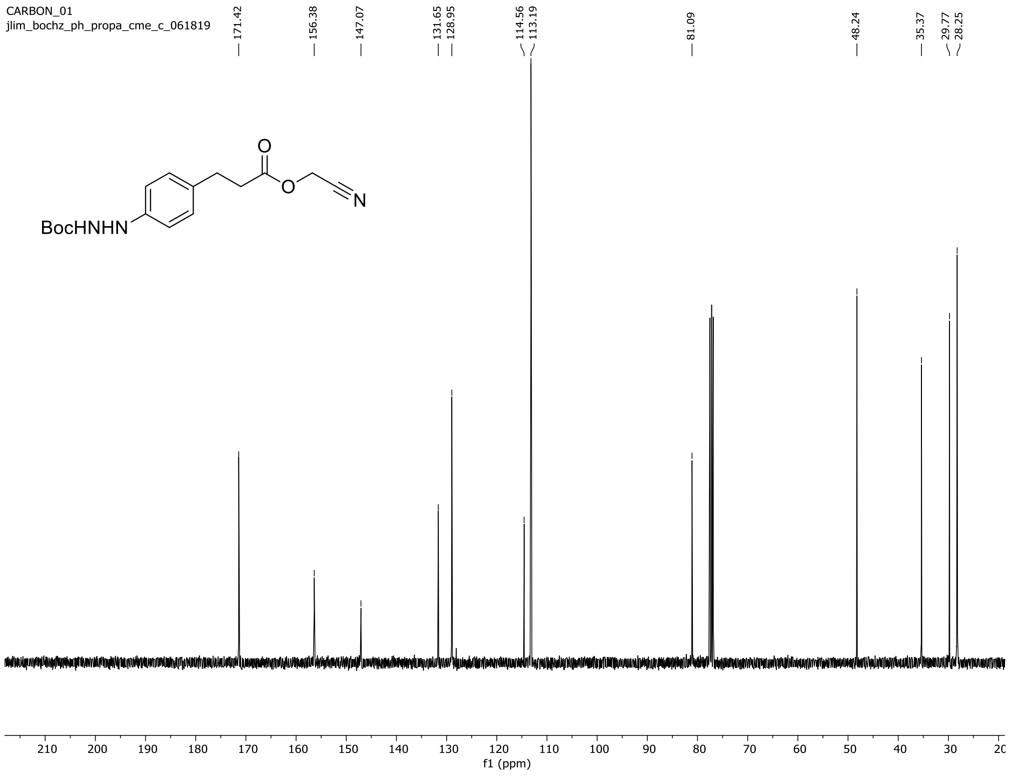

Plasmid map

>pJL1_StrepII

GGATCCTGCAGTTGAGATCCTTTTTTTCTGCGCGTAATCTGCTGCTTGCAAACAAAAAAACCACCGCTACCAGCGGTGGTTTGTTTGCCGGATCAAGAG
CTACCAACTCTTTTTCCGAAGGTAACTGGCTTCAGCAGAGCGCAGATACCAAATACTGTCCTTCTAGTGTAGCCGTAGTTAGGCCACCACTTCAAGAAC
TCTGTAGCACCGCCTACATACCTCGCTCTGCTAATCCTGTTACCAGTGGCTGCTGCCAGTGGCGATAAGTCGTGTCTTACCGGGTTGGACTCAAGACGA
TAGTTACCGGATAAGGCGCAGCGGTCGGGCTGAACGGGGGGTTCGTGCACACAGCCCAGCTTGGAGCGAACGACCTACACCGAACTGAGATACCTACAG
CGTGAGCATTGAGAAAGCGCCACGCTTCCCGAAGGGAGAAAGGCGGACAGGTATCCGGTAAGCGGCAGGGTCGGAACAGGAGAGCGCACGAGGGAGCTT
CCAGGGGGAAACGCCTGGTATCTTTATAGTCCTGTCGGGTTTCGCCACCTCTGACTTGAGCGTCGATTTTTGTGATGCTCGTCAGGGGGGCGGAGCCTA
TGGAAACGAATTCAGATCTCGATCCCGCGAAATTAATACGACTCACTATAGGGAGACCACAACGGTTTCCCTCTAGAAATAATTTTGTTTAACTTTAAG
AAGGAGATATA[***CATATG*****TGGTCTCATCCGCAGTTCGAAAAATCCACCTAGTAA*****GTCGAC*]CGGCTGCTAACAAAGCCCGAAAGGAAGCTGAGTTGGCT
GCTGCCACCGCTGAGCAATAACTAGCATAACCCCTTGGGGCCTCTAAACGGGTCTTGAGGGGTTTTTTGCTGAAAGCCAATTCTGATTAGAAAAACTCA
TCGAGCATCAAATGAAACTGCAATTTATTCATATCAGGATTATCAATACCATATTTTTGAAAAAGCCGTTTCTGTAATGAAGGAGAAAACTCACCGAGG
CAGTTCCATAGGATGGCAAGATCCTGGTATCGGTCTGCGATTCCGACTCGTCCAACATCAATACAACCTATTAATTTCCCCTCGTCAAAAATAAGGTTA
TCAAGTGAGAAATCACCATGAGTGACGACTGAATCCGGTGAGAATGGCAAAAGCTTATGCATTTCTTTCCAGACTTGTTCAACAGGCCAGCCATTACGC
TCGTCATCAAAATCACTCGCATCAACCAAACCGTTATTCATTCGTGATTGCGCCTGAGCGAGACGAAATACGCGATCGCTGTTAAAAGGACAATTACAA
ACAGGAATCGAATGCAACCGGCGCAGGAACACTGCCAGCGCATCAACAATATTTTCACCTGAATCAGGATATTCTTCTAATACCTGGAATGCTGTTTTC
CCGGGGATCGCAGTGGTGAGTAACCATGCATCATCAGGAGTACGGATAAAATGCTTGATGGTCGGAAGAGGCATAAATTCCGTCAGCCAGTTTAGTCTG
ACCATCTCATCTGTAACATCATTGGCAACGCTACCTTTGCCATGTTTCAGAAACAACTCTGGCGCATCGGGCTTCCCATACAATCGATAGATTGTCGCA
CCTGATTGCCCGACATTATCGCGAGCCCATTTATACCCATATAAATCAGCATCCATGTTGGAATTTAATCGCGGCTTCGAGCAAGACGTTTCCCGTTGA
ATATGGCTCATAACACCCCTTGTATTACTGTTTATGTAAGCAGACAGTTTTATTGTTCATGATGATATATTTTTATCTTGTGCAATGTAACATCAGAGA
TTTTGAGACACAACGT

***CATATG*: NdeI**

***GTCGAC*: SalI**

TGGTCTCATCCGCAGTTCGAAAAA: strep tag

>pJL1_StrepII

[*CATATG*TGGTCTCATCCGCAGTTCGAAAAATCCACCTAGTAA*GTCGAC*]

fMetTrpSerHisProGlnPheGluLysSerThr

References

1. Pangborn, A.B., Giardello, M.A., Grubbs, R.H., Rosen, R.K. & Timmers, F.J. Safe and convenient procedure for solvent purification. *Organometallics* **15**, 1518-1520 (1996).
2. West, J.B. et al. Enzymes as Synthetic Catalysts - Mechanistic and Active-Site Considerations of Natural and Modified Chymotrypsin. *J Am Chem Soc* **112**, 5313-5320 (1990).
3. Niwa, N., Yamagishi, Y., Murakami, H. & Suga, H. A flexizyme that selectively charges amino acids activated by a water-friendly leaving group. *Bioorg Med Chem Lett* **19**, 3892-3894 (2009).
4. Goto, Y., Katoh, T. & Suga, H. Flexizymes for genetic code reprogramming. *Nat Protoc* **6**, 779-790 (2011).
5. Tuckey, C., Asahara, H., Zhou, Y. & Chong, S. Protein synthesis using a reconstituted cell-free system. *Curr Protoc Mol Biol* **108**, 16 31 11-22 (2014).
